# A systematic review of passing fit testing of the masks and respirators used during the COVID-19 pandemic: Part 1-quantitative fit test procedures

**DOI:** 10.1371/journal.pone.0293129

**Published:** 2023-10-26

**Authors:** Anahita Fakherpour, Mehdi Jahangiri, Janis Jansz

**Affiliations:** 1 Student Research Committee, Department of Occupational Health and Safety Engineering, School of Health, Shiraz University of Medical Sciences, Shiraz, Iran; 2 Department of Occupational Health and Safety Engineering, Research Center for Health Sciences, School of Health, Shiraz University of Medical Sciences, Shiraz, Iran; 3 School of Mines: Minerals, Energy and Chemical Engineering, Faculty of Science and Engineering, Curtin University, Perth, Australia; Hanyang University - Seoul Campus: Hanyang University, REPUBLIC OF KOREA

## Abstract

**Background:**

During respiratory infection pandemics, masks and respirators are highly sought after, especially for frontline healthcare workers and patients carrying respiratory viruses. The objective of this study was to systematically review fit test pass rates and identify factors influencing the fitting characteristics.

**Methods:**

Potentially relevant studies were identified using PubMed, Scopus, Web of Science, and Science Direct during the COVID-19 pandemic from February 5, 2020, to March 21, 2023. The search strategy using the following keywords was conducted: Quantitative Fit Test, Condensation Nuclei Counter, Controlled Negative Pressure, PortaCount, Sibata, Accufit, Fit, Seal, Mask, Respirator, Respiratory Protective Device, Respiratory Protective Equipment, Protective Device, Personal Protective Equipment, COVID-19, Coronavirus, and SARS-CoV-2. The quality of the included studies was also assessed using the Newcastle-Ottawa scale.

**Results:**

A total of 137 articles met the eligibility criteria. Fifty articles had a quality score of less than 7 (good quality). A total of 21 studies had a fit test pass rate of less than 50%. 26 studies on disposable respirators and 11 studies on reusable respirators had an FF of less than 50 and less than 200, respectively. The most influential factors include respirator brand/model, style, gender, ethnicity, facial dimensions, facial hair, age, reuse, extensive movement, seal check, comfort and usability assessment, and training.

**Conclusion:**

37.36% of the disposable respirator studies and 43% of the reusable respirator studies did not report fit test results. 67.86% of the disposable respirator studies had a fit test pass rate greater than 50%, and 35.84% of these studies had an FF greater than 100. Also, 85.71% of the reusable respirator studies had a fit test pass rate greater than 50%, and 52.77% of these studies had an FF greater than 1000. Overall, the fit test pass rate was relatively acceptable. Newly developed or modified respirators must undergo reliable testing to ensure the protection of HCWs. Subject and respirator characteristics should be considered when implementing fit testing protocols. An optimal fit test panel should be developed prior to respirator design, certification, procurement decisions, and selection procedures.

## Introduction

According to the hierarchy of controls, respiratory protective equipment (RPE) usage is inevitably considered one of the preventive and controlling measures during the COVID-19 pandemic [1]. There has been a strong demand for N95 filtering facepiece respirators (FFRs) and surgical masks during respiratory infection pandemics, particularly for the frontline healthcare workers (HCWs) who are exposed to high-risk aerosol-generating procedures (AGPs), including incubation, bronchoscopy, manual ventilation, open suctioning, and high speed drilling in dental procedures, whether through potential contact, droplet, or airborne transmission, and for the patients seeking care who may be potentially transmitting the respiratory viruses through the air [2–5].

The optimal performance of the respirators is dependent on both filtration efficiency and fitting characteristics. Meanwhile, these two main factors warranted the users’ protection by reducing the emission and spread of viral respiratory pathogens through airborne droplets and aerosols and reducing the inhalation of airborne respiratory contaminants (viruses, chemical agents, etc.) [6, 7]. The fit testing procedures are of great importance in international regulations and standards [8–12]. Filtration efficiency determines how well proposed masks or respirators’ filter media filter particles containing viruses, bacteria, and other contaminants [13]. The respirator fitting represents the fitting of a mask or respirator into anthropometric facial dimensions such that there are no gaps or air contaminant leaks between the sealing surface area of the skin and the facepiece [13, 14]. Furthermore, a respirator with a higher filtration efficiency might provide less respiratory protection compared to a respirator with a lower filtration efficiency. In this case, air preferentially passes through the face-seal area due to its lower resistance than the filter media [15].

The respirator fit testing procedure is one of the key elements of the respiratory protection program (RPP), with the aim of selecting a well-fitting respirator with a specific make, model, style, and size. To do so, it is required to provide various sizes, styles, brands, and models to ensure the users’ utmost protection. Overall, two fit testing procedures are classified as qualitative fit testing (QLFT) and quantitative fit testing (QNFT). The QLFT is a dichotomous test based on subjective response to a challenge agent with a distinctive taste or odor. The QNFT is an objective technique (FF) that involves measuring the ratio of challenge agent concentration inside the respirator (C_in_) to its concentration outside the respirator (C_out_) while conducting the same set of exercises [8–12].

Overall, the investigations revealed that users were mainly concerned with the filter media used for masks and respirators’ making processes (spun bond, melt blown, nanofiber, etc.) and their expected level of filtration efficiency; therefore, less attention was paid to the mask/respirator fitting characteristics during the COVID-19 pandemic [16, 17]. Recent evidence highlights the utmost importance of fit testing adoption to assure the effectiveness of respirators, which might boost regulatory compliance and break the COVID-19 transmission chain [18].

The effectiveness of masks and respirators, as well as decontamination and reprocessing strategies, have been investigated in certain systematic reviews and meta-analyses; however, the fitting characteristics have not yet undergone a thorough evaluation [19–27]. Only one meta-analysis was conducted by Chopra et al. (2021) to examine the influence of ethnicity and gender on respirator fitting [28]. In the current study, we systematically reviewed the studies performed on respirator fitting and affective factors during COVID-19. On the other side, we investigated which countries adopted or implemented respirator fit testing protocols during the COVID-19 pandemics? What were the overall passing rates? Which factors (subjects and respirator features) could significantly affect the fitting capability? Furthermore, we assessed which types of RPE and QNFT protocols were preferably used and then considered possible challenges and limitations obtained during the fit testing. Lastly, we reviewed the quality level of the included studies and summarized their strengths and weaknesses.

Accordingly, this study might serve to emphasize the significance of respirator fitting and also be useful in adopting measures for RPE design and production, revising fast and affordable fit testing protocols, and developing respiratory protection guidelines for potential future pandemics.

## Methods

### Ethical statement

The current study was approved by the ethics committee of Shiraz University of Medical Sciences (IR.SUMS.SCHEANUT.REC.1400.093).

### Search strategy

This work was conducted following the Preferred Reporting Items for Systematic Reviews and Meta-Analyses (PRISMA) guidelines 2020 (http://www.prisma-statement.org/) [29]. See S1 Appendix-PRISMA 2020 Checklist. A comprehensive search for primary literature using five databases, including PubMed (https://pubmed.ncbi.nlm.nih.gov/), Scopus (https://www.scopus.com/), Web of Science (https://www2.wosgs.ir/wos/woscc/advanced-search), Science Direct (https://www.sciencedirect.com/), one scientific website named “Centers for Disease Control and Prevention” (https://www.cdc.gov/), and one scientific journal named “The International Society for Respiratory Protection” (https://www.isrp.com/) during the COVID-19 pandemic from February 5, 2020 to March 21, 2023. To take into account all references cited in the studies, the researchers manually searched the reference lists of the retrieved articles.

Also, the grey literature search was performed using the Google Scholar (https://scholar.google.com/), Google (http://google.com/) search engine, ProQuest (https://www.proquest.com/), Medrxiv (https://www.medrxiv.org/search), OpenGrey (https://onlinelibrary.london.ac.uk/resources/databases/opengrey), and Epistemonikos

(https://www.epistemonikos.org/), Morbidity and Mortality Weekly Report (MMWR)-CDC (https://www.cdc.gov/mmwr/index.html), Wiley Online Library (https://onlinelibrary.wiley.com/), Springer link (https://link.springer.com/), and Nature (https://www.nature.com/) to ensure further studies or relevant electronic documents might not to have been missed. Search terms included (Mask OR Respirator OR Personal Protective Equipment OR respiratory protective device OR Protective Device, Respiratory Protective Equipment, Respiratory Protective Device) AND (Quantitative Fit Test, Condensation Nuclei Counter, Controlled Negative Pressure, PortaCount, Sibata, Accufit, Fit, Seal), AND (COVID-19, Coronavirus, and SARS-CoV-2). Meanwhile, the Medical Subject Headings (MeSH) term, including “Respiratory Protective Device,” was applied to enhance the search and include associated synonyms in the search. The search strategy and excluded articles were provided in S2 Appendix.

#### Study selection and eligibility

All documents, including original articles, letters, and reports related to the QNFT procedures and affective factors (subject characteristics and respirator features), were included in the research. We excluded book chapters, review articles, meta-analyses, and guidelines. A total of 137 full texts fulfilled the eligibility criteria.

### Data extraction and study quality assessment

Two reviewers (A.F & M.J) independently screened the titles and abstracts of all studies obtained from the comprehensive search. In the next step, two reviewers (A.F & M.J) independently retrieved the full-texts of the included studies, reviewed them, and selected the final studies. Afterwards, the study data, including the first author, number of study subjects, respirator features (type, brand, model, size, and style), subject characteristics (gender: female or male, occupation: HCWs or non- HCWs), country, type of standard QNFT procedure (including, Occupational Safety and Health Administration (OSHA), American National Standards Institute (ANSI), Health and Safety Executive (HSE), International Organization for Standardization (ISO), European Standard (EN), The National Institute for Occupational Safety and Health (NIOSH), Australian/New Zealand (AS/NZS), Canadian Standards Association (CSA), etc.), respirator fit tester (PortaCount, Sibata, etc.), fit test failure or pass rate by respirator brand, model, style, and gender of subjects, and where possible, the relationships between the factors influencing the fit testing and mask or respirator fitting were noted in the study extraction tables (Tables 1–2). All studies obtained during the search strategy, screening, and selection process were imported into EndNote X9 software.

**Table 1:** Quantitative fit testing of disposable masks or respirators and affective factors.

Study	Respirator Features (Brand, model, size, style)	Subject Characteristics	Findings
Brandel et al., 2020 [123]	DIY homemade mask	One male and one female mannequin heads	The developed mask minimized the air flow around the edge and had a FF≥2 (acceptable range: 1.5–2).
Buckley et al., 2020 [146]	HensNest face mask	A single test subject	The FFs for the HensNest (HEPA, 1 ply): 8, HensNest (HEPA, 3 ply): 23, HensNest (Grocery Bag): 4, HensNest (Coffee Filter), Surgical mask, Sewn mask, and Bandana: 1, and HensNest (Tea Towel): 3, were low in comparison to those of the N95 respirator (83).
Coyle et al., 2022 [147]	Three-ply cotton mask	Source simulator and one subject	The FFs of the 3-ply cloth mask were 4.1 ± 2.6 (n = 43) for the recipient and 1.7 ± 0.6 (n = 42) for the source simulator.
Dang et al., 2021 [148]	A novel sewn Glidden mask	Six volunteers	Four out of six volunteers passed the Glidden mask; two failed subjects had FFs of 20 per H600-Filti-H600 (in three layers) and 98 per H600-H600 (in two layers). This mask demonstrated the intermediate protection between a surgical mask and N95 FFR. The mask size, material stiffness, and plasticity could influence the fitting.
Drouillard et al., 2022 [39]	Fifty-two cotton fabrics	Two testers (One male and one female)	The FFEs of the tested masks were significantly different (p<0.001). The mean control medical mask had an FFE of 55.3±2.1%. The best-performing fabric mask was WP036 (a bed sheet) with a mean FFE of 65.6±4.6%, followed by WP028 (tea towel; 65.0±1.4%) and WP047 (batik; 64.3±0.7%). The overall FF for all studied masks (fabric masks or medical masks) ranged from 1.6–3.0, and the FFE ranged from 39.81–65.57.
**Study**	**Respirator Features (Brand, model, size, style)**	**Subject Characteristics**	**Findings**
Duncan et al., 2021 [149]	Reusable cloth face masks, disposable procedure masks, KN95 masks and N95 respirators, Fabric 2-layer masks, Multi-layer masks, Disposable procedure/ surgical masks, KN95 masks, and N95 masks	8–26 volunteers (Males, females)	The GMD for the TILPF was measured for the N95 FFR: 165.7, for the KN95: 6.2, for the procedure mask: 2.26, for the multi-layer: 1.77, and for the fabric 2-layer: 1.42.
Mueller et al., 2020 [150]	Sewn fabric face masks and standard surgical masks	One female subject	The N95-1 mask had an FF of 126 (∼99.2%) and the N95-2 had an FF of 10.6 (∼90.6%). The FF for the surgical mask ranged from 57–67 (FFE: 50%–75%). The mean FF for the cloth surgical-style mask was 63, mean FFE: 58.6%, and for the fabric surgical-style mask was FF: 63.36, mean FFE: 57.84%. The mean FF for the fabric cone-shaped masks was 53.71, mean FFE: 86.20%, and also for the duck-bill-shaped mask, FF: 60.90, mean FFE: 64.2%. The nylone overlayer increased the mean particle removal efficiency.
Reutman et al., 2021 [151]	Homemade face mask	Ten subjects (Six males, four females)	The overall “adopted” fit factor (aFF) ranged from 1–27 for the prototyped mask (overall aFF: 7.1 ± 1.6), 2–196 for the mask A (overall aFF: 12.6 ± 4.4), and 1–9 for the mask B (overall aFF: 2.5±3.2). A significant difference was observed in overall aFF between subjects and within subjects. A highly significant difference was observed between the mask types (p < 0.001).
Teesing et al., 2020 [119]	25 materials	Laboratory setup and one female subject	The highest FF for the 3M FFP21862+ was 134, Duckbill with a seam on the inside (ePM₁ 85%) was 130, Duckbill with a seam on the outside (ePM₁ 85%) was 120, respectively. All remaining fabrics had a lower FF range of 8–79. The duckbill fabric filter mask would provide a better fit than the surgical mask (FF:4). Two layers of quilt fabric with a household paper towel could be adequate for users’ protection.
**Study**	**Respirator Features (Brand, model, size, style)**	**Subject Characteristics**	**Findings**
Wentworth et al., 2020 [120]	Homemade masks	Laboratory set up	N95 UV 2 Cycle Test#3, N95 UV 2 Cycle Test#4, N95 Plasma Test, N95 Plasma Test #2, N95 Plasma Test #1, Yellow Surgical Mask Ntension Surgical Mask Prototype Double Layer/ Single Layer, Strainrite provided White or Grey PolyPro SB, and Ntension Prototype 2 Test had an FF of 200 as well as the N95 mask.
Lindsley et al., 2021 [126]	Procedure mask, cloth mask, neck gaiter, and face shield	Headform	The FFs for the procedure mask were 2.9 (0.5), for the cloth mask: 1.3 (0.1), for the Neck gaiter (single layer): 1.7 (0.5), for the Neck gaiter (double layer): 1.9 (0.4), and for the N95 respirator:198 (3.5). The N95 respirator outperformed compared to the remaining device (p< 0.0001).
Sato et al., 2020 [95]	Four masks, including Japan Medical Products Co HopesVR face mask (JM-28C) Pleated-type nonactivated carbon mask, NisshoSangyo Co NS surgical mask (14732), KOKEN LTD Hirac (type 350) Pleated-type activated carbon mask, KOKEN LTD Hirac (type 350) cup-type nonactivated carbon mask, and KOKEN LTD MaskyMD cup-type activated carbon mask	Four pharmacists (Two males, two females)	The leakage rate and particle reduction rate were achieved: 14.8±10.5, 70.8±11.3, per the Pleated-type nonactivated carbon mask, 34.8±26.9, 48.5±28.4, per the Pleated-type activated carbon mask, 0.3±0.4, 99.3±0.7, per cup-shape nonactivated carbon mask, 5.6±19.4, 33.6±10.9, per the cup-shape activated carbon mask. The cup-shape respirators, particularly the activated carbon type, were the most effective.
Ardon-Dryer et al., 2021 [49]	HDX N95 respirator, AOXING and ARUN KN95 respirator, NANO KN95 respirator, 3D-printed Montana Mask equipped with MERV 13-AIRx, MERV 13-H, and HEPA filters	Manikin headform	All N95 and KN95 respirators passed the fit tests. The Montana masks with any of the three filters failed the fit tests. Also, homemade duckbill masks made from the Halyard H600 sterilization wrap and WypAll X80 reusable wipe failed the fit tests.
**Study**	**Respirator Features (Brand, model, size, style)**	**Subject Characteristics**	**Findings**
Bodas et al., 2022 [108]	Two flat-fold BYD DE2322 N95 and Care Essentials (CE) MSK-002 P2 masks	300 participants, including HCWs or employees (87 males, 205 females)	The fitting of the MSK-002 mask (57%) with an FF of ∼200 was significantly higher than the BYD DE2322 mask (18%) with an FF of ∼70, p <0.001. The overall subjective rating for the CE MSK-002 was significantly higher for the BYD (p<0.001).
Cameron et al., 2020 [74]	Five respirators including 3M 1860 and 1860S N95, ProShield TN01–11, TN01–12 N95 respirators, and 3M Aura 1870+ mask	371 HCWs	23 (6.2%) subjects failed the first four masks, and 6 (1.6%) failed all five masks. The 3M 1860S had the highest (18.2%) failure rate.
Chan et al., 2021 [76]	Seven types of P2/N95 respirators, including 3M 8210, 3M 8110S, 3M 1860, 3M 1860S, Proshield N95 ‘duckbill’ respirator, Halyard N95 duckbill respirator (standard), Halyard N95 duckbill respirator (small)	59 HCWs (23 males, 36 females)	The fit test failure rate was (69% (40/58)) for the first selection of N95/P2 respirators. The 3M^TM^ 1860 respirator had the highest passing rate (67%) and the Halyard small duckbill had the lowest passing rates (8%). Also, 69% of the respirators failed the QNFT. The seal checks could not detect the respirator fitting capability (PPV: 34.1%, 95%CI: 25.0–40.5). The sensitivity, specificity, positive predictive value, and negative predictive value for the seal check were as follows: Se: 77.8%, Sp: 32.5%, PPV: 34.1%, and NPV: 76.5%. The median [IQR] FFs were calculated: 3M 1860: 148 (50–200), 3M 8210: 89 (46–200), 3M 1860S: 69.5 (10–86), 3M 8110S: 61.5 (50–80), Halyard (small): 29 (18–124), Proshield: 21 (10–50), and Halyard (regular): 17 (7–36). Also, the subjects’ perception improved from 39.2% (20/51), pre-test (before fit testing) to 81.8% (36/44), post-test (after fit testing).
**Study**	**Respirator Features (Brand, model, size, style)**	**Subject Characteristics**	**Findings**
Christopher et al., 2021 [89]	3M1860 N95 respirators (regular/small size)	305 staff of Health service center (110 males, 195 females)	Failure rates of fit testing in females were significantly higher than those in males due to being small-boned (6.67% vs. 2.72%; p<0.0001). The reasons for the fit test failure of females were considered mainly due to small bone structure and for males due to facial hair.
Cloet et al., 2022 [91]	Three masks, including MNmask v1 (small, medium, large), MNmask v2 (small, medium, large), and KN95	Nine female dental students	The FFs were obtained for the MNmask v1: 93.3, the MNmask v2: 438.0, and the KN95 mask: 4.9. The results of the activity and usability assessments indicated that the KN95 had the highest usability score due to its loose-fitting. MNmask v2 had higher usability scores (subjective discomfort, wear efficiency, and speech intelligibility), and breathability than the MNmask v1; however, it obtained lower stability. Because the paracord bands in MNmask v2 resulted in higher wear efficiency, but lower stability score.
Cloet et al., 2022 [90]	Three N95 FFRs, including MNmask v1, MNmask v2, and KN95 respirators	Nine female Dental students	The passing rates and mean FFs were obtained for the MNmask v1: 22.22%, 93.32+141.35, MNmask v2: 77.78%, 438.0+436.15, KN95: 0%, 4.86±2.15. The subjects scored the MNmask v1 as the most fitting (level of confidence in the mask seal) and the MNmask v2 as the most stable mask. Factors such as fit, comfort, material, and design are vital to solving the users’ challenges. The nose wire and nose discomfort, foam and chin and cheekbone discomfort, bands and head and neck discomfort, and filter material and skin discomfort (symptoms of rash, skin indentations, and itching) are crucial to take into account.
Griffin et al., 2022 [152]	Four novel masks (MNmask v1, MNmask v2, MNmask Reusable, and MNmask Procedural)	Nine participants (Four males, five females)	Eight of nine (88.89%) participants passed the N95 3M Aura 9210^+^ mask (mean ± S.D FF: 220.9±169.2), seven participants (77.78%) passed the MNmask v2 (mean ± S.D FF: 438.0±436.1). Five participants (55.56%) passed the N95 3M 1860 mask (mean ± S.D FF: 89.6±45.4). Two participants (22.22%) passed the MNmask v1 mask (mean ± S.D FF: 93.3 ±141.3).
**Study**	**Respirator Features (Brand, model, size, style)**	**Subject Characteristics**	**Findings**
Duncan et al., 2020 [133]	Surgical-style 3M 1870 N95 FFR	Eight subjects	The initial GRPF and the end-of-day GRPF ranged from 100–426 and 13–169, respectively. The SWPFs for all the test subjects ranged from 10–84. On a day-to-day basis, as the number of reuses increased, the GRPF increased or decreased relative to the day prior. The GRPF for all test subjects was less than the initial GRPF after 18–19 wears on day 5 (p<0.05).
Fabre et al., 2021 [68]	3M 1860 N95 FFRs (Dome shaped) and 3M1870 N95 FFRs (Duck-bill shaped)	92 Physicians/ Advanced practitioners (15 males, 77 females)	Five out of 16 N95 fit failures (31%) were identified by seal check. 18 N95 respirators failed one or more screening tests; 16 (89%) of them failed the PortaCount QNFT (overall failure rate: 17%). 83% of the N95s were effective, as they passed the fit test of the 3M N95s after a median of 40 donnings by the HCWs.
Nakamoto et al., 2021 [70]	Three N95 respirators, including Duckbill-shaped HPR-R/HPR-S, dome-shaped Hi-Luck 350, and three-panel flat-fold 9211 respirators	41 participants, including 24 doctors and 17 nurses (16 males, 25 females)	The overall fit testing passing rate for reusing three styles of N95 respirators was 35 (85.4%). There were no significant differences among the studied N95 respirators. The fit test passing rate was constant after the first week of reuse (fit test 1): overall: 41 (100%), Duckbill-shape: 23 (100%), Cup: 10 (100%), and Three-panel flat-fold: 8 (100%). The fit passing rates after second week reuse (fit test 2): overall: 37 (90%), Duckbill-shape: 21 (91%), Cup: 10 (100%), Three-panel flat-fold: 6 (75%), and after third week reuse (fit test 3): overall: 35 (85%), Duckbill-shape: 19 (82%), Cup: 10 (100%), and Three-panel flat-fold: 6 (75%).
Greenawald et al., 2021 [131]	Five FFRs, including 3M 1870 N95, 3M 8210 N95, 3M 9010 N95, 3M 1860 N95 surgical mask, and KIMBERLY-CLARK N95 (KC) 46827 Surgical Mask	25 volunteers	The FFs of both 3M 1870 and 3M 9010 respirators and each stockpiled Lot were not significant. Higher proportions passed the 3M 1860 Lot C compared to the control (81% vs. 58%; p<0.04). The 3M 8210 Lot B had lower passing rates than the control (32% vs. 76%; p<0.002). The passing rates of KC 46827 Lots A, B, and C were lower than the control (9.0%, 8.0%, and 9.0% vs. 58%).
**Study**	**Respirator Features (Brand, model, size, style)**	**Subject Characteristics**	**Findings**
Hai et al., 2022 [94]	Four FFRs including surgical mask, double mask (cloth mask on top normal surgical mask), N95 mask, New innovative stick-on mask, LEKAD.	One subject (Physiotherapy lecturer)	Among all, the stick-on mask Lekad compared to the other FFRs, obtained a high FF≥200. The FF were measured for the N95 mask: 6.5, for the Double mask: 5.75, and for the normal surgical mask: 5. There were significant differences among the FFRs (p = 0.012).
Han et al., 2021 [77]	Three brands of the N95 respirators, including 3M 8210 (free size), Halyard Health N9586727/86827 (medium size), and Dobu 201 (medium size)	183 HCWs, including nurses in intensive care units, emergency medical centers, and nationally designated isolation treatment beds (Four males, 179 females)	The overall passing rate and FF for the 3M were 46 (50%), 82.95±69.38, Halyard Health were 16 (33.3%), 104.64±54.36, and Dobu were 1 (2.3%), 17.64±20.52, (p<0.001).
Hwang et al., 2020 [71]	Three N95 FFRs, including 3M 1870+, 3M 1860, Kimberly Clark 46727	44 HCWs, including medical doctor, Nurse, Emergency medical technician (15 males, 29 females)	All subjects passed the fit test. 32 subjects (73%) failed the fit test for at least one of the three chest compressions. Also, a significant difference was noted between the PPG and the APG (94% vs. 61%, p = 0.02). Also, 8 (18%) of the subjects experienced strap loosening (5 per PPG vs. 3 APG, p = 0.09). The fit test failures after the USCs were not significantly different (10 per PPG vs. 16 per APG, p = 0.73).
Fakherpour et al., 2021 [130]	20 models of FFRs (KN95, N95, N99, FFP2, and FFP3)	37 volunteers (12 males, 25 females)	Eleven out of 20 FFRs had a passing rate lower than 10%. The highest proportions of passing the fit test were 43% (Uvex-Silv Air 2200) and 27% (3M8514 N95 and Termeh PAG3711 N99/FFP3), respectively. A significant difference was observed among the studied FFRs by mean FFs (p < 0.001). cup-shaped respirators achieved a higher FF than the flat-fold ones (48 vs. 30, p < 0.001).
**Study**	**Respirator Features (Brand, model, size, style)**	**Subject Characteristics**	**Findings**
Jankusol et al., 2023 [153]	A duckbill-shaped N95 respirator	34 Physicians (20 males, 14 females)	The lowest FF for VL was higher than DL (168 vs. 88, p = 0.048). There was no significant difference between the ET intubation with VL vs. with DL (88.2% vs. 67.6%, p = 0.065).
Joshi et al., 2021 [44]	Commercial N-95 respirator, surgical mask, and cloth mask	Laboratory set up	The FFs for the face fix position were 28.86±5.37 using the PortaCount and 30.43±7.43 using the CPC. The FFs for the sealed position were 61.43±17.46 using the PortaCount and 58.95±13.89 using the CPC.
Jean-Romain et al., 2021 [56]	151 FFRs	Three volunteers (Two males, one female)	55% of the tested products failed the fit test.
Jung et al., 2021 [87]	3M 1870 N95 respirators	Ten Asian female infection control practitioners	60%, 70%, and 90% of the subjects failed the fit test after 2, 3, and 4 successive donnings per one hour donning. 50% of fit testing failures occurred after a single use of one hour and 30% after a single use of two hours.
Kamal et al., 2023 [154]	Proshield® TN01-11 duckbill N95 respirator	60 volunteer HCWs (Nine males, 51 females)	The fitting passing rate increased from 13.3% to 81.7%. The mean FF increased from 40.3 to 193.0, (p<0.001). The OR significantly increased after the application of safety goggle (OR: 42, 95% CI: 7.14–1697.9, p < 0.0001).
Kyaw et al., 2021 [82]	Three respirators, including 3 panel flat-fold 3M 1870 three-panel flat-fold respirator, cup-shaped 3M 1860 respirator, and Duckbilled ProShield respirator	70 HCWs (22 males, 48 females)	44 (63%) of the subjects experienced fogging, and 35 (70%) failed the fit test. The OR for fogging of eyeglasses to determine poor fitting was 2.10 (95% CI: 0.78–5.67, p = 0.22). Also, fogging had low sensitivity (71%) and low specificity (46%). The fogging of eyeglasses had an AUC ROC of 0.59.
Landry et al., 2022 [109]	OBE Premium surgical mask & 3M Aura 1870A N95 respirators	One male HCW	Only the fit-test _PASSED_ N95 respirator resulted in lower virus counts compared to the control. The HEPA filter, when combined with the fit test _PASSED_ N95 mask, could protect against exposure to high virus loads.
**Study**	**Respirator Features (Brand, model, size, style)**	**Subject Characteristics**	**Findings**
Lindsley et al., 2021 [121]	Nineteen masks/ respirators including two N95 respirators, two medical masks, and 15 reusable cloth masks (face masks, neck gaiters, and bandanas)	Elastomeric manikin headforms & three human subjects	The 3M 1860 N95 respirator had an FF of 163.8 and the BYD N95 respirator had an FF of 147.9 on human subjects per N95 mode and were 45.3 and 54.9 per all particle sizes, respectively. The FF of the 3M 1818 surgical mask was 78.6 on human subjects. The Manikin fit factors per coughing for the 3M 1860 N95, BYD N95 respirators, and 3M 1818 surgical mask were 198, 25, and 26.1, and per the exhalation, they were 132.5, 113.5, and 27.5, respectively.
Long et al., 2022 [118]	Masks with healthcare (N95 model 3M 1870 and 1860 N95 respirators, HY8510, H500, H100, Abdominal Pad, Surgical Mask, Sterilization Box Filter, Pediatric Drape, Bair Cover, Surgical Gown, Chux, Shoe Cover, and Mayo Stand Cover) and consumer material (Vacuum Bag, HVAC Filter, Smart Fab, Interfacing, Lawn Fabric, Shopping Bag, Paper Towel, Pillowcase, T shirt, Cotton, Chemex (coffee filter)	Laboratory setup	The N95 3M 1870 and 1860 N95 respirators had the highest FE (99.43% and 98.89%, FF: 175.44, 90.09, respectively). The FEs for the medical-grade materials ranged from 30 to 86% (FF: 1.43–7.14). H500, Halyard corresponded to the highest FEs of 87% of all medical grade-materials (FF: 7.69). The FEs for consumer grade materials ranged from 35–53% (FF: 1.54–2.13). The FE for the vacuum bag was higher than that of the coffee filter and cotton cloth (82% vs. ∼30% and 20%, FF: 5.55 vs. 1.43 and 1.25).
Milosevic et al., 2021 [107]	Eight N95 respirators (cup-shaped 3M 1860, cup-shaped 3M 1860S, flat-fold 3M 1870+, cup-shaped 3M 8110S, cup-shaped 3M 8210, flat-fold BYD DE2322, duckbill BSN TN01-11, and duckbill BSN TN01-12)	6287 HCWs (2089 males, 4198 females)	93.3% passed the fit test. 57% passed the first FFR, 21% and 14%, and 9% of the participants passed the 2 and 3, 4 or more models of FFRs, respectively. Among all, the cup-shaped 3M 1860S respirator had the highest odd ratio (OR) for passing the fit test (2.22, 95% CI, 1.94–2.54). The passing rates for the participants in the age group 18–29 were significantly higher than those with ages ranging from 30 to 59 (58.9% vs. 53.56%). The OR for passing rate for males was lower than for females (48.1% vs. 59.9%, OR: 0.85 vs. 1, p <0.001).
**Study**	**Respirator Features (Brand, model, size, style)**	**Subject Characteristics**	**Findings**
Ng et al., 2022 [80]	Four N95 respirators, including: semi-rigid cup 3M 1860 or 1860S, flat-fold cup BYD Care DE2322, duckbill BSN Medical ProShield or Fluidshield surgical masks Halyard, and 3M Aura 9320A+ three-panel flat-fold types	2161 HCWs, including medical practitioner, nursing, allied health medical imaging, other health care worker, non-clinical employee, pharmacist, dental professional (532 males, 1586 females)	The passing rates for the semirigid cup respirators (65.0%), flat-fold respirators (32.4%), for the duckbill respirators (59.2%), and three-panel flat-fold respirators (96.4%) were obtained. The three-panel flat-fold respirators had the highest comfort and usability values and the semi-rigid cup respirators had the lowest comfort and usability values.
O’Kelly et al., 2021 [132]	N95 respirator, surgical and two fabric face masks	One participant	The filtration efficiency of the N95 respirator, surgical mask, and two fabric face masks against the fine particles was 99.6%, 78.2%, and 62.6%-87.1%, respectively. Their FFs were 250, 4.59, and 2.68–7.75, respectively.
O’Kelly et al. 2021 [102]	Five N95 respirators, including 3M N95 8511, 3M 8200, Aero Pro AP0028, Makrite 9500, Xiantao Zong ZYB-11, one Zhong Jian Le KN95 respirator, one surgical mask, and five fabric masks,	Seven participants (Three HCWs, one industry workforce)	The N95 respirators provided more protection than the others. Three out of seven subjects passed the fit testing of the 3M 8511 N95, and two subjects passed the 3M 8200 N95 respirator (mean FF: 72.3). All subjects failed the Xiantao Zong respirator and Aero Pro respirator (mean FF: 13.2 and 35.5, respectively). One passed the Makrite respirator (FF: 37.7). The KN95 respirator, fabric masks, and surgical mask had a mean FF of 2.2, 2.2, and 3.2, respectively. There was a poor correlation between the fit checks and QNFF values.
**Study**	**Respirator Features (Brand, model, size, style)**	**Subject Characteristics**	**Findings**
O’Kelly et al., 2022 [155]	3M 8511 and 3M 8200 N95 respirators	Two participants (One male, one female)	The experiments indicated that as the FF increased, the gap size decreased. The minimum gap size to compromise N95 performance was about 1.5–3 mm^2^. The gap sizes of 0.4 mm and 0.8 mm had no impact on FF, while a gap size of 1.4 mm led to decrease in FF by a factor of 2.0, and a 2.9-mm gap decreased FF by approximately a factor of 4.0. A gap size of 1.4 mm or higher resulted in an FF of 23.9.
Park et al., 2021 [41]	Five 3M N95 respirators (Ever Green C250, DOBU LIFE TECH 201, DOBU LIFE TECH 500, 3M1860, and 3M 9210+) and six KF94 medical masks (3 horizontal and 3 vertical folding types; two large and one medium-sizes)	30 HCWs, including nurses and doctors (14 males, 16 females)	The N95 respirators had higher overall adequate protection rate (pass rate) by FFs than the KF masks (48.7% vs. 1.1%, 94.0 vs. 4.0, OR: 84.4, p<0.001, respectively) and by leakage rate (42.0% vs. 2.8%). Also, the passing rates and FFs for the 3M N95 respirators were higher than for the Korean (domestic) masks (25.6% vs. 83.3%, 38.5 vs. 200, OR: 25.3, p<0.001, respectively). Face length and age were significantly associated with adequate protection.
Popov et al., 2022 [137]	Five type respirators, including 3M EHR 7502, 3M particulate respirator 8511, N95 Particulate respirator KN95, Surgical mask, and Cotton mask	Three Caucasian male volunteers	The N95 respirator passed the fit test (FF>100). But the KN95 had a low FF> 4 or 7 after multiple tests. The FFs were for the 3M EHR 7502: 460–660, 3M 8511 N95: 113–211, KN95 respirator: 24–57, surgical mask: 2–4, and cotton mask: 2–7. The extensive head and body movements could affect the respirator adjustment.
Regli et al., 2022 [42]	BSN Medical Proshield N95 respirator model TN01 (TN01-11 medium or TN01-12 small)	44 HCWs	The fit test rates for modified and standard QNFT procedures were 74% and 42%, respectively. The modified fast QNFT had a low sensitivity of 50%, a TP of 26%, a TN of 47%, and a FN of 26% compared to the standard QNFT procedure.
**Study**	**Respirator Features (Brand, model, size, style)**	**Subject Characteristics**	**Findings**
Regli et al., 2021 [5]	ProshieldVR N95 respirators (small TN01-12 and medium TN01-11) & 3M 9322A^+^ P2 N95 respirator	84 staff from the Department of Anaesthesia and Pain Medicine including 53 predominantly anaesthetists and 31 predominantly anaesthetic technicians (40 males, 44 females)	The first passing rate was 47% (34 out of 72), and the overall fit pass rate was approximately 79% (63 out of 80). Different mask types and sizes resulted in higher fit test pass rates. The QNFT had a higher pass rate than the QLFT per N95 respirator not used (74 vs. 59, p<0.006). The QNFT and QLFT had a low significant agreement (k = 0.32).
Prince et al., 2021 [139]	Five commonly protective face masks including 3M N95 respirator model 8210, Dr Puri KF94 supplied with ear loops and clip, Lei Shi De KN95, Medline Industries ear loop procedure masks, Hanesbrands reusable 3-ply 100% cotton fabric masks	Ten adult male staff members (Five with full facial hair, five with no facial hair)	The N95 respirator had the highest FFE compared to all the studied masks (85.3%). The FFEs for the KF94 and KN95 decreased to 61.9% and 54.9%, respectively. The FFEs for the procedure and cloth masks ranged from 30.6% to 39.4%. Also, the exercise band resulted in improving the FFE (96.1% for the N95 and 80.2% for the KF94, 65.7% for the KN95, 36.6% for the Procedure mask, and 40.6% for the Cloth/cotton mask, respectively).
Sandaradura et al., 2020 [115]	3M Flat fold 1870 P2/N95 respirator	105 male hospital employees (38 clean-shaven, 67 unclean- shaven)	Approximately 32% passed the fit test, of which 47% were clean-shaven. Facial hair growth resulted in FF reduction. The OR for respirator fit was 0.74 (95% CI 0.21–2.52, p = 0.08) for light stubble, 0.45 (95% CI 0.12–1.57, p = 0.26) for moderate to heavy stubble, 0.04 (95% CI 0–0.28, p<0.001) for full beard, and 0.56 [95% CI 0.05–4.48, p = 0.85] for other types of facial hair, compared to no facial hair.
**Study**	**Respirator Features (Brand, model, size, style)**	**Subject Characteristics**	**Findings**
De-Yñigo-Mojado et al., 2021 [38]	Surgical masks and FFP3 respirators (Moldex-2505, 3M Aura-9332+, and 3M K-113, Surgical masks (Shell type))	63 male HCWs (32 with facial hair, 31 without facial hair)	No significant difference was found between the bearded and non-bearded HCWs by the FFs of the studied surgical masks (2.37 ± 0.73 vs. 4.68 ± 7.52 p = 0.788). However, significant differences were found between the HCWs with and without facial hair by the FFs of FFP3 (30.59 ± 29.98 vs. 65.75 ± 37.58 p<0.01).
Sasko et al., 2023 [57]	N95, P2, and reusable respirators	HCWs	One reusable respirator failed the fit test. Of the 686 N95 and P2 respirators tested, 377 (55%) passed the fit test. But 22.3% failed at least one or more fit test exercises. 109 out of the 294 usual respirators supplied before the COVID-19 pandemic passed the fit test. 268 out of the 392 additional respirators supplied passed the fit test.
Seo et al., 2021 [78]	Two types of N95 filtering face-piece respirators (DOBU MASK 201 N95, Clean Top N95 C250)	56 HCWs, including doctor, nurse paramedic, other medical technologists (14 males, 42 females)	The overall fit test pass rate was about 98.2. The medium face size (51.8%), small face size (35.7%), and outlier group (10.7%) had the highest passing rate, respectively. No significant difference was found between the participants’ face sizes, whether they passed or failed the fit test (p<0.767). The GM±GSD FFs for the medium, small, and outlier categories were 25.72±2.41, 25.51±4.58, and 22.97±8.12, respectively. Females had significantly higher passing rates than males (41.1% vs. 10.7%; p<0.028). The face size distribution was significantly different between the NIOSH bivariate panel subjects and Korean HCWs (p = 0.009). 10.7% of the subjects were outliers who did not place within the panel’s cells.
Seo et al., 2020 [32]	Four types of N95 masks	35 HCWs (14 males, 21 females)	The overall passing rate was 21%. There was no significant difference between the N95 respirators with/without a nose pad (25.6±23.1 vs. 29.1±47.6, p = 0.1551. Also, no significant difference was found among the four types of respirators (p = 0.4863). No significant difference was found between the males and females (29.2±38.6 vs. 26.1±36.8, p = 0.9961).
**Study**	**Respirator Features (Brand, model, size, style)**	**Subject Characteristics**	**Findings**
Sheikh et al., 2022 [66]	3M N95 1870+ respirator, Honeywell DC 365, 3M 1860, 3M 1860s, 3M 1804s, a 3M 6000 reusable elastomeric half-facepiece respirator	36 HCWs, including physician, nurse, respiratory therapist (Six males, 30 females)	36 out of 41 (97.3%) HCWs passed the fit test. 23 of the 36 (63.9%) passed the first try, while the remaining 13 required more than one fit test. 27 (75%) HCWs were fitted to the 3M 1870+, 4 (11.2%) were fitted to the Honeywell DC 365, 3 (8.3%) were fitted to the 3M 1804s, and one subject (2.8%) was fitted to the 3M 1860s. The overall FF for non-White 175 (32) and White males; 200 (0), for non-White 165 (30), and for White 175 (31) females were obtained. The FFs for the males were higher than for the females (majority of the participants). 27 of the 36 (75%) HCWs were out of panel.
Sickbert-Bennett et al., 2020 [156]	3M 1860 N95 respirator, surgical mask with ties, procedure mask with ear loops	Two participants (One male, one female)	The FFE for the N95 respirator in the wrong size was not significantly decreased (90–95%, FF: 10–20). The FFEs for all non-approved respirators (n = 6) were lower than 95% (FF: 20). The mean FFE of surgical masks with ties (71.5% (5.5%), FF: 3.45 (1.06)) and procedure masks with ear loops (38.1% (11.4%), FF: 1.62 (1.13)) was lower than that of the 3M 1860 N95 respirator (98.5% (0.4%), FF: 66.67 (1)).
Suen et al., 2022 [75]	Four N95 FFRs, including three traditional 3M FFR models 1860, 1860S and 1870+ and a nanofibre N95 FFR	104 nursing students (21 males, 83 females)	69 (66.3%) subjects passed the Best-fitting 3M FFR and 82 (78.8%) passed the nanofiber FFR. The best-fitting 3M FFR had higher failure rate than the nanofibre FFR (33.7% vs. 21.2%, p = 0.417) after the procedures. The average FFs of both traditional and nanofibre FFRs decreased after performing nursing procedures (3M FFR: 185.08 vs. 135.52; nanofibre FFR: 188.44 vs. 149.13, p>0.05). The nanofibre FFR had significantly higher usability than the 3M FFRs (i.e., facial heat, breathability, facial pressure, speech intelligibility, itchiness, difficulty of maintaining the mask in place, comfort on ear lobe and overall comfort level), p<0.001.
Goh et al., 2022 [96]	Two N95 respirators with micro fan (MF) and AIR+ Smart Mask	106 children (59 boys, 47 girls)	All subjects passed the fit tests. The respirators with or without MF were safe for children. The novel respirator could enhance the comfort and experience of wearing the mask.
**Study**	**Respirator Features (Brand, model, size, style)**	**Subject Characteristics**	**Findings**
Salter et al., 2021 [43]	Cloth masks (17 cotton batting masks), Moldex N95 respirator model 2212 (reference)	Laboratory set up	There were not significant differences between FFs and filtration efficiencies for the cloth masks with and without the gaskets in the first set (90.4% vs. 90.0%, FF: 10.0 vs. 10.3). In the second set, the average filtering effectiveness for the masks with gaskets was 77.3% (FF: 4.4). The average filtering effectiveness for the mask with and without a nylon layer over the mask included 76.5% vs. 83.7%, FF: 4.25 vs. 6.13, per third set.
Vahabzadeh‐Hagh et al., 2022 [98]	3M 1860 N95 respirator	One patient	The FE for the Polypropylene sterilization wrap (97.31 ± 0.32, FF: 37.17) was similar to the 3M N95 1860 respirator (96.52%, FF: 28.73).
Vo et al., 2020 [157]	North N95 FFR model 7130N95	Eight subjects (Four males, four females)	All subjects passed the fit test. The SWPFs obtained from CPCs had a good agreement with SMPSs. The CPCs, PAMSs, and reference SMPSs had GM SWPF trends under similar simulated workplace activities. GM SWPF decreased with increasing simulated activities. There were no significant differences between the GM overall SWPF of SMPS and CPC (at low concentration: 28.56 ± 1.07 vs. 23.16 ± 1.16, p = 0.17, and medium concentration: 36.93 ± 1.35 vs. 29.48 ± 1.41, p = 0.23).
Vuma et al., 2021 [105]	N95 FFR (3M 1860 FFR)	25 employees of the National Institute for Occupational Health (NIOH) (Nine males, 16 females)	The median FFs were 195 (139–200) for the fit test 1, 161 (110–200) for the fit test 2, 167 (132–200) for the fit test 3, 124 (79–198) for the fit test 4, 168 (75–200) for the fit test 5, and 150 (72–192) for the fit test 6. Two subjects (8%) had FF<100 fit test 2, 6 (24%) at fit test 3, 8 (32%) at tests 4, 5, and 6. Thirteen subjects (52%) had FF>100. There was a significant difference between the FFs of the first and sixth tests (195 vs. 150; p = 0.0271) but not between the second and sixth FFs (161 vs. 150; p = 0.3584). The FFs for the males and females were similar. Also, the overall FFs for infrequent users were higher than for frequent users.
**Study**	**Respirator Features (Brand, model, size, style)**	**Subject Characteristics**	**Findings**
Williams et al., 2021 [64]	Two duckbill models of N95 FFRs, including Halyard FluidshieldVR N95 and the BSN Medical ProShieldVR N95 respirators	96 anaesthetic staff, including Anaesthetic consultants and trainees (55 males and 41 females)	The passing rates for the Halyard Fluidshield (77%) and ProShieldVR (65%) were not statistically significant (p = 0.916). The median IQR for the Halyard Fluidshield was 144 (102–196) and the ProShieldVR was 119 (29–200), p = 0.09. Also, there were low agreements between the USCs and fit tests (0.16 for the Halyard Fluidshield, 0.08 for the ProShieldVR). The diagnostic tests showed PPV: 79.8%, NPV: 41.7% Sensitivity: 90.5% Specificity: 22.7% Overall accuracy: 75%, for the Halyard Fluidshield respirator and PPV: 66.7%, and NPV: 46.4%. Sensitivity 80.6%, Specificity: 26.5% Overall accuracy: 61.5%, for the ProShield respirator.
Williams et al., 2021 [40]	Two types of three-panel flat-fold respirators including Trident^TM^ P2 FFR and 3M 9320A+ Aura three-panel flat-fold N95 FFR	500 HCWs including nursing, medical practitioner, aged care/ disability worker, allied health, medical imaging, other healthcare worker, pharmacist, non-clinical role (122 males, 378 females)	The Trident^TM^ respirator had a significantly higher overall fit test passing rate (99.2% vs. 92.6%, p<0.001) and first-attempt passing rate (76.4% vs. 92.6%, p<0.001) than that of the 3M^TM^ Aura respirator. Also, the median (IQR) FFs for the Trident^TM^ were significantly higher than for the 3M^TM^ Aura (201 (201–201) vs. 201 (166–201), p<0.001).
**Study**	**Respirator Features (Brand, model, size, style)**	**Subject Characteristics**	**Findings**
Williams et al., 2022 [111]	Two brands of 3M Aura 3M 9320A+ FFP2 and 3M 1870+ N95 surgical masks	1000 participants from Royal Melbourne Hospital (332 males, 668 females)	The 3M 9320A+ had a significantly higher passing rate (94.6% vs. 91.7%, p<0.001) and FF (183±37.9 vs. 175.0±45.4, p<0.001) than the 3M 1870+ FFR. The overall passing rate was 89.2%. A fair agreement was observed between the passing rates of two FFRs (k = 0.38). Males had higher passing rates and FFs than females: 96.7% vs. 93.6%, p = 0.04; 187.2±32.2 vs. 181.0±40.3, p = 0.006, per 3M Aura 9320A+; 97.6% vs. 88.8%, p<0.001, 185.2±31.5; 170.0 vs. 50.2, per 3M Aura 1870+, p<0.001.
Williams et al., 2022 [158]	Halyard N95 FFR flat-fold duckbill respirator	350 HCWs (81 males, 230 females)	72.2% of participants passed the fit testing using the handhold, and 52% passed the lanyard technique (p<0.001). The overall FF for the Hand-hold technique, 167 (89–201) was higher than the Lanyard technique, 112 (52–196), p<0.001. A fair agreement was observed between the two techniques (k = 0.39). The method of sampling tube stabilization during QNFT could lead to false negative fit testing results due to inadequate tube stabilization.
Lim et al., 2020 [97]	PNTD KF80 disposable particulate respirator	20 older female participants	The mean leakage rates in the first, second, and third tests were 73.6%, 71.5%, and 72.8%, respectively. The overall passing rate was 14.3%. Only 3 (14.3%), 4 (19%), and 6 (29%) of the participants passed the fit test (leakage test), respectively.
Mottay et al., 2020 [88]	Twelve KN95 respirator brands (total of 36 masks)	Seven HCWs and laboratory workers (One male, six females)	35 out of 36 masks failed the USCs. The KN95 respirators had lower passing proportions of the USC than that of the N95 respirators (1/36 (3%) vs. 12/12 (100%); p<0.0001). 15 out of 36 (42%) and 12 out of 12 (100%) passed the USCs of the KN95 respirators and N95 respirators, respectively, using modification of ear-loop tension using head straps or staples or the face seal improvement using Micropore 3M tape. None of the respirators passed the QLFT, and then, they did not proceed to the QNFT.
**Study**	**Respirator Features (Brand, model, size, style)**	**Subject Characteristics**	**Findings**
Zhang et al., 2020 [128]	Four models of FFRs: including three N95 and one FFP3 respirator (two cup-shaped, two flat-fold styles)	85 volunteers (31 males, 54 females)	The passing rates and GSD FF for four models were 52.9% (112.5±58.4), 61.2% (121.5±57.0), 40.0% (92.2±62.6), and 63.5% (121.0±58.7), respectively. A significant difference in passing rates among the four models was found (p<0.05). Only 17 (20%) subjects passed the fit test of four models. There was a significant difference in passing rates for model 3 between males (54.8%) and females (31.5%). The passing rates and GM FFs for the flat-fold respirators (51.8%, 92.2; model 3 and 121; model 4) were lower than those for cup-shaped ones (57.1%, 121.5; model 1 and 121.5; model 2). There were significant differences between passed and failed subjects in face length, and nose height, nose length (p<0.05).
Boogaard et al., 2020 [159]	Three different types of locally-produced facemasks, including Reinier 0.1, DSM 1.0, and Reinier 1.0	Three subjects	The min and max IL were obtained for the Reinier 0.1: 4.2, 4.8; for the DSM 1.0: 6.7%, 14.6%; and for the Reinier 1.0: 0.5%, 0.8%. The Reinier-0.1 and -1.0 models had acceptable Max IL <8%. Whereas, the DSM 1.0 did not meet the value (14.6%).
Carvalho et al., 2021 [117]	EN149:2001 approved-N99 and FFP3 respirators	1182 HCWs (365 males, 817 females)	Males were better fitted to the respirators than females (mean first-attempt passing rate: 51.46% vs. 42.66%, adjusted OR: 2.07, 95%CI (1.66–2.60) p<0.001). Among the various ethnic groups, White staff were better fitted than other participants (p<0.001). The Whites had a significantly higher pass rate than non-Whites (48.7%).
Caggiari et al., 2023 [134]	FFP3 respirator	9592 HCWs (2009 males, 7583 females)	17% of the subjects failed all attempts, 60% passed one attempt, and <15% passed between the 2 and 5 attempts. White male subjects had the highest pass rates (74%). The odds for males’ fit success were higher than those of females (OR: 1.51; 95%CI: 1.27–1.81). The HCW with a low BMI <18.5 kg/m^2^ had significantly lower odds of passing fit testing compared with other groups (OR: 0.516, 95%CI: 0.362–0.735, p<0.0001). There was a slight difference between the results of the fit test and the measurements of the face.
**Study**	**Respirator Features (Brand, model, size, style)**	**Subject Characteristics**	**Findings**
De‐Yñigo‐Mojado et al., 2021 [37]	FFP3 and surgical masks	74 nurses (37 males, 37 females)	There were no significant differences among males (2.86±2.73) and females (3.55±6.34) by mean FFs for the surgical masks (p = 0.18). There were significant differences among males (30.82±28.42) and females (49.65±43.04) by mean FFs for the FFP3 (p = 0.037). According to the OSHA criteria, only 2.70% and 13.51% of male and female nurses passed (p = 0.199). Whereas, 21.62% and 48.64% of male and female nurses passed using the FFP3 respirator, according to the AIHA criteria (p = 0.027).
De-Yñigo-Mojado et al., 2020 [65]	FFP3 respirators, surgical masks, and other types of masks	78 physicians (37 males, 41 females)	The FFs for the FFP3 respirator were higher than for the surgical mask and other types of masks (40.7±37.8, 95% CI (32.3–49.1) vs. 3.2±5.0, 95% CI (2.1–4.3), p<0.001).
Green et al., 2021 [114]	86 FFP3 respirator types (3M, RFP3FV, Easimask FSM, and Alpha Solway)	22783 hospital staff (4863 males, 17920 females)	Approximately 20% of the HCWs failed the fit test during the COVID-19 pandemic. The mean passing rate was 80.74%. The males had higher failure rates for all respirators than the females (20.1% vs. 19.9%). Failure rates of the HCWs from BAME backgrounds were high (25.69%). Across all seven hospitals, 18.98% of men tested failed the fit-test for all masks tested; 19.89% of females tested failed the fit-test for all masks used (X^2^ = 0.079, p = 0.398).
Sun et al., 2020 [160]	Two brands of FFRs, including FFP3 brand A and FFP1 brand B	Eight test subjects	A linear relationship was determined between the PortaCount (without N95-Companion) and flame photometer under all conditions (R^2^ = 0.9704). The distribution of particle size was similar in almost all cases. The SWPFs from CPC were correlated with SMPS (R^2^ = 0.70).
Vanhooydonck et al., 2021 [62]	Novel FFP3 FFR	ND	The fit testing of VMX Silicon 10A (1.5 mm) and Rolyan Polycushion (3.2 mm) in three replications showed that both obtained FF ranges 210–550 and 320–420, respectively.
**Study**	**Respirator Features (Brand, model, size, style)**	**Subject Characteristics**	**Findings**
Winski et al., 2019 [100]	3M 8835 + FFP3 respirator	262 employees (237 males, 25 females)	Fourteen (5.3%) subjects had FF < 100. The median FF was 416 (IQR: 294–604). No correlation was found between FF and face length (r = −0.08; p = 0.214) and a negative correlation was observed between FF and face width (r = −0.17; p = 0.006) and jaw width (r = −0.28; p<0.001). No differences were determined between the NIOSH panel face sizes (including small: 17, medium: 145, and large: 97) and FF (p = 0.194). All small-face subjects passed the fit test.
Chapman et al., 2022 [84]	A locally manufactured N95 respirator	33 HCWs	The fit test passing rate was 63.6% (21 out of 33). Also, 19 participants passed the large size and two passed the small size. No participants passed the X-large size. 84.8% of the participants failed at least one of the fit tests before passing. The fit coaching for the failure groups was provided by the manufacturer’s instructions to assure the users’ well-fitting respirators. The mean FF was 162.4 ± 31.8 for the pass groups and 65.4 ± 60.8 for the failure groups.
Chen et al., 2022 [161]	3M N95 respirator model 8210	21 healthy participants (Seven males, 14 females)	The progressive trend in the FFEs from reference (86.1%, FF: 7.19) to manufacturer paper (93.3%, FF: 14.92), video (97.5%, FF: 40), and post-staff intervention (98.3%, FF: 58.82) was observed. The video instruction (p<0.037) and staff intervention (p<0.033) sessions significantly improved the FFEs for the baseline.
Clark et al., 2021 [92]	3M 8210 N95 & 3M 1860S N95 surgical masks	65 dental and dental hygiene students (45 males, 20 females)	All participants knew how to wear the N95 respirator. 41 (63%) participants noted that their safety perceptions altered after fit testing.
Inolopú et al., 2023 [145]	12 models FFRs	263 HCWs	Among all, 87 (33.1%) HCWs had FF>100, 27 (10.3%) ranged 50–99, and 149 (56.7%) had FF<50. The 3M N95 1860 had highest FF (mean FF: 126.0, 95% CI (109.4–146.6)). The 3M respirator models increased the FF after post-instructional FF (p≤0.01).
**Study**	**Respirator Features (Brand, model, size, style)**	**Subject Characteristics**	**Findings**
Low et al., 2021 [140]	BSN Medical ProShieldVR N95 respirator model TN01 (TN01-11 medium or TN01-12 small)	65 participants including the Anaesthetists, anaesthesia registrars, and nurses (33 males, 32 females)	The fit test passing rate was lower than the USCs passing rate (22 (34%) vs. 65 (100%), p<0.0001). The overall passing rate following the education of the remaining 16 participants was 38 (58%).
Ngobeni et al., 2020 [81]	HALYARD Health N95-FFRs (46827, small size and 46727, regular size)	37 HCWs (Two males, 35 females)	Approximately 37 out of 99 (37.4%) of the HCWs underwent both QNFT and QLFT. A total of 17 (45.9%) passed the QNFT procedures (S_e_ = 0.45, Sp = 0.50). About eight out of 37 (34.8%) passed the N95-FFR model 46727 and three (60%) passed the N95-FFR model 46827. 46% of the HCWs (11/24) who had worn a respirator before and 47% of the HCWs (9/19) who had received prior training passed the fit test.
Robertsen et al., 2020 [55]	ND	240 participants (146 males, 18 females, others not determined)	An improvement in knowledge of Group 1 (5.0 vs. 6.0) and Group 2 (5.50 vs. 6.25), attitudes (4.29 vs. 4.43), and organizational support of Group 1 (5.50 vs. 5.67) occurred, while an improvement in subjective norms related to RPE use occurred in intervention Group 2 (3.50 vs. 4.33). No significant difference was observed in intention to use or rate of respirator use. Participation in both groups could improve the intention to use respirators.
Seo et al., 2021 [33]	Two types of domestic N95 masks (Folder and Cup styles)	59 HCWs (16 males, 43 females)	The GM±GSD FF value for the cup-style was significantly higher than the folder type (62.18±3.22 vs. 22.65±4.18, p = 0.001). There was a significant difference between the FFs before and after training (p = 0.0015).
**Study**	**Respirator Features (Brand, model, size, style)**	**Subject Characteristics**	**Findings**
Williams et al., 2021 [59]	Four N95 respirators, including Semi-rigid cup 3M-1860 or 1860S, Flat-fold BYD Care, Duckbill BSN medical ProShield, and Halyard Fluidshield	125 HCWs (29 males, 94 females, 2 other)	The knowledge, donning and doffing skills, and USCs were significantly improved (p< 0.01).
Yeon et al., 2020 [72]	TB N95 mask	56 HCWs, including nurses (One male, 55 females)	There were no significant differences between knowledge and attitude toward PPE use. 19 (68%) of the experimental group and 14 (50%) of the control group passed the fit test (p = 0.354).
Xiao et al., 2023 [83]	N95 mask	442 Hospital staff, Property logistics staff (272 males, 170 females)	Significant differences were found between various training programs and the passing fit test rate (p<0.05). Passing rates increased after three tests, as follows: 239 (54.07%), 355 (80.32%) and 405 (91.63%), respectively.

Note:

FFR: filtering facepiece respirator

QNFT: Quantitative Fit Test

QLFT: Qualitative Fit Test

CNC: Condensation Nuclei Counter

CPC: Condensation Particle Counter

FF: Fit Factor

TILPF: Total Inward Leakage Protection Performance

aFF: adopted fit factor

HCWs: Healthcare workers

Se: Sensitivity

Sp: Specificity

PPV: Positive predictive value

NPV: Negative predictive value

FFE: fitted filtration efficiency

GRPF: General respirator protection factor

PPG: partially passed group

APG: group that passed all exercises

ET: Endotracheal tube intubation

VL: video laryngoscopy

DL: direct laryngoscopy

OR: odd ratio

AUC: area under the curve

ROC: receiver operating characteristic curve

QNFF values: quantitative fit factor

TP: true positive

TN: true negative

FN: false negative

FE: filtration efficiency

SWPF: simulated workplace protection factor

IL: inward leakage

CI: Confidence Interval

BAME: Black, Asian, and Minority Ethnic

NIOSH: The National Institute for Occupational Safety and Health

NIOH: National Institute for Occupational Health

EHRs: Elastomeric half-facepiece respirators /reusable facepiece respirators

PF: protection factor

SSM: silicone-molded face mask

GM, GSD: geometric mean, geometric standard deviation

RFC: respirator fit capability

PPR: panel passing rate

PAPR: powered air purifying respirator

TIL: Total Inward Leakage

MAVerIC: Modified Airway from VEntilatoR Circuit

LPFs: laboratory protection factors

OV cartridges: organic vapor cartridges

AFM: Anaesthesia Face Mask

MSM: Modified Snorkeling Mask

IQR: Interquartile Range

APR: air-purifying respirator

SCBA: self-contained breathing apparatus

ND: Not determined

**Table 2:** Quantitative fit testing of reusable masks or respirators and affective factors.

Study	Respirator Features (Brand, model, size, style)	Subject Characteristics	Findings
Anwari et al., 2021 [136]	A novel reusable half-face respirator	Eight different volunteers, including members of the design and testing team (Six males, two females)	Seven out of eight (87.5%) tests passed. Although the Manitoba SSR mask with Intersurgical Hydro-Mini filter obtained the FF of the 108, failed the fit test exercises, including turning side-to-side; 93, talking; 83, and bending; 92 <100.
Chichester et al., 2020 [61]	Additively manufactured respirators	ND	Nine separate fit test evaluations were conducted. The AMR equipped with large foam and N95 and P100 filters could provide satisfactory protection (FF≥200) compared to the N95 mask (FF: 189).
Fadairo et al., 2020 [36]	Eight brands of half-facepiece and full-facepiece respirators (3M, MSA, North and Moldex) equipped with 3M, North, MSA, Moldex P-100 filters	Mannequin and eight subjects (Six African American males, one African American female, and one Asian male)	There was a significant difference in the results of CNC using the mannequin under ambient and controlled environmental conditions (26319.1 vs. 18382.6, p = 0.0005) in contrast to the CNP results (1679.50 vs. 1879.75, p = 0.7247). While no significant difference was observed in the CNP or CNC for the subjects (p> 0.05). Also, significant differences were observed in ambient and environmental conditions using the mannequin and subjects.
Hondjeu et al., 2021 [127]	Duo silicone respirator and 3M N95 respirators (1870+, 1860, 1860S 8210, and 9105S)	41 HCWs	The passing rates for the 3M N95 disposable and Duo reusable respirators were 58.5% and 100%, respectively. The 3M 1870+ and 8210 respirators had the highest pass rates (78% and 83%, respectively). The harmonic means of the FF for the Duo respirator was higher than for the N95 respirators (2959 vs. 77.4, p< 0.0001). The N95 had a lower passing rate during dynamic maneuvers than stationary maneuvers (61% vs. 73%, p< 0.0001). Also, seven subjects (17.1%) were outside of the NIOSH panel.
**Study**	**Respirator Features (Brand, model, size, style)**	**Subject Characteristics**	**Findings**
Ballard et al., 2021 [142]	3D-printed prototypes from the rigid (n = 5 designs) and flexible polymers (n = 5 designs), and disposable N95 respirator	Four HCWs	The 3D-printed prototypes with rigid materials did not pass the QNFT procedure. Also, three out of the five prototypes with flexible materials failed the fit test. Only two final 3D-printed prototypes with flexible materials had an overall mean FF of 138 (108–168) compared to the control N95 respirator (FF> 200, p<0.001).
Ballard et al., 2021 [53]	N95 respirators (a cloth-based respirator (Sewn Sterilization Wrap), three 3D-printed respirators (P100 Adaptor, Self-Moldable 3D Printed and Multi-Part 3D Printed) and one repurposed from medical supplies (Elastomeric), and 3M 1860 N95 FFR	Seven adult volunteers, including, intended users (HCWs)	Only the EHR equipped with a HEPA filter passed the fit test on both small and large face- standardized users (FF: 110 and 108, respectively).
Duda et al., 2020 [46]	Six 3D-printed face mask designs	Four participants	The PF and TIL values were measured: HSU FM V3: 2.19, 45.69%; HSU FM V4: 2.43, 41.24; Montana mask: 1.72, 58.25%; Maker mask: 1.88, 53.35%; PLA COVID-19 mask: 2.81, 35.71%; TPU COVID-19 mask: 2.33, 43.01%; and Fabric mask: 2.23, 44.78%.
Imbrie-Moore et al., 2020 [47]	3D-printed mask adaptor	Six subjects	All subjects passed the fit testing of the proposed mask. The overall FF was 148>100.
Levine et al., 2022 [48]	3D Printed Masks (Covid Mask Respirator, Low Poly, and Covid-19 Respirator), N95 and a KN95 respirators	Five volunteers (Three males, two females)	The Mask 1, Mask 3, and KN95 respirators had an FF of 52.2, 1.8, and 5.4, respectively. The Mask 2 (Low Poly Low Poly Covid-19 Face Mask Respirator) had a higher FF≥100. All subjects passed the quantitative fit testing of Mask 2 and the N95 respirator. There was no significant difference between the mean FFs for the Mask 2 and the N95 respirators (141.25 vs. 175.60, p< 0.226).
**Study**	**Respirator Features (Brand, model, size, style)**	**Subject Characteristics**	**Findings**
Liu et al., 2020 [52]	3M^TM^ re-usable elastomeric respirators equipped with a 3D-printed adaptor	Eight volunteers (Five males, three females)	All volunteers passed the USCs. All eight volunteers passed the fit test. Also, all females were fitted with the 3M 7501 (small) respirator.
Manomaipiboon et al., 2020 [116]	Silicone VJR-NMU N99 half-piece respirator	41 HCWs (21 males, 20 females)	32 (78%) subjects passed the first fit test. After tightening the O-ring trap, seven subjects passed the fit test (77.8%). Five subjects passed the third fit test (80%). The overall fit test passing rate was 40/41 (97.6%). One subject failed, even after adjusting the strap for the third time.
Martelly et al., 2021 [129]	A Reusable, Hot Water Moldable, Additively Manufactured Mask	13 subjects (Six males, seven females)	There was an improvement in fit between the unmolded and molded masks (7 ± 17 vs. 143 ± 62). The molded mask had a passing rate of 77% (10 out of 13).
Meadwell et al., 2019 [143]	Nine designs of elastomer	One human subject	The pressure testing performed well; however, it could not be substituted by robust fit testing. The highest FF obtained by continuous ribs-soft elastomer (18.51; 1129/61).
McLeod et al., 2021 [124]	3M EHR model 6000	Mannequin	The FFs were highest for the EHRs with two layers of 7093 3M NIOSH P100 Particulate Filter was 2281, and two layers of P100 3M 2097 NIOSH were 1678. The FF for the combinations of Super-calendered Final Product (1 ply)-Side overhang and P100 3M 2097 NIOSH was 341. The FF for the combinations of uncalendered Final Product (2 ply)-Side overhang and P100 3M 2097 NIOSH was 215.
Ng et al., 2020 [112]	The reusable silicone-molded face mask (SSM)	40 HCWs (20 males, 20 females)	The mean harmonic FFs for the N95 respirator and SSM were 137.9 and 6316.7, respectively. The overall passing rates for the mentioned masks were 65% and 100%, respectively.
**Study**	**Respirator Features (Brand, model, size, style)**	**Subject Characteristics**	**Findings**
Roche et al., 2022 [51]	Personalized 3D-printed respirator	50 HCWs (21 males, 29 females)	In the control group, 38 subjects passed and 12 failed the FFP3. In the test group, 44 passed and six failed the 3D-printed respirator. 11 subjects who failed the FFP3 passed the 3D-printed respirator. Conversely, five who passed the FFP3 failed the 3D-printed respirator. No significant difference was found in the fitting rate of both respirators (170 vs. 180, p = 0.21).
Chughtai et al., 2020 [162]	CleanSpace™ lightweight tight-fitting half-facepiece PAPR	20 HCWs including nursing and medical staff (13 males, seven females)	All participants passed the fit test with a GM FF (GSD) of 6768 (3755).
Germonpre et al., 2020 [58]	Snorkel Masks	Staff of Belgium Hospitals (HCWs)	The modified snorkel masks had high FFs. Subea A: 58, Subea B, C: 200+, Subea D: 200++, Subea E: 52, Seac: 200+, Aqualung:117, Cressi: 157, Ocean Reef A:57, Ocean Reef B, C, D, and E: 200+, and 3M Aura 9322+ FFP2: 62.
Greig et al., 2020 [141]	Modified full-face snorkel mask	One male user	The novel mask failed the fit test despite passing the USCs. Then, it was considered that the QNFT procedures was required for the full-face mask.
Greig et al., 2022 [85]	Full-face snorkel mask	16 clinical staff (Seven males, nine females)	One fit test considered a pass when a P3 was mounted with an uncoated adaptor to a snorkel mask (FF: 564). No subjects passed using the coated adaptor. All subjects who used the HME filter failed the fit test (median (IQR) FF: 8 (3–23)). The coated P3 adaptors had a higher median (IQR) FF than the uncoated P3 ones (899 (350–1396) vs. 349 (169–462)).
Grinshpun et al., 2020 [163]	Three makes and models of respirators, N95 FFR, P100 FFR, and half-mask elastomeric facepiece (11 respirators)	25 adult subjects (9 males, 16 females)	The AccuFIT 9000 could identify poor-fitting respirators with a sensitivity of 0.95, a specificity of 0.97, and a Kappa of 0.92.
**Study**	**Respirator Features (Brand, model, size, style)**	**Subject Characteristics**	**Findings**
Harmata et al., 2022 [164]	Three Full-face piece gas respirators, including MP-5, MP-6, and Promask	Ten participants	The FFs were for the MP-6 mask, 1460, for the MP-5 mask, 950, and for the Promask mask, 850, were obtained. The FFs for the MP-6 masks three days, the MP-5 mask, and the Promask after two days reached <10000.
Kechli et al., 2020 [60]	Full-face snorkel mask	ND	The modified full-face snorkel mask had an overall FF of 142. The only talking exercise had an FF of 94< 100.
Kroo et al., 2021 [165]	Modified Full-Face Snorkel Masks (Pneumask)	Three volunteers	All three subjects passed the QNFT procedure.
Nicholson et al., 2021 [144]	Ocean Reef Aria full face snorkel masks (medium/large, small/medium, large/extra large), and S/M full-faced snorkel masks	One user	The FFs of the 3M 6800 full-face respirator, Snorkel mask with a duct tape, Snorkel mask with no modifications, and snorkel mask with a mouth cover remove were 333867, 32281, 15448, and 1105, respectively.
Persing et al., 2021 [166]	3M HER with P100 (OV) cartridges model 65021HA1	A single member of the research team	The LPFs for the DC CPC and PortaCount were similar, while the DC OPC was different from PortaCount. The LPFs of the PortaCount was 89, DC CPC was 77, and DC OPC was 156, per the target LPF of 100 against the Sodium chloride aerosol and 370, 330, and 961, respectively, per the target LPF of 300 against the Sodium chloride aerosol.
Pettinger et al., 2021 [63]	Three respirators, including FFP2 respirator, Anaesthesia Face Mask (AFM), and full-face Modified Snorkeling Mask (MSM)	Ten HCWs, including anaesthesiology residents (Five males, five females)	The seal check failure rates for the FFP2 (control) were 37 (41%), 10 (11%) for the AFM, and 6 (7%) for the MSM. There was no significant difference among the FFs of the studied respirators. The fit test passed rates for the FFP2 (control) were 5 (50%), 8 (80%) for the AFM, and 7 (70%) for the MSM, p = 0.69.
**Study**	**Respirator Features (Brand, model, size, style)**	**Subject Characteristics**	**Findings**
Bergman et al., 2019 [167]	Six respirators, including three families of full-facepiece respirators, including a one-size-only family, a two-size family, and a three-size family equipped with P-100 filters	25 subjects	The PPR was more than 75%. One of two donning achieved the FF of 500. The PPR for the three-size, two-size, and one-size families were 100, 79, and 88%, respectively. The PPR decreased with increasing FFs of 500, 1000, and 2000.
Chehade et al., 2021 [168]	Two masks, including assembled mask Hans Rudolf full-face mask & Respironics Performax full-face mask	20 volunteers from Oklahoma City Veteran Affairs Health Care System (10 males, 10 females)	All participants passed the test with the GM±GSD of 2317±3.8.
Han et al., 2022 [99]	Three types of respirators, including N95, half-facepiece mask, and full-facepiece mask	50 volunteer college students (25 males, 25 females)	There was a high correlation between two fit testers (p< 0.00001). The FF of 100 per N95 respirator determined by PortaCount equalized to the FF of 75 by SIBATA MT. There was very high consistency between two devices for half- and full-facepiece respirators, which both satisfied the values specified by the ANSI standard. But the N95 respirator did not meet the ANSI requirement.
**Study**	**Respirator Features (Brand, model, size, style)**	**Subject Characteristics**	**Findings**
Rengasamy et al., 2021 [169]	NIOSH-approved elastomeric half-facepiece, full-facepiece, and PAPRs with respirators tight-fitting and loose-fitting facepiece	16 subjects	The FFs were obtained for the MSA EHR: 1507, North EHR: 1667, MSA Full-facepiece: 4670, North Full-facepiece: 7753, PAPR-tight fitting; MSA: 7731, Bullard: 3799. Also, the TILs for the MSA EHR for corn oil aerosol were significantly larger than for NaCl aerosol (0.197 vs. 0.056) and for the North EHR (0.086 vs. 0.038). However, the TILs for the NaCl aerosol were significantly larger than for corn oil aerosol per the PAPRs but not per the full-facepiece respirators, including the MSA PAPR-tight fitting (0.010 vs. 0.003), Bullard PAPR-tight fitting (0.011 vs. 0.002), 3M PAPR-loose-fitting (0.013 vs. 0.003), Bullard PAPR-fitting (0.015 vs. 0.002), MSA Full-facepiece (0.046 vs. 0.049), and 3M Full-facepiece (0.015 vs. 0.016).
Sietsema et al., 2022 [106]	NIOSH-approved Envo quarter facepiece elastomeric respirator	25 HCWs of Rush University hospital (14 males, 11 females)	The median (5th and 95th percentile) FF was 188 (48, 201), SWPF-truncated SWPF was 181 (94, 199), and non-truncated SWPF was 570 (153, 1508).
Weng et al., 2022 [170]	Novel full-face mask	18 participants, (Eight males, 10 females)	The mask could provide acceptable protection.
**Study**	**Respirator Features (Brand, model, size, style)**	**Subject Characteristics**	**Findings**
Clinkard et al., 2021 [69]	N95, snorkel masks with high-efficiency filters and snorkel masks with powered-air purifying respirators	51 HCWs (24 males, 27 females)	59% and 20% of participants failed at one or more fit test exercises using the N95s and snorkel masks with high-efficiency filters, respectively. 24% and 12% of the subjects failed the overall FFs of N95 and snorkel masks with high-efficiency filters. The mean FF for snorkel masks with a PAPR (12177) and snorkel masks with a high-efficiency filter (2939) was significantly higher than that of the N95 mask (144), p< 0.05. The passing proportions of the N95 respirator (65%) and snorkel mask with a high-efficiency filter (92%) were lower than those of the snorkel mask with PAPR (100%, p< 0.01).
Convissar et al., 2020 [35]	Modified Airway from VEntilatoR Circuit (MAVerIC)	One anesthesia provider	The cost-benefit quantitative fit testing procedure consisted of Bag valve mask (an Ambu bag) with a pressure manometer was carried out using the MAVerIC.
Toigo et al., 2021 [67]	Aria Ocean Reef® full-face snorkeling mask	71 HCWs, including nurses, respiratory therapists, physicians, residents, patient attendants, technicians, and care advisors	Four out of 71 subjects underwent the QNFT, and all of them passed. 55 out of 67 conducted fit tests and passed the QLFT. 83.1% of the subjects who could not pass the fit testing of medical respirators passed the fit testing of the snorkel mask.
**Study**	**Respirator Features (Brand, model, size, style)**	**Subject Characteristics**	**Findings**
Cass et al., 2022 [93]	Two N95 respirator brands and CleanSpace HALO® powered air-purifying respirator	189 ICU staff members, including doctors, nurses, allied health professionals, and support staff member (61 males, 128 females)	Fit testing failure rates were 18/60 (30.0%) for the 3M and 33/107 (30.8%) for the Halyard. The passing fit test rate increased from 88/189 (46.6%, 95% CI, 39.3–53.9%) on unassisted fitting to 105/189 (55.6%, 95% CI 48.2–62.8%) after the provision of assistance on the first respirator type worn and 131/189 (69.3%, 95% CI ¼ 62.2e75.8%) per the second respirator type. Fifty-eight of 189 (30.7%, 95% CI, 24.2–37.8%) failed on both N95 respirator types, and 47 (100%) subjects proceeded to and passed the fit testing on CleanSpace HALO® PAPR.
Baba et al., 2022 [104]	Replaceable particulate respirators (RPRs) Chiyoda model 1180–05 and PAPR Chiyoda model BL–321S.	Ten participants from University of Occupational and Environmental Health (Eight males, two females)	The passing rate and mean FFs of both RPR (i.e., RPR-H: at resting state 3 and at exercise state: 2 out of 10 subjects, 68.2 vs. 118.7) and PAPR (i.e., PAPR-R: at resting state 10 and at exercise state: 9, 786.5 vs. 444.5) obtained from the exercising tasks were higher than the resting state (p<0.001). But the PAPR provided satisfactory protection (FF> 100).
Grinshpun et al., 2020 [122]	3M Versaflow, TR-300+ PAPR	Ten human subjects and manikin	The MPF was measured ranged from 5000–10000. The SWPF ranged from 3000–10000. A near-perfect correlation was observed between two methods (0.997). There was a high correlation between RePM and CPC in measuring different particle size ranges. High sensitivity (96.3%) and specificity (100%) achieved on human subjects at a response time of 60 sec.
Kessel et al., 2022 [110]	PAPR	One HCW (A rural healthcare provider)	The helmet equipped with two layers of H600 filter media had the highest FF of 2229 against NaCl and 28942 against SiO2.
**Study**	**Respirator Features (Brand, model, size, style)**	**Subject Characteristics**	**Findings**
McGrath et al., 2022 [86]	Bubble-PAPR	15 clinical and non-clinical staff (Five males, 10 females)	Ten subjects passed the fit test. The mean FF was 16931> 500.
Nagel et al., 2021 [50]	3D printable PAPR	Two subjects	The novel PAPR obtained the FF of 1362≥500 using the PortaCount.
Goto et al., 2021 [79]	Tight-fitting PAPR (BL-321H half-mask respirator and a BLA-62; KOKEN LTD filter)	Fifty-four HCWs, including doctor, nurse, and others HCWs (33 males, 21 females)	42 (78%) of the subjects failed at least one of the three sessions of chest compression (SWPF <500). 39 (72%), 30 (56%), and 25 (46%) failed in the first, second, and third sessions, respectively. The median (IQR) for overall SWPF was 4304 (685–16191). Therefore, tight-fitting PAPR could not provide adequate protection.
Ng et al., 2023 [135]	HALO PAPR	Eight HCWs (Four males, four females)	The mean FF was higher than 1000. There were no significant differences before, during, or after the chest compression. The FFs were when power off: 1869 (617–4333), 1748 (378–6881), and 1243 (669–3881), respectively and when power on: 3576 (2128–6109), 4290 (2048–4931), and 4135 (2913–6890), respectively.
Rees et al., 2021 [171]	PAPR	Five subjects (Three males, two females)	The mean FF for the PAPR was 1851 (277). The FF was not reduced during the speech, and there were exaggerated maneuvers. It is required that PAPR be equipped with a powered pack to ensure protection for the users.
Sekoguchi et al., 2020 [103]	BL-321S Koken Ltd. PAPR with tight-fitting and half-facepiece respirator	Ten subjects of University of Occupational and Environmental Health (Eight males, two females)	The leakage rate for the RPR was 1.82–10.92% (FF: 9.16–54.94) and 0.18–0.42% (FF: 238.10–555.55) for the PAPR. The performance of the RPR decreased, while the performance of the PAPR was not significantly different.
**Study**	**Respirator Features (Brand, model, size, style)**	**Subject Characteristics**	**Findings**
Sekoguchi et al., 2022 [101]	Two respirators, including SHIGEMATSU WORKS DR77SR2 and SHIGEMATSU WORKS Sy11G2 PAPR	Eight men workplace participants	The GM ±SD WFPs for the C-RPR, U-RPR, and PAPR were 17.7±2.59, 27.0±3.86, and 117.3±5.25.
Temmesfeld et al., 2022 [125]	Novel PAPR	Six subjects (One male, five females) and one mannequin	The TIL for the surgical helmet with a PAPR filter adaptor using a mannequin did not exceed 0.07% (FF: 1428.57) for any particle size at any time of the 23-minute-lasting loading cycle. Also, the mean and maximum TIL obtained from testing on subjects were 0.00465% (FF: 21505.38) and 0.00759% (FF:13175.23), respectively.
Rowlett et al., 2021 [54]	Elastomeric half-mask respirators	327 ASSPs	90% of the participants were familiar with the QNFT procedures. Only a significant difference was found in the perceived accuracy of the QNFT by level of experience (p = 0.006).
Xu et al., 2023 [45]	Four MSA Safety respirators, including two half masks (410 and 420 air-purifying respirators) and two full masks (3S air-purifying respirator and Ultra Elite SCBA)	225 chemical plant operators and maintenance and laboratory personnel	The passing rates were 88.1% for males and 75.6% for females. Most females donned small size respirators due to thin face and sharp chin. Gender had a significant effect on fitting (X^2^ = 5.186, p = 0.023). Other factors had not significant influence on respirator fitting. The half-masks had lower passing rate than the full-masks (84.7 vs. 91.6%, p< 0.05). The 410 and 420 models of APRs (81.6% vs. 86.5%, respectively). The passing rate for 3S APR was 90.0% and for Ultra Elite SCBA was 95.2%.
Rowlett et al., 2021 [54]	Elastomeric half-mask respirators	327 ASSPs	90% of the participants were familiar with the QNFT procedures. Only a significant difference was found in the perceived accuracy of the QNFT by level of experience (p = 0.006).

Note:

FF: Fit Factor

QNFT: Quantitative Fit Test

HCWs: Healthcare workers

AMR: Additively manufactured respirators

IQR: Interquartile Range

CNC: Condensation Nuclei Counter

CPC: Condensation Particle Counter

CNP: Controlled Negative Pressure

HEPA: High Efficiency Particulate Air Filter

NIOSH: The National Institute for Occupational Safety and Health

SSM: silicone-molded face mask

GM±GSD: geometric mean+ geometric standard deviation

PAPR: powered air purifying respirator

JIS: Japan Industrial Standard

RPRs: Replaceable particulate respirators

TIL: Total Inward Leakage

SWPF: simulated workplace protection factor

MAVerIC: Modified Airway from VEntilatoR Circuit

S/M: small/medium

MSM: Modified Snorkeling Mask

AFM: Anaesthesia Face Mask

ICU: Intensive care unit

EHRs: elastomeric half-facepiece respirators / reusable facepiece respirators

APR: air-purifying respirator

SCBA: self-contained breathing apparatus

ND: Not determined

ASSP: American Society of Safety Professionals

USCs: User Seal Checks

**Fig 1:**
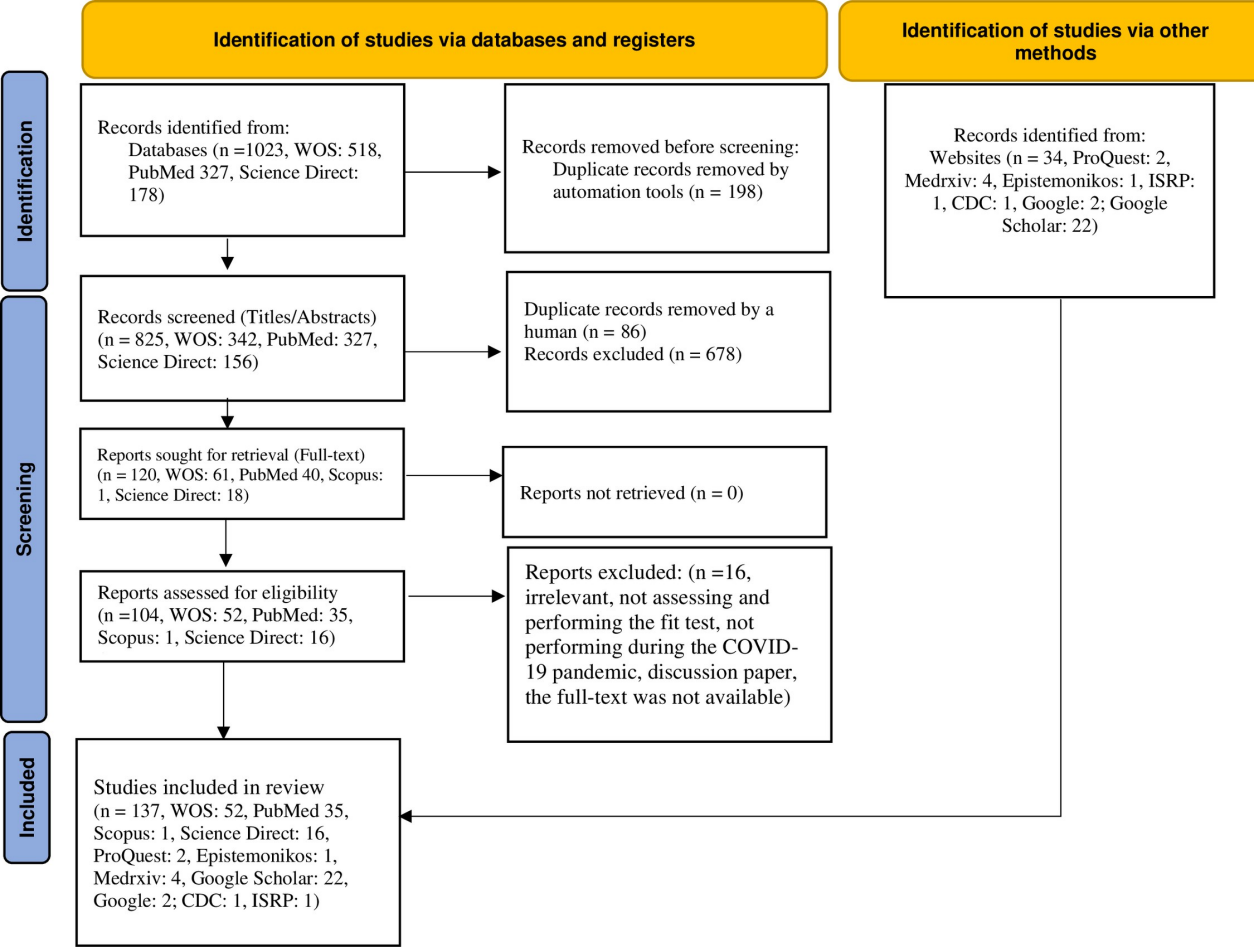
Overview of systematic review execution according to PRISMA 2020 flow diagram.

Then, the quality assessment of included studies was performed using the Newcastle- Ottawa Scale (NOS) checklist for quality assessment of observational cross-sectional studies. To do so, the quality of studies was calculated and categorized into four groups: “Unsatisfactory” (four stars or less), “Satisfactory” (five to six stars), and “Good” (seven to eight stars), and “Very Good” (nine to ten stars) [30, 31]. The results of the study quality assessment were recorded in S3 Appendix. Considerably, any disagreements during screening, eligibility, selection, data extraction, and quality assessment of included studies were resolved by consensus-based discussion between two reviewers or by the decision of the third independent reviewer (J.J). The selection process for study articles is depicted in Fig 1.

## Results

### Study characteristics

**Fig 2:**
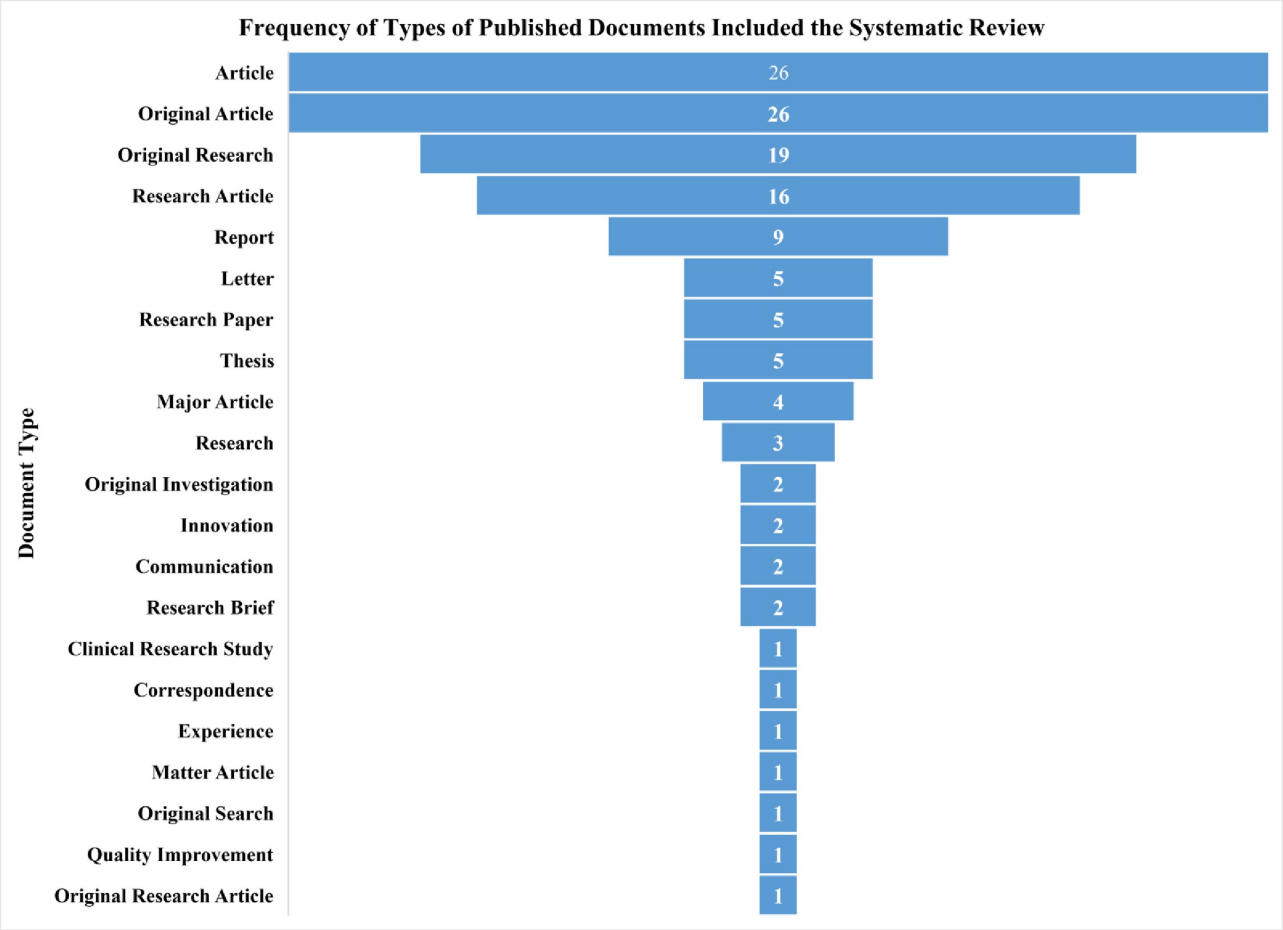
Numbers of published studies on quantitative fit testing during the COVID-19 pandemic.

A total of 137 included studies were performed on quantitative fit testing procedures. Two of the included studies were in Korean, which were translated into English in order not to miss the data in the systematic review [32, 33]. One published online ahead of print research article could not be retrieved [34]. The number of QNFT studies and type of documents that have been published during the COVID-19 pandemic can be depicted in Fig 2. Accordingly, 26 out of 137 (18.98%) studies have been equally published as articles and as original articles, 19 (13.87%) original researches, and 16 (11.68%) research articles, respectively (Fig 2). The document type and study design of the included studies were presented in S4 Appendix.

**Fig 3:**
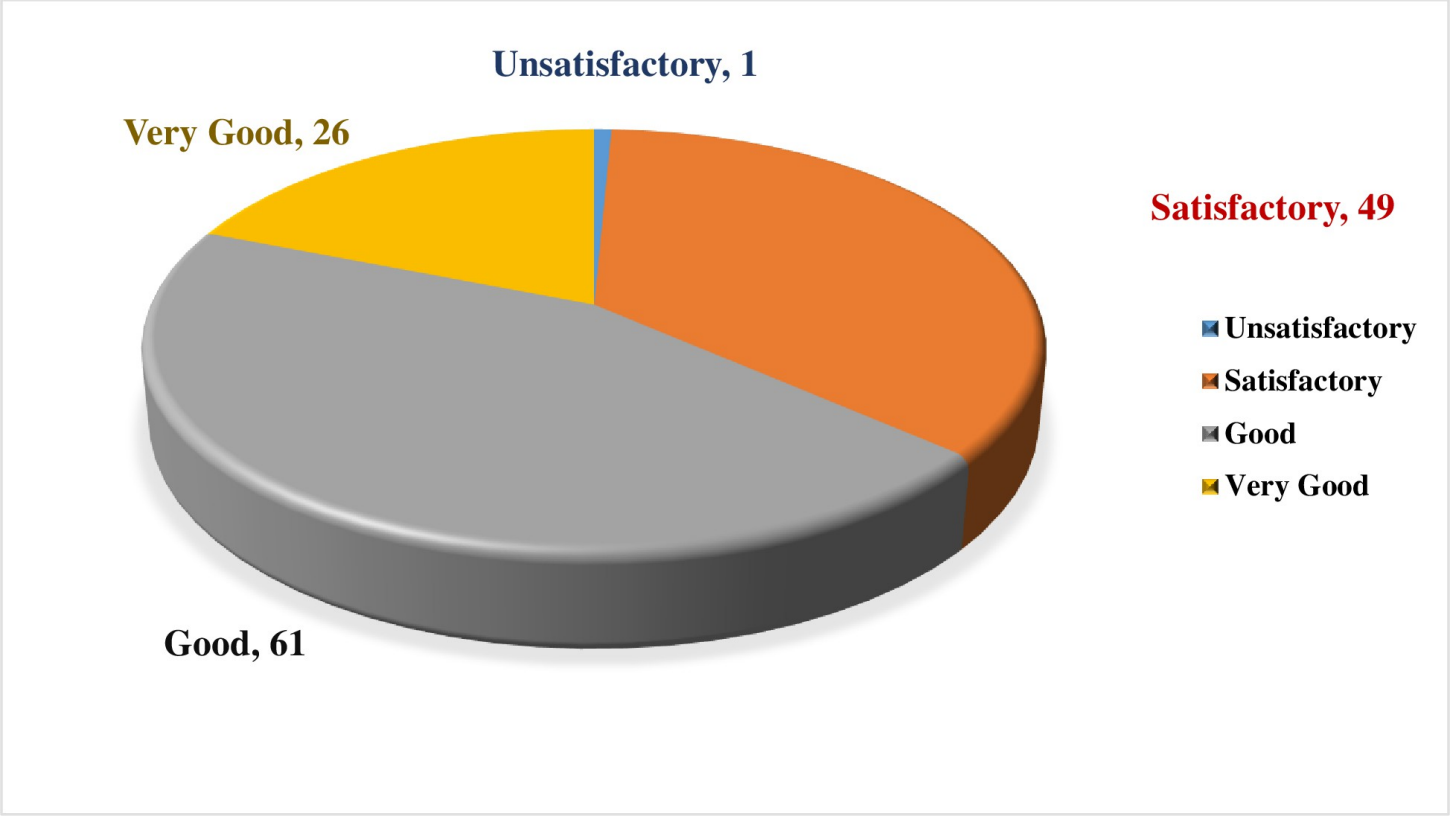
Results of quality assessment of included studies in the systematic review.

According to the results from the quality assessment of the included studies in the systematic review (Fig 3), 44.52% of the studies were classified as “Good” quality and 18.98% were categorized as “Very Good” quality. The results obtained from Fig 3 indicate that 36.50% of the studies did not meet the high-quality score due to reasons such as a lack of study design, sampling strategy, sample size calculation, and statistical analysis (S3 Appendix). Therefore, it seems that researchers need to seriously consider all the aspects and details of the study design and research methodology when developing it.

**Fig 4:**
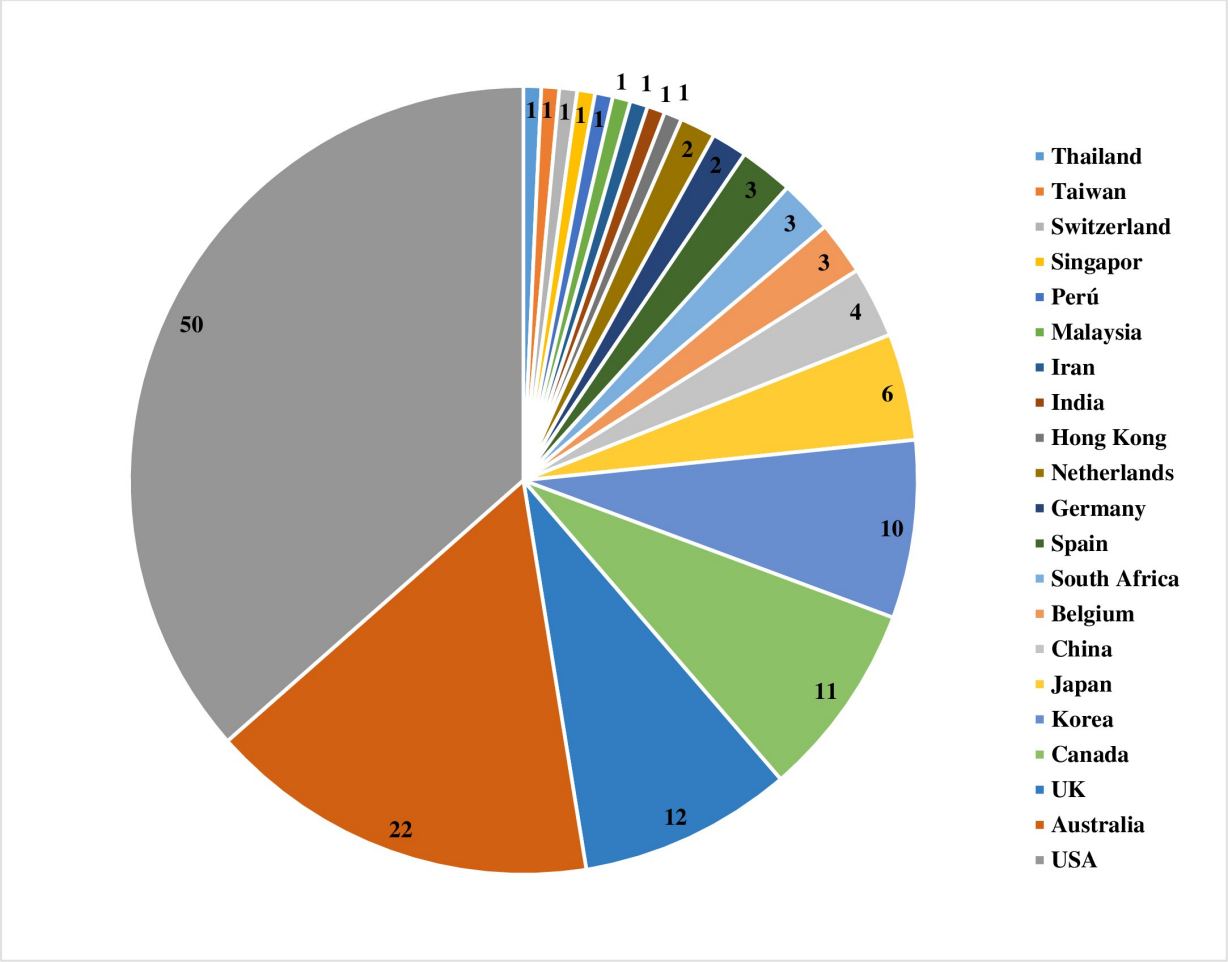
Numbers of studies conducted in different countries during the COVID-19 pandemic.

As can be depicted from Fig 4, all included studies corresponded to a total of 21 countries, including 8 studies (38.1%) performed regarding quantitative fit testing in the developing countries and 13 studies (61.9%) performed in the developed countries. The majority of the studies during the COVID-19 pandemic corresponded to the United States (36.50%) and Australia (16.06%), respectively. It draws the conclusion that fit testing protocols are regulated as one of the legal requirements in developed countries. On the other aspect, it is quite revealing that the implementation of fit testing protocols has been well-established by legal authorities and legislators, manufacturers, employers or managers, and even workplace users in these countries.

**Fig 5:**
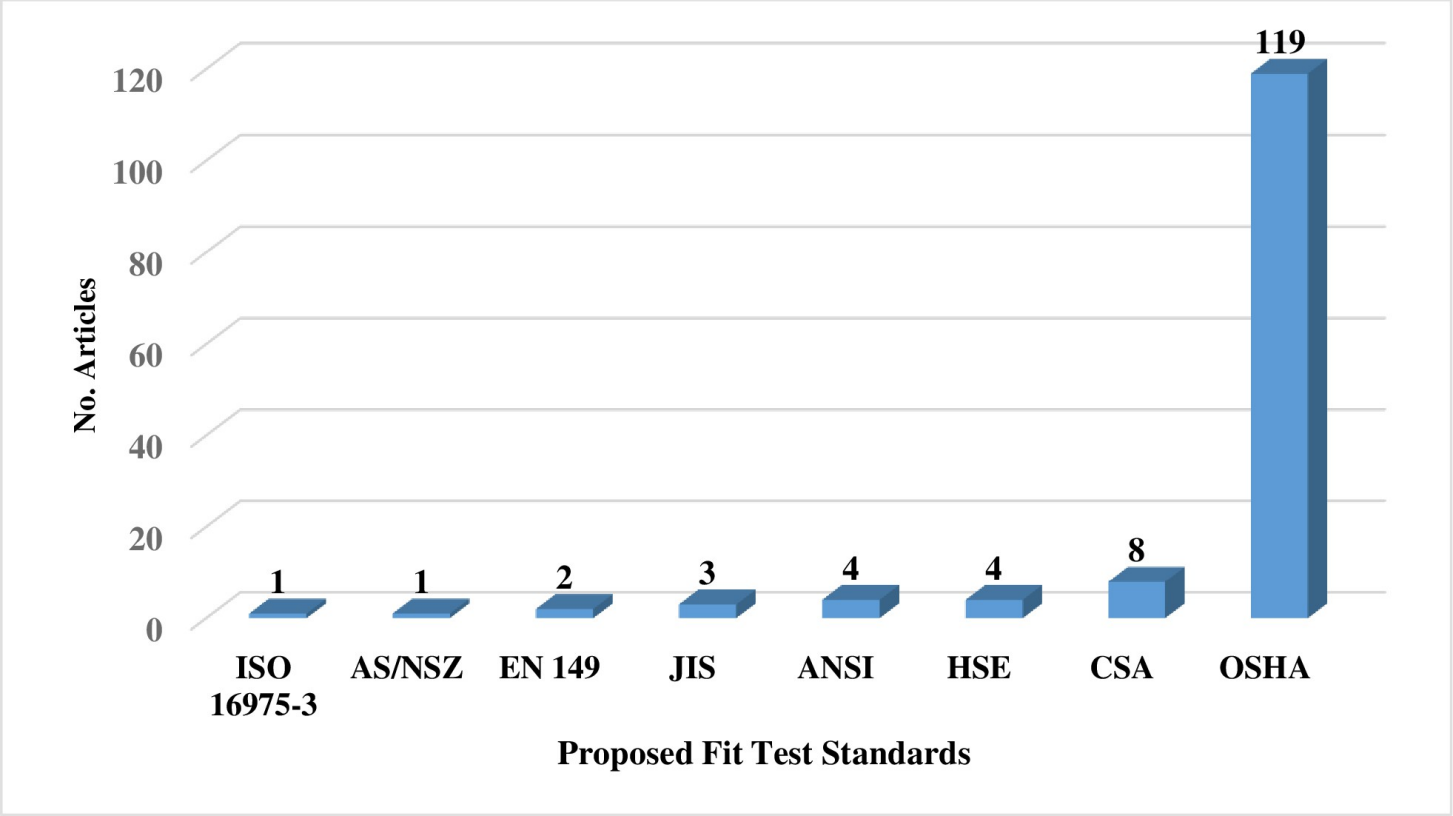
Proposed fit test standards in the included studies.

All fit test standards proposed by the included studies were noted in Fig 5. A considerable proportion of studies proposed the OSHA standard, per regulation 29 CFR 19.10.134. One study did not propose a respiratory protection standard [35]. Three studies proposed both OSHA and ANSI standards [36–38], and one study proposed OSHA and CSA standards [39]. Also, one study proposed the AS/NZS and OSHA standards [40]. It seems that the precise selection and determination of the type of the proposed fit testing standards, protocols, and acceptable FF relevant to the workplace contaminants and proper respirator being assessed (100, 200, 500, 1000, etc.) before implementing the fit testing is so vital. The most striking result to emerge from the Fig 5 is that the adoption of fit test protocols without consideration of a specific fit test standard is impermissible.

**Fig 6:**
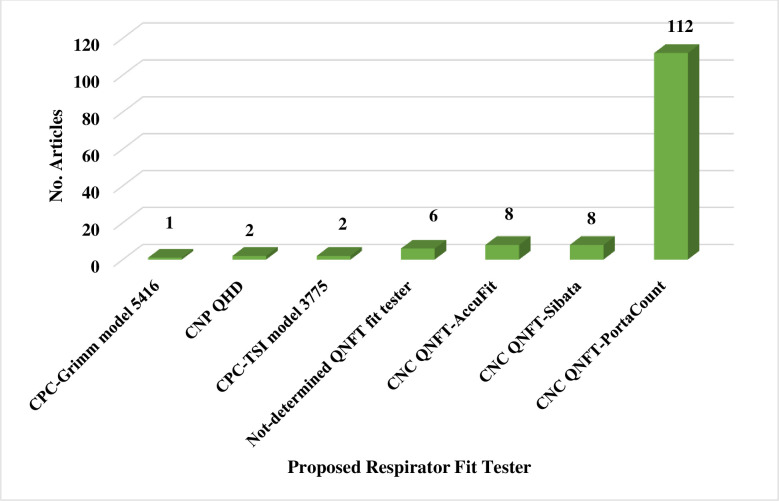
Proposed respirator fit testers by included studies during the COVID-19 pandemic.

The proportions of proposed quantitative fit testing procedures during the COVID-19 outbreak are presented in Fig 6. As observed, the highest proportion of fit testing procedures corresponded to the Condensation Nuclei Counter (CNC)-based PortaCount QNFT protocol (84.21%). After that, CNC-based Sibita and -AccuFit fit testers account for 6.01% and 6.01% of the included studies, respectively.

One study used the Sibita fit tester for measuring the leakage rate≤ 5% and PortaCount fit tester for measuring the FF≥100. Surprisingly, the findings of the PortaCount are consistent with those of the Sibita fit tester in such a way that the N95 respirators had a higher probability of providing protection than the KF94 masks [41]. Regli et al., compared the results of standard PortaCount fit tester model 8038 and modified fast PortaCount model 8048 fit testers. It is somewhat surprising that modified fast protocol led to a higher fit test passing rate than that of the standard fit testing protocol [42].

Another study by Salter et al., utilized the Accufit 9000 and PortaCount 8020 fit testers and found that cloth masks made from available materials with a filtration efficiency of 70–90% could be considered as a safe option during the shortage. Moreover, the Effective Fiber Mask Program (EFMP) was strongly suggested for the mass production of optimized fabric masks [43]. Joshi et al. noted that the TSI PortaCount Pro^+^ model 8038 was comparable to the Grimm Condensation Particle Counter (CPC) fit tester [44]. Fadairo et al. applied the CNC-based TSI PortaCount Pro^+^ model 8038 and Controlled Negative Pressure (CNP)-based (QHD) fit testers. One unanticipated finding was that a significant difference found in the results of CNC fit test protocol under ambient and controlled environmental conditions using the mannequin in contrast to the CNP protocol [36]. Xu et al. assessed the fit testing results of TSI PortaCount model 8038 compared to those QHD Quantifit tester. Surprisingly, there was a significant difference between the CNC and CNP results with respect to facing forward, bending over, shaking the head, wearing the mask again, and moving the head up and down [45]. It is evident that the CNC protocol-based TSI PortaCount fit tester is the best known and most commonly used by researchers compared to the remaining fit test protocols and fit testers.

**Fig 7:**
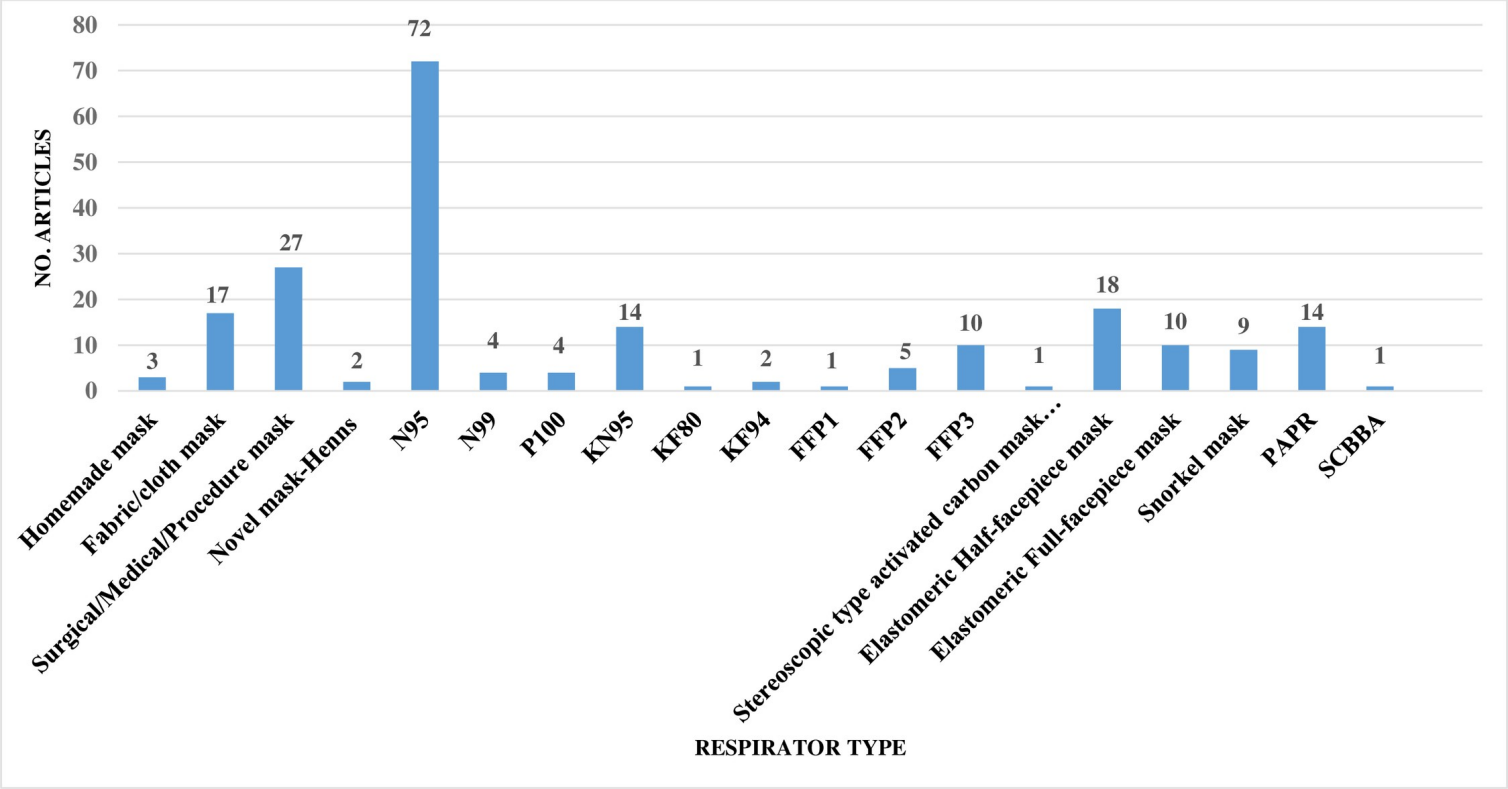
Types of masks and respirators assessed during the COVID-19 pandemic.

The proportion of studies in which evaluated the fitting characteristics of masks or respirators is shown in Fig 7. As can be seen, the highest proportions of studies attributed to the 72 studies on N95 masks, 27 studies on procedure masks or surgical masks; 18 studies on half-facepiece EHRs; 17 studies on cloth or fabric masks; 14 studies on both KN95 respirators and Powered Air Purifying Respirators (PAPRs), respectively. Considerably, ten studies applied three-dimensional (3D) printing materials and rapid prototyping techniques to design and make the half-facepiece filtering and elastomer respirators [46–53]. Two studies did not report the mask and respirator characteristics being utilized [54, 55]. Furthermore, the filter level, brand, and model of the FFRs were not determined in the study by Jean-Romain et al. [56]. It would seem that different masks or respirators may provide different levels of respiratory protection with respect to the subject and the mask or respirator characteristics and the nature of the user’s workplace tasks, so it is not necessary to rely on only one type of respirator to implement fit testing protocols as an essential component of RPP.

### Quantitative fit test studies

The comprehensive reviews of the included studies are presented in Tables 1 and 2. The results were described in more detail in S5 Appendix. A total of 79 studies were conducted regarding disposable masks and or respirators (cloth masks, fabric masks, surgical masks, N95, FFP2, FFP3, KN95, K94, etc.), 49 studies on reusable masks and or respirators (snorkel masks, half-face piece respirators, full-face piece respirators, PAPR, and SCBA), and nine studies concerning knowledge, attitude, perception, skill, and training toward fit testing.

A total of 49803 subjects (comprised of 92 studies) reported in all studies, of which 12391 were males and 35695 were females. Approximately 1717 gender of subjects (45.21%) was not reported. In total of 46 studies did not report the gender proportion. Among them, four studies did not report the number of study subjects in more detail [43, 53, 57, 58], and two studies did not comprehensively and clearly report the number of study subjects [55, 59]. Also, in three studies, no subject characteristics were presented [60–62].

Subjects of 60 studies were HCWs, the professional group’ HCWs were included the following: four studies: anaesthetists and predominantly anaesthetic technicians, anaesthetic consultants and trainees [5, 35, 63, 64], five studies: physicians [65–69], one study: respiratory therapist [66], twenty-one: nurses [37, 40, 41, 59, 66, 67, 69–83], one study: administration [84], four studies: allied health staff [40, 59, 80, 84], seven studies: medical or clinical staff [59, 73, 80, 82, 84–86], one study: paramedic staff [78], three studies: medical practitioner [40, 80, 83], one study: aged care or disability worker [40], two studies: medical imaging staff [40, 80], five studies: other healthcare worker [40, 59, 78–80], five studies: non-clinical role [40, 80, 81, 83, 86], one study: infection control practitioners [87], two studies: laboratory workers [45, 88], eight studies: doctors [41, 70, 71, 76, 78–80, 83], one study: health center workers [89], four studies: dental and dental hygiene students [80, 90–92], one study: emergency medical technician [71], one study: ICU staff members [93], two studies: physiotherapy lecturer [81, 94], one study: radiographer [81], and three studies: pharmacists [40, 80, 95]. The study by Thiam Goh et al. performed on children [96] and other research by Lim et al. conducted on elderly females [97]. One study was conducted on one patient [98]. The study by Xu et al. was conducted on chemical plant operators and maintenance personnel [45].

Other subjects’ studies were as follows: five studies: college or university students [75, 90–92, 99], one study: UK employees [100], two studies: industry workforce or workplace participants [101, 102], two studies: Japan University of Occupational and Environmental Health [103, 104], one study: employees of the National Institute for Occupational Health (NIOH) [105], and one study: American Society of Safety Professionals (ASSP) [54]. Notably, the HCW subjects’ occupations or professional groups were not mentioned in nineteen studies [32, 33, 42, 51, 53, 58, 88, 106–117]. The subjects’ occupations in the remaining 69 studies were not exactly determined. Six studies did not characterize the numbers of study subjects [43, 44, 57, 60–62]. Also, four studies had no human subjects; then, they were categorized as laboratory studies [44, 118–120]. Eight studies were conducted using a manikin, or mannequin, or headform [36, 49, 121–126]. Among all, five studies were performed using human subjects and one manikin [36, 121–123, 125].

The quantitative fit test studies conducted on disposable respirators or masks, including cloth, or fabric, or homemade masks, surgical, or medical, or procedure masks, FFRs (e.g., N95, N99, P100, FFP2, FFP3, KN95, and KF80), and FFs of the cloth and surgical masks compared to those of FFRs in the present systematic review are presented in Table 1.

Accordingly, in Table 1, 56 out of 87 studies reported the mean passing fit testing proportions, of which 38 studies had a fit testing passing rate higher than 50% and 24 studies had a fit testing passing rate higher than 70%. 53 out of 87 studies reported mean FFs, of which 27 studies, including three studies on Cloth masks and 24 studies on FFRs with a mean FF≥ 50, and 19 studies had a mean FF≥ 100. Among these, only 27 studies reported both mean passing fit testing proportions and mean FFs. This finding seems to have highlighted the important role of fit testing in all potentially hazardous situations, for all individuals exposed to all types of respiratory hazards, including but not limited to hazardous workplaces. In the next section, we will present the principal findings of the current investigation regarding the factors influencing the fitting characteristics.

Thirteen studies reported that there were significant differences in passing fit test rates of masks or respirators [39–41, 65, 69, 77, 94, 108, 111, 127–131]. In the study by Martelly et al., the significant level was not reported; however, due to the considerable difference between the two studied respirators, it could be considered as a statistically significant difference in terms of FF (7.0 vs. 143) [129]. Whereas, no significant differences were determined between the studied masks or respirators in the six studies [32, 48, 51, 63, 131, 132]. Due to fit test principle called “OSFA” which stands for there is no one size fits all, fit test results would be unpredictable and each subject could fit with a specific brand, model, style, and size. Also, it seems that the respirator model or brand must be considered as one of the factors influencing fitting characteristics in order to ensure optimal respiratory protection for the users. For example, in the study by Drouillard et al. [39], the average FFE of the control medical mask (55.3%) was lower than that of the fabric mask (64.97%), whereas the Bandana masks exhibited lower FFEs (39.8%-48.1%). It would appear that the material characteristics, such as fabric weight and pore size were significant factors influencing FFE. An increase in fabric weight could result in higher FFE, whereas a reduction in pore diameter could enhance the FFE.

A study found that the N95 respirators had a higher FF than the K94 ones. Also, fixing ear straps with hooks significantly improved respiratory protection rates of KF94 respirators by FF (1.1% vs. 12.8%, p<0.001) [41]. With similar filtration efficiency, particles could leak through the face-seal area; in that case, the poor KF94 respirators would endanger HCWs during AGPs. Application of a hook to fix the loops at the back of the head is considered a fitting improvement strategy. Therefore, a valid fit testing method is highly needed, and a higher-protected PAPR should be substituted if possible [41].

A total of eight studies assessed the influence of respirator style on respirator fitting [33, 70, 80, 82, 95, 107, 128, 130]. In four studies, cup-shaped respirators had the highest passing fit rate [33, 70, 80, 130]. Cup-shaped, duckbill, and flat-fold respirators also had the highest fit test pass rates, respectively in two studies [33, 70]. Ng et al. found that the three-panel flat-fold had a higher passing rate than cup-shaped ones [80]. Contrary to expectations, no significant difference was observed between the respirator styles in the study by Zhang et al. (cup: 57.1% vs. flat-fold: 51.8%) [128]. This inconsistency may be related to the variety of molds that manufacturers are commonly used to produce respirators for the users with different facial dimensions and ethnicities. Besides, application of non-standard or inappropriate commercially available molds by some manufacturers during respirator production may adversely affect the fitting capability and optimal respiratory protection.

Six studies were conducted regarding the effects of extended reuse on fitting characteristics [66, 68, 70, 87, 133, 134]. Sheikh et al. showed that the trend of pass rate was downward from the first attempt to the fourth attempt and upward by the fifth attempt [66]. Fabre et al. estimated all donned N95s less than 12 times, and the probability of an N95 maintaining a good fit was >95% for up to 23 donnings [68]. One study reported that the general respirator protection factor (GRPF) tended to increase or decrease from the previous day as the number of reuses per day increased. Overall, the GRPF for all subjects was lower than the initial GRPF after donning on day 5 [133]. Jung et al. determined that the successive donning led to a reduction in fit test passing rate and thus highlighted that in high-risk situations like those involving aerosol-generating operations, N95 respirators should only be used once and for no longer than one-hour [87]. Contrary to expectations, two studies reported high fit rates after reuse of N95 respirators. It is implied that acceptable fit prior to donning the reused respirators in real healthcare setting should be ensured by implementing a valid fit testing protocol, preferably the QNFT, due to infrequent or false passes in fit testing [68, 70]. However, it would be better to adopt the reuse technique for a short period of time [70].

Ten studies were conducted on the impact of fit test exercise type on fitting characteristics [45, 60, 71, 77, 79, 104, 127, 135–137]. Amongst, three studies evaluated chest compression [71, 79, 135]. Hwang et al. demonstrated that about 73% of the HCWs failed the fit testing on at least one of the three chest compressions [71]. Similarly, Goto et al. confirmed that a high proportion (78%) of the HCWs failed at least one of the three compressions [79]. In contrast, the investigation of the fitting of PAPR during compression by Ng et al. concentrated on the fact that no significant differences were observed in passing the fit testing before, during, or after the chest compression, regardless of the HALO PAPR power mode, which could be considered an alternative to the N95 respirator. Nonetheless, other aspects, including doffing difficulty and perceived communication interference should be paid attention [135], In the Han et al. investigation, the face-nose adhesion also decreased due to the effects of gravity, and the FFs of all three groups of respirators considerably dropped during the waist-bending exercise. When caring for patients who require airborne precautions, healthcare professionals should avoid bending at the waist. This happened because the fit test exercises revealed a variation in fit [77]. The fit test was not passed by talking exercise in the research by Xu et al. [45] and Kechli et al. [60]. The fit test exercises had a greater FF than resting exercises, according to the Baba et al. study [104]. Anwari et al. deemed "Failure" three of the fit test exercises, which involved bending, talking, and side-to-side movement [136]. Likewise, another research pointed out that extensive head and body movements could disrupt the adjustment and fitting of respirator [137]. These findings seriously focused on the fact that unpredictable movements (heavy or light workload) of the subjects while performing the tasks with environmental situations and verbal communications could affect the provision of respiratory protection to the users and increase their exposure to workplace hazards; therefore, the need for proper respirator selection and donning and standard fit testing procedures are strongly suggested.

Eight studies looked at the effect of gender on fitting ability [32, 37, 66, 78, 89, 107, 111, 114, 117], with four of these highlighting that males had a higher pass rate than females [66, 89, 111, 117]. Christopher et al. highlighted the reasons for failing the fit test for females, including facial asymmetry (8%), small bones (77%), and none reported (15%), and the reasons for males, including facial hair (91%), large bones (3%), and small bones (6%) [89]. In the study by Williams et al., although males had a higher passing rate than females; however, females were fitted with the 3M 9320A more often than males (7.3% vs. 1.5%, p< 0.001) [111]. In contrast, some other studies reported that females had a higher pass rate than males [78, 107, 114]. Two studies also found no significant differences in fit test pass rates by gender [32, 37]. Overall, gender appears to be one of the factors influencing fit testing; therefore, providing a variety of respirators in terms of brand, model, style, and size is also of great importance.

Three studies examined the impact of ethnicity on fitting capability [66, 117, 134]. In the study by Sheikh et al., White males or females received a higher FF than those of whether non-White males or females, and then this study addressed that gender and ethnicity should be considered to reflect the diversity of Canadian HCWs [66]. Other studies found that male and White ethnic HCWs were significantly more likely to succeed in fitting compared to females [117, 134]. It would appear that manufacturers are required to design and produce respirators that are relevant to the facial dimensions of their population in terms of gender and age distribution.

Two studies, by Seo et al. and Winski et al., found that face size categories had no effect on fitting [78, 100]. Also, seven studies investigated the influence of facial dimensions on fitting [37, 41, 66, 78, 100, 128, 134]. In the study by De-Yñigo‐Mojado et al., there were significant variations in face length, breadth, and depth between males and females. As a result of the lager face length, depth, and width dimensions in males, as well as the presence of facial hair, the males had lower FFs [37]. In the Seo et al. research, the facial dimensions of the Korean people compared to the NIOSH bivariate panel were significant. However, due to the insignificant difference in passing rates among the face-size groups, it is required to develop a unique fit test panel for the Korean users [78]. Furthermore, in the research of Winski et al., only significant differences were observed between face width and jaw width with FF, and they concluded that increasing the ratio of face width to jaw width (10%) could significantly increase FF [100]. The research of Zhang et al. also showed that bitragion submandibular arc had an inverse relationship and face length had a direct relationship with FF [128]. There was also a slight difference between the fit test results and facial dimensions (e.g., facial length, nasal length and protrusion, alar and biocular width) reported by Sheikh et al. [66]. Park et al. determined that face length, age, department of current work, and career were associated with an adequate protection rate [41]. It seems that taking facial dimensions into account when designing respirators has resulted in optimal production.

Two studies comprehensively evaluated the applicability of the NIOSH bivariate fit test panel to the Korean and American Sheikh populations, [66, 78]. In addition, five studies concluded that the NIOSH bivariate fit test panel may not be representative of the proposed population; thereby, these studies outlined the need to develop the optimal fit test panel representing the facial dimensions is necessary [66, 78, 100, 127, 130]. Additionally, if the fit test results are indistinguishable between the NIOSH cells [78, 100], adjustments must be made. To optimize the NIOSH bivariate panel for the proposed population, facial dimensions relevant to the respirator fitting must be measured, not just face length and width, which are proper predictions of respirator fitting based on the ISO 16976–2 standard [138].

Four studies concluded the negative effects of facial hair on fitting capability [38, 89, 115, 139]. Another point is that the influence of facial hair on the fitting capability of surgical masks was less than that of the FFP3 respirators. The lower FFs for the HCWs without facial hair while wearing surgical masks could be attributed to their cranial shape and facial anatomy [38]. Two factors, including small bone structure for females and facial hair for males, are considered to be the main challenges for fit test failure [89]. Subjects must be clean-shaven prior to fit testing and while donning a tight-fitting respirator; otherwise, positive pressure respirators (such as PAPR, etc.) should be worn [37]. For example, Sandaradura et al. found that the odds of failing the fit test were 1.35 for light stubble, 2.22 for moderate to heavy stubble, and 25 times higher for a full beard than for no facial hair [115]. In the Prince et al. research, the influence of beard length on respirator fitting showed that the rapid inhalation and facial movements associated with speech are likely to cause a loose-fitting mask or respirator to pull toward the face, as opposed to the sealing challenge of a rigid respirator to obtain a tight fit [139]. It appears that facial hair could get stuck under the straps while adjusting the respirator on the face, then it might play as an interfering factor. In that case, subjects feel a false sense of protection whilst inhaling respiratory contaminants through creating a gap between the users’ face and respirator’s facepiece.

Two studies evaluated the effect of age group on fit test results [41, 107]. Park et al. found that age could increase the likelihood of passing the fit test [41]. In contrast, the pass rate for subjects aged 18–29 years was significantly higher than for those aged 30–59 years. Consequently, older age groups and male groups were associated with significantly higher fit test failure rates [107]. In light of the above, it is noteworthy that the age of the subjects is taken into account in the fit test survey.

Ten studies evaluated the impact of user seal checks (USCs) on passing the fit test [52, 59, 63, 64, 68, 71, 88, 102, 140, 141]. There were no similarities between the results of the USCs and fit tests in eight studies [63, 64, 68, 71, 88, 102, 140, 141]. It may seem that the USCs could only detect the gross leakage around the sealing surface area and considered as a proper adjusting the respirator into face; however, users should not fully rely on the USCs; instead, they need to concentrate on the fit test protocols to ensure respiratory protection.

Seven studies evaluated the subjective indices (comfort, usability, activity, speech intelligibility, etc.) regarding the respirators tested [52, 66, 75, 80, 90, 91, 96]. In the research by Ng et al., among four respirator styles, overall comfort and overall assessment values were highest for the three-panel flat-fold respirator and lowest for the semi-rigid cup respirator. To ensure respiratory protection for HCWs, procurement procedures should take into account comfort and usability values, fit testing results, and performance evaluation [80]. According to the Cloet et al. study, the design of a high-performance respirator must take into account activity (breathability and stability) and usability (subjective discomfort, wear efficiency, and speech intelligibility) factors. It is obvious that in addition to the protective factors, ergonomic parameters should be considered during the selection or replacement of a new brand, model, style, or size of respirator [91].

The results of fit testing of reusable masks and respirators are shown in Table 2. A total of 50 studies performed on EHRs were reviewed. A total of 21 studies reported the mean fit test pass rate, of which 18 studies reported a relatively high pass rate (≥50%) and 17 studies reported a high pass rate (≥70%). In addition, 36 studies reported mean FFs, including 25 studies with mean FF≥ 200, 21 studies with mean FF≥ 500, and 19 studies with mean FF≥ 1000. Of these, only 11 studies reported both mean fit test pass rates and mean FFs.

In five studies of reusable respirators, optimal fit was not achieved [46, 53, 141–143]. Despite the fact that all of the 3D-printed prototypes in the Ballard et al. study were built of flexible materials, three of them failed to offer an acceptable fit into the facial dimensions of four individuals. Importantly, fit testing procedures must be conducted on a sufficient sample of consumers in order to make adjustments to the prototypes that have been put to the test feasible [142]. In another study by Ballard et al., 3D-printed prototypes equipped with only HEPA filters could pass the fit test. This finding concentrated on the fact that the type of filter used for fit testing of EHRs is of great value, because improper filters lead to the leakage and provide a false sense of protection [53]. Duda et al. noted that the studied 3D-printed face masks could not be used in clinical settings. The main causes of this are leakages associated with the connection of the masks with the filter material, particularly unwanted leakages brought on by the simplified filter box construction, as well as the low flexibility of the material and the thin sealing line, which prevent the necessary sealing performance on the face [46]. It is undeniable that the respirators with 3D-printed designs are made of subtle, heavy, and complicated components with different materials, and components’ connections. In this regard, it is masterwork and hard challenging to achieve an acceptable fit.

In the study by Martelly et al., molding a reusable respirator could serve as another strategy to improve fitting and be utilized as a safe substitution during the shortage of N95 respirators. Accordingly, one key factor in obtaining proper respirator fitting is the strap tension and orientation. Keeping the top strap from sliding to the back of the head caused problems for the subjects with short and smooth hair, which in turn influenced the fitting during fit testing. Other subjects with long or short, textured hair keep the strap from sliding by either using a ponytail or friction [129]. It is evident that the subjects’ hairstyle acted as an interference factor, causing the head straps to slip and loosen, thereby disrupting the proper fit.

Fifteen studies were performed regarding the reusable respirators compared to the FFRs [48, 51, 53, 58, 61, 63, 67, 69, 93, 94, 99, 112, 127, 137, 142]. All those studies reported that reusable respirators achieved a higher passing fit test rate than those of FFRs. The novel Duo mask, consisting of two inhalation valves, one exhalation valve, and two filters, reduced inspiratory resistance and dead space while prolonging the service life of filter [127]. Ballard et al. remarked that the 3D-prototype respirator is a desirable alternative to the N95 respirator when achieving the optimal fitting is impossible [142].

The Stick-on mask Lekad improved FF by 40, 35, and 30 times compared to surgical, double, and N95 masks. The Duo mask showed a higher FF than N95, suggesting disposable respirators could replace reusable masks in terms of bidirectional protection requirements and cost-benefit analysis [94]. It is strongly suggested to compare the fitting characteristics of novel reusable respirators to those of traditional EHRs or FFRs to undergo various fit testing procedures (CNC vs. CNP) in order to learn and understand about the variations, restrictions, and FFs offered to users with various anatomical features. For example, in the Nicholson et al. study, a full-face respirator was compared with three different types of Snorkel masks, with comparable results [144]. It would seem that a series of prototype designs using various molds and multi-system sizes may overcome the technical difficulties and create respirators that could serve the intended market. Nonetheless, it is strongly recommended that modified commercial respirators due to unstable protection be required to undergo rigorous testing to ensure that the HCWs remain protected.

Additionally, it is preferred that respirators be evaluated when employees are doing duties in actual workplaces or simulating work processes as part of a fit test exercise for SWPF or WPF evaluation to ensure the optimum protection. Besides, not only is the performance evaluation of commercial, modified, or newly developed respirators critical to meeting the standard criteria, but comfort, usability, and activity evaluations are also highly recommended. Since disposable respirators were lighter, they were more comfortable than reusable respirators for a short period of time; however, some limitations, such as a lower level of protection and variability of protection rate due to structural damage or prolonged use or reuse, possible contamination of the outside of the respirator, a lower filtration level and unacceptable fit, and the inability to be worn by individuals with asthmatic, cardiovascular, and hypertensive diseases, etc., could occur.

A total of twelve studies, including eleven studies on disposable respirators (Table 1) and one study on reusable respirators (Table 2) were assessed the influence of fit testing procedure on attitude, knowledge, perception, or training in fit testing. A total of were conducted. Three studies were conducted on knowledge [55, 72, 92], three studies on attitude [55, 59, 72], two studies on perception [54, 92], one study on skills [59], and six studies assessing the influence of training on fit testing [33, 81, 83, 84, 140, 145]. Accordingly, in two studies, knowledge [55, 59], in one study, attitude [55], in two studies, perception [54, 92], in one study, skills [59], and in six studies, training [33, 81, 83, 84, 140, 145] regarding the fit testing improved. Training plans (online or visual inspection of respirator fit and verbal suggestions for adjustment) could improve knowledge, attitude, perception, skill level in properly donning the respirator, and the importance of performing fit tests, resulting in reliable fit test results and passing the fit test.

## Discussion

The present study aimed to evaluate the fitting capability of all kinds of masks and respirators and explore the relationship between mask or respirator fitting and affective factors during the COVID-19 pandemic. Some key findings obtained from this study are presented below.

According to the risk of bias assessment, although 50 (36.50%) out of 137 studies, except for one, possessed an acceptable quality score. However, those studies have some considerable weaknesses in terms of study design and methodology. To do so, this investigation informs specialists and researchers that before developing a study on respiratory protection, all aspects and research process steps must be deeply considered. Some important values that were neglected and need to be improved in the studies are as follows: acceptable sample size (calculation, justification), type of study (experimental, cross-sectional, observational, etc.), study design (blinding, randomization, control group), subject characteristics (number, gender, occupation, age, BMI, facial dimensions, etc.), respirator features (filtration level, brand, style, size), and exact and full reports of study findings.

In this review, 31 out of 87 studies (35.63%) and 34 out of 87 studies (39.08%) conducted on disposable masks or respirators did not report the mean fit test pass rate and mean FF, respectively. Similarly, 29 out of 50 studies (58%) and 14 out of 50 studies (28%) on reusable respirators did not report the mean fit test pass rate and mean FF, respectively. This issue was a major concern among the studies, so it is highly necessary that researchers report the results more clearly and comprehensively to enhance the importance and value of the study and make those results more useful and convincing to the relevant readers or users.

Among the reported studies on disposable masks, 18 out of 56 studies had a pass rate lower than 50%, 26 out of 53 studies had an FF lower than 50, and 31 out of 50 studies had an FF lower than 100, respectively. It concluded that the fit test failure rate in these studies was relatively high. Providing multiple brands, styles, or sizes could benefit respirator users achieve an optimal FF. Also, among the reported studies on reusable masks, 3 out of 21 and 4 out of 21 studies had pass rates lower than 50% and 70%, respectively. 11 out of 36 studies had an FF lower than 200, and 17 out of 36 studies had an FF lower than 1000. It can be found that most of the studies had an acceptable fit test pass rate (≥50%) and FF (≥200). Because there are a considerable number of non-reported studies, the final decision on the results of all studies would be challenging.

One possible reason for the low passing rate among these studies could be due to the limited supply of standard masks and respirators, such as N95 types, in order to provide optimal fitting for the users with high-risk duties (e.g., AGPs); in particular, for the HCWs exposed to suspected or confirmed COVID-19 patients. Moreover, it might be that only one size or one style of masks or respirators underwent fit testing procedures. To overcome this issue, according to the principle “there is no OSFA respirator”, every user could not be fitted into a respirator of a specific brand, model, style, and size; therefore, managers and employers are required to provide a variety of respirators with combinations of brands, models, styles, and sizes to ensure the satisfactory protection for the workers [172]. Another reason could be that those studies rely on only the filtration efficiency; the respirator fitting into anthropometric dimensions as one of the affective factors on respiratory protection has been neglected [7, 16].

Respirator type and brand were reported to have a significant effect on respirator fit. Overall, all disposable and reusable respirators had a specific structure and design that could affect their fitting characteristics, e.g., material characteristics, including rigid or soft materials, fabric or filter weight, pore size, and number of layers; and design factors, such as head straps, nose clips, and ear fixation; and inspiratory and expiratory valves, are affective factors on optimum fitting. Unexpected leakage from component connections or installations (inflexible or heavy molds, valves, and straps) was considered a notable concern [46]. Clogging and disinfection are other challenges that increase backpressure, ultimately resulting in a decrease in FFs [137].

In the study by Ballard et al., a well-fitting EHR equipped with HEPA filters (high filtration level) could be comparable to the commercial N95 respirator [53]. Roche et al. demonstrated that a 3D-printed respirator would be comparable to the FFP3 without compromising verbal communication [51]. Germonpre et al. outlined that a modified snorkel mask with 3D-printed adaptors could outperform the N95 fit and be superior to temporary adaptations [58]. Another study found that the N95 respirator and snorkel mask with high-efficiency filters could provide inconsistent protection compared to the snorkel mask with PAPR. Therefore, robust testing is needed to assure the protection of the HCWs [69]. One of the disadvantages is that although the PAPR could obtain a consistent and adequate level of respiratory protection during compression, it could create doffing difficulty and communication performance interference [135]. Another study stated that a 3D-printed respirator could be utilized when subjects do not pass the fit testing of a commercial N95 respirator due to style, size, or variations in face morphology. Particularly, it could be an appropriate alternative to disposable respirators due to continuous failure of the fit test following the adoption of reuse and disinfection procedures [142].

Another finding was that the cup-shaped respirators fitted more than all styles. Likewise, the cup-shaped activated carbon was considered the best option for filtering anticancer drugs in a clinical setting [95]. Since each user has a specific face shape (anatomical structure, hollow, protrusion, etc.) with regards to BMI, ethnicity, age, etc., it is necessary to provide a variety of respirators for fit testing to identify the best fit option in terms of protective and ergonomic aspects. As the number of models, sizes, or styles increases, the likelihood of subjects succeeding in fit testing increases. Ciotti et al. stated the cup-shaped respirators were more suitable for HCWs with large faces [173]. However, the three-panel flat-fold style was more fitted into the anthropometric dimensions of the Australian HCWs with the highest comfort and usability scores [130]. It is recommended that manufacturers design and make masks or respirators following approaches towards multiple-size-systems (3-, 4-, and 5-size) [174] instead of single-size system (OSFA) and various styles [173] (cup-shaped, flat-fold, and duckbill) to fit the proposed users, including HCWs, industrial workers, etc.

Extensive reuse was reported as another factor influencing respirator fit. Given that continuous and repeated donning of the respirator over several days will impede the quality of fitting due to possible contamination or deterioration of the respirator’s components. Nevertheless, fit testing of reused respirators prior to entry into hazardous workplaces is essential. Subjects with high-risk occupations should be cautious about excessive movement. Notably, a properly fitted respirator would not provide protection for the HCWs, thereby impairing the protective performance of the respirator, because chest compression during CPR requires significantly rapid, intense, and dynamic upper body movements that are more dynamic than QNFT exercise [71]. To ensure the provision of respirators in real situations (e.g., emergencies), it is strongly suggested that investigators adopt fit test protocols in a simulated scenario such as chest compressions. If it is necessary, the respirators will be changed or effective control strategies will be implemented in the workplace.

Differences in anthropometrical dimensions between females and males could considerably affect the results of respirator fit testing [117]. Given that the design and production of RPE are mostly based on males’ dimensions. Proper selection and certification of RPE is so hard-working. Due to the specific effect of gender on respirator fit, careful attention must be paid to the design and selection of respirators that are appropriate for their facial dimensions.

The facial dimension is another affective factor. An optimal and unique respirator fit test panel (RFTP) based on the facial dimensions of the proposed population should be developed before respirator design, certification, and selection [175–179]. This issue could assess the procurement decision-making procedures for respirator stocking, preventing poor respirator supply and the scarcity of correctly sized respirators [117].

Comfort, usability, and activity indices are three paramount factors in determining the respirator fitting. Moreover, as part of a comprehensive RPP, four classifications of RPE characteristics are taken into account, including "safe and effective; compatible with work activities; comfortable and tolerable for the duration of wear; and compliant with relevant standards, guidelines, and policies", which benefit from proper respirator evaluation and selection [180]. The necessity of implementing fit testing and performance evaluation of HCWs when making procurement decisions was emphasized by Ng et al. In the interim, wearer compliance, respirator fitting, and purchasing decisions are influenced by the fit test passing rate, usability, and comfort evaluations [80]. Training on proper selection, donning and doffing, and the importance of fit testing protocols could improve the subjects’ knowledge, perception, attitude, skill, and experience toward respirator fit testing compared to the pre-fit test steps [59, 76, 83, 161].

### Limitations

The included studies lack the appropriate or proper study design, sampling strategy, sample size calculation, statistical analysis, and study procedure (e.g., fit testing of respirators with various brands, models, styles, and sizes). Another limitation is that some studies did not report the features of the respirators (brand, style, size, filtration level, etc.) or subjects (gender, age, occupation; high/low physical workload, etc.) being fit tested. Furthermore, small sample sizes are another weakness. In this study, a comprehensive systematic review was conducted to evaluate the fitting capability of all kinds of masks and respirators and to explore the relationship between respirator fitting and affective factors during the COVID-19 pandemic.

## Conclusion

37.36% of the disposable respirator studies and 43% of the reusable respirator studies did not report fit test results. 67.86% of the disposable respirator studies had a fit test pass rate greater than 50%, and 35.84% of these studies had an FF greater than 100. Also, 85.71% of the reusable respirator studies had a fit test pass rate greater than 50%, and 52.77% of these studies had an FF greater than 1000. Overall, the fit test pass rate was relatively acceptable. Newly developed or modified respirators must undergo reliable testing to ensure the protection of HCWs. Subject and respirator characteristics should be considered when implementing fit testing protocols. An optimal fit test panel should be developed prior to respirator design, certification, procurement decisions, and selection procedures.

## Supporting information

S1 Appendix PRISMA checklist 2020. (DOCX)

S2 Appendix Search strategy & excluded papers. (DOCX)

S3 Appendix Quality assessment. (DOCX)

S4 Appendix Study design and paper type. (DOCX)

S5 Appendix Comprehensive results. (DOCX)
