# Supplementary material for: A systematic review of passing fit testing of the masks and respirators used during the COVID-19 pandemic: Part 1-quantitative fit test procedures
Source: PLoS One. 2023 Oct 26;18(10):e0293129. doi: 10.1371/journal.pone.0293129 (PMC10602271; doi:10.1371/journal.pone.0293129)
Supplement: S2 Appendix — (DOCX) [file pone.0293129.s002.docx]

**S2 Appendix. Search Strategy & Excluded Papers**

**Search Strategy**

Mask OR Respirator OR Personal Protective Equipment OR respiratory protective device OR Protective Device, Respiratory Protective Equipment, Respiratory Protective Device) AND (Quantitative Fit Test, Condensation nuclei counter, Controlled negative pressure, PortaCount, Sibata, Accufit, Fit, Seal), AND (COVID-19, and Coronavirus, and SARS-CoV-2.

**Table 1. Lists of Excluded Articles**

| **No.** | **Author, Year** | **Article Title** | **Exclusion’s reason** |
| --- | --- | --- | --- |
| 1 | Check et al., 2021 [1] | Failure Rates During Reuse of Disposable N95 Masks in Clinical Practice in the Emergency Department | No quantitative fit test (QNFT) was performed in this study. |
| 2 | Coffey et al., 2019 [2] | The Respirator Fit Capability Test: Enhancing the Efficacy of Filtering Facepiece Respirators | No QNFT was performed in this study. |
| 3 | Huage et al., 2012 [3] | Real-time fit of a respirator during simulated health care tasks | This study was not performed during the COVID-19 pandemic. |
| 4 | Charm et al, 2010 [4] | The fitness of N95 respirators among undergraduate Chinese nursing students in Hong Kong | This study was not performed during the COVID-19 pandemic. Also, the full-text was not available |
| 5 | Holland, 2020 [5] | COVID-19 Personal Protective Equipment (PPE) for the emergency physician | This study (discussion paper) was not performed during the COVID-19 pandemic, |
| 6 | Ohara et al., 2022 [6] | Fabrication of a highly protective 3D-printed mask and evaluation of its viral filtration efficiency using a human head mannequin | No QNFT was performed in this study. |
| 7 | Segura et al., 2020 [7] | Comparison of Two Respirator User Training Methods:Video and One-On-One Training + | The full-text was not available. |
| 8 | Ganesan et al., 2021 [8] | N95 or P2 respirator fit testing policy in Australia: implementation issues to consider. Medical Journal of Australia | No QNFT was performed in this study, letter. |
| 9 | Ippolito et al., 2021 [9] | Personal protective equipment use by healthcare workers in intensive care unit during the early phase of COVID-19 pandemic in Italy: a secondary analysis of the PPE-SAFE survey | No QNFT was performed in this study. Also, no provide complete data on QNFT. |
| 10 | Stannard et al., 2022 [10] | Evaluating the fit-effectiveness of fabric-based reusable face masks on 3D printed NIOSH headforms | No formal fit test was conducted in this study (subjective study). |
| **No.** | **Author, Year** | **Article Title** | **Exclusion’s reason** |
| 11 | Siah et al., 2022 [11] | Using infrared imaging and deep learning in fit-checking of respiratory protective devices among healthcare professionals | No provide complete data on QNFT. |
| 12 | Loeb et al., 2022 [12] | Medical Masks Versus N95 Respirators for Preventing COVID-19 Among Health Care Workers | No fit testing procedure was conducted. |
| 13 | Youness et al., 2020 [13] | Assessment of N-95 Facemask for Use in COVID-19 Pandemic in Case of Shortage of Personal Protective Equipment | The complete data was not provided. |
| 14 | Scully et al., 2022 [14] | Enhancing respiratory protection in skilled nursing facilities during the COVID-19 pandemic: A public health fit-test training program | The complete data was not provided. |
| 15 | Sucipta et al., 2023 [15] | Development of respirator design for children using bamboo-based activated carbon filter and bipolar ionization | The QLFT fit test was performed. |
| 16 | Yeung et al., 2020 [16] | Assessment of Proficiency of N95 Mask Donning Among the General Public in Singapore (Mask Fit Test) | No formal fit test was conducted (subjective) in this study. |
