# Supplementary material for: A systematic review of passing fit testing of the masks and respirators used during the COVID-19 pandemic: Part 1-quantitative fit test procedures
Source: PLoS One. 2023 Oct 26;18(10):e0293129. doi: 10.1371/journal.pone.0293129 (PMC10602271; doi:10.1371/journal.pone.0293129)
Supplement: S3 Appendix — (DOCX) [file pone.0293129.s003.docx]

**S3 Appendix. Overview of All the Studies Included in This Systematic Review and Quality Assessment of Studied Based on NEWCASTLE –QTTAWA**

| **No.** | **First author** | **Title** | **Selection** | **Comparability** | **Outcome** | **Quality**  **Score** |
| --- | --- | --- | --- | --- | --- | --- |
|  | Brandel et al., 2020 [1] | Testing and Developing DIY Masks | 2 | 1 | 2 | 5 |
|  | Buckley et al., 2020 [2] | Quantitative Respirator Fit Testing of HensMask | 2 | 1 | 2 | 5 |
|  | Coyle et al., 2021 [3] | Reduction of exposure to simulated respiratory aerosols using ventilation, physical distancing, and universal masking | 4 | 1 | 3 | 8 |
|  | Dang et al., 2021 [4] | Challenges in Predicting the Filtration Performance  of a Novel Sewn Mask: Scale-up from Filter Holder  to Mannequin Measurements | 3 | 1 | 2 | 6 |
|  | Drouillard et al., 2022 [5] | Fitted filtration efficiency and breathability of 2-ply cotton masks: Identification of cotton consumer categories acceptable for homemade cloth mask construction | 3 | 1 | 3 | 7 |
|  | Duncan et al., 2021 [6] | The protective performance of reusable cloth face masks, disposable procedure masks, KN95 masks and N95 respirators: Filtration and total inward leakage | 3 | 1 | 3 | 7 |
|  | Mueller et al., 2020 [7] | The protective performance of reusable cloth face masks, disposable procedure masks, KN95 masks and N95 respirators: Filtration and total inward leakage | 3 | 1 | 3 | 7 |
|  | Reutman et al., 2021 [8] | Quantitative Method for Comparative Assessment of Particle Removal Efficiency of Fabric Masks as Alternatives to Standard  Surgical Masks for PPE | 2 | 1 | 2 | 5 |
|  | Teesing et al., 2020 [9] | Homemade facemasks: particle filtration, breathability, fit, and other performance characteristics | 2 | 1 | 3 | 6 |
|  | Wentworth et al., 2020 [10] | Advanced Manufacturing Center_Comparative Protective Mask  Material Testing | 3 | 1 | 2 | 6 |
|  | Lindsley et al., 2021 [11] | Efficacy of face masks, neck gaiters and face  shields for reducing the expulsion of simulated  cough-generated aerosols | 3 | 1 | 3 | 7 |
|  | Sato et al., 2020 [12] | Measurement of the leak rate of masks used for anticancer drug handling using a mask fitting tester | 3 | 1 | 3 | 7 |
|  | Ardon-Dryer et al., 2021 [13] | Mask Material Filtration Efficiency and Mask Fitting  at the Crossroads: Implications during Pandemic Times | 2 | 1 | 2 | 5 |
|  | Bodas et al., 2022 [14] | A randomised crossover trial of two flat-foldcup respirators: BYD DE2322 N95 versus Care Essentials MSK-002 P2 | 5 | 1 | 3 | 9 |
|  | Cameron et al., 2020  [15] | Quantitative fit testing with limited supplies of respirator masks in hospital personnel during the COVID-19 pandemic | 3 | 1 | 2 | 6 |
|  | Chan et al., 2021 [16] | Fit Testing Disposable P2/N95 Respirators during COVID-19 in Victoria, Australia: Fit Check Evaluation, Failure Rates, and a Survey of Healthcare Workers | 4 | 1 | 3 | 8 |
|  | Christopher et al., 2021 [17] | The PPE Pandemic: Sex-Related Discrepancies of N95 Mask Fit | 3 | 1 | 3 | 7 |
|  | Cloet et al., 2022 [18] | Design considerations for protective mask development: A remote mask usability evaluation | 4 | 1 | 3 | 8 |
|  | Cloet et al., 2022 [19] | Activity And Usability Evaluation Of Alternative  Protective Face Mask Designs | 3 | 1 | 2 | 6 |
|  | Griffin et al., 2022 [20] | Protective Masks Utilizing Nonendangered Components | 4 | 1 | 2 | 7 |
|  | Duncan et al., 2020 [21] | The impact of extreme reuse and extended wear  conditions on protection provided by a surgicalstyle  N95 filtering facepiece respirator | 4 | 1 | 3 | 8 |
|  | Fabre et al., 2021 [22] | N95 Filtering Facepiece Respirators Remain Effective After Extensive Reuse During the COVID-19 Pandemic | 3 | 1 | 3 | 7 |
|  | Nakamoto et al., 2021 [23] | Reusing N95 Respirators at Weekly Intervals During the COVID-19 Pandemic | 3 | 1 | 2 | 6 |
|  | Greenawald et al., 2021 [24] | Evaluation of Fit and Strap Extension Performance of Stockpiled Filtering Facepiece Respirators from One U.S. Facility | 4 | 1 | 3 | 8 |
|  | Hai et al., 2022 [25] | Comparing Quality of Fitting of Different Filtering Face Pieces in a Healthcare Worker: A preliminary observational study | 2 | 1 | 3 | 6 |
|  | Han et al., 2021 [26] | Comparison of N95 Respiratory Mask Fit Testing | 3 | 1 | 3 | 7 |
|  | Hwang et al., 2020 [27] | N95 filtering facepiece respirators do not reliably afford respiratory  protection during chest compression: A simulation study | 5 | 1 | 3 | 9 |
|  | Fakherpour et al., 2021 [28] | Quantitative fit testing of filtering face-piece respirators  during the COVID-19 pandemic reveals anthropometric deficits  in most respirators available in Iran | 3 | 2 | 3 | 8 |
|  | Jankusol et al., 2023 [29] | Different fit factors for the N95 respirator during endotracheal intubation: Comparing video laryngoscopy and direct laryngoscopy | 5 | 1 | 3 | 9 |
|  | Joshi et al., 2021 [30] | Quick laboratory methodology for determining the particle filtration efficiency of face masks/ respirators in the wake of COVID-19 pandemic | 2 | 1 | 2 | 5 |
|  | Jean-Romain et al., 2021 [31] | Distribution of low quality filtering facepiece  respirators during the COVID-19 pandemic: an  independent analysis of the situation in Switzerland | 2 | 1 | 2 | 5 |
|  | Jung et al., 2021 [32] | Fit-failure rate associated with simulated reuse and extended use of N95 respirators assessed by a quantitative fit test | 2 | 1 | 2 | 5 |
|  | Kamal et al., 2023 [33] | Safety Goggles with Elastic Headband to Improve N95 Fit Following Failed Quantitative Fit Test | 5 | 1 | 3 | 9 |
|  | Kyaw et al., 2021 [34] | Prediction of N95 Respirator Fit from Fogging of Eyeglasses: A Pilot Study | 4 | 1 | 3 | 8 |
|  | Landry et al., 2022 [35] | Fit-Tested N95 Masks Combined With Portable  High-Efficiency Particulate Air Filtration Can  Protect Against High Aerosolized Viral Loads Over  Prolonged Periods at Close Range | 3 | 1 | 3 | 7 |
|  | Lindsley et al., 2021 [36] | A comparison of performance metrics for cloth face masks as source control devices for simulated cough and exhalation aerosols | 2 | 1 | 2 | 5 |
|  | Long et al., 2022 [37] | Measurement of filtration efficiencies of healthcare and consumer materials using  modified respirator fit tester setup | 4 | 1 | 3 | 8 |
|  | Milosevic et al., 2021 [38] | P2/N95 filtering facepiece respirators: Results of a large-scale quantitative mask fit testing program in Australian health care workers | 4 | 2 | 3 | 9 |
|  | Ng et al., 2022 [39] | N95 respirators: quantitative fit test pass rates and usability and comfort assessment by health care workers | 5 | 1 | 3 | 9 |
|  | O’Kelly et al., 2021 [40] | How well do face masks protect the wearer compared to public perceptions? | 2 | 1 | 2 | 5 |
|  | O’Kelly et al. 2021 [41] | Comparing the fit of N95, KN95, surgical, and cloth face masks and assessing the accuracy of fit checking | 2 | 1 | 2 | 5 |
|  | O’Kelly et al., 2022 [42] | Experimental Measurement of the Size of Gaps Required to Compromise Fit of an N95 Respirator | 3 | 1 | 2 | 6 |
|  | Park et al., 2021 [43] | Fit Test for N95 Filtering Facepiece Respirators and KF94 Masks for Healthcare Workers: a Prospective Single-center Simulation Study | 3 | 1 | 3 | 7 |
|  | Popov et al., 2022 [44] | Development and Application of a Modified Procedure for Quantitative Fit Testing of Disposable Masks and Respirators. Journal of occupational and environmental hygiene. | 3 | 1 | 2 | 6 |
|  | Regli et al., 2022 [45] | N95 Masks to Protect Health Care Workers  Is the New Fast Fit-Test Protocol Cutting Corners? | 2 | 1 | 2 | 5 |
|  | Regli et al., 2021 [46] | More than half of front-line healthcare workers unknowingly used an N95/P2 mask without adequate airborne protection: An audit in a tertiary institution | 3 | 1 | 3 | 7 |
|  | Prince et al., 2021 [47] | Assessing the effect of beard hair lengths on face masks used as personal protective equipment during the COVID-19 pandemic | 3 | 1 | 2 | 6 |
|  | Sandaradura et al., 2020 [48] | A close shave? Performance of P2/N95 respirators in healthcare workers with facial hair: results of the BEARDS (BEnchmarking Adequate Respiratory DefenceS) study | 5 | 1 | 3 | 9 |
|  | De-Yñigo-Mojado et al., 2021 [49] | Facial Hair Decreases Fit Factor of Masks and Respirators in Healthcare Providers | 5 | 1 | 3 | 9 |
|  | Sasko et al., 2023 [50] | Fit testing of masks worn by frontline healthcare workers | 3 | 1 | 2 | 6 |
|  | Seo et al., 2021 [51] | Necessity of the Fit Test Panel for Korean Respirator Users: Application to Korean Healthcare Workers | 5 | 1 | 3 | 9 |
|  | Seo et al., 2020 [52] | Fit Testing for Domestic N95 Medical Masks | 4 | 1 | 3 | 8 |
|  | Sheikh et al., 2022 [53] | N95 Respirator Fit for a Diverse Population of Healthcare Workers: A Mixed-Methods, Prospective, Pilot and Feasibility Study | 5 | 1 | 2 | 8 |
|  | Sickbert Bennett et al., 2020 [54] | Filtration Efficiency of Hospital Face Mask Alternatives Available for Use During the COVID-19 Pandemic | 3 | 1 | 2 | 6 |
|  | Suen et al., 2022 [55] | Comparing mask fit and usability of traditional and nanofibre N95 filtering facepiece respirators before and after nursing procedures | 5 | 1 | 3 | 9 |
|  | Goh et al., 2022 [56] | A randomised clinical trial to evaluate the safety, fit, comfort of a novel N95 mask in children | 5 | 1 | 3 | 9 |
|  | Salter et al., 2021 [57] | Reinventing Cloth Masks in the Face of Pandemics | 2 | 1 | 2 | 5 |
|  | [Vahabzadeh‐Hagh](https://scholar.google.com/citations?user=JOaLyxoAAAAJ&hl=en&oi=sra) et al., 2022 [58] | Patient-worn endoscopy mask to protect against viral transmission | 3 | 1 | 2 | 6 |
|  | Vo et al., 2020 [59] | A technique to measure respirator protection  factors against aerosol particles in simulated  workplace settings using portable instruments | 3 | 1 | 3 | 7 |
|  | Vuma et al., 2021 [60] | The Effect on Fit of Multiple Consecutive Donning and Doffing of N95 Filtering Facepiece Respirators | 5 | 1 | 3 | 9 |
|  | Williams et al., 2021 [61] | A randomised crossover study to compare the user seal check and quantitative fit test between two types of duckbill N95 particulate respirator masks: The Halyard FluidshieldVR N95 and the BSN Medical ProShieldVR N-95 particulate respirator masks | 5 | 1 | 3 | 9 |
|  | Williams et al., 2021 [62] | Randomized crossover study comparing quantitative  fit tests between Trident_ and 3M_ Aura_ N95/P2  respirators | 5 | 1 | 3 | 9 |
|  | Williams et al., 2022 [63] | Quantitative fit-test concordance of a pair of similar-fit 3M Aura respirator models, 3M 9320A+ and 3M 1870+: A randomized crossover study | 5 | 1 | 3 | 9 |
|  | Williams et al., 2022 [64] | Optimizing twin sampling tube stabilization improves quantitative fit test results for flat-fold duckbill filtering facepiece respirators | 5 | 1 | 3 | 9 |
|  | Lim et al., 2020 [65] | Eﬀects of Particulate Respirator Use on Cardiopulmonary Function in Elderly Women: a Quasi-Experimental Study | 5 | 1 | 3 | 9 |
|  | Mottay et al., 2020 [66]  ‎ | KN95 filtering facepiece respirators distributed in South Africa fail safety testing protocols | 2 | 1 | 3 | 6 |
|  | Zhang et al., 2020 [67] | The relationship between the filtering facepiece respirator fit and the facial anthropometric dimensions | 4 | 1 | 3 | 8 |
|  | Boogaard et al., 2020 [68] | Efficacy assessment of newly-designed and locally-produced  filtering facemasks during the SARS-CoV-2 pandemic | 2 | 2 | 2 | 6 |
|  | Carvalho et al., 2021 [69] | Prospective observational study of gender and ethnicity biases in respiratory protective equipment for healthcare workers in the COVID-19 pandemic | 4 | 1 | 3 | 8 |
|  | Caggiari et al., 2023 [70] | Retrospective evaluation of factors affecting successful fit testing of respiratory protective equipment during the early phase of COVID-19 | 4 | 1 | 3 | 8 |
|  | De‐Yñigo‐Mojado et al., 2021 [71] | Fit factor compliance of masks and FFP3 respirators in nurses: A case–control gender study | 3 | 2 | 3 | 8 |
|  | De-Yñigo-Mojado et al., 2020 [72] | Fit factor of masks used by Physicians in Clinical Settings | 5 | 1 | 3 | 9 |
|  | Green et al., 2021 [73] | Fit-testing of respiratory protective equipment in the UK during the initial response to the COVID-19  pandemic | 3 | 1 | 3 | 7 |
|  | Sun et al., 2020 [74] | Evaluation of a New Workplace Protection Factore Measuring Method for Filtering Facepiece Respirator | 3 | 1 | 3 | 7 |
|  | Vanhooydonck et al., 2021 [75] | Case study into the successful emergency production and certification of a filtering facepiece respirator for Belgian hospitals during the COVID-19 pandemic | 3 | 1 | 2 | 6 |
|  | Winski et al., 2019 [76] | If the mask fits: Facial dimensions and mask performance | 4 | 1 | 3 | 8 |
|  | Chapman et al., 2022 [77] | Quantitative respirator fit tests for P2/N95 in Australian general practice | 3 | 1 | 2 | 6 |
|  | Chen et al., 2022 [78] | Improvement in Fitted Filtration Efficiency of N95  Respirators With Escalating Instruction of the Wearer | 4 | 1 | 3 | 8 |
|  | Clark et al., 2021 [79] | Alteration of Perceptions of Safety Before and After Fit Testing among College of Dentistry Students | 3 | 1 | 3 | 7 |
|  | Inolopú et al., 2023 [80] | Quantitative fit testing on filtering facepiece respirators in use by Peruvian healthcare workers caring for tuberculosis patients during the COVID-19 pandemic: PROFIT study 2020 | 4 | 1 | 3 | 8 |
|  | Low et al., 2021 [81] | Pass rate of the BSN Medical ProShieldVR N95 filtering facepiece using quantitative fit testing in frontline anaesthetists and anaesthesia nurses working on a COVID-19 airway team | 3 | 1 | 3 | 7 |
|  | Ngobeni et al., 2020 [82] | Qualitative Versus Quantitative Fit-Testing of Two Commonly Used Respirators in Resource-Limited Healthcare Facilities | 3 | 2 | 3 | 8 |
|  | Robertsen et al., 2020 [83] | The Effect of a Knowledge-Based Intervention on the Use of Respirators in the Norwegian Smelter Industry | 4 | 1 | 3 | 8 |
|  | Seo et al., 2021 [84] | Fit comparison of Domestic N95 Medical Masks in a Fit test | 4 | 1 | 3 | 8 |
|  | Williams et al., 2021 [85] | Impacts on staff after implementation of a  respiratory protection program in a Victorian public hospital | 4 | 1 | 3 | 8 |
|  | Yeon et al., 2020 [86] | Effects of Education on the Use of Personal  Protective Equipment for Reduction of  Contamination: A Randomized Trial | 4 | 1 | 3 | 8 |
|  | Xiao et al., 2023 [87] | Investigation of a Mask Fitness Test Based on Self-Efficacy and Diversified Training in the Assessment System for Nosocomial Infection Training | 5 | 1 | 3 | 9 |
|  | Anwari et al., 2021 [88] | Development, manufacturing, and  preliminary validation of a reusable half-face respirator during the COVID-19 pandemic | 4 | 1 | 3 | 8 |
|  | Chichester et al., 2020 [89] | Evaluation of an Additively Manufactured Respirator for Personnel Protection from Particulates | 3 | 2 | 2 | 7 |
|  | Fadairo et al., 2020 [90] | Comparison of Condensation Nuclei Counter and Controlled  Negative Pressure Methods under Different Environmental  Conditions Tested with a Mannequin and Human Subjects | 4 | 1 | 3 | 8 |
|  | Hondjeu et al., 2021 [91] | A reusable, locally manufactured, half- face  respirator provides better protection than fit tested  disposable N95 masks: development and quantitative fit-testing comparison | 3 | 1 | 2 | 6 |
|  | Ballard et al., 2021 [92] | Quantitative Fit Tested N95  Respirator-Alternatives Generated With CT Imaging and 3D Printing: A Response to Potential Shortages During the COVID-19 Pandemic | 2 | 1 | 2 | 5 |
|  | Ballard et al., 2021 [93] | Protection levels of N95-level respirator substitutes proposed during the COVID-19 pandemic: safety concerns and quantitative evaluation procedure | 2 | 1 | 2 | 5 |
|  | Duda et al., 2020 [94] | Potential risks of a widespread use of 3D printing for the manufacturing of face masks during the severe acute respiratory syndrome coronavirus 2 pandemic | 2 | 1 | 2 | 5 |
|  | Imbrie-Moore et al., 2020 [95] | Quadrupling the N95 Supply during the COVID-19 Crisis with an Innovative 3D-Printed Mask Adaptor | 2 | 1 | 2 | 5 |
|  | Levine et al., 2022 [96] | Face Off: 3D-Printed Masks as a Cost-Effective and Reusable Alternative to N95 Respirators: A Feasibility Study | 4 | 1 | 3 | 8 |
|  | Liu et al., 2020 [97] | Adapting re-usable elastomeric respirators to utilize anaesthesia circuit filters using a 3D-printed adaptor – a potential alternative to address N95 shortages during the  COVID-19 pandemic | 2 | 1 | 2 | 5 |
|  | Manomaipiboon et al., 2020 [98] | The new silicone N99 half-piece respirator, VJR-NMU N99: A novel and effective tool to prevent COVID-19 SIBATA | 5 | 1 | 2 | 8 |
|  | Martelly et al., 2021 [99] | Moldable Mask: A Reusable, Hot Water Moldable, Additively  Manufactured Mask to Be Used as an N95 Alternative | 2 | 1 | 2 | 5 |
|  | Meadwell et al., 2019 [100] | In Search of a Performing Seal: Rethinking the Design of Tight-Fitting  Respiratory Protective Equipment Facepieces for Users With Facial  Hair | 3 | 1 | 2 | 6 |
|  | McLeod et al., 2021 [101] | An Exploration of Thermomechanical Softwood Pulp for N95 Respiratory Mask Production | 3 | 1 | 2 | 6 |
|  | Ng et al., 2020 [102] | Subject validation of reusable N95 stop-gap filtering facepiece respirators in COVID-19 pandemic | 3 | 1 | 3 | 7 |
|  | Roche et al., 2022 [103] | Personalised 3D printed respirators for healthcare workers during the COVID-19 pandemic | 4 | 1 | 3 | 8 |
|  | Chughtai et al., 2020 [104] | Selection and Use of Respiratory Protection by Healthcare Workers to Protect from Infectious Diseases in Hospital Settings | 3 | 1 | 2 | 6 |
|  | Germonpre et al., 2020 [105] | Evaluation of Protection Level, Respiratory Safety,  and Practical Aspects of Commercially Available  Snorkel Masks as Personal Protection Devices  Against Aerosolized Contaminants and SARS-CoV2 | 2 | 1 | 2 | 5 |
|  | Greig et al., 2020 [106] | Safety testing improvised COVID-19 personal protective equipment based on a modified full-face snorkel mask | 2 | 1 | 2 | 5 |
|  | Greig et al., 2022 [107] | A crossover study assessing the protective efficacy of improvised snorkel-based improvised respirators | 4 | 1 | 3 | 8 |
|  | Grinshpun et al., 2020 [108] | Evaluation of AccuFIT 9000: A Novel Apparatus for Quantitative Fit Testing of Particulate Respirators | 3 | 1 | 3 | 7 |
|  | Harmata et al., 2022 [109] | Rules for fitting filtering gas masks | 3 | 1 | 2 | 6 |
|  | Kechli et al., 2020 [110] | Modifying a Full-Face Snorkel Mask to Meet N95 Respirator Standards for Use With Coronavirus  Disease 2019 Patients | 2 | 1 | 2 | 5 |
|  | Kroo et al., 2021 [111] | Pneumask: Modified Full-Face Snorkel Masks as  Reusable Personal Protective Equipment for Hospital Personnel | 2 | 1 | 2 | 5 |
|  | Nicholson et al., 2021 [112] | Modified full-face snorkel mask as COVID-19 personal protective equipment: Quantitative results | 2 | 1 | 3 | 6 |
|  | Persing et al., 2021 [113] | Comparing respirator laboratory protection factors measured with novel personal instruments to those from the PortaCount | 5 | 1 | 3 | 9 |
|  | Pettinger et al., 2021 [114] | Verification of two Alternative Do-it-yourself  Equipment Respirators Seal as COVID-19 Protection (VADERS-CoV): a quality assessment pilot study | 2 | 2 | 3 | 7 |
|  | Bergman et al., 2019 [115] | Assessment of respirator fit capability test criteria for full-facepiece air-purifying respirators | 4 | 1 | 3 | 8 |
|  | Chehade et al., 2021 [116] | Feasibility study: Proposed alternative to N95 respirator during the personal protective equipment shortage from COVID-19 pandemic | 3 | 1 | 3 | 7 |
|  | Han et al., 2022 [117] | Comparisons of fit factors between two quantitative fit testers (PortaCount® vs MT®) | 5 | 1 | 3 | 9 |
|  | Rengasamy et al., 2021 [118] | Evaluation of total inward leakage for NIOSH-approved elastomeric half-facepiece, fullfacepiece, and powered air-purifying respirators using sodium chloride and corn oil aerosols | 5 | 1 | 3 | 9 |
|  | Sietsema et al., 2022 [119] | Simulated workplace protection factor study of a quarter-facepiece  elastomeric respirator | 5 | 1 | 3 | 9 |
|  | Weng et al., 2022 [120] | A full‑face mask for protection against respiratory infections | 3 | 1 | 3 | 7 |
|  | Clinkard et al., 2021 [121] | Evaluation of N95 respirators, modified snorkel masks and low-cost powered air-purifying respirators: a prospective observational cohort study in healthcare workers | 3 | 1 | 3 | 7 |
|  | Convissar et al., 2020 [122] | Personal Protective Equipment N95 Facemask Shortage Quick Fix: The Modified Airway From VEntilatoR Circuit (MAVerIC) | 2 | 1 | - | 3 |
|  | Toigo et al., 2021 [123] | Fit Testing Retrofitted Full-Face Snorkel Masks  as a Form of Novel Personal Protective Equipment During the COVID-19 Pandemic | 5 | 1 | 2 | 8 |
|  | Cass et al., 2022 [124] | The adequacy of user seal checking for N95 respirators compared to formal fit testing: A multicentred observational study | 5 | 1 | 3 | 9 |
|  | Baba et al., 2022 [125] | Comparison of respiratory protection during exercising tasks between different wearing methods of  replaceable particulate respirators and powered air-purifying respirators | 4 | 1 | 3 | 8 |
|  | Grinshpun et al., 2020 [126] | New respirator performance monitor (RePM) for powered air-purifying respirators | 3 | 1 | 3 | 7 |
|  | Kessel et al., 2022 [127] | An Emergency Powered Air-Purifying Respirator From Local Materials and its Efficacy Against Aerosolized Nanoparticles | 3 | 1 | 2 | 6 |
|  | McGrath et al., 2022 [128] | Bubble-PAPR: Phase I clinical evaluation of an ‘in-house’ developed prototype powered air-purifying  respirator for use by healthcare workers | 5 | 1 | 2 | 8 |
|  | Nagel et al., 2021 [129] | Novel 3D printable powered air purifying respirator for emergency use during PPE shortage of the COVID-19 pandemic: a study protocol and device safety analysis | 2 | 1 | 2 | 5 |
|  | Goto et al., 2021 [130] | The protective effect of tight-fitting powered air-purifying respirators during chest compressions | 5 | 1 | 3 | 9 |
|  | Ng et al., 2023 [131] | HALO CleanSpace PAPR evaluation: Communication, respiratory protection, and usability | 4 | 1 | 3 | 8 |
|  | Rees et al., 2021 [132] | Powered air-purifying respirators: a solution to shortage of FFP3 filtering facepiece respirators in the  operating theatre | 3 | 1 | 2 | 6 |
|  | Sekoguchi et al., 2020 [133] | Evaluation of the performance of replaceable particulate and powered air-purifying respirators considering non-recommended wearing methods | 3 | 1 | 3 | 7 |
|  | Sekoguchi et al., 2022 [134] | Measurement of the workplace protection factor of replaceable particulate and powered air-purifying respirators in Japanese dust-generating occupations | 4 | 1 | 3 | 8 |
|  | Temmesfeld et al., 2022 [135] | Surgical helmets can be converted into efficient disinfectable powered  air-purifying respirators | 3 | 1 | 2 | 6 |
|  | Xu et al., 2023 [136] | Conducting quantitative mask fit tests: application details and affecting factors | 4 | 1 | 3 | 8 |
|  | Rowlett et al., 2021 [137] | The Perceptions Of The Quantitative And Qualitative Fit Testing Protocols And Relevance Of The Clean-Shaven Guidance For The Current Field Of Reusable Elastomeric Half-Mask Respirators | 5 | 1 | 3 | 9 |

**References**

1. Brandel A, Tanner J, Gao J, Kelly N, Snyder A. Testing and Developing DIY Masks. University of Michigan. 2020. Available from: <https://deepblue.lib.umich.edu/handle/2027.42/164436?show=full>.

2. Buckley J, Gladle M, Murray K, Sample W. SUBJECT: Quantitative Respirator Fit Testing of HensMask. University of Delaware. 2020. Available from: <https://me.udel.edu/wp-content/uploads/2020/04/HensNest-FitTest.pdf>.

3. Coyle JP, Derk RC, Lindsley WG, Boots T, Blachere FM, Reynolds JS, et al. Reduction of exposure to simulated respiratory aerosols using ventilation, physical distancing, and universal masking. Indoor Air. 2022;32(2):e12987. doi: 10.1111/ina.12987. PMID: 35225389

4. Dang AJ, Kumfer BM, Bertroche JT, Glidden JO, Oxford CR, Jammalamadaka U, et al. Challenges in predicting the filtration performance of a novel sewn mask: Scale-up from filter holder to mannequin measurements. Aerosol Air Qual Res. 2021;21(6). doi: 10.4209/aaqr.200629.

5. Drouillard KG, Tomkins A, Lackie S, Laengert S, Baker A, Clase CM, et al. Fitted filtration efficiency and breathability of 2-ply cotton masks: Identification of cotton consumer categories acceptable for home-made cloth mask construction. PLoS One. 2022;17(3):e0264090. doi: 10.1371/journal.pone.0264090. PMID: 35316263

6. Duncan S, Bodurtha P, Naqvi S. The protective performance of reusable cloth face masks, disposable procedure masks, KN95 masks and N95 respirators: Filtration and total inward leakage. PLoS One. 2021;16(10):e0258191. doi: 10.1371/journal.pone.0258191. PMID: 34614026

7. Mueller AV, Eden MJ, Oakes JM, Bellini C, Fernandez LA. Quantitative Method for Comparative Assessment of Particle Removal Efficiency of Fabric Masks as Alternatives to Standard Surgical Masks for PPE. Matter. 2020;3(3):950-62. doi: 10.1016/j.matt.2020.07.006. PMID: 32838296

8. Reutman SR, Reponen T, Yermakov M, A. Grinshpun S. Homemade facemasks: particle filtration, breathability, fit, and other performance characteristics. J Occup Environ Hyg. 2021;18(7):334-44. doi: 10.1080/15459624.2021.1925124. PMID: 34080950

9. Teesing GR, van Straten B, de Man P, Horeman-Franse T. Is there an adequate alternative to commercially manufactured face masks? A comparison of various materials and forms. J Hosp Infect. 2020;106(2):246-53. doi: 10.1016/j.jhin.2020.07.024. PMID: 32763333

10. Wentworth F. Advanced Manufacturing Center_Comparative Protective Mask Material Testing. 2020.

11. Lindsley WG, Blachere FM, Law BF, Beezhold DH, Noti JD. Efficacy of face masks, neck gaiters and face shields for reducing the expulsion of simulated cough-generated aerosols. Aerosol Sci Technol. 2021;55(4):449-57. doi: 10.1080/02786826.2020.1862409. PMID: 35924077

12. Sato J, Yamawaki Y, Ito M, Endo M, Tanaka R, Shino M. Measurement of the leak rate of masks used for anticancer drug handling using a mask fitting tester. J Oncol Pharm Pract. 2020;26(6):1318-23. doi: 10.1177/1078155219891210. PMID: 31822202

13. Ardon-Dryer K, Warzywoda J, Tekin R, Biros J, Almodovar S, Weeks BL, et al. Mask Material Filtration Efficiency and Mask Fitting at the Crossroads: Implications during Pandemic Times. Aerosol Air Qual Res. 2021;21(7). doi: 10.4209/aaqr.200571.

14. Bodas CR, Ng I, Kave B, Begg F, Williams DL. A randomised crossover trial of two flat-fold cup respirators: BYD DE2322 N95 versus Care Essentials MSK-002 P2. Infect Dis Health. 2022. doi: 10.1016/j.idh.2022.08.002. PMID: 36207250

15. Cameron S, Cheung W, Cronin N, Griffiths K, Hunt R, Innes L, et al. Quantitative fit testing with limited supplies of respirator masks in hospital personnel during the COVID-19 pandemic. Aust Health Rev. 2020;44(4):542-3. doi: 10.1071/ah20154. PMID: 32718421

16. Chan JK, Yep KH, Magarey S, Keon-Cohen Z, Acheson M. Fit Testing Disposable P2/N95 Respirators during COVID-19 in Victoria, Australia: Fit Check Evaluation, Failure Rates, and a Survey of Healthcare Workers. COVID. 2021;1(1):83-96. doi: 10.3390/covid1010007.

17. Christopher L, Rohr-Kirchgraber T, Mark S. The PPE Pandemic: Sex-Related Discrepancies of N95 Mask Fit. EMJ Microbiol Infect Dis.2(1):57-63. doi: 10.33590/emjmicrobiolinfectdis/20-00215.

18. Cloet A, Yu M, Arnold J, Griffin L. Activity and Usability Evaluation of Alternative Protective Face Mask Designs. Proc Hum Factors Ergon Soc Annu Meet. 2022;66(1):1682-6. doi: 10.1177/1071181322661162.

19. Cloet A, Griffin L, Yu M, Durfee W. Design considerations for protective mask development: A remote mask usability evaluation. Appl Ergon. 2022;102:103751. doi: 10.1016/j.apergo.2022.103751. PMID: 35339761

20. Griffin L, Yu MJ, Cloet A, Arnold S, Carlson N, Hillmyer M, et al. Protective Masks Utilizing Nonendangered Components. J Med Device. 2022;16(1). doi: 10.1115/1.4053720. PMID: 35280214

21. Duncan S, Bodurtha P, Bourgeois C, Dickson E, Jensen C, Naqvi S. The impact of extreme reuse and extended wear conditions on protection provided by a surgical-style N95 filtering facepiece respirator. J Occup Environ Hyg. 2020;17(11-12):546-59. doi: 10.1080/15459624.2020.1829633. PMID: 33166226

22. Fabre V, Cosgrove SE, Hsu YJ, Jones GF, Helsel T, Bukowski J, et al. N95 filtering face piece respirators remain effective after extensive reuse during the coronavirus disease 2019 (COVID-19) pandemic. Infect Control Hosp Epidemiol. 2021;42(7):896-9. doi: 10.1017/ice.2021.76. PMID: 33602376

23. Nakamoto K, SARAYA Sr T, Kurai D, Fukukawa N, Taneoka T, Shimasaki T, et al. Reusing N95 Respirators at Weekly Intervals During the COVID-19 Pandemic. Cureus. 2021;13(2).

24. Greenawald LA, Moore SM, Yorio PL. PPE CASE: Evaluation of Fit and Strap Extension Performance of Stockpiled Filtering Facepiece Respirators from One U.S. Facility. By Greenawald LA, Moore SM, and Yorio PL. Pittsburgh, PA: U.S. Department of Health and Human Services, Centers for Disease Control and Prevention, National Institute for Occupational Safety and Health, NPPTL Report Number P2021-0102. 2021.

25. Hai CH, Hua KK, Fu GQ, Singh K, Wah YC. Comparing Quality of Fitting of Different Filtering Face Pieces in a Healthcare Worker: A preliminary observational study. J Posit School Psychol. 2022;6(3):4012–8.

26. Han S-H, Cha K-S, Yoo S-Y, Han JO. Comparison of N95 Respiratory Mask Fit Testing. Korean J Healthc Assoc Infect Control 2021;26(2):108-14. doi: 10.14192/kjicp.2021.26.2.108.

27. Hwang SY, Yoon H, Yoon A, Kim T, Lee G, Jung KY, et al. N95 filtering facepiece respirators do not reliably afford respiratory protection during chest compression: A simulation study. Am J Emerg Med. 2020;38(1):12-7. doi: 10.1016/j.ajem.2019.03.041. PMID: 30955924

28. Fakherpour A, Jahangiri M, Seif M, Charkhand H, Abbaspour S, Floyd EL. Quantitative fit testing of filtering face-piece respirators during the COVID-19 pandemic reveals anthropometric deficits in most respirators available in Iran. J Environ Health Sci Eng. 2021;19(1):805-17. doi: 10.1007/s40201-021-00648-3. PMID: 33875931

29. Jankusol K, Chaiear N, Mitsungnern T. Different fit factors for the N95 respirator during endotracheal intubation: Comparing video laryngoscopy and direct laryngoscopy. Asia Pac J Sci Technol. 2023;28(2):APST-28-02-12. doi: 10.14456/apst.2023.28.

30. Joshi M, Khan A, Sapra BK. Quick laboratory methodology for determining the particle filtration efficiency of face masks/respirators in the wake of COVID-19 pandemic. J Ind Text.51(5S

):7622S-40S. doi: 10.1177/1528083720975084.

31. Jean-Romain D, David V, Guillaume S, de Damien C, Walter Z, Vincent P, et al. Distribution of low quality filtering facepiece respirators during the COVID-19 pandemic: An independent analysis of the situation in Switzerland. Swiss Med Wkly. 2021;151(3). doi: 10.4414/smw.2021.20459. PMID: 33516162

32. Jung J, Kim J, Yang H, Lim Y-J, Kwak S-H, Hong MJ, et al. Fit-failure rate associated with simulated reuse and extended use of N95 respirators assessed by a quantitative fit test. Infect Control Hosp Epidemiol. 2021;42(11):1313-7. doi: 10.1017/ice.2021.5. PMID: 33487185

33. Kamal M, Bhatti M, Stewart WC, Johns M, Collins D, Shehabi Y, et al. Safety Goggles with Elastic Headband to Improve N95 Fit Following Failed Quantitative Fit Test. Indian J Crit Care Med

2023;27(6):386. doi: 10.5005/jpjournals-10071-24473. PMID: 37378367

34. Kyaw S, Johns M, Lim R, Stewart WC, Rojas N, Thambiraj SR, et al. Prediction of N95 Respirator Fit from Fogging of Eyeglasses: A Pilot Study. Indian J Crit Care Med. 2021;25(9):976-80. doi: 10.5005/jp-journals-10071-23947. PMID: 34963713

35. Landry SA, Subedi D, Barr JJ, MacDonald MI, Dix S, Kutey DM, et al. Fit-tested N95 masks combined with portable HEPA filtration can protect against high aerosolized viral loads over prolonged periods at close range. J Infect Dis. 2022; 226(2):199-207. doi: 10.1093/infdis/jiac195. PMID: 35535021

36. Lindsley WG, Blachere FM, Beezhold DH, Law BF, Derk RC, Hettick JM, et al. A comparison of performance metrics for cloth masks as source control devices for simulated cough and exhalation aerosols. Aerosol Sci Technol. 2021;55(10):1125-42. doi: 10.1080/02786826.2021.1933377. PMID: 35923216

37. Long KD, Woodburn EV, Berg IC, Chen V, Scott WS. Measurement of filtration efficiencies of healthcare and consumer materials using modified respirator fit tester setup. PLoS One. 2020;15(10). doi: 10.1371/journal.pone.0240499. PMID: 33048980

38. Milosevic M, Biswas RK, Innes L, Ng M, Darendeliler AM, Wong A, et al. P2/N95 filtering facepiece respirators: Results of a large-scale quantitative mask fit testing program in Australian health care workers. Am J Infect Control. 2022;50(5). doi: 10.1016/j.ajic.2021.12.016. PMID: 34971710

39. Ng I, Kave B, Begg F, Bodas CR, Segal R, Williams D. N95 respirators: quantitative fit test pass rates and usability and comfort assessment by health care workers. Med J Aust. 2022;217(2):88-93. doi: 10.5694/mja2.51585. PMID: 35645035

40. O’Kelly E, Arora A, Ward J, Clarkson PJ. How well do face masks protect the wearer compared to public perceptions? medRxiv [Preprint] 2021 [Posted 2021 January 31]. [6 p.]. Available from: <https://www.medrxiv.org/content/10.1101/2021.01.27.21250645v1.full-text>. doi: 10.1101/2021.01.27.21250645.

41. O'Kelly E, Arora A, Pirog S, Ward J, Clarkson PJ. Comparing the fit of N95, KN95, surgical, and cloth face masks and assessing the accuracy of fit checking. PLoS One. 2021;16(1). doi: 10.1371/journal.pone.0245688. PMID: 33481870

42. O’Kelly E, Arora A, Pirog S, Ward J, Clarkson PJ. Experimental Measurement of the Size of Gaps Required to Compromise Fit of an N95 Respirator. Disaster Med Public Health Prep. 2022;17:1-13. doi: 10.1017/dmp.2022.23. PMID: 35057880

43. Park JJ, Seo YB, Lee J. Fit Test for N95 Filtering Facepiece Respirators and KF94 Masks for Healthcare Workers: a Prospective Single-center Simulation Study. J Korean Med Sci. 2021;36(21):e140. doi: 10.3346/jkms.2021.36.e140. PMID: 34060256

44. Popov T, Popov G, Basse A. Development and Application of a Modified Procedure for Quantitative Fit Testing of Disposable Masks and Respirators. J Occup Environ Hyg. 2022;19(5):266-70. doi: 10.1080/15459624.2022.2050741. PMID: 35259072

45. Regli A, Sommerfield A, Thalayasingam P, von Ungern-Sternberg BS. N95 Masks to Protect Health Care Workers: Is the New Fast Fit-Test Protocol Cutting Corners? Chest. 2022;161(6):1606-8. doi: 10.1016/j.chest.2022.01.048. PMID: 35131299

46. Regli A, Thalayasingam P, Bell E, Sommerfield A, von Ungern-Sternberg BS. More than half of front-line healthcare workers unknowingly used an N95/P2 mask without adequate airborne protection: An audit in a tertiary institution. Anaesth Intensive Care. 2021;49(5):404-11. doi: 10.1177/0310057X211007861. PMID: 34325537

47. Prince SE, Chen H, Tong H, Berntsen J, Masood S, Zeman KL, et al. Assessing the effect of beard hair lengths on face masks used as personal protective equipment during the COVID-19 pandemic. J Expo Sci Environ Epidemiol. 2021;31(6):953-60. doi: 10.1038/s41370-021-00337-1. PMID: 34006963

48. Sandaradura I, Goeman E, Pontivivo G, Fine E, Gray H, Kerr S, et al. A close shave? Performance of P2/N95 respirators in healthcare workers with facial hair: results of the BEARDS (BEnchmarking Adequate Respiratory DefenceS) study. J Hosp Infect. 2020;104(4):529-33. doi: 10.1016/j.jhin.2020.01.006. PMID: 31978416

49. De-Yñigo-Mojado B, Becerro-de-Bengoa-Vallejo R, Losa-Iglesias ME, Madera-García J, Rodríguez-Sanz D, Calvo-Lobo C, et al. Facial Hair Decreases Fit Factor of Masks and Respirators in Healthcare Providers. Biology (Basel). 2021;10(10). doi: 10.3390/biology10101031. PMID: 34681128

50. Sasko LM, Oliver B, Smith SM. Fit testing of masks worn by frontline healthcare workers. Infect Control Hosp Epidemiol. 2023:1-2. doi: 10.1017/ice.2022.268. PMID: 36594247

51. Seo H, Myong J-P, Kang B-k, Kwon Y-i. Necessity of the Fit Test Panel for Korean Respirator Users: Application to Korean Healthcare Workers. J Int Soc Respir Prot. 2021;38(2):1-11.

52. Seo H, Kang B-k, Kwon Y-i. Fit testing for domestic N95 medical masks. J Korean Soc Occup Environ Hyg. 2020;30(2):124-33. doi: 10.15269/JKSOEH.2020.30.2.124.

53. Sheikh F. N95 Respirators for a Diverse Population of Healthcare Workers: A Mixed-Methods, Pilot and Feasibility Study. M.Sc. Thesis, McMaster University. 2022. Available from: <https://macsphere.mcmaster.ca/handle/11375/28032>.

54. Sickbert-Bennett EE, Samet JM, Clapp PW, Chen H, Berntsen J, Zeman KL, et al. Filtration Efficiency of Hospital Face Mask Alternatives Available for Use During the COVID-19 Pandemic. JAMA Intern Med. 2020;180(12):1607-12. doi: 10.1001/jamainternmed.2020.4221. PMID: 32780113

55. Suen LKP, Guo YP, Ho SSK, Au-Yeung CH, Lam SC. Comparing mask fit and usability of traditional and nanofibre N95 filtering facepiece respirators before and after nursing procedures. J Hosp Infect. 2020;104(3):336-43. doi: 10.1016/j.jhin.2019.09.014. PMID: 31545991

56. Goh DYT, Mun MW, Lee WLJ, Teoh OH, Rajgor DD. A randomised clinical trial to evaluate the safety, fit, comfort of a novel N95 mask in children. Sci Rep. 2019;9(1):18952. doi: 10.1038/s41598-019-55451-w. PMID: 31831801

57. Salter SJRA. Reinventing cloth masks in the face of pandemics. 2021;41(5):731-44.

58. Vahabzadeh‐Hagh AM, Patel SH, Stramiello JA, Weissbrod PA. Patient‐worn endoscopy mask to protect against viral transmission. Laryngoscope Investig Otolaryngol. 2022;7(1):190-6. doi: 10.1002%2Flio2.708. PMID: 35155797

59. Vo E, Horvatin M, Bergman M, Wu B, Zhuang Z. A technique to measure respirator protection factors against aerosol particles in simulated workplace settings using portable instruments. J Occup Environ Hyg. 2020;17(5):231-42. doi: 10.1080/15459624.2020.1735640. PMID: 32243774

60. Vuma CD, Manganyi J, Wilson K, Rees D. The effect on fit of multiple consecutive donning and doffing of N95 filtering facepiece respirators. Ann Work Expo Health. 2019;63(8):930-6. doi: 10.1093/annweh/wxz060. PMID: 31504129

61. Williams DL, Kave B, Lee K, Segal R, Krieser RB, Mezzavia PM, et al. A randomised crossover study to compare the user seal check and quantitative fit test between two types of duckbill N95 particulate respirator masks: The Halyard Fluidshield (R) N95 and the BSN Medical ProShield (R) N-95 particulate respirator masks. Anaesth Intensive Care. 2021;49(2):112-8. doi: 10.1177/0310057X20974022. PMID: 33818131

62. Williams DL, Kave B, Begg F, Bodas C, Ng I. Randomized crossover study comparing quantitative fit tests between Trident (TM) and 3M (TM) Aura (TM) N95/P2 respirators. Infect Dis Health. 2022;27(2):61-5. doi: 10.1016/j.idh.2021.10.002. PMID: 34799300

63. Williams DL, Kave B, Begg F, Bodas C, Ng I. Quantitative fit-test concordance of a pair of similar-fit 3M Aura respirator models, 3M 9320A+ and 3M 1870+: A randomized crossover study. Infect Control Hosp Epidemiol. 2022;44(2):1-4. doi: 10.1017/ice.2022.67. PMID: 35387701

64. Williams DL, Kave B, Bodas C, Begg F, Roberts M, Ng I. Optimizing twin sampling tube stabilization improves quantitative fit test results for flat-fold duckbill filtering facepiece respirators. Am J Infect Control. 2022;51(6):694-8. doi: 10.1016/j.ajic.2022.09.026. PMID: 36216035

65. Lim Y-H, Kim W, Choi Y, Kim H-C, Na G, Kim H-R, et al. Effects of Particulate Respirator Use on Cardiopulmonary Function in Elderly Women: a Quasi-Experimental Study. J Korean Med Sci. 2020;35(10). doi: 10.3346/jkms.2020.35.e64. PMID: 32174063

66. Mottay L, Le Roux J, Perumal R, Esmail A, Timm L, Sivarasu S, et al. KN95 filtering facepiece respirators distributed in South Africa fail safety testing protocols. S Afr Med J. 2020;111(3):13162. doi: 10.7196/samj.2021.v111i3.15381. PMID: 33334390

67. Zhang X, Jia N, Wang Z. The relationship between the filtering facepiece respirator fit and the facial anthropometric dimensions among Chinese people. Ind Health. 2020;58(4):318-24. doi: 10.2486/indhealth.2019-0158. PMID: 31787708

68. Boogaard B, Tas A, Nijssen J, Broeren F, van den Dobbelsteen J, Verhoeven V, et al. Efficacy Assessment of Newly-designed Filtering Facemasks during the SARS-CoV-2 Pandemic. Aerosol Air Qual Res. 2021;21(3):200424. doi: 10.4209/aaqr.2020.07.0424.

69. Carvalho CYM, Schumacher J, Greig PR, Wong DJN, El-Boghdadly K. Prospective observational study of gender and ethnicity biases in respiratory protective equipment for healthcare workers in the COVID-19 pandemic. BMJ Open. 2021;11(5):e047716. doi: 10.1136/bmjopen-2020-047716. PMID: 34016664

70. Caggiari S, Bader D, Packman Z, Robinson J, Tranka S, Böhning D, et al. Retrospective evaluation of factors affecting successful fit testing of respiratory protective equipment during the early phase of COVID-19. BMJ Open. 2023;13(5):e065068. doi: 10.1136/bmjopen-2022-065068. PMID: 37230519

71. De‐Yñigo‐Mojado B, Madera‐García J, Becerro‐De‐Bengoa‐Vallejo R, Losa‐Iglesias ME, Rodríguez‐Sanz D, Calvo‐Lobo C, et al. Fit factor compliance of masks and FFP3 respirators in nurses: A case–control gender study. J Adv Nurs. 2021. doi: 10.1111/jan.14823. PMID: 33733471

72. De-Yñigo-Mojado B, Madera-García J, Becerro-de-Bengoa-Vallejo R, Losa-Iglesias ME, Rodríguez-Sanz D, San-Antolín M, et al. Fit factor of masks used by Physicians in Clinical Settings. Int J Med Sci. 2020;17(17):2696-702. doi: 10.7150/ijms.50657. PMID: 33162797

73. Green S, Gani A, Bailey M, Brown O, Hing CB. Fit-testing of respiratory protective equipment in the UK during the initial response to the COVID-19 pandemic. J Hosp Infect. 2021;113:180-6. doi: 10.1016/j.jhin.2021.04.024. PMID: 33940089

74. Sun C, Thelen C, Sanz IS, Wittmann A. Evaluation of a new workplace protection factor–measuring method for filtering facepiece respirator. Saf Health Work. 2020;11(1):61-70. doi: 10.1016/j.shaw.2019.11.001. PMID: 32206375

75. Vanhooydonck A, Van Goethem S, Van Loon J, Vandormael R, Vleugels J, Peeters T, et al. Case study into the successful emergency production and certification of a filtering facepiece respirator for Belgian hospitals during the COVID-19 pandemic. J Manuf Syst. 2021;60:876-92. doi: 10.1016/j.jmsy.2021.03.016. PMID: 33814674

76. Winski TA, Mueller WA, Graveling RA. If the mask fits: facial dimensions and mask performance. Int J Ind Ergon. 2019;72:308-10. doi: 10.1016/j.ergon.2019.05.011.

77. Chapman D, Chapman L, Ganesan A. Quantitative respirator fit tests for P2/N95 in Australian general practice. Aust J Gen Pract. 2022;51. doi: 10.31128/ajgp-covid-51-1. PMID: 35172324

78. Chen H, Pennington ER, Case MW, Tong H, Rappold AG, Samet JM, et al. Improvement in Fitted Filtration Efficiency of N95 Respirators With Escalating Instruction of the Wearer. AJPM Focus. 2022;1(1):100014. doi: 10.1016/j.focus.2022.100014. PMID: 36338466

79. Clark TH. Alteration of Perceptions of Safety Before and After Fit Testing among College of Dentistry Students. MSc. degree, University of Nebraska Medical Center. 2021. Available from: <https://digitalcommons.unmc.edu/cgi/viewcontent.cgi?article=1136&context=coph_slce>.

80. Inolopú J, Mayma K, Curisinche-Rojas M, Aylas R, Flores JA, Rosales J. Quantitative Fit Testing on Filtering Facepiece Respirators in Use by Peruvian Healthcare Workers Caring for Tuberculosis Patients During the COVID-19 Pandemic: PROFIT Study 2020. 2023. doi: 10.3390/ijerph20166618. PMID: 37623201

81. Low CS, Weinberg L, Ellard LM, Hacking DF, Banyasz D. Pass rate of the BSN Medical ProShield® N95 filtering facepiece using quantitative fit testing in frontline anaesthetists and anaesthesia nurses working on a COVID-19 airway team. Anaesth Intensive Care. 2021;49(4):322-3. doi: 10.1177/0310057x21997150. PMID: 34039048

82. Ngobeni K. Qualitative Versus Quantitative Fit-Testing of Two Commonly Used Respirators in Resource-Limited Healthcare Facilities: University of Johannesburg (South Africa); 2020.

83. Robertsen Ø, Hegseth MN, Føreland S, Siebler F, Eisemann M, Vangberg HCB. The Effect of a Knowledge-Based Intervention on the Use of Respirators in the Norwegian Smelter Industry. Front Psychol. 2020;11:270. doi: 10.3389%2Ffpsyg.2020.00270. PMID: 32153476

84. Seo H, Kwon Y-i, Myong J-P, Kang B-k. Fit comparison of Domestic N95 Medical Masks in a Fit test. J Korean Soc Occup Environ Hyg. 2021;31(1):94-104. doi: 10.15269/JKSOEH.2021.31.1.94.

85. Williams D, Kave B, Begg F, Marshall C, Segal R, Ng I. Impacts on staff after implementation of a respiratory protection program in a Victorian public hospital. Infect Dis Health. 2021;26(4):265-72. doi: 10.1016/j.idh.2021.06.001. PMID: 34176771

86. Yeon JH, Shin YS. Effects of education on the use of personal protective equipment for reduction of contamination: a randomized trial. SAGE Open Nurs. 2020;6:2377960820940621. doi: 10.1177/2377960820940621. PMID: 33415295

87. Xiao B, Sun L-L, Yuan J, Xiao W-L, Liu Y, Cai M-Y, et al. Investigation of a Mask Fitness Test Based on Self-Efficacy and Diversified Training in the Assessment System for Nosocomial Infection Training. Infect Drug Resist. 2023;16:313-22. doi: 10.2147/idr.s388784. PMID: 36691491

88. Anwari V, Ng WCK, Hondjeu ARM, Xiao ZX, Afenu E, Trac J, et al. Development, manufacturing, and preliminary validation of a reusable half-face respirator during the COVID-19 pandemic. PLoS One. 2021;16(3). doi: 10.1371/journal.pone.0247575. PMID: 33730106

89. Chichester DL, Hix JD, Johnson JT, Ocampo Giraldo LA, Watson SM, Mortensen BT, et al. Evaluation of an Additively Manufactured Respirator for Personnel Protection from Particulates. Idaho National Lab.(INL), Idaho Falls, ID (United States); 2020.

90. Fadairo OJ. Comparison of Condensation Nuclei Counter and Controlled Negative Pressure Methods under Different Environmental Conditions Tested with a Mannequin and Human Subjects. Doctoral dissertation, West Virginia University. 2020. Available from: <https://researchrepository.wvu.edu/etd/7986/>. doi: 10.33915/etd.7986.

91. Hondjeu ARM, Ng WC, Anwari V, Xiao MZ, Rozenberg D, Kazlovich K, et al. A reusable, locally manufactured, half-face respirator provides better protection than fitted disposable N95 masks: development and quantitative fit-testing comparison. Research Square [Preprint] 2021 [cited 2023 July 9] Available from: <https://wwwresearchgatenet/publication/351605094_A_reusable_locally_manufactured_half-_face_respirator_provides_better_protection_than_fitted_disposable_N95_masks_development_and_quantitative_fit-testing_comparison>. doi: 10.21203/rs.3.rs-456096/v1.

92. Ballard DH, Jammalamadaka U, Meacham KW, Hoegger MJ, Burke BA, Morris JA, et al. Quantitative Fit Tested N95 Respirator-Alternatives Generated With CT Imaging and 3D Printing: A Response to Potential Shortages During the COVID-19 Pandemic. Acad Radiol. 2021;28(2):158-65. doi: 10.1016%2Fj.acra.2020.11.005. PMID: 33257256

93. Ballard DH, Dang AJ, Kumfer BM, Weisensee PB, Meacham JM, Scott AR, et al. Protection levels of N95-level respirator substitutes proposed during the COVID-19 pandemic: safety concerns and quantitative evaluation procedures. BMJ Open. 2021;11(9):e045557. doi: 10.1136/bmjopen-2020-045557. PMID: 34475144

94. Duda S, Hartig S, Hagner K, Meyer L, Intriago PW, Meyer T, et al. Potential risks of a widespread use of 3D printing for the manufacturing of face masks during the severe acute respiratory syndrome coronavirus 2 pandemic. J 3D Print Med. 2020;4(3):135-47. doi: 10.2217%2F3dp-2020-0014.

95. Imbrie-Moore AM, Park MH, Zhu Y, Paulsen MJ, Wang H, Woo YJ. Quadrupling the N95 Supply during the COVID-19 Crisis with an Innovative 3D-Printed Mask Adaptor. Healthcare (Basel). 2020;8(3). doi: 10.3390/healthcare8030225. PMID: 32717841

96. Levine M, Levine L, Xun H, Mathew PJ, Singh D, Gerber A, et al. Face Off: 3D Printed Masks as a Cost-Effective and Reusable Alternative to N95 Respirators: A Feasibility Study. Am J Med. 2022;135(9):1109-15. doi: 10.1016/j.amjmed.2022.04.026. PMID: 35580720

97. Liu DCY, Koo TH, Wong JKK, Wong YH, Fung KSC, Chan Y, et al. Adapting re-usable elastomeric respirators to utilise anaesthesia circuit filters using a 3D-printed adaptor - a potential alternative to address N95 shortages during the COVID-19 pandemic. Anesthesia. 2020;75(8):1022-7. doi: 10.1111/anae.15108. PMID: 32348561

98. Manomaipiboon A, Pupipatpab S, Chomdee P, Boonyapatkul P, Trakarnvanich T. The new silicone N99 half-piece respirator, VJR-NMU N99: A novel and effective tool to prevent COVID-19. PLoS One. 2020;15(12). doi: 10.1371/journal.pone.0237206. PMID: 33382705

99. Martelly E, Li C, Shimada K. Moldable Mask: A Reusable, Hot Water Moldable, Additively Manufactured Mask to Be Used as an N95 Alternative. Materials (Basel). 2021;14(22). doi: 10.3390/ma14227082. PMID: 34832483

100. Meadwell J, Paxman-Clarke L, Terris D, Ford P. In search of a performing seal: Rethinking the design of tight-fitting respiratory protective equipment facepieces for users with facial hair. Saf Health Work. 2019;10(3):275-304. doi: 10.1016/j.shaw.2019.05.001. PMID: 31497325

101. McLeod KER. An exploration of thermomechanical softwood pulp for N95 respiratory mask production. BSc. Thesis, Saint Mary’s University, Halifax, Nova Scotia. 2021. Available from: <https://library2.smu.ca/handle/01/29526?show=full>.

102. Ng WCK, Mbadjeu Hondjeu AR, Syrett A, Caragata R, Rozenberg D, Xiao Z, et al. Subject validation of reusable N95 stop-gap filtering facepiece respirators in COVID-19 pandemic. PLoS One. 2020;15(11):e0242304. doi: 10.1371/journal.pone.0242304. PMID: 33186406

103. Roche AD, McConnell AC, Donaldson K, Lawson A, Tan S, Toft K, et al. Personalised 3D printed respirators for healthcare workers during the COVID-19 pandemic. Front Med Technol. 2022;4:45. doi: 10.3389/fmedt.2022.963541. PMID: 35982716

104. Chughtai AA, Seale H, Rawlinson WD, Kunasekaran M, Macintyre CR. Selection and use of respiratory protection by healthcare workers to protect from infectious diseases in hospital settings. Ann Work Expo Health. 2020;64(4):368-77. doi: 10.1093/annweh/wxaa020c. PMID: 32144412

105. Germonpre P, Van Rompaey D, Balestra C. Evaluation of Protection Level, Respiratory Safety, and Practical Aspects of Commercially Available Snorkel Masks as Personal Protection Devices Against Aerosolized Contaminants and SARS-CoV2. Int J Environ Res Public Health. 2020;17(12). doi: 10.3390%2Fijerph17124347. PMID: 32575366

106. Greig P, Carvalho C, El‐Boghdadly K, Ramessur S. Safety testing improvised COVID‐19 personal protective equipment based on a modified full‐face snorkel mask. Anaesthesia. 2020;75(7):970-1. doi: 10.1111/anae.15085. PMID: 32275770

107. Greig PR, Bradshaw J, Carvalho C, Iwaszko L, Ramessur S, Schumacher J, et al. A crossover study assessing the protective efficacy of improvised snorkel-based improvised respirators. J Intensive Care Soc. 2022;23(3):359-61. doi: 10.1177/1751143721991056. PMID: 36033251

108. Grinshpun SA, Yermakov M, Kano M. Evaluation of AccuFIT 9000: A Novel Apparatus for Quantitative Fit Testing of Particulate Respirators. Ann Work Expo Health. 2020;65(4):458-62. doi: 10.1093/annweh/wxaa116. PMID: 33345279

109. Harmata W, Kamionek D. Rules for fitting filtering gas masks. Sci J Mil Univ Land Forces. 2022;54(2 ):179-95. doi: 10.5604/01.3001.0015.8971.

110. Kechli MK, Lerman J, Ross MM. Modifying a Full-Face Snorkel Mask to Meet N95 Respirator Standards for Use With Coronavirus Disease 2019 Patients. AA Pract. 2020;14(7):e01237. doi: 10.12132FXAA.0000000000001237. PMID: 32539273

111. Kroo L, Kothari A, Hannebelle M, Herring G, Pollina T, Chang R, et al. Modified full-face snorkel masks as reusable personal protective equipment for hospital personnel. PLoS One. 2021;16(1). doi: 10.1371/journal.pone.0244422. PMID: 33439902

112. Nicholson K, Henke-Adams A, Henke DM, Kravitz AV, Gay HA. Modified full-face snorkel mask as COVID-19 personal protective equipment: Quantitative results. HardwareX. 2021;9:e00185. doi: 10.1016/j.ohx.2021.e00185. PMID: 33655089

113. Persing AJ, Sietsema M, Farmer K, Peters TM. Comparing respirator laboratory protection factors measured with novel personal instruments to those from the PortaCount. J Occup Environ Hyg. 2021;18(2):65-71. doi: 10.1080/15459624.2020.1864152. PMID: 33406010

114. Pettinger M, Momeni M, Michaud C, Van Dyck M, Kahn D, Lemaire G. Verification of two Alternative Do-it-yourself Equipment Respirators Seal as COVID-19 Protection (VADERS-CoV): a quality assessment pilot study. Acta Anaesth Belg. 2020;72(2):101-7. doi: 10.56126/72.2.7.

115. Bergman MS, Zhuang Z, Xu SS, Rengasamy S, Lawrence RB, Boutin B, et al. Assessment of respirator fit capability test criteria for full-facepiece air-purifying respirators. J Occup Environ Hyg. 2019;16(7):489-97. doi: 10.1080/15459624.2019.1609006. PMID: 31107187

116. Chehade AEH, Stephenson J, Floyd E, Keddissi J, Abdo T, Thind S, et al. Feasibility study: Proposed alternative to N95 respirator during the personal protective equipment shortage from COVID-19 pandemic. J Emerg Manag. 2021;19(7):193-202. doi: 10.5055/jem.0611. PMID: 34723379

117. Han D-H, Seo H, Kang B-k, Jang H, Kim H, Shim S. Comparisons of Fit Factors Between Two Quantitative Fit Testers (PortaCount vs. MT). Saf Health Work. 2022;13(4):500-6. doi: 10.1016/j.shaw.2022.10.001. PMID: 36579005

118. Rengasamy S, Zhuang Z, Lawrence RB, Boutin B, Yorio P, Horvatin M, et al. Evaluation of total inward leakage for NIOSH-approved elastomeric half-facepiece, full-facepiece, and powered air-purifying respirators using sodium chloride and corn oil aerosols. J Occup Environ Hyg. 2021;18(7):305-13. doi: 10.1080/15459624.2021.1919685. PMID: 34038318

119. Sietsema M, Hamza H, Brosseau LMJJoO, Hygiene E. Simulated workplace protection factor study of a quarter facepiece elastomeric respirator. J Occup Environ Hyg. 2022;20(1):33-9. <https://doi.org/10.1080/15459624.2022.2145014>. PMID: 36416662

120. Weng C-H, Kao C-L, Chiu P-W, Huang S-P, Kuo Y-S, Lin Y-Y, et al. A full-face mask for protection against respiratory infections. BioMedical Engineering OnLine. 2022;21(1):62. 10.1186/s12938-022-01027-1.

121. Clinkard D, Mashari A, Karkouti K, Fedorko L. Evaluation of N95 respirators, modified snorkel masks and low-cost powered air-purifying respirators: a prospective observational cohort study in healthcare workers. Anaesthesia. 2021. doi: 10.1111/anae.15392. PMID: 33470422

122. Convissar D, Berra L, Chang MG, Bittner EA. Personal Protective Equipment N95 Facemask Shortage Quick Fix: The Modified Airway From VEntilatoR Circuit (MAVerIC). Cureus. 2020;12(5). doi: 10.7759/cureus.7914. PMID: 32440384

123. Toigo S, Jacques M, Razek T, Rajda E, Omelon S, Dankoff F, et al. Fit Testing Retrofitted Full-Face Snorkel Masks as a Form of Novel Personal Protective Equipment During the COVID-19 Pandemic. Disaster Med Public Health Prep. 2021:1-16. doi: 10.1017/dmp.2021.133. PMID: 33926606

124. Cass HG, Hanlon GC, McKenzie DP, Harley NS, Kelly DN, Barrett JA. The adequacy of user seal checking for N95 respirators compared to formal fit testing: A multicentred observational study. Aust Crit Care. 2022. doi: 10.1016/j.aucc.2022.08.012. PMID: 36244917

125. Baba H, Ando H, Ikegami K, Sekoguchi S, Shirasaka T, Ogami A. Comparison of respiratory protection during exercise tasks between different methods of wearing replaceable particulate respirators and powered air-purifying respirators. Ind Health. 2022:2021-0268. doi: 10.2486/indhealth.2021-0268. PMID: 35569997

126. Grinshpun SA, Corey J, Yermakov M, Wu B, Strickland KT, Bergman M, et al. New respirator performance monitor (RePM) for powered air-purifying respirators. J Occup Environ Hyg. 2020;17(11-12):538-45. doi: 10.1080/15459624.2020.1814491. PMID: 32941118

127. Kessel J, Saevig CS, Hill WC, Kessel B, Hull MS. An Emergency Powered Air-Purifying Respirator From Local Materials and its Efficacy Against Aerosolized Nanoparticles. Inquiry. 2022;59:469580221087837. doi: 10.1177/00469580221087837. PMID: 35341353

128. McGrath BA, Shelton CL, Gardner A, Coleman R, Lynch J, Alexander PG, et al. Bubble-PAPR: a phase 1 clinical evaluation of the comfort and perception of a prototype powered air-purifying respirator for use by healthcare workers in an acute hospital setting. BMJ Open. 2023;13(5):e066524. doi: 10.1136/bmjopen-2022-066524. PMID: 37156585

129. Nagel J, Gilbert C, Duchesne J. Novel 3D printable powered air purifying respirator for emergency use during PPE shortage of the COVID-19 pandemic: a study protocol and device safety analysis. BMJ Open. 2021;11(8):e049605. doi: 10.1136/bmjopen-2021-049605. PMID: 34446492

130. Goto Y, Jingushi N, Hiraiwa H, Ogawa H, Sakai Y, Kasugai D, et al. The protective effect of tight-fitting powered air-purifying respirators during chest compressions. Am J Emerg Med. 2021;49:172-7. doi: 10.1016/j.ajem.2021.06.012. PMID: 34118785

131. Ng I, Lee K, Kave B, Kluger M, Paynter C, Segal R, et al. HALO CleanSpace PAPR evaluation: Communication, respiratory protection, and usability. Infect Control Hosp Epidemiol. 2023;44(2):295-301. doi: 10.1017/ice.2022.71. PMID: 35361300

132. Rees P, Watson S, Corcoran J, Slade D, Pathmanaban O, Bibi A, et al. Powered air-purifying respirators: a solution to shortage of FFP3 filtering facepiece respirators in the operating theatre. Br J Surg. 2021;108(4):e160-e1. doi: 10.1093/bjs/znab008. PMID: 33778849

133. Sekoguchi S, Shirasaka T, Ando H, Ikegami K, Ogami A. Evaluation of the performance of replaceable particulate and powered air-purifying respirators considering non-recommended wearing methods. Ind Health. 2020;58(6). doi: 10.2486%2Findhealth.2020-0056. PMID: 32863380

134. Sekoguchi S, Ando H, Ikegami K, Yoshitake H, Baba H, Ogami A. Measurement of the workplace protection factor of replaceable particulate and powered air-purifying respirators in Japanese dust-generating occupations. J UOEH. 2022;44(1):15-24. doi: 10.7888/juoeh.44.15. PMID: 35249937

135. Temmesfeld MJ, Gorzkowska-Sobas AA, Hedlund K, Øyen MØ, Kanten L, Grant P, et al. Surgical helmets can be converted into efficient disinfectable powered air-purifying respirators. Am J Infect Control. 2022;50(6):624-30. doi: 10.1016/j.ajic.2021.12.002. PMID: 34958857

136. Xu X, Zhao L, Zhu Y, Du B, Zhu B, Zhang H, et al. Conducting quantitative mask fit tests: application details and affecting factors. Front Public Health. 2023;11. doi: 10.3389/fpubh.2023.1218191.

137. Rowlett JM. The Perceptions of the Quantitative and Qualitative Fit Testing Protocols and Relevance of the Clean-Shaven Guidance for the Current Field of Reusable Elastomeric Half-Mask Respirators. Doctoral dissertation. Indiana University of Pennsylvania. 2021. Available from: <https://www.proquest.com/openview/81c0bc1ae4ea1bc16767325e4240ec44/1?pq-origsite=gscholar&cbl=18750&diss=y>. .
