# Supplementary material for: A systematic review of passing fit testing of the masks and respirators used during the COVID-19 pandemic: Part 1-quantitative fit test procedures
Source: PLoS One. 2023 Oct 26;18(10):e0293129. doi: 10.1371/journal.pone.0293129 (PMC10602271; doi:10.1371/journal.pone.0293129)
Supplement: S4 Appendix — (DOCX) [file pone.0293129.s004.docx]

**S4 Appendix. Proportions of Study Types and Paper Types Included in the Systematic Review**

| **No.** | **Study** | **Title** | **Study type** | **Paper type** |
| --- | --- | --- | --- | --- |
|  | Brandel et al., 2020 [1] | Testing and Developing DIY Masks | Design project | Report |
|  | Buckley et al., 2020 [2] | Quantitative Respirator Fit Testing of HensMask | Experimental study | Article, preprint |
|  | Coyle et al., 2021 [3] | Reduction of exposure to simulated respiratory aerosols using ventilation, physical distancing, and universal masking | Experimental study | Original Article |
|  | Dang et al., 2021 [4] | Challenges in Predicting the Filtration Performance of a Novel Sewn Mask: Scale-up from Filter Holder to Mannequin Measurements | - | Original Research |
|  | Drouillard et al., 2022 [5] | Fitted filtration efficiency and breathability of 2-ply cotton masks: Identification of cotton consumer categories acceptable for homemade cloth mask construction | Experimental study | Research Article |
|  | Duncan et al., 2021 [6] | The protective performance of reusable cloth face masks, disposable procedure masks, KN95 masks and N95 respirators: Filtration and total inward leakage | Experimental study | Research Article |
|  | Mueller et al., 2020 [7] | Quantitative Method for Comparative Assessment of Particle Removal Efficiency of Fabric Masks as Alternatives to Standard Surgical Masks for PPE | Experimental study | Matter Article |
|  | Reutman et al., 2021 [8] | Homemade facemasks: particle filtration, breathability, fit, and other performance characteristics | Experimental study | Original Research |
|  | Teesing et al., 2020 [9] | Is there an adequate alternative to commercially manufactured face masks? A comparison of various materials and forms | - | Article |
|  | Wentworth et al., 2020 [10] | Advanced Manufacturing Center_Comparative Protective Mask Material Testing | Experimental study | Report |
|  | Lindsley et al., 2021 [11] | Efficacy of face masks, neck gaiters and face shields for reducing the expulsion of simulated cough-generated aerosols | Experimental study | Original Article |
|  | Sato et al., 2020 [12] | Measurement of the leak rate of masks used for anticancer drug handling using a mask fitting tester, tape medical | Experimental study | Original Article |
|  | Ardon-Dryer et al., 2021 [13] | Mask Material Filtration Efficiency and Mask Fitting at the Crossroads: Implications during Pandemic Times | Experimental study | Original Research |
|  | Bodas et al., 2022 [14] | A randomised crossover trial of two flat-fold cup respirators: BYD DE2322 N95 versus Care Essentials MSK-002 P2 | Prospective randomized, non-blinded crossover study | Research paper |
|  | Cameron et al., 2020  [15] | Quantitative fit testing with limited supplies of respirator masks in hospital personnel during the COVID-19 pandemic | Experimental study, cross-sectional study | Letter to the Editor |
|  | Chan et al., 2021 [16] | Fit Testing Disposable P2/N95 Respirators during COVID-19 in Victoria, Australia: Fit Check Evaluation, Failure Rates, and a Survey of Healthcare Workers | Observational study | Original Article |
|  | Christopher et al., 2021 [17] | The PPE Pandemic: Sex-Related Discrepancies of N95 Mask Fit | Retrospective review | Original Article |
|  | Cloet et al., 2022 [18] | Design considerations for protective mask development: A remote mask usability evaluation | Randomized Controlled Trial, repeated measures, mixed methods design, convenience sampling | Article |
|  | Cloet et al., 2022 [19] | Activity And Usability Evaluation of Alternative  Protective Face Mask Designs | Randomized trial with  convenience sampling | Research Article |
|  | Griffin et al., 2022 [20] | Protective Masks Utilizing Nonendangered Components | - | Design Innovation Paper |
|  | Duncan et al., 2020 [21] | The impact of extreme reuse and extended wear conditions on protection provided by a surgicalstyle N95 filtering facepiece respirator | Trial study | Article |
|  | Fabre et al., 2021 [22] | N95 Filtering Facepiece Respirators Remain Effective After Extensive Reuse During the COVID-19 Pandemic | Experimental study, cross-sectional | Research brief |
|  | Nakamoto et al., 2021 [23] | Reusing N95 Respirators at Weekly Intervals During the COVID-19 Pandemic | Experimental study | Original Article |
|  | Greenawald et al., 2021 [24] | Evaluation of Fit and Strap Extension Performance of Stockpiled Filtering Facepiece Respirators from One U.S. Facility | - | - |
|  | Hai et al., 2022 [25] | Comparing Quality of Fitting of Different Filtering Face Pieces in a Healthcare Worker: A preliminary observational study | A preliminary observational study | Article |
|  | Han et al., 2021 [26] | Comparison of N95 Respiratory Mask Fit Testing | Descriptive survey | Original Article |
|  | Hwang et al., 2020 [27] | N95 filtering facepiece respirators do not reliably afford respiratory protection during chest compression: A simulation study | Single-center simulation study | Article |
|  | Fakherpour et al., 2021 [28] | Quantitative fit testing of filtering face-piece respirators during the COVID-19 pandemic reveals anthropometric deficits in most respirators available in Iran | Cross-sectional study | Research Article |
|  | Jankusol et al., 2023 [29] | Different fit factors for the N95 respirator during endotracheal intubation: Comparing video laryngoscopy and direct laryngoscopy | Randomized crossover study | Research Article |
|  | Joshi et al., 2021 [30] | Quick laboratory methodology for determining the particle filtration efficiency of face masks/ respirators in the wake of COVID-19 pandemic | Experimental study | Original Research |
|  | Jean-Romain et al., 2021 [31] | Distribution of low quality filtering facepiece respirators during the COVID-19 pandemic: an independent analysis of the situation in Switzerland | Experimental study | Original article |
|  | Jung et al., 2021 [32] | Fit-failure rate associated with simulated reuse and extended use of N95 respirators assessed by a quantitative fit test | Experimental study | Original Article |
|  | Kamal et al., 2023 [33] | Safety Goggles with Elastic Headband to Improve N95 Fit Following Failed Quantitative Fit Test | Prospective before-and-after study | Original Article |
|  | Kyaw et al., 2021 [34] | Prediction of N95 Respirator Fit from Fogging of Eyeglasses: A Pilot Study | A Pilot Study | Original Article |
|  | Landry et al., 2022 [35] | Fit-Tested N95 Masks Combined With Portable High-Efficiency Particulate Air Filtration Can Protect Against High Aerosolized Viral Loads Over Prolonged Periods at Close Range | Experimental design | Major Article |
|  | Lindsley et al., 2021 [36] | A comparison of performance metrics for cloth face masks as source control devices for simulated cough and exhalation aerosols | Experimental study | Original Research |
|  | Long et al., 2022 [37] | Measurement of filtration efficiencies of healthcare and consumer materials using modified respirator fit tester setup | - | Research Article |
|  | Milosevic et al., 2021 [38] | P2/N95 filtering facepiece respirators: Results of a large-scale quantitative mask fit testing program in Australian health care workers | Experimental study | Major article |
|  | Ng et al., 2022 [39] | N95 respirators: quantitative fit test pass rates and usability and comfort assessment by health care workers | - | Research |
|  | O’Kelly et al., 2021 [40] | How well do face masks protect the wearer compared to public perceptions? | Experimental study | Article |
|  | O’Kelly et al. 2021 [41] | Comparing the fit of N95, KN95, surgical, and cloth face masks and assessing the accuracy of fit checking | Experimental study | Research Article |
|  | O’Kelly et al., 2022 [42] | Experimental Measurement of the Size of Gaps Required to Compromise Fit of an N95 Respirator | Experimental study | Original Research |
|  | Park et al., 2021 [43] | Fit Test for N95 Filtering Facepiece Respirators and KF94 Masks for Healthcare Workers: a Prospective Single-center Simulation Study | Prospective Single-center Simulation Study | Original Article |
|  | Popov et al., 2022 [44] | Development and Application of a Modified Procedure for Quantitative Fit Testing of Disposable Masks and Respirators | Experimental study | Article |
|  | Regli et al., 2022 [45] | N95 Masks to Protect Health Care Workers Is the New Fast Fit-Test Protocol Cutting Corners? | Audit, Experimental study | Research Letter |
|  | Regli et al., 2021 [46] | More than half of front-line healthcare workers unknowingly used an N95/P2 mask without adequate airborne protection: An audit in a tertiary institution | Audit | Article |
|  | Prince et al., 2021 [47] | Assessing the effect of beard hair lengths on face masks used as personal protective equipment during the COVID-19 pandemic | Experimental study | Article |
|  | Sandaradura et al., 2020 [48] | A close shave? Performance of P2/N95 respirators in healthcare workers with facial hair: results of the BEARDS (BEnchmarking Adequate Respiratory DefenceS) study | Experimental study | Short Report |
|  | De-Yñigo-Mojado et al., 2021 [49] | Facial Hair Decreases Fit Factor of Masks and Respirators in Healthcare Providers. | Controlled randomized clinical trial study | Article |
|  | Sasko et al., 2023 [50] | Fit testing of masks worn by frontline healthcare workers | Cross-sectional study | Research Brief |
|  | Seo et al., 2021 [51] | Necessity of the Fit Test Panel for Korean Respirator Users: Application to Korean Healthcare Workers | Experimental study | ISRP Original research |
|  | Seo et al., 2020 [52] | Fit testing for domestic N95 medical masks Korean-translation | - | Original Article |
|  | Sheikh et al., 2022 [53] | N95 Respirator Fit for a Diverse Population of Healthcare Workers: A Mixed-Methods, Prospective, Pilot and Feasibility Study | A multi-center mixed-method Prospective, Pilot And Feasibility study, convergent parallel design, Convenience and purposive sampling | Thesis |
|  | Sickbert Bennett et al., 2020 [54] | Filtration Efficiency of Hospital Face Mask Alternatives Available for Use During the COVID-19 Pandemic | Quality-improvement study | Original Investigation |
|  | Suen et al., 2022 [55] | Comparing mask fit and usability of traditional and nanofibre N95 filtering facepiece respirators before and after nursing procedures | Experimental study, one-group multiple comparison design. | Article |
|  | Goh et al., 2022 [56] | A randomised clinical trial to evaluate the safety, fit, comfort of a novel N95 mask in children | Randomised, two-period crossover clinical trial study | Scientific Reports |
|  | Salter et al., 2021 [57] | Reinventing Cloth Masks in the Face of Pandemics | Experimental study | Original Research Article |
|  | [Vahabzadeh‐Hagh](https://scholar.google.com/citations?user=JOaLyxoAAAAJ&hl=en&oi=sra) et al., 2022 [58] | Patient-worn endoscopy mask to protect against viral  transmission | Single subject study | Original Search |
|  | Vo et al., 2020 [59] | A technique to measure respirator protection factors against aerosol particles in simulated workplace settings using portable instruments | Trial study | Article |
|  | Vuma et al., 2021 [60] | The Effect on Fit of Multiple Consecutive Donning and Doffing of N95 Filtering Facepiece Respirators | Experimental study | Original Article |
|  | Williams et al., 2021 [61] | A randomised crossover study to compare the user seal check and quantitative fit test between two types of duckbill N95 particulate respirator masks: The Halyard FluidshieldVR N95 and the BSN Medical ProShieldVR N-95 particulate respirator masks | Randomized Controlled Trial, A randomised crossover study | Article |
|  | Williams et al., 2021 [62] | Randomized crossover study comparing quantitative fit tests between Trident_ and 3M_ Aura_ N95/P2 respirators | Prospective randomized crossover study | Research paper |
|  | Williams et al., 2022 [63] | Quantitative fit-test concordance of a pair of similar-fit 3M Aura respirator models, 3M 9320Aþ and 3M 1870þ: A randomized crossover study | Prospective randomized crossover study | Original Article |
|  | Williams et al., 2022 [64] | Optimizing twin sampling tube stabilization improves quantitative fit  test results for flat-fold duckbill filtering facepiece respirators | Prospective randomized crossover study | Major Article |
|  | Lim et al., 2020 [65] | Eﬀects of Particulate Respirator Use on Cardiopulmonary Function in Elderly Women: a Quasi-Experimental Study | Quasi-experimental study | Original Article |
|  | Mottay et al., 2020 [66]  ‎ | *KN95* filtering facepiece respirators distributed in South Africa fail safety testing protocols | Experimental study, Observational study | Research |
|  | Zhang et al., 2020 [67] | The relationship between the filtering facepiece respirator fit and the facial anthropometric dimensions | Experimental study | Original Article |
|  | Boogaard et al., 2020 [68] | Efficacy assessment of newly-designed and locally-produced filtering facemasks during the SARS-CoV-2 pandemic | Experimental study | Original Research |
|  | Carvalho et al., 2021 [69] | Prospective observational study of gender and ethnicity biases in respiratory protective equipment for healthcare workers in the COVID-19 pandemic | Prospective observational study | Original research |
|  | Caggiari et al., 2023 [70] | Retrospective evaluation of factors affecting successful fit testing of respiratory protective equipment during the early phase of COVID-19 | Retrospective study | Original Research |
|  | De‐Yñigo‐Mojado et al., 2021 [71] | Fit factor compliance of masks and FFP3 respirators in nurses: A case–control gender study | Case – control  gender study | Original Research Empirical Research – Quantitative |
|  | De-Yñigo-Mojado et al., 2020 [72] | Fit factor of masks used by Physicians in Clinical Settings | Cross-sectional study | Research Paper |
|  | Green et al., 2021 [73] | Fit-testing of respiratory protective equipment in the UK during the initial response to the COVID-19 pandemic | Retrospective analysis | Article |
|  | Sun et al., 2020 [74] | Evaluation of a New Workplace Protection Factor Measuring Method for Filtering Facepiece Respirator | Experimental study | Original Article |
|  | Vanhooydonck et al., 2021 [75] | Case study into the successful emergency production and certification of a filtering facepiece respirator for Belgian hospitals during the COVID-19 pandemic | Case study | Article |
|  | Winski et al., 2019 [76] | If the mask fits: Facial dimensions and mask performance | Experimental study | Article |
|  | Chapman et al., 2022 [77] | Quantitative respirator fit tests for P2/N95 in Australian general practice | Case study | Letter |
|  | Chen et al., 2022 [78] | Improvement in Fitted Filtration Efficiency of N95 Respirators With Escalating Instruction of the Wearer | Experimental study | Quality Improvement |
|  | Clark et al., 2021 [79] | Alteration of Perceptions of Safety Before and After Fit Testing among College of Dentistry Students | Cross-sectional study | Capstone Experience |
|  | Inolopú et al., 2023 [80] | Quantitative fit testing on filtering facepiece respirators in use by Peruvian healthcare workers caring for tuberculosis patients during the COVID-19 pandemic: PROFIT study 2020 | - | Article |
|  | Low et al., 2021 [81] | Pass rate of the BSN Medical ProShieldVR N95 filtering facepiece using quantitative fit testing in frontline anaesthetists and anaesthesia nurses working on a COVID-19 airway team | Single-Centre, Prospective Cohort study | Letter |
|  | Ngobeni et al., 2020 [82] | Qualitative Versus Quantitative Fit-Testing of Two Commonly Used Respirators in Resource-Limited Healthcare Facilities | Correlation non-experimental cross-sectional study | Dissertation or Thesis |
|  | Robertsen et al., 2020 [83] | The Effect of a Knowledge-Based Intervention on the Use of Respirators in the Norwegian Smelter Industry | Controlled before-and-after study | Original Research |
|  | Seo et al., 2021 [84] | Fit comparison of Domestic N95 Medical Masks in a Fit test- Korean-translation | - | Original Article |
|  | Williams et al., 2021 [85] | Impacts on staff after implementation of a respiratory protection program in a Victorian public hospital | Prospective observational study | Research paper |
|  | Yeon et al., 2020 [86] | Effects of Education on the Use of Personal Protective Equipment for Reduction of Contamination: A Randomized Trial | Randomized control group pretest–posttest design (Randomized Controlled Trial) |  |
|  | Xiao et al., 2023 [87] | Investigation of a Mask Fitness Test Based on Self-Efficacy and Diversified Training in the Assessment System for Nosocomial Infection Training | - | Original Research |
|  | Anwari et al., 2021 [88] | Development, manufacturing, and preliminary validation of a reusable half-face respirator during the COVID-19 pandemic | Validation study | Research Article |
|  | Chichester et al., 2020 [89] | Evaluation of an Additively Manufactured Respirator for Personnel Protection from Particulates | Design | Technical Report |
|  | Fadairo et al., 2020 [90] | Comparison of Condensation Nuclei Counter and Controlled Negative Pressure Methods under Different Environmental Conditions Tested with a Mannequin and Human Subjects | Comparative study | PhD Thesis |
|  | Hondjeu et al., 2021 [91] | A reusable, locally manufactured, half- face respirator provides better protection than fit tested disposable N95 masks: development and quantitative fit-testing comparison | Experimental study | Research Article |
|  | Ballard et al., 2021 [92] | Quantitative Fit Tested N95 Respirator-Alternatives Generated With CT Imaging and 3D Printing: A Response to Potential Shortages During the COVID-19 Pandemic | Experimental study | Original Investigation |
|  | Ballard et al., 2021 [93] | Protection levels of N95-level respirator substitutes proposed during the COVID-19 pandemic: safety concerns and quantitative evaluation procedure | Experimental study | Original research |
|  | Duda et al., 2020 [94] | Potential risks of a widespread use of 3D printing for the manufacturing of face masks during the severe acute respiratory syndrome coronavirus 2 pandemic | Experimental study | Research Article |
|  | Imbrie-Moore et al., 2020 [95] | Quadrupling the N95 supply during the COVID-19 crisis with an innovative 3D-printed mask adaptor | Experimental study | Communication |
|  | Levine et al., 2022 [96] | Face Off: 3D-Printed Masks as a Cost-Effective and Reusable Alternative to N95 Respirators: A Feasibility Study | A Feasibility Study | Clinical Research Study |
|  | Liu et al., 2020 [97] | Adapting re-usable elastomeric respirators to utilize anaesthesia circuit filters using a 3D-printed adaptor – a potential alternative to address N95 shortages during the COVID-19 pandemic | Experimental study | Original Article |
|  | Manomaipiboon et al., 2020 [98] | The new silicone N99 half-piece respirator, VJR-NMU N99: A novel and effective tool to prevent COVID-19 | - | Research Article |
|  | Martelly et al., 2021 [99] | Moldable mask: a reusable, hot water moldable, additively manufactured mask to be used as an n95 alternative | Experimental study | Article |
|  | Meadwell et al., 2019 [100] | In search of a performing seal: rethinking the design of tight-fitting respiratory protective equipment facepieces for users with facial hair | Experimental study | Original Article |
|  | McLeod et al., 2021 [101] | An exploration of thermomechanical softwood pulp for n95 respiratory mask production | Experimental study | Thesis |
|  | Ng et al., 2020 [102] | Subject validation of reusable N95 stop-gap filtering facepiece respirators in COVID-19 pandemic | Experimental, Cohort study | Research Article |
|  | Roche et al., 2022 [103] | Personalised 3D printed respirators for healthcare workers during the COVID-19 pandemic | Randomized Controlled Trial (RCT) | Original Research |
|  | Chughtai et al., 2020 [104] | Selection and use of respiratory protection by healthcare workers to protect from infectious diseases in hospital settings | Experimental study | Original Article |
|  | Germonpre et al., 2020 [105] | Evaluation of protection level, respiratory safety, and practical aspects of commercially available snorkel masks as personal protection devices against aerosolized contaminants and sars-cov2 | Experimental study | Article |
|  | Greig et al., 2020 [106] | Safety testing improvised COVID-19 personal protective equipment based on a modified full-face snorkel mask | - | Correspondence |
|  | Greig et al., 2022 [107] | A crossover study assessing the protective efficacy of improvised snorkel-based improvised respirators | A crossover study, convenience sample |  |
|  | Grinshpun et al., 2020 [108] | Evaluation of AccuFIT 9000: a novel apparatus for quantitative fit testing of particulate respirators | Experimental study | Original Article |
|  | Harmata et al., 2022 [109] | Rules for fitting filtering gas masks | - | Original Article |
|  | Kechli et al., 2020 [110] | Modifying a full-face snorkel mask to meet n95 respirator standards for use with coronavirus disease 2019 patients | Experimental study | Innovation |
|  | Kroo et al., 2021 [111] | Pneumask: modified full-face snorkel masks as reusable personal protective equipment for hospital personnel | Experimental study | Research Article |
|  | Nicholson et al., 2021 [112] | Modified full-face snorkel mask as COVID-19 personal protective equipment: Quantitative results | Experimental study | Original Research |
|  | Persing et al., 2021 [113] | Comparing respirator laboratory protection factors measured with novel personal instruments to those from the PortaCount | Experimental study | Article |
|  | Pettinger et al., 2021 [114] | Verification of two alternative do-it-yourself equipment respirators seal as covid-19 protection (vaders-cov): a quality assessment pilot study | Quality assessment pilot study | Research Article |
|  | Bergman et al., 2019 [115] | Assessment of respirator fit capability test criteria for full-facepiece air-purifying respirators | Experimental study | Article |
|  | Chehade et al., 2021 [116] | Feasibility study: Proposed alternative to N95 respirator during the personal protective equipment shortage from COVID-19 pandemic | Single-arm, single-center, feasibility study | Article |
|  | Han et al., 2022 [117] | Comparisons of fit factors between two quantitative fit testers (PortaCount® vs MT®) | - | Short Communication |
|  | Rengasamy et al., 2021 [118] | Evaluation of total inward leakage for NIOSH approved elastomeric half-facepiece, fullfacepiece, and powered air-purifying respirators using sodium chloride and corn oil aerosols | Randomized Controlled Trial | Article |
|  | Sietsema et al., 2022 [119] | Simulated workplace protection factor study of a quarter-facepiece elastomeric respirator | Experimental study | Research Article |
|  | Weng et al., 2022 [120] | A full‑face mask for protection against respiratory infections | validation study | Research |
|  | Clinkard et al., 2021 [121] | Evaluation of N95 respirators, modified snorkel masks and low-cost powered air-purifying respirators: a prospective observational cohort study in healthcare workers | A prospective observational cohort study | Original Article |
|  | Convissar et al., 2020 [122] | Personal protective equipment n95 facemask shortage quick fix: the modified airway from ventilator circuit (maveric) | Experimental study | Technical Report |
|  | Toigo et al., 2021 [123] | Fit testing retrofitted full-face snorkel masks as a form of novel personal protective equipment during the covid-19 pandemic | A retrospective  study | Brief Report |
|  | Cass et al., 2022 [124] | The adequacy of user seal checking for N95 respirators compared to formal fit testing: A multicentred observational study | A prospective, observational study | Research paper |
|  | Baba et al., 2022 [125] | Comparison of respiratory protection during exercising tasks between different 1 wearing methods of replaceable particulate respirators and powered air-purifying respirators | Experimental study, Crossover comparison study | Case Report |
|  | Grinshpun et al., 2020 [126] | New respirator performance monitor (RePM) for powered air-purifying respirators | Validation study | Research Article |
|  | Kessel et al., 2022 [127] | An Emergency Powered Air-Purifying Respirator from Local Materials and its Efficacy Against Aerosolized Nanoparticles. | Experimental study |  |
|  | McGrath et al., 2022 [128] | Bubble-PAPR: Phase I clinical evaluation of an ‘in-house’ developed prototype powered air-purifying respirator for use by healthcare workers | Design study  Trial | Article |
|  | Nagel et al., 2021 [129] | Novel 3D printable powered air purifying respirator for emergency use during PPE shortage of the COVID-19 pandemic: a study protocol and device safety analysis | Experimental study | Original research |
|  | Goto et al., 2021 [130] | The protective effect of tight-fitting powered air-purifying respirators during chest compressions | Single-center simulation study | Article |
|  | Ng et al., 2023 [131] | HALO CleanSpace PAPR evaluation: Communication, respiratory protection, and usability | Simulation study | Original Article |
|  | Rees et al., 2021 [132] | Powered air-purifying respirators: a solution to shortage of FFP3 filtering facepiece respirators in the operating theatre | - | Research Letter |
|  | Sekoguchi et al., 2020 [133] | Evaluation of the performance of replaceable particulate and powered air-purifying respirators considering non-recommended wearing methods | Crossover trial | Case Report |
|  | Sekoguchi et al., 2022 [134] | Measurement of the workplace protection factor of replaceable particulate and powered air-purifying respirators in japanese dust-generating occupations | Crossover trial | Original Research |
|  | Temmesfeld et al., 2022 [135] | Surgical helmets can be converted into efficient disinfectable powered air-purifying respirators | Experimental study | Major Article |
|  | Xu et al., 2023 [136] | Conducting quantitative mask fit tests: application details and affecting factors | Experimental study | Original Research |
|  | Rowlett et al., 2021 [137] | The perceptions of the quantitative and qualitative fit testing protocols and relevance of the clean-shaven guidance for the current | Observational, Cross-sectional study | PhD Thesis |

**References**

.1 Brandel A, Tanner J, Gao J, Kelly N, Snyder A. Testing and Developing DIY Masks. University of Michigan. 2020. Available from: <https://deepblue.lib.umich.edu/handle/2027.42/164436?show=full>.

.2 Buckley J, Gladle M, Murray K, Sample W. SUBJECT: Quantitative Respirator Fit Testing of HensMask. University of Delaware. 2020. Available from: <https://me.udel.edu/wp-content/uploads/2020/04/HensNest-FitTest.pdf>.

.3 Coyle JP, Derk RC, Lindsley WG, Boots T, Blachere FM, Reynolds JS, et al. Reduction of exposure to simulated respiratory aerosols using ventilation, physical distancing, and universal masking. Indoor Air. 2022;32(2):e12987. doi: 10.1111/ina.12987. PMID: 35225389

.4 Dang AJ, Kumfer BM, Bertroche JT, Glidden JO, Oxford CR, Jammalamadaka U, et al. Challenges in predicting the filtration performance of a novel sewn mask: Scale-up from filter holder to mannequin measurements. Aerosol Air Qual Res. 2021;21(6). doi: 10.4209/aaqr.200629.

.5 Drouillard KG, Tomkins A, Lackie S, Laengert S, Baker A, Clase CM, et al. Fitted filtration efficiency and breathability of 2-ply cotton masks: Identification of cotton consumer categories acceptable for home-made cloth mask construction. PLoS One. 2022;17(3):e0264090. doi: 10.1371/journal.pone.0264090. PMID: 35316263

.6 Duncan S, Bodurtha P, Naqvi S. The protective performance of reusable cloth face masks, disposable procedure masks, KN95 masks and N95 respirators: Filtration and total inward leakage. PLoS One. 2021;16(10):e0258191. doi: 10.1371/journal.pone.0258191. PMID: 34614026

.7 Mueller AV, Eden MJ, Oakes JM, Bellini C, Fernandez LA. Quantitative Method for Comparative Assessment of Particle Removal Efficiency of Fabric Masks as Alternatives to Standard Surgical Masks for PPE. Matter. 2020;3(3):950-62. doi: 10.1016/j.matt.2020.07.006. PMID: 32838296

.8 Reutman SR, Reponen T, Yermakov M, A. Grinshpun S. Homemade facemasks: particle filtration, breathability, fit, and other performance characteristics. J Occup Environ Hyg. 2021;18(7):334-44. doi: 10.1080/15459624.2021.1925124. PMID: 34080950

.9 Teesing GR, van Straten B, de Man P, Horeman-Franse T. Is there an adequate alternative to commercially manufactured face masks? A comparison of various materials and forms. J Hosp Infect. 2020;106(2):246-53. doi: 10.1016/j.jhin.2020.07.024. PMID: 32763333

.10 Wentworth F. Advanced Manufacturing Center_Comparative Protective Mask Material Testing. 2020.

.11 Lindsley WG, Blachere FM, Law BF, Beezhold DH, Noti JD. Efficacy of face masks, neck gaiters and face shields for reducing the expulsion of simulated cough-generated aerosols. Aerosol Sci Technol. 2021;55(4):449-57. doi: 10.1080/02786826.2020.1862409. PMID: 35924077

.12 Sato J, Yamawaki Y, Ito M, Endo M, Tanaka R, Shino M. Measurement of the leak rate of masks used for anticancer drug handling using a mask fitting tester. J Oncol Pharm Pract. 2020;26(6):1318-23. doi: 10.1177/1078155219891210. PMID: 31822202

.13 Ardon-Dryer K, Warzywoda J, Tekin R, Biros J, Almodovar S, Weeks BL, et al. Mask Material Filtration Efficiency and Mask Fitting at the Crossroads: Implications during Pandemic Times. Aerosol Air Qual Res. 2021;21(7). doi: 10.4209/aaqr.200571.

.14 Bodas CR, Ng I, Kave B, Begg F, Williams DL. A randomised crossover trial of two flat-fold cup respirators: BYD DE2322 N95 versus Care Essentials MSK-002 P2. Infect Dis Health. 2022. doi: 10.1016/j.idh.2022.08.002. PMID: 36207250

.15 Cameron S, Cheung W, Cronin N, Griffiths K, Hunt R, Innes L, et al. Quantitative fit testing with limited supplies of respirator masks in hospital personnel during the COVID-19 pandemic. Aust Health Rev. 2020;44(4):542-3. doi: 10.1071/ah20154. PMID: 32718421

.16 Chan JK, Yep KH, Magarey S, Keon-Cohen Z, Acheson M. Fit Testing Disposable P2/N95 Respirators during COVID-19 in Victoria, Australia: Fit Check Evaluation, Failure Rates, and a Survey of Healthcare Workers. COVID. 2021;1(1):83-96. doi: 10.3390/covid1010007.

.17 Christopher L, Rohr-Kirchgraber T, Mark S. The PPE Pandemic: Sex-Related Discrepancies of N95 Mask Fit. EMJ Microbiol Infect Dis.2(1):57-63. doi: 10.33590/emjmicrobiolinfectdis/20-00215.

.18 Cloet A, Yu M, Arnold J, Griffin L. Activity and Usability Evaluation of Alternative Protective Face Mask Designs. Proc Hum Factors Ergon Soc Annu Meet. 2022;66(1):1682-6. doi: 10.1177/1071181322661162.

.19 Cloet A, Griffin L, Yu M, Durfee W. Design considerations for protective mask development: A remote mask usability evaluation. Appl Ergon. 2022;102:103751. doi: 10.1016/j.apergo.2022.103751. PMID: 35339761

.20 Griffin L, Yu MJ, Cloet A, Arnold S, Carlson N, Hillmyer M, et al. Protective Masks Utilizing Nonendangered Components. J Med Device. 2022;16(1). doi: 10.1115/1.4053720. PMID: 35280214

.21 Duncan S, Bodurtha P, Bourgeois C, Dickson E, Jensen C, Naqvi S. The impact of extreme reuse and extended wear conditions on protection provided by a surgical-style N95 filtering facepiece respirator. J Occup Environ Hyg. 2020;17(11-12):546-59. doi: 10.1080/15459624.2020.1829633. PMID: 33166226

.22 Fabre V, Cosgrove SE, Hsu YJ, Jones GF, Helsel T, Bukowski J, et al. N95 filtering face piece respirators remain effective after extensive reuse during the coronavirus disease 2019 (COVID-19) pandemic. Infect Control Hosp Epidemiol. 2021;42(7):896-9. doi: 10.1017/ice.2021.76. PMID: 33602376

.23 Nakamoto K, SARAYA Sr T, Kurai D, Fukukawa N, Taneoka T, Shimasaki T, et al. Reusing N95 Respirators at Weekly Intervals During the COVID-19 Pandemic. Cureus. 2021;13(2).

.24 Greenawald LA, Moore SM, Yorio PL. PPE CASE: Evaluation of Fit and Strap Extension Performance of Stockpiled Filtering Facepiece Respirators from One U.S. Facility. By Greenawald LA, Moore SM, and Yorio PL. Pittsburgh, PA: U.S. Department of Health and Human Services, Centers for Disease Control and Prevention, National Institute for Occupational Safety and Health, NPPTL Report Number P2021-0102. 2021.

.25 Hai CH, Hua KK, Fu GQ, Singh K, Wah YC. Comparing Quality of Fitting of Different Filtering Face Pieces in a Healthcare Worker: A preliminary observational study. J Posit School Psychol. 2022;6(3):4012–8.

.26 Han S-H, Cha K-S, Yoo S-Y, Han JO. Comparison of N95 Respiratory Mask Fit Testing. Korean J Healthc Assoc Infect Control 2021;26(2):108-14. doi: 10.14192/kjicp.2021.26.2.108.

.27 Hwang SY, Yoon H, Yoon A, Kim T, Lee G, Jung KY, et al. N95 filtering facepiece respirators do not reliably afford respiratory protection during chest compression: A simulation study. Am J Emerg Med. 2020;38(1):12-7. doi: 10.1016/j.ajem.2019.03.041. PMID: 30955924

.28 Fakherpour A, Jahangiri M, Seif M, Charkhand H, Abbaspour S, Floyd EL. Quantitative fit testing of filtering face-piece respirators during the COVID-19 pandemic reveals anthropometric deficits in most respirators available in Iran. J Environ Health Sci Eng. 2021;19(1):805-17. doi: 10.1007/s40201-021-00648-3. PMID: 33875931

.29 Jankusol K, Chaiear N, Mitsungnern T. Different fit factors for the N95 respirator during endotracheal intubation: Comparing video laryngoscopy and direct laryngoscopy. Asia Pac J Sci Technol. 2023;28(2):APST-28-02-12. doi: 10.14456/apst.2023.28.

.30 Joshi M, Khan A, Sapra BK. Quick laboratory methodology for determining the particle filtration efficiency of face masks/respirators in the wake of COVID-19 pandemic. J Ind Text.51(5S

):7622S-40S. doi: 10.1177/1528083720975084.

.31 Jean-Romain D, David V, Guillaume S, de Damien C, Walter Z, Vincent P, et al. Distribution of low quality filtering facepiece respirators during the COVID-19 pandemic: An independent analysis of the situation in Switzerland. Swiss Med Wkly. 2021;151(3). doi: 10.4414/smw.2021.20459. PMID: 33516162

.32 Jung J, Kim J, Yang H, Lim Y-J, Kwak S-H, Hong MJ, et al. Fit-failure rate associated with simulated reuse and extended use of N95 respirators assessed by a quantitative fit test. Infect Control Hosp Epidemiol. 2021;42(11):1313-7. doi: 10.1017/ice.2021.5. PMID: 33487185

.33 Kamal M, Bhatti M, Stewart WC, Johns M, Collins D, Shehabi Y, et al. Safety Goggles with Elastic Headband to Improve N95 Fit Following Failed Quantitative Fit Test. Indian J Crit Care Med

2023;27(6):386. doi: 10.5005/jpjournals-10071-24473. PMID: 37378367

.34 Kyaw S, Johns M, Lim R, Stewart WC, Rojas N, Thambiraj SR, et al. Prediction of N95 Respirator Fit from Fogging of Eyeglasses: A Pilot Study. Indian J Crit Care Med. 2021;25(9):976-80. doi: 10.5005/jp-journals-10071-23947. PMID: 34963713

.35 Landry SA, Subedi D, Barr JJ, MacDonald MI, Dix S, Kutey DM, et al. Fit-tested N95 masks combined with portable HEPA filtration can protect against high aerosolized viral loads over prolonged periods at close range. J Infect Dis. 2022; 226(2):199-207. doi: 10.1093/infdis/jiac195. PMID: 35535021

.36 Lindsley WG, Blachere FM, Beezhold DH, Law BF, Derk RC, Hettick JM, et al. A comparison of performance metrics for cloth masks as source control devices for simulated cough and exhalation aerosols. Aerosol Sci Technol. 2021;55(10):1125-42. doi: 10.1080/02786826.2021.1933377. PMID: 35923216

.37 Long KD, Woodburn EV, Berg IC, Chen V, Scott WS. Measurement of filtration efficiencies of healthcare and consumer materials using modified respirator fit tester setup. PLoS One. 2020;15(10). doi: 10.1371/journal.pone.0240499. PMID: 33048980

.38 Milosevic M, Biswas RK, Innes L, Ng M, Darendeliler AM, Wong A, et al. P2/N95 filtering facepiece respirators: Results of a large-scale quantitative mask fit testing program in Australian health care workers. Am J Infect Control. 2022;50(5). doi: 10.1016/j.ajic.2021.12.016. PMID: 34971710

.39 Ng I, Kave B, Begg F, Bodas CR, Segal R, Williams D. N95 respirators: quantitative fit test pass rates and usability and comfort assessment by health care workers. Med J Aust. 2022;217(2):88-93. doi: 10.5694/mja2.51585. PMID: 35645035

.40 O’Kelly E, Arora A, Ward J, Clarkson PJ. How well do face masks protect the wearer compared to public perceptions? medRxiv [Preprint] 2021 [Posted 2021 January 31]. [6 p.]. Available from: <https://www.medrxiv.org/content/10.1101/2021.01.27.21250645v1.full-text>. doi: 10.1101/2021.01.27.21250645.

.41 O'Kelly E, Arora A, Pirog S, Ward J, Clarkson PJ. Comparing the fit of N95, KN95, surgical, and cloth face masks and assessing the accuracy of fit checking. PLoS One. 2021;16(1). doi: 10.1371/journal.pone.0245688. PMID: 33481870

.42 O’Kelly E, Arora A, Pirog S, Ward J, Clarkson PJ. Experimental Measurement of the Size of Gaps Required to Compromise Fit of an N95 Respirator. Disaster Med Public Health Prep. 2022;17:1-13. doi: 10.1017/dmp.2022.23. PMID: 35057880

.43 Park JJ, Seo YB, Lee J. Fit Test for N95 Filtering Facepiece Respirators and KF94 Masks for Healthcare Workers: a Prospective Single-center Simulation Study. J Korean Med Sci. 2021;36(21):e140. doi: 10.3346/jkms.2021.36.e140. PMID: 34060256

.44 Popov T, Popov G, Basse A. Development and Application of a Modified Procedure for Quantitative Fit Testing of Disposable Masks and Respirators. J Occup Environ Hyg. 2022;19(5):266-70. doi: 10.1080/15459624.2022.2050741. PMID: 35259072

.45 Regli A, Sommerfield A, Thalayasingam P, von Ungern-Sternberg BS. N95 Masks to Protect Health Care Workers: Is the New Fast Fit-Test Protocol Cutting Corners? Chest. 2022;161(6):1606-8. doi: 10.1016/j.chest.2022.01.048. PMID: 35131299

.46 Regli A, Thalayasingam P, Bell E, Sommerfield A, von Ungern-Sternberg BS. More than half of front-line healthcare workers unknowingly used an N95/P2 mask without adequate airborne protection: An audit in a tertiary institution. Anaesth Intensive Care. 2021;49(5):404-11. doi: 10.1177/0310057X211007861. PMID: 34325537

.47 Prince SE, Chen H, Tong H, Berntsen J, Masood S, Zeman KL, et al. Assessing the effect of beard hair lengths on face masks used as personal protective equipment during the COVID-19 pandemic. J Expo Sci Environ Epidemiol. 2021;31(6):953-60. doi: 10.1038/s41370-021-00337-1. PMID: 34006963

.48 Sandaradura I, Goeman E, Pontivivo G, Fine E, Gray H, Kerr S, et al. A close shave? Performance of P2/N95 respirators in healthcare workers with facial hair: results of the BEARDS (BEnchmarking Adequate Respiratory DefenceS) study. J Hosp Infect. 2020;104(4):529-33. doi: 10.1016/j.jhin.2020.01.006. PMID: 31978416

.49 De-Yñigo-Mojado B, Becerro-de-Bengoa-Vallejo R, Losa-Iglesias ME, Madera-García J, Rodríguez-Sanz D, Calvo-Lobo C, et al. Facial Hair Decreases Fit Factor of Masks and Respirators in Healthcare Providers. Biology (Basel). 2021;10(10). doi: 10.3390/biology10101031. PMID: 34681128

.50 Sasko LM, Oliver B, Smith SM. Fit testing of masks worn by frontline healthcare workers. Infect Control Hosp Epidemiol. 2023:1-2. doi: 10.1017/ice.2022.268. PMID: 36594247

.51 Seo H, Myong J-P, Kang B-k, Kwon Y-i. Necessity of the Fit Test Panel for Korean Respirator Users: Application to Korean Healthcare Workers. J Int Soc Respir Prot. 2021;38(2):1-11.

.52 Seo H, Kang B-k, Kwon Y-i. Fit testing for domestic N95 medical masks. J Korean Soc Occup Environ Hyg. 2020;30(2):124-33. doi: 10.15269/JKSOEH.2020.30.2.124.

.53 Sheikh F. N95 Respirators for a Diverse Population of Healthcare Workers: A Mixed-Methods, Pilot and Feasibility Study. M.Sc. Thesis, McMaster University. 2022. Available from: <https://macsphere.mcmaster.ca/handle/11375/28032>.

.54 Sickbert-Bennett EE, Samet JM, Clapp PW, Chen H, Berntsen J, Zeman KL, et al. Filtration Efficiency of Hospital Face Mask Alternatives Available for Use During the COVID-19 Pandemic. JAMA Intern Med. 2020;180(12):1607-12. doi: 10.1001/jamainternmed.2020.4221. PMID: 32780113

.55 Suen LKP, Guo YP, Ho SSK, Au-Yeung CH, Lam SC. Comparing mask fit and usability of traditional and nanofibre N95 filtering facepiece respirators before and after nursing procedures. J Hosp Infect. 2020;104(3):336-43. doi: 10.1016/j.jhin.2019.09.014. PMID: 31545991

.56 Goh DYT, Mun MW, Lee WLJ, Teoh OH, Rajgor DD. A randomised clinical trial to evaluate the safety, fit, comfort of a novel N95 mask in children. Sci Rep. 2019;9(1):18952. doi: 10.1038/s41598-019-55451-w. PMID: 31831801

.57 Salter SJRA. Reinventing cloth masks in the face of pandemics. 2021;41(5):731-44.

.58 Vahabzadeh‐Hagh AM, Patel SH, Stramiello JA, Weissbrod PA. Patient‐worn endoscopy mask to protect against viral transmission. Laryngoscope Investig Otolaryngol. 2022;7(1):190-6. doi: 10.1002%2Flio2.708. PMID: 35155797

.59 Vo E, Horvatin M, Bergman M, Wu B, Zhuang Z. A technique to measure respirator protection factors against aerosol particles in simulated workplace settings using portable instruments. J Occup Environ Hyg. 2020;17(5):231-42. doi: 10.1080/15459624.2020.1735640. PMID: 32243774

.60 Vuma CD, Manganyi J, Wilson K, Rees D. The effect on fit of multiple consecutive donning and doffing of N95 filtering facepiece respirators. Ann Work Expo Health. 2019;63(8):930-6. doi: 10.1093/annweh/wxz060. PMID: 31504129

.61 Williams DL, Kave B, Lee K, Segal R, Krieser RB, Mezzavia PM, et al. A randomised crossover study to compare the user seal check and quantitative fit test between two types of duckbill N95 particulate respirator masks: The Halyard Fluidshield (R) N95 and the BSN Medical ProShield (R) N-95 particulate respirator masks. Anaesth Intensive Care. 2021;49(2):112-8. doi: 10.1177/0310057X20974022. PMID: 33818131

.62 Williams DL, Kave B, Begg F, Bodas C, Ng I. Randomized crossover study comparing quantitative fit tests between Trident (TM) and 3M (TM) Aura (TM) N95/P2 respirators. Infect Dis Health. 2022;27(2):61-5. doi: 10.1016/j.idh.2021.10.002. PMID: 34799300

.63 Williams DL, Kave B, Begg F, Bodas C, Ng I. Quantitative fit-test concordance of a pair of similar-fit 3M Aura respirator models, 3M 9320A+ and 3M 1870+: A randomized crossover study. Infect Control Hosp Epidemiol. 2022;44(2):1-4. doi: 10.1017/ice.2022.67. PMID: 35387701

.64 Williams DL, Kave B, Bodas C, Begg F, Roberts M, Ng I. Optimizing twin sampling tube stabilization improves quantitative fit test results for flat-fold duckbill filtering facepiece respirators. Am J Infect Control. 2022;51(6):694-8. doi: 10.1016/j.ajic.2022.09.026. PMID: 36216035

.65 Lim Y-H, Kim W, Choi Y, Kim H-C, Na G, Kim H-R, et al. Effects of Particulate Respirator Use on Cardiopulmonary Function in Elderly Women: a Quasi-Experimental Study. J Korean Med Sci. 2020;35(10). doi: 10.3346/jkms.2020.35.e64. PMID: 32174063

.66 Mottay L, Le Roux J, Perumal R, Esmail A, Timm L, Sivarasu S, et al. KN95 filtering facepiece respirators distributed in South Africa fail safety testing protocols. S Afr Med J. 2020;111(3):13162. doi: 10.7196/samj.2021.v111i3.15381. PMID: 33334390

.67 Zhang X, Jia N, Wang Z. The relationship between the filtering facepiece respirator fit and the facial anthropometric dimensions among Chinese people. Ind Health. 2020;58(4):318-24. doi: 10.2486/indhealth.2019-0158. PMID: 31787708

.68 Boogaard B, Tas A, Nijssen J, Broeren F, van den Dobbelsteen J, Verhoeven V, et al. Efficacy Assessment of Newly-designed Filtering Facemasks during the SARS-CoV-2 Pandemic. Aerosol Air Qual Res. 2021;21(3):200424. doi: 10.4209/aaqr.2020.07.0424.

.69 Carvalho CYM, Schumacher J, Greig PR, Wong DJN, El-Boghdadly K. Prospective observational study of gender and ethnicity biases in respiratory protective equipment for healthcare workers in the COVID-19 pandemic. BMJ Open. 2021;11(5):e047716. doi: 10.1136/bmjopen-2020-047716. PMID: 34016664

.70 Caggiari S, Bader D, Packman Z, Robinson J, Tranka S, Böhning D, et al. Retrospective evaluation of factors affecting successful fit testing of respiratory protective equipment during the early phase of COVID-19. BMJ Open. 2023;13(5):e065068. doi: 10.1136/bmjopen-2022-065068. PMID: 37230519

.71 De‐Yñigo‐Mojado B, Madera‐García J, Becerro‐De‐Bengoa‐Vallejo R, Losa‐Iglesias ME, Rodríguez‐Sanz D, Calvo‐Lobo C, et al. Fit factor compliance of masks and FFP3 respirators in nurses: A case–control gender study. J Adv Nurs. 2021. doi: 10.1111/jan.14823. PMID: 33733471

.72 De-Yñigo-Mojado B, Madera-García J, Becerro-de-Bengoa-Vallejo R, Losa-Iglesias ME, Rodríguez-Sanz D, San-Antolín M, et al. Fit factor of masks used by Physicians in Clinical Settings. Int J Med Sci. 2020;17(17):2696-702. doi: 10.7150/ijms.50657. PMID: 33162797

.73 Green S, Gani A, Bailey M, Brown O, Hing CB. Fit-testing of respiratory protective equipment in the UK during the initial response to the COVID-19 pandemic. J Hosp Infect. 2021;113:180-6. doi: 10.1016/j.jhin.2021.04.024. PMID: 33940089

.74 Sun C, Thelen C, Sanz IS, Wittmann A. Evaluation of a new workplace protection factor–measuring method for filtering facepiece respirator. Saf Health Work. 2020;11(1):61-70. doi: 10.1016/j.shaw.2019.11.001. PMID: 32206375

.75 Vanhooydonck A, Van Goethem S, Van Loon J, Vandormael R, Vleugels J, Peeters T, et al. Case study into the successful emergency production and certification of a filtering facepiece respirator for Belgian hospitals during the COVID-19 pandemic. J Manuf Syst. 2021;60:876-92. doi: 10.1016/j.jmsy.2021.03.016. PMID: 33814674

.76 Winski TA, Mueller WA, Graveling RA. If the mask fits: facial dimensions and mask performance. Int J Ind Ergon. 2019;72:308-10. doi: 10.1016/j.ergon.2019.05.011.

.77 Chapman D, Chapman L, Ganesan A. Quantitative respirator fit tests for P2/N95 in Australian general practice. Aust J Gen Pract. 2022;51. doi: 10.31128/ajgp-covid-51-1. PMID: 35172324

.78 Chen H, Pennington ER, Case MW, Tong H, Rappold AG, Samet JM, et al. Improvement in Fitted Filtration Efficiency of N95 Respirators With Escalating Instruction of the Wearer. AJPM Focus. 2022;1(1):100014. doi: 10.1016/j.focus.2022.100014. PMID: 36338466

.79 Clark TH. Alteration of Perceptions of Safety Before and After Fit Testing among College of Dentistry Students. MSc. degree, University of Nebraska Medical Center. 2021. Available from: <https://digitalcommons.unmc.edu/cgi/viewcontent.cgi?article=1136&context=coph_slce>.

.80 Inolopú J, Mayma K, Curisinche-Rojas M, Aylas R, Flores JA, Rosales J. Quantitative Fit Testing on Filtering Facepiece Respirators in Use by Peruvian Healthcare Workers Caring for Tuberculosis Patients During the COVID-19 Pandemic: PROFIT Study 2020. 2023. doi: 10.3390/ijerph20166618. PMID: 37623201

.81 Low CS, Weinberg L, Ellard LM, Hacking DF, Banyasz D. Pass rate of the BSN Medical ProShield® N95 filtering facepiece using quantitative fit testing in frontline anaesthetists and anaesthesia nurses working on a COVID-19 airway team. Anaesth Intensive Care. 2021;49(4):322-3. doi: 10.1177/0310057x21997150. PMID: 34039048

.82 Ngobeni K. Qualitative Versus Quantitative Fit-Testing of Two Commonly Used Respirators in Resource-Limited Healthcare Facilities: University of Johannesburg (South Africa); 2020.

.83 Robertsen Ø, Hegseth MN, Føreland S, Siebler F, Eisemann M, Vangberg HCB. The Effect of a Knowledge-Based Intervention on the Use of Respirators in the Norwegian Smelter Industry. Front Psychol. 2020;11:270. doi: 10.3389%2Ffpsyg.2020.00270. PMID: 32153476

.84 Seo H, Kwon Y-i, Myong J-P, Kang B-k. Fit comparison of Domestic N95 Medical Masks in a Fit test. J Korean Soc Occup Environ Hyg. 2021;31(1):94-104. doi: 10.15269/JKSOEH.2021.31.1.94.

.85 Williams D, Kave B, Begg F, Marshall C, Segal R, Ng I. Impacts on staff after implementation of a respiratory protection program in a Victorian public hospital. Infect Dis Health. 2021;26(4):265-72. doi: 10.1016/j.idh.2021.06.001. PMID: 34176771

.86 Yeon JH, Shin YS. Effects of education on the use of personal protective equipment for reduction of contamination: a randomized trial. SAGE Open Nurs. 2020;6:2377960820940621. doi: 10.1177/2377960820940621. PMID: 33415295

.87 Xiao B, Sun L-L, Yuan J, Xiao W-L, Liu Y, Cai M-Y, et al. Investigation of a Mask Fitness Test Based on Self-Efficacy and Diversified Training in the Assessment System for Nosocomial Infection Training. Infect Drug Resist. 2023;16:313-22. doi: 10.2147/idr.s388784. PMID: 36691491

.88 Anwari V, Ng WCK, Hondjeu ARM, Xiao ZX, Afenu E, Trac J, et al. Development, manufacturing, and preliminary validation of a reusable half-face respirator during the COVID-19 pandemic. PLoS One. 2021;16(3). doi: 10.1371/journal.pone.0247575. PMID: 33730106

.89 Chichester DL, Hix JD, Johnson JT, Ocampo Giraldo LA, Watson SM, Mortensen BT, et al. Evaluation of an Additively Manufactured Respirator for Personnel Protection from Particulates. Idaho National Lab.(INL), Idaho Falls, ID (United States); 2020.

.90 Fadairo OJ. Comparison of Condensation Nuclei Counter and Controlled Negative Pressure Methods under Different Environmental Conditions Tested with a Mannequin and Human Subjects. Doctoral dissertation, West Virginia University. 2020. Available from: <https://researchrepository.wvu.edu/etd/7986/>. doi: 10.33915/etd.7986.

.91 Hondjeu ARM, Ng WC, Anwari V, Xiao MZ, Rozenberg D, Kazlovich K, et al. A reusable, locally manufactured, half-face respirator provides better protection than fitted disposable N95 masks: development and quantitative fit-testing comparison. Research Square [Preprint] 2021 [cited 2023 July 9] Available from: <https://wwwresearchgatenet/publication/351605094_A_reusable_locally_manufactured_half-_face_respirator_provides_better_protection_than_fitted_disposable_N95_masks_development_and_quantitative_fit-testing_comparison>. doi: 10.21203/rs.3.rs-456096/v1.

.92 Ballard DH, Jammalamadaka U, Meacham KW, Hoegger MJ, Burke BA, Morris JA, et al. Quantitative Fit Tested N95 Respirator-Alternatives Generated With CT Imaging and 3D Printing: A Response to Potential Shortages During the COVID-19 Pandemic. Acad Radiol. 2021;28(2):158-65. doi: 10.1016%2Fj.acra.2020.11.005. PMID: 33257256

.93 Ballard DH, Dang AJ, Kumfer BM, Weisensee PB, Meacham JM, Scott AR, et al. Protection levels of N95-level respirator substitutes proposed during the COVID-19 pandemic: safety concerns and quantitative evaluation procedures. BMJ Open. 2021;11(9):e045557. doi: 10.1136/bmjopen-2020-045557. PMID: 34475144

.94 Duda S, Hartig S, Hagner K, Meyer L, Intriago PW, Meyer T, et al. Potential risks of a widespread use of 3D printing for the manufacturing of face masks during the severe acute respiratory syndrome coronavirus 2 pandemic. J 3D Print Med. 2020;4(3):135-47. doi: 10.2217%2F3dp-2020-0014.

.95 Imbrie-Moore AM, Park MH, Zhu Y, Paulsen MJ, Wang H, Woo YJ. Quadrupling the N95 Supply during the COVID-19 Crisis with an Innovative 3D-Printed Mask Adaptor. Healthcare (Basel). 2020;8(3). doi: 10.3390/healthcare8030225. PMID: 32717841

.96 Levine M, Levine L, Xun H, Mathew PJ, Singh D, Gerber A, et al. Face Off: 3D Printed Masks as a Cost-Effective and Reusable Alternative to N95 Respirators: A Feasibility Study. Am J Med. 2022;135(9):1109-15. doi: 10.1016/j.amjmed.2022.04.026. PMID: 35580720

.97 Liu DCY, Koo TH, Wong JKK, Wong YH, Fung KSC, Chan Y, et al. Adapting re-usable elastomeric respirators to utilise anaesthesia circuit filters using a 3D-printed adaptor - a potential alternative to address N95 shortages during the COVID-19 pandemic. Anesthesia. 2020;75(8):1022-7. doi: 10.1111/anae.15108. PMID: 32348561

.98 Manomaipiboon A, Pupipatpab S, Chomdee P, Boonyapatkul P, Trakarnvanich T. The new silicone N99 half-piece respirator, VJR-NMU N99: A novel and effective tool to prevent COVID-19. PLoS One. 2020;15(12). doi: 10.1371/journal.pone.0237206. PMID: 33382705

.99 Martelly E, Li C, Shimada K. Moldable Mask: A Reusable, Hot Water Moldable, Additively Manufactured Mask to Be Used as an N95 Alternative. Materials (Basel). 2021;14(22). doi: 10.3390/ma14227082. PMID: 34832483

.100 Meadwell J, Paxman-Clarke L, Terris D, Ford P. In search of a performing seal: Rethinking the design of tight-fitting respiratory protective equipment facepieces for users with facial hair. Saf Health Work. 2019;10(3):275-304. doi: 10.1016/j.shaw.2019.05.001. PMID: 31497325

.101 McLeod KER. An exploration of thermomechanical softwood pulp for N95 respiratory mask production. BSc. Thesis, Saint Mary’s University, Halifax, Nova Scotia. 2021. Available from: <https://library2.smu.ca/handle/01/29526?show=full>.

.102 Ng WCK, Mbadjeu Hondjeu AR, Syrett A, Caragata R, Rozenberg D, Xiao Z, et al. Subject validation of reusable N95 stop-gap filtering facepiece respirators in COVID-19 pandemic. PLoS One. 2020;15(11):e0242304. doi: 10.1371/journal.pone.0242304. PMID: 33186406

.103 Roche AD, McConnell AC, Donaldson K, Lawson A, Tan S, Toft K, et al. Personalised 3D printed respirators for healthcare workers during the COVID-19 pandemic. Front Med Technol. 2022;4:45. doi: 10.3389/fmedt.2022.963541. PMID: 35982716

.104 Chughtai AA, Seale H, Rawlinson WD, Kunasekaran M, Macintyre CR. Selection and use of respiratory protection by healthcare workers to protect from infectious diseases in hospital settings. Ann Work Expo Health. 2020;64(4):368-77. doi: 10.1093/annweh/wxaa020c. PMID: 32144412

.105 Germonpre P, Van Rompaey D, Balestra C. Evaluation of Protection Level, Respiratory Safety, and Practical Aspects of Commercially Available Snorkel Masks as Personal Protection Devices Against Aerosolized Contaminants and SARS-CoV2. Int J Environ Res Public Health. 2020;17(12). doi: 10.3390%2Fijerph17124347. PMID: 32575366

.106 Greig P, Carvalho C, El‐Boghdadly K, Ramessur S. Safety testing improvised COVID‐19 personal protective equipment based on a modified full‐face snorkel mask. Anaesthesia. 2020;75(7):970-1. doi: 10.1111/anae.15085. PMID: 32275770

.107 Greig PR, Bradshaw J, Carvalho C, Iwaszko L, Ramessur S, Schumacher J, et al. A crossover study assessing the protective efficacy of improvised snorkel-based improvised respirators. J Intensive Care Soc. 2022;23(3):359-61. doi: 10.1177/1751143721991056. PMID: 36033251

.108 Grinshpun SA, Yermakov M, Kano M. Evaluation of AccuFIT 9000: A Novel Apparatus for Quantitative Fit Testing of Particulate Respirators. Ann Work Expo Health. 2020;65(4):458-62. doi: 10.1093/annweh/wxaa116. PMID: 33345279

.109 Harmata W, Kamionek D. Rules for fitting filtering gas masks. Sci J Mil Univ Land Forces. 2022;54(2 ):179-95. doi: 10.5604/01.3001.0015.8971.

.110 Kechli MK, Lerman J, Ross MM. Modifying a Full-Face Snorkel Mask to Meet N95 Respirator Standards for Use With Coronavirus Disease 2019 Patients. AA Pract. 2020;14(7):e01237. doi: 10.12132FXAA.0000000000001237. PMID: 32539273

.111 Kroo L, Kothari A, Hannebelle M, Herring G, Pollina T, Chang R, et al. Modified full-face snorkel masks as reusable personal protective equipment for hospital personnel. PLoS One. 2021;16(1). doi: 10.1371/journal.pone.0244422. PMID: 33439902

.112 Nicholson K, Henke-Adams A, Henke DM, Kravitz AV, Gay HA. Modified full-face snorkel mask as COVID-19 personal protective equipment: Quantitative results. HardwareX. 2021;9:e00185. doi: 10.1016/j.ohx.2021.e00185. PMID: 33655089

.113 Persing AJ, Sietsema M, Farmer K, Peters TM. Comparing respirator laboratory protection factors measured with novel personal instruments to those from the PortaCount. J Occup Environ Hyg. 2021;18(2):65-71. doi: 10.1080/15459624.2020.1864152. PMID: 33406010

.114 Pettinger M, Momeni M, Michaud C, Van Dyck M, Kahn D, Lemaire G. Verification of two Alternative Do-it-yourself Equipment Respirators Seal as COVID-19 Protection (VADERS-CoV): a quality assessment pilot study. Acta Anaesth Belg. 2020;72(2):101-7. doi: 10.56126/72.2.7.

.115 Bergman MS, Zhuang Z, Xu SS, Rengasamy S, Lawrence RB, Boutin B, et al. Assessment of respirator fit capability test criteria for full-facepiece air-purifying respirators. J Occup Environ Hyg. 2019;16(7):489-97. doi: 10.1080/15459624.2019.1609006. PMID: 31107187

.116 Chehade AEH, Stephenson J, Floyd E, Keddissi J, Abdo T, Thind S, et al. Feasibility study: Proposed alternative to N95 respirator during the personal protective equipment shortage from COVID-19 pandemic. J Emerg Manag. 2021;19(7):193-202. doi: 10.5055/jem.0611. PMID: 34723379

.117 Han D-H, Seo H, Kang B-k, Jang H, Kim H, Shim S. Comparisons of Fit Factors Between Two Quantitative Fit Testers (PortaCount vs. MT). Saf Health Work. 2022;13(4):500-6. doi: 10.1016/j.shaw.2022.10.001. PMID: 36579005

.118 Rengasamy S, Zhuang Z, Lawrence RB, Boutin B, Yorio P, Horvatin M, et al. Evaluation of total inward leakage for NIOSH-approved elastomeric half-facepiece, full-facepiece, and powered air-purifying respirators using sodium chloride and corn oil aerosols. J Occup Environ Hyg. 2021;18(7):305-13. doi: 10.1080/15459624.2021.1919685. PMID: 34038318

.119 Sietsema M, Hamza H, Brosseau LMJJoO, Hygiene E. Simulated workplace protection factor study of a quarter facepiece elastomeric respirator. J Occup Environ Hyg. 2022;20(1):33-9. <https://doi.org/10.1080/15459624.2022.2145014>. PMID: 36416662

.120 Weng C-H, Kao C-L, Chiu P-W, Huang S-P, Kuo Y-S, Lin Y-Y, et al. A full-face mask for protection against respiratory infections. BioMedical Engineering OnLine. 2022;21(1):62. 10.1186/s12938-022-01027-1.

.121 Clinkard D, Mashari A, Karkouti K, Fedorko L. Evaluation of N95 respirators, modified snorkel masks and low-cost powered air-purifying respirators: a prospective observational cohort study in healthcare workers. Anaesthesia. 2021. doi: 10.1111/anae.15392. PMID: 33470422

.122 Convissar D, Berra L, Chang MG, Bittner EA. Personal Protective Equipment N95 Facemask Shortage Quick Fix: The Modified Airway From VEntilatoR Circuit (MAVerIC). Cureus. 2020;12(5). doi: 10.7759/cureus.7914. PMID: 32440384

.123 Toigo S, Jacques M, Razek T, Rajda E, Omelon S, Dankoff F, et al. Fit Testing Retrofitted Full-Face Snorkel Masks as a Form of Novel Personal Protective Equipment During the COVID-19 Pandemic. Disaster Med Public Health Prep. 2021:1-16. doi: 10.1017/dmp.2021.133. PMID: 33926606

.124 Cass HG, Hanlon GC, McKenzie DP, Harley NS, Kelly DN, Barrett JA. The adequacy of user seal checking for N95 respirators compared to formal fit testing: A multicentred observational study. Aust Crit Care. 2022. doi: 10.1016/j.aucc.2022.08.012. PMID: 36244917

.125 Baba H, Ando H, Ikegami K, Sekoguchi S, Shirasaka T, Ogami A. Comparison of respiratory protection during exercise tasks between different methods of wearing replaceable particulate respirators and powered air-purifying respirators. Ind Health. 2022:2021-0268. doi: 10.2486/indhealth.2021-0268. PMID: 35569997

.126 Grinshpun SA, Corey J, Yermakov M, Wu B, Strickland KT, Bergman M, et al. New respirator performance monitor (RePM) for powered air-purifying respirators. J Occup Environ Hyg. 2020;17(11-12):538-45. doi: 10.1080/15459624.2020.1814491. PMID: 32941118

.127 Kessel J, Saevig CS, Hill WC, Kessel B, Hull MS. An Emergency Powered Air-Purifying Respirator From Local Materials and its Efficacy Against Aerosolized Nanoparticles. Inquiry. 2022;59:469580221087837. doi: 10.1177/00469580221087837. PMID: 35341353

.128 McGrath BA, Shelton CL, Gardner A, Coleman R, Lynch J, Alexander PG, et al. Bubble-PAPR: a phase 1 clinical evaluation of the comfort and perception of a prototype powered air-purifying respirator for use by healthcare workers in an acute hospital setting. BMJ Open. 2023;13(5):e066524. doi: 10.1136/bmjopen-2022-066524. PMID: 37156585

.129 Nagel J, Gilbert C, Duchesne J. Novel 3D printable powered air purifying respirator for emergency use during PPE shortage of the COVID-19 pandemic: a study protocol and device safety analysis. BMJ Open. 2021;11(8):e049605. doi: 10.1136/bmjopen-2021-049605. PMID: 34446492

.130 Goto Y, Jingushi N, Hiraiwa H, Ogawa H, Sakai Y, Kasugai D, et al. The protective effect of tight-fitting powered air-purifying respirators during chest compressions. Am J Emerg Med. 2021;49:172-7. doi: 10.1016/j.ajem.2021.06.012. PMID: 34118785

.131 Ng I, Lee K, Kave B, Kluger M, Paynter C, Segal R, et al. HALO CleanSpace PAPR evaluation: Communication, respiratory protection, and usability. Infect Control Hosp Epidemiol. 2023;44(2):295-301. doi: 10.1017/ice.2022.71. PMID: 35361300

.132 Rees P, Watson S, Corcoran J, Slade D, Pathmanaban O, Bibi A, et al. Powered air-purifying respirators: a solution to shortage of FFP3 filtering facepiece respirators in the operating theatre. Br J Surg. 2021;108(4):e160-e1. doi: 10.1093/bjs/znab008. PMID: 33778849

.133 Sekoguchi S, Shirasaka T, Ando H, Ikegami K, Ogami A. Evaluation of the performance of replaceable particulate and powered air-purifying respirators considering non-recommended wearing methods. Ind Health. 2020;58(6). doi: 10.2486%2Findhealth.2020-0056. PMID: 32863380

.134 Sekoguchi S, Ando H, Ikegami K, Yoshitake H, Baba H, Ogami A. Measurement of the workplace protection factor of replaceable particulate and powered air-purifying respirators in Japanese dust-generating occupations. J UOEH. 2022;44(1):15-24. doi: 10.7888/juoeh.44.15. PMID: 35249937

.135 Temmesfeld MJ, Gorzkowska-Sobas AA, Hedlund K, Øyen MØ, Kanten L, Grant P, et al. Surgical helmets can be converted into efficient disinfectable powered air-purifying respirators. Am J Infect Control. 2022;50(6):624-30. doi: 10.1016/j.ajic.2021.12.002. PMID: 34958857

.136 Xu X, Zhao L, Zhu Y, Du B, Zhu B, Zhang H, et al. Conducting quantitative mask fit tests: application details and affecting factors. Front Public Health. 2023;11. doi: 10.3389/fpubh.2023.1218191.

.137 Rowlett JM. The Perceptions of the Quantitative and Qualitative Fit Testing Protocols and Relevance of the Clean-Shaven Guidance for the Current Field of Reusable Elastomeric Half-Mask Respirators. Doctoral dissertation. Indiana University of Pennsylvania. 2021. Available from: <https://www.proquest.com/openview/81c0bc1ae4ea1bc16767325e4240ec44/1?pq-origsite=gscholar&cbl=18750&diss=y>. .
