# Supplementary material for: A systematic review of passing fit testing of the masks and respirators used during the COVID-19 pandemic: Part 1-quantitative fit test procedures
Source: PLoS One. 2023 Oct 26;18(10):e0293129. doi: 10.1371/journal.pone.0293129 (PMC10602271; doi:10.1371/journal.pone.0293129)
Supplement: S5 Appendix — (DOCX) [file pone.0293129.s005.docx]

**Table 1. Quantitative fit testing of disposable masks respirators and affective factors**

| **Study** | **Respirator Features**  **(Brand, model, size, style)** | **Subject Characteristics** | **Country** | **Standard Fit Testing Procedure** | **Study Procedure** | **Findings** |
| --- | --- | --- | --- | --- | --- | --- |
| Brandel et al., 2020 [1] | DIY homemade mask | One male and one female mannequin heads | USA | OSHA ‎‎29CFR ‎‎1910.134  QNFT protocol, TSI PortaCount  Pro | A DIY mask was developed to combat the shortage of medical masks. | The developed mask minimized the air flow around the edge and had an FF≥2 (acceptable range: 1.5-2). |
| Buckley et al., 2020 [2] | HensNest face mask | A single test subject | USA | OSHA ‎‎29CFR ‎‎1910.134 QNFT protocol, TSI PortaCount  Pro+8038 | The fit testing of the novel mask was compared to the N95 respirator, surgical mask, sewn mask, and Bandana. | The FFs for the HensNest (HEPA, 1 ply): 8, HensNest (HEPA, 3 ply): 23, HensNest (Grocery Bag): 4, HensNest (Coffee Filter), Surgical mask, Sewn mask, and Bandana: 1, and HensNest (Tea Towel): 3, were low in comparison to those of the N95 respirator (83). |
| Coyle et al., 2021 [3] | Three-ply cotton mask | Source simulator and one subject | USA | OSHA ‎‎29CFR ‎‎1910.134 QNFT protocol, TSI PortaCount Pro+ | The fitting of the 3-ply cotton mask was compared to the 3M 1860 N95 respirator. | The FFs of the three-ply cloth mask were 4.1 ± 2.6 (n = 43) for the recipient and 1.7 ± 0.6 (n = 42) for the source simulator. |
| Dang et al., 2021 [4] | A novel sewn Glidden mask | Six volunteers | USA | OSHA 29 CFR 1910.134  QNFT protocol, TSI PortaCount  model 8048 | The fitting of three designs of sewn masks, including H600-Filti-H600, H600-MERV-H600, H600-Swiffer-H600 was evaluated. | Four out of six volunteers passed the Glidden mask; two failed subjects had FFs of 20 per H600-Filti-H600 (in three layers) and 98 per H600-H600 (in two layers). This mask demonstrated the intermediate protection between a surgical mask and N95 FFR. The mask size, material stiffness, and plasticity could influence the fitting. |

| **Study** | **Respirator Features**  **(Brand, model, size, style)** | **Subject Characteristics** | **Country** | **Standard Fit Testing Procedure** | **Study Procedure** | **Findings** |
| --- | --- | --- | --- | --- | --- | --- |
| Drouillard et al., 2022 [5] | Fifty-two cotton fabrics | Two testers (One male and one female) | Canada | Modified OSHA ‎‎29CFR ‎‎1910.134 &  CSA Z94.4–2002,  QNFT protocol, TSI PortaCount  model 8038 | The fitting of 16 constructed fabric masks was compared to the L1 medical mask. | The FFEs of the tested masks were significantly different (p*<*0.001). The mean control medical mask had an FFE of 55.3±2.1%. The best- performing fabric mask was WP036 (a bed sheet) with a mean FFE of 65.6±4.6%, followed by WP028 (tea towel; 65.0±1.4%) and WP047 (batik; 64.3±0.7%). The overall FF for all studied (fabric masks or medical masks) ranged from 1.6-3.0, and the FFE ranged from 39.81-65.57. |
| Duncan et al., 2021 [6] | Weddingstar & C2QC Multi-layer reusable cloth face masks, 2-layer, silk fabric and Quilt batting/cotton masks, Henan Liwei disposable procedure masks, MedSup & TAIDAKANG KN95 masks and 3M N95 respirators model 9210, Halyard Health FLUIDSHIELD 2, and North Safety Products model 7130N95, Gerson 2130, 3M™ 8110s, and 3M™ 1870 | 8-26 volunteers | Canada | CSA Z94.4-18  QNFT protocol, TSI PortaCount Pro+ model 8038 | The Total Inward Leakage Protection Performance (TILPF) was assessed for five group masks. | The GMD for the TILPF was measured for the N95 FFR: 165.7, for the KN95: 6.2, for the procedure mask: 2.26, for the multi-layer: 1.77, and for the fabric 2-layer: 1.42. |
| **Study** | **Respirator Features**  **(Brand, model, size, style)** | **Subject Characteristics** | **Country** | **Standard Fit Testing Procedure** | **Study Procedure** | **Findings** |
| Mueller et al., 2020 [7] | Sewn fabric facemasks and standard surgical masks | One subject | USA | OSHA 29 CFR 1910.134  QNFT protocol, TSI PortaCount  model 8028 | The fabricated and standard masks were assessed during the QNFT procedure. | The N95-1 mask had an FF of 126 (⁓99.2%) and the N95-2 had an FF of 10.6 (⁓90.6%). The FF for the surgical mask ranged from 57-67 (FFE: 50%–75%). The mean FF for the cloth surgical-style mask was 63, mean FFE: 58.6%, and for the fabric surgical-style mask was FF: 63.36, mean FFE: 57.84%. The mean FF for the fabric cone-shaped masks was 53.71, mean FFE: 86.20%, and also for the duck-bill-shaped mask, FF: 60.90, mean FFE: 64.2%. The nylone overlayer increased the mean particle removal efficiency. |
| Reutman et al., 2021 [8] | Homemade  facemask | 10 subjects (Six males, four females) | USA | OSHA ‎‎29CFR ‎‎1910.134  QNFT protocol, TSI PortaCount  Pro+ model 8048 | A three-layer facemask prototype, which was made from well-performing materials, was fit tested compared to the two commercial surgical masks. | The overall “adopted” fit factor (aFF) ranged from 1-27 for the prototyped mask (overall aFF: 7.1 ± 1.6), 2-196 for the mask A (overall aFF: 12.6 ± 4.4), and 1-9 for the mask B (overall aFF: 2.5±3.2). A significant difference was observed in overall aFF between subjects and within subjects. A highly significant difference was observed between the mask types (p < 0.001). |

| **Study** | **Respirator Features**  **(Brand, model, size, style)** | **Subject Characteristics** | **Country** | **Standard Fit Testing Procedure** | **Study Procedure** | **Findings** |
| --- | --- | --- | --- | --- | --- | --- |
| Teesing et al., 2020 [9] | 25 materials | Laboratory setup and one woman | Netherlands | OSHA ‎‎29CFR ‎‎1910.134 QNFT protocol, AccuFIT 9000 Respirator Fit Test apparatus | The FEs for the 25 combinations of materials were assessed. Also, an ordinary laboratory paper towel was used as a control. An IIR-surgical mask served as a reference. | The highest FF for the 3M FFP21862+ was 134, Duckbill with a seam on the inside (ePM₁ 85%) was 130, Duckbill with a seam on the outside (ePM₁ 85%) was 120, respectively.  All remaining fabrics had a lower FF range of 8-79. The duckbill fabric filter mask would provide a better fit than the surgical mask (FF:4). Two layers of quilt fabric with a household paper towel could be adequate for users’ protection. |
| Wentworth et al., 2020 [10] | Homemade masks | Laboratory set up | USA | OSHA ‎‎29CFR ‎‎1910.134  QNFT protocol, TSI PortaCount  Pro model 8038 | The TSI PortaCount was fitted with a test chamber to seal the material with a worm-drive clamp or threaded band.  The fitting of homemade masks was compared to the N95 mask. | N95 UV 2 Cycle Test#3, N95 UV 2 Cycle Test#4, N95 Plasma Test, N95 Plasma Test #2, N95 Plasma Test #1, Yellow Surgical Mask, Ntension Surgical Mask Prototype Double Layer/ Single Layer, Strainrite provided White or Grey PolyPro SB, and Ntension Prototype 2 Test had an FF of 200 as well as the N95 mask. |
| Lindsley et al., 2021 [11] | Procedure mask, cloth mask, neck gaiter, and face shield | Headform | USA | OSHA 29 CFR 1910.134  QNFT protocol, TSI PortaCount | The FFs for the procedure mask, cloth mask, neck gaiter, and face shield were compared to the N95 respirator. | The FFs for the procedure mask were 2.9 (0.5), for the cloth mask: 1.3 (0.1), for the Neck gaiter (single layer): 1.7 (0.5), for the Neck gaiter (double layer): 1.9 (0.4), and for the N95 respirator:198 (3.5). The N95 respirator outperformed compared to the remaining device (p< 0.0001). |
| **Study** | **Respirator Features**  **(Brand, model, size, style)** | **Subject Characteristics** | **Country** | **Standard Fit Testing Procedure** | **Study Procedure** | **Findings** |
| Sato et al., 2020 [12] | Four masks, including Japan Medical Products Co HopesVR face mask (JM-28C) Pleated-type nonactivated carbon mask, NisshoSangyo Co NS surgical mask (14732), KOKEN LTD Hirac（type 35）Pleated-type activated carbon mask, KOKEN LTD Hirac （type 350）cup-type nonactivated carbon mask, and KOKEN LTD MaskyMD cup-type activated carbon mask | Four pharmacists  (Two males, two females) | Japan | OSHA ‎‎29CFR ‎‎1910.134  QNFT protocol, Sibata quantitative fit tester model MT-03 | The leakage rate and particle reduction rate of four studied masks were evaluated. | The leakage rate and particle reduction rate were achieved: 14.8±10.5, 70.8±11.3, per the Pleated-type nonactivated carbon mask, 34.8±26.9, 48.5±28.4, per the Pleated-type activated carbon mask, 0.3±0.4, 99.3±0.7, per cup-shape nonactivated carbon mask, 5.6±19.4, 33.6±10.9, per the cup-shape activated carbon mask. The cup-shape respirators, particularly the activated carbon type, were the most effective. |
| Ardon-Dryer et al., 2021 [13] | HDX N95 respirator, AOXING and ARUN KN95 respirators, NANO KN95 respirator, 3D-printed Montana Mask equipped with MERV 13-AIRx, MERV 13-H, and HEPA filters | Manikin headform | USA | OSHA 29 CFR 1910.134  QNFT protocol, TSI PortaCount  model 8030 | The fitting characteristics of the studied all N95 and KN95 respirators were evaluated. | All N95 and KN95 respirators passed the fit tests. The Montana masks with any of the three filters failed the fit tests. Also, homemade duckbill masks made from the Halyard H600 sterilization wrap and WypAll X80 reusable wipe failed the fit tests. |
| **Study** | **Respirator Features**  **(Brand, model, size, style)** | **Subject Characteristics** | **Country** | **Standard Fit Testing Procedure** | **Study Procedure** | **Findings** |
| Bodas et al., 2022 [14] | Two flat-fold BYD DE2322 N95 and Care Essentials (CE)  MSK-002 P2  masks | 300 participants including Healthcare workers or employees (87 males, 205 females) | Australia | Modified OSHA ‎‎29CFR ‎‎1910.134 QNFT protocol,  TSI PortaCount model 8048 Pro^+^ | The FFs of the studied masks were compared with each other. | The fitting of the MSK-002 mask (57%) with an FF of ~200 was significantly higher than the BYD DE2322 mask (18%) with an FF of ~70, p <0.001. The overall subjective rating for the CE MSK-002 was significantly higher for the BYD (p<0.001). |
| Cameron et al., 2020 [15] | Five respirators including 3M 1860 and 1860S N95, ProShield TN01–11, TN01–12 N95 respirators, and 3M Aura 1870+ mask | 371 HCWs | Australia | OSHA 29CFR 1910.134  QNFT protocol, TSI PortaCount Pro^+^ model 8038 | The QNFT procedure was conducted using the TSI PortaCount Pro respirator fit tester on a total of 371 HCWs. | 23 (6.2%) failed the first four masks, and 6 (1.6%) failed all five masks. The 3M 1860S had the highest (18.2%) failure rate. |

| **Study** | **Respirator Features**  **(Brand, model, size, style)** | **Subject Characteristics** | **Country** | **Standard Fit Testing Procedure** | **Study Procedure** | **Findings** |
| --- | --- | --- | --- | --- | --- | --- |
| Chan et al., 2021 [16] | Seven types of P2/N95 respirators  (3M 8210, 3M 8110S, 3M 1860, 3M 1860S, Proshield N95 ‘duckbill’ respirator, Halyard N95 duckbill respirator (standard), Halyard N95 duckbill respirator (small) | 59 HCWs  (23 males, 36 females) | Australia | OSHA ‎‎29CFR ‎‎1910.134  QNFT protocol, TSI PortaCount  Pro+ model 8048 | The P2/N95 respirators’ seal checks were compared to the FFs obtained from the QNFT procedures. | The fit test failure rate was (69% (40/58)) for the first selection of N95/P2 respirators. The 3M^TM^ 1860 respirator had the highest passing rate (67%) and the Halyard small duckbill had the lowest passing rates (8%). Also, 69% of the respirators failed the QNFT. The seal checks could not detect the respirator fitting capability (PPV: 34.1%, 95%CI: 25.0-40.5). The sensitivity, specificity, positive predictive value, and negative predictive value for the seal check were as follows: Se: 77.8%, Sp: 32.5%, PPV: 34.1%, and NPV: 76.5%. The median [IQR] FFs were calculated: 3M 1860: 148 (50–200), 3M 8210: 89 (46–200), 3M 1860S: 69.5 (10–86), 3M 8110S: 61.5 (50–80), Halyard (small): 29 (18–124), Proshield: 21 (10–50), and Halyard (regular): 17 (7–36). Also, the subjects’ perception improved from 39.2% (20/51), pre-test (before fit testing), to 81.8% (36/44), post-test (after fit testing). |

| **Study** | **Respirator Features**  **(Brand, model, size, style)** | **Subject Characteristics** | **Country** | **Standard Fit Testing Procedure** | **Study Procedure** | **Findings** |
| --- | --- | --- | --- | --- | --- | --- |
| Christopher et al., 2021 [17] | 3M1860 N95 respirators (regular/small size) | 305 staff of  health services centre  (110 males,  195 females) | USA | OSHA ‎‎29CFR ‎‎1910.134  QNFT protocol, TSI PortaCount  Pro+ model 8038 | The fit testing of the N95 respirator was compared between males and females. | Failure rates of fit testing in females were significantly higher than those in males due to being small-boned (6.67% vs. 2.72%; p<0.0001). The reasons for the fit test failure of females were considered mainly due to small bone structure and for males due to facial hair. |
| Cloet et al., 2022 [18] | Three masks, including MNmask v1 (small, medium, large), MNmask v2 (small, medium, large), and KN95 respirator | Nine female dental students | USA | OSHA ‎‎29CFR ‎‎1910.134 QNFT protocol, TSI PortaCount  Pro+ model 8038 | The FFs for the produced masks were compared to the KN95 mask. | The FFs were obtained for the MNmask v1: 93.3, the MNmask v2: 438.0, and the KN95 mask: 4.9. The results of the activity and usability assessment indicated that the KN95 had the highest usability score due to its loose-fitting.  MNmask v2 had higher usability scores (subjective discomfort, wear efficiency, and speech intelligibility), and breathability than the MNmask v1; however, it obtained lower stability. Because the paracord bands in MNmask v2 resulted in a higher wear efficiency, but lower stability score. |

| **Study** | **Respirator Features**  **(Brand, model, size, style)** | **Subject Characteristics** | **Country** | **Standard Fit Testing Procedure** | **Study Procedure** | **Findings** |
| --- | --- | --- | --- | --- | --- | --- |
| Cloet et al., 2022 [19] | Three N95 FFRs, including MNmask v1, MNmask v2, and KN95 respirator | Nine female Dental students | USA | OSHA ‎‎29CFR ‎‎1910.134 QNFT protocol, PortaCount Pro + model 8038 | The fitting of the studied respirators was examined. | The passing rates and mean FFs were obtained for the MNmask v1: 22.22%, 93.32+141.35, MNmask v2: 77.78%, 438.0+436.15, KN95: 0%, 4.86±2.15. The subjects scored the MNmask v1 as the most fitting (level of confidence in the mask seal) and the MNmask v2 as the most stable mask. Factors such as fit, comfort, material, and design are vital to solving the users’ challenges. The nose wire and nose discomfort, foam and chin and cheekbone discomfort, bands and head and neck discomfort, and filter material and skin discomfort (symptoms of rash, skin indentations, and itching) are crucial to take into account. |
| Griffin et al., 2022 [20] | Four novel masks (MNmask v1, MNmask v2, MNmask Reusable, and MNmask Procedural), three N95, and one KN95 masks | Nine participants (Four males, five females) | USA | OSHA ‎‎29CFR ‎‎1910.134  QNFT protocol, TSI PortaCount | Four mask designs, including MNmask v1, MNmask v2 (new version of MNmask v1), MNmask Reusable, and MNmask procedure, were developed.  The quantitative fitting of MNmask v1 and MNmask v2 was compared to one KN95 mask and three commercial masks, including GB2626-2019, N95 KCC 62355, N95 3M Aura 9210^+^, and N95 3M 1860. | None of the participants passed the KN95 GB2626-2019 and N95 KCC 62355 masks.  Eight of nine (88.89%) participants passed the N95 3M Aura 9210^+^ mask (mean ± S.D FF: 220.9±169.2), seven participants (77.78%) passed the MNmask v2 (mean ± S.D FF: 438.0±436.1). Five participants (55.56%) passed the N95 3M 1860 mask (mean ± S.D FF: 89.6±45.4). Two participants (22.22%) passed the MNmask v1 mask (mean ± S.D FF: 93.3 ±141.3). |
| **Study** | **Respirator Features**  **(Brand, model, size, style)** | **Subject Characteristics** | **Country** | **Standard Fit Testing Procedure** | **Study Procedure** | **Findings** |
| Duncan et al., 2020 [21] | Surgical-style 3M 1870 N95 FFR | Eight subjects | Canada | CSA. Z94.4-18-2018  QNFT protocol, TSI PortaCount Pro^+^ model 8038 | The seven subjects reused the mask for up to 5 days, up to 4 times per day, and the SWPFs were measured while walking and moving between floors in a building by stairs, paper and computer work-related activities, and task-based activities involving bending, kneeling, reaching, and lifting (60–80 min per each activity), and subject 8 was the control (wore the mask for 1.5 hr). Also, the general protection factor (GRPF) or total inward aerosol leakage for the subjects was measured before and after the trial to ensure the provision of respiratory protection. | The initial GRPF and the end-of-day GRPF ranged from 100–426 and 13–169, respectively.  The SWPFs for all the test subjects ranged from 10–84. On a day-to-day basis, as the number of reuses increased, the GRPF increased or decreased relative to the day prior. The GRPF for all test subjects was less than the initial GRPF after 18–19 wears on day 5 (p<0.05). |
| Fabre et al., 2021 [22] | 3M 1860 N95 FFRs (Dome shaped) and 3M1870 N95 FFRs (Duck-bill shaped) | 92 Physicians/ Advanced practitioners  (15 males, 77 females) | USA | OSHA ‎‎29CFR ‎‎1910.134  QNFT protocol, TSI PortaCount Plus 8028 | The effects of reuse of the N95 FFRs were examined using the quantitative and qualitative fit tests. | Five out of 16 N95 fit failures (31%) were identified by seal check. 18 N95 respirators failed one or more screening tests; 16 (89%) of them failed the PortaCount QNFT (overall failure rate: 17%). 83% of the N95s were effective, as they passed the fit test of the 3M N95s after a median of 40 donnings by the HCWs. |

| **Study** | **Respirator Features**  **(Brand, model, size, style)** | **Subject Characteristics** | **Country** | **Standard Fit Testing Procedure** | **Study Procedure** | **Findings** |
| --- | --- | --- | --- | --- | --- | --- |
| Nakamoto et al., 2021 [23] | Three N95 respirators, including  Duckbill-shaped HPR-R/HPR-S, dome-shaped Hi-Luck 350, and three-panel flat-fold 9211 respirators | 41 participants,  including 24 doctors and 17 nurses (16 males, 25 females) | Japan | OSHA ‎‎29CFR ‎‎1910.134  QNFT protocol, Sibata quantitative fit tester model MT-03 | The impact of N95 respirator reuse on fit testing was assessed. Fit testing procedures were utilized weekly up to three weeks. | The overall fit testing passing rate for reusing three styles of N95 respirators was 35 (85.4%). There were no significant differences among the studied N95 respirators.  The fit test passing rate was constant after the first week of reuse (fit test 1): overall: 41 (100), Duckbill-shape: 23 (100), cup-shaped: 10 (100), and Three-panel flat-fold: 8 (100). The fit passing rates after second week reuse (fit test 2): overall: 37 (90), Duckbill-shape: 21 (91), cup-shaped: 10 (100), Three-panel flat-fold: 6 (75)), and after third week reuse (fit test 3): overall: 35 (85), Duckbill-shape: 19 (82), cup-shaped:10 (100), and Three-panel flat-fold: 6 (75). |
| Greenawald et al., 2021 [24] | Five FFRs, including  3M 1870 N95, 3M 8210 N95, 3M 9010 N95, 3M 1860 N95 surgical mask, and  KIMBERLY-CLARK N95 (KC) 46827 Surgical Mask | Twenty-five volunteers | USA | OSHA ‎‎29CFR ‎‎1910.134 QNFT protocol,  TSI PortaCount Pro+ model 8038 | The FFs of the studied stockpiled respirators were compared to those of control respirators. | The FFs of both 3M 1870 and 3M 9010 respirators and each stockpiled Lot were not significant. Higher proportions passed the 3M 1860 Lot C compared to the control (81% vs. 58%; p<0.04). The 3M 8210 Lot B had lower passing rates than the control (32% vs. 76%; p<0.002) and lower than the control (74.44 vs. 127.13, p<0.001). The passing rates of KC 46827 Lots A, B, and C were lower than the control (9%, 8%, and 9% vs. 58%). |
| **Study** | **Respirator Features**  **(Brand, model, size, style)** | **Subject Characteristics** | **Country** | **Standard Fit Testing Procedure** | **Study Procedure** | **Findings** |
| Hai et al., 2022 [25] | Four FFRs including surgical mask, double mask (cloth mask on top normal surgical mask), N95 mask, New innovative stick-on mask, LEKAD. | One subject  (Physiotherapy lecturer) | Malaysia | Modified OSHA ‎‎29CFR ‎‎1910.134 CNC QNFT protocol, TSI PortaCount | Four studied FFRs underwent the QNFT procedure. | Among all, the stick-on mask Lekad compared to the other FFRs, obtained a high FF≥200. The FF were measured for the N95 mask: 6.5, for the Double mask: 5.75, and for the normal surgical mask: 5. There were significant differences among the FFRs (p=0.012). |
| Han et al., 2021 [26] | Three brands of the N95 respirators  including 3M 8210 (free size), Halyard Health N9586727/86827 (medium size), and [Dobu 201 (medium size)](https://aiden.health/products/dobu-n95-respirator-mask-model-201?variant=42166801334484) | 183 HCWs, including nurses in intensive care units, emergency medical centers, and nationally designated isolation treatment beds  (Four males, 179 females) | Korea | OSHA ‎‎29CFR ‎‎1910.134 QNFT protocol, TSI PortaCount  Pro+ model 8038 | The fit test passing rates for the three respirators were examined. | The overall passing rate and FF for the 3M were 46 (50%), 82.95±69.38, Halyard Health were 16 (33.3%), 104.64±54.36, and Dobu were 1(2.3%), 17.64±20.52, (p<0.001). |
| Hwang et al., 2020 [27] | Three N95 FFRs, including 3M 1870+,  3M 1860, Kimberly  Clark 46727 | 44 HCWs, including medical doctor, nurse, emergency medical technician (15 males, 29 females) | Korea | OSHA ‎‎29CFR ‎‎1910.134 QNFT protocol, PortaCount Pro+ model 8038 | The FF of the HCWs while performing three chest compression sessions. After that, they underwent USCs using the same respirator and performed the 4^th^ compression in order to assess the influence of chest compression on respirator slipping down. | All subjects passed the fit test. 32 subjects (73%) failed the fit test for at least one of the three chest compressions. Also, a significant difference was noted between the PPG and the APG (94% vs. 61%, p=0.02). Also, 8 (18%) of the subjects experienced strap loosening (5 per PPG vs. 3 APG, p=0.09). The fit test failures after the USCs were not significantly different (10 per PPG vs. 16 per APG, p= 0.73). |
| **Study** | **Respirator Features**  **(Brand, model, size, style)** | **Subject Characteristics** | **Country** | **Standard Fit Testing Procedure** | **Study Procedure** | **Findings** |
| Fakherpour et al., 2021 [28] | 20 models of FFRs (KN95, N95, N99, FFP2, and FFP3) | 37 volunteers (12 males, 25 females) | Iran | OSHA ‎‎29CFR ‎‎1910.134 QNFT protocol,  TSI PortaCount  Pro+ model 8038 | The quantitative fit testing procedure was performed on a total of 20 models of studied FFRs using the TSI PortaCount fit tester. | Eleven out of 20 FFRs had a passing rate lower than 10%. The highest proportions of passing the fit test were 43% (Uvex-Silv Air 2200) and 27% (3M8514 N95 and Termeh PAG3711 N99/FFP3), respectively. A significant difference was observed among the studied FFRs by mean FFs (p < 0.001). Cup-shaped respirators achieved a higher FF than the flat-fold ones (48 vs. 30, p < 0.001). |
| Jankusol et al., 2023 [29] | A duckbill-shaped N95 respirator | 34 Physicians (20 males, 14 females) | Thailand | OSHA ‎‎29CFR ‎‎1910.134 QNFT protocol, TSI PortaCount model 8048 Pro+ | The FFs of the N95 respirator during Endotracheal tube (ET) intubation were compared between video laryngoscopy (VL) and direct laryngoscopy (DL). | The lowest FF for VL was higher than DL (168 vs. 88, p=0.048). There was no significant difference between the ET intubation with VL vs. with DL (88.2% vs. 67.6%, p=0.065). The angle of head-up and hip bending with VL was lower than with DL (0° vs. 10°, p<0.001, 0° vs. 32°, p<0.001). The ET intubation with VL achieved a higher fitting than with DL due to the physicians’ requirement for lower movement. |

| **Study** | **Respirator Features**  **(Brand, model, size, style)** | **Subject Characteristics** | **Country** | **Standard Fit Testing Procedure** | **Study Procedure** | **Findings** |
| --- | --- | --- | --- | --- | --- | --- |
| Joshi et al., 2021 [30] | Commercial N-95 respirator, surgical mask, and cloth  mask | ND | India | OSHA 29 CFR 1910.134  QNFT protocols, TSI PortaCount Pro^+^ model 8038 & condensation particle counter (CPC) Grimm CPC (model no. 5416 | The FFs for the three types of masks were assessed during the QNFT CNC and CPC procedures. | The FFs for the face fix position were 28.86±5.37 using the Portacount and 30.43±7.43 using the CPC. The FFs for the sealed position were 61.43±17.46 using the Portacount and 58.95±13.89 using the CPC. |
| Jean-Romain et al., 2021 [31] | 151 FFRs | Three  volunteers (Two males, one female) | Switzerland | OSHA 29CFR 1910.134  QNFT protocol, TSI PortaCount Pro+ model 8038 | The fit testing of the studied FFRs was evaluated using the PortaCount Pro by two laboratories called Spiez Laboratory and Unisanté -TOXpro SA. | 55% of the tested products failed the fit test. |
| Jung et al., 2021 [32] | 3M 1870 N95 respirators | 10 Asian female infection control practitioners | South Korea | OSHA ‎‎29CFR ‎‎1910.134 QNFT protocol,  TSI PortaCount Pro^+^ model 8038 | The failure rate of N95 respirator was determined according to the number of donning/ doffing and the donning time (hr). | 60%, 70%, and 90% of the subjects failed the fit test after 2, 3, and 4 successive donnings per one-hour donning. 50% of fit testing failures occurred after a single use of one hour and 30% after a single use of two hours. |
| Kamal et al., 2023 [33] | Proshield® TN01-11 duckbill N95 respirator | 60 volunteer HCWs (Nine males, 51 females) | Australia | OSHA ‎‎29CFR ‎‎1910.134 QNFT protocol, TSI PortaCount Pro+ model 8048 | The fitting passing rate was assessed while donning a 3M Fahrenheit antifog safety goggle and N95 respirator and compared to the N95 respirator alone. | The fitting passing rate increased from 13.3% to 81.7%. The mean FF increased from 40.3 to 193.0, (p<0.001). The OR significantly increased after the application of the safety goggle (OR: 42, 95% CI: 7.14–1697.9, p < 0.0001). |
| **Study** | **Respirator Features**  **(Brand, model, size, style)** | **Subject Characteristics** | **Country** | **Standard Fit Testing Procedure** | **Study Procedure** | **Findings** |
| Kyaw et al., 2021 [34] | Three respirators, including three-panel flat-fold 3M 1870 flat-fold respirator, cup-shaped 3M 1860 respirator, and Duckbilled ProShield respirator | Seventy HCWs (22 males, 48 females) | Australia | OSHA 29 CFR 1910.134  QNFT protocol, TSI PortaCount Pro model 8048 | The effects of eyeglasses’ fogging on respirator fitting were examined. | 44 (63%) of the subjects experienced fogging, and 35 (70%) failed the fit test. The OR for fogging of eyeglasses to determine poor fitting was 2.10 (95% CI: 0.78–5.67, p=0.22). Also, fogging had low sensitivity (71%) and low specificity (46%). The fogging of eyeglasses had an AUC- ROC of 0.59. |
| Landry et al., 2022 [35] | OBE Premium surgical mask & 3M Aura 1870A N95 respirators | One male HCW | Australia | OSHA ‎‎29CFR ‎‎1910.134 QNFT protocol, TSI PortaCount model 8048  Fit Tester | The effects of fit test _PASSED_  and fit test _FAILED_ N95 respirators with and without PPE wearing (mask, gloves, gown, face shield) on exposure to Virus aerosol  was compared to a control. | Only the fit-test _PASSED_ N95 respirator resulted in lower virus counts compared to the control. The HEPA filter, when combined with the fit test _PASSED_ N95 mask, could protect against exposure to high virus loads. |
| Lindsley et al., 2021 [36] | Nineteen masks/ respirators including  two N95 respirators, two medical masks, and 15 reusable cloth masks (face masks, neck gaiters, and bandanas) | Elastomeric manikin headforms  & three human subjects | USA | OSHA 29 CFR 1910.134  QNFT protocol, TSI PortaCount  model 8038 | The FFs of the studied masks and respirators were examined by the modified QNFT procedure. | The 3M 1860 N95 respirator had an FF of 163.8 and the BYD N95 respirator had an FF of 147.9 on human subjects per N95 mode and were 45.3 and 54.9 per all particle sizes, respectively. The FF of the 3M 1818 surgical mask was 78.6 on human subjects. The Manikin fit factors per coughing for the 3M 1860 N95, BYD N95 respirators, and 3M 1818 surgical mask were 198, 25, and 26.1, and per the exhalation, they were 132.5, 113.5, and 27.5, respectively. |

| **Study** | **Respirator Features**  **(Brand, model, size, style)** | **Subject Characteristics** | **Country** | **Standard Fit Testing Procedure** | **Study Procedure** | **Findings** |
| --- | --- | --- | --- | --- | --- | --- |
| Long et al., 2022 [37] | Masks with healthcare (N95 model 3M 1870 and 1860 N95 respirators, HY8510,  H500, H100, Abdominal Pad, Surgical Mask, Sterilization Box Filter, Pediatric Drape, Bair Cover, Surgical Gown, Chux, Shoe Cover, and Mayo Stand Cover) and consumer material (Vacuum Bag, HVAC Filter, Smart Fab, Interfacing, Lawn Fabric, Shopping Bag, Paper Towel, Pillowcase, T shirt, Cotton, Chemex (coffee filter) | Laboratory setup | USA | OSHA ‎‎29CFR ‎‎1910.134 QNFT protocol, PortaCount Pro + model 8038 | The FE for the hospital-grade materials (healthcare-grade) and consumer-grade material (public) were assessed. | The N95 3M 1870 and 1860 N95 respirators had the highest FE (99.43% and 98.89%, FF: 175.44, 90.09, respectively). The FEs for the medical-grade materials ranged from 30 to 86% (FF: 1.43-7.14).  H500, Halyard corresponded to the highest FEs of 87% of all medical grade-materials (FF: 7.69). The FEs for consumer grade materials ranged from 35-53% (FF: 1.54-2.13). The FE for the vacuum bag was higher than that of the coffee filter and cotton cloth (82% vs. ~30% and 20%, FF: 5.55 vs. 1.43 and 1.25). |

| **Study** | **Respirator Features**  **(Brand, model, size, style)** | **Subject Characteristics** | **Country** | **Standard Fit Testing Procedure** | **Study Procedure** | **Findings** |
| --- | --- | --- | --- | --- | --- | --- |
| Milosevic et al., 2021 [38] | Eight N95 Eight N95 respirators (cup-shaped 3M 1860, cup-shaped 3M 1860S, flat-fold 3M 1870+, cup-shaped 3M 8110S, cup-shaped 3M 8210, flat-fold BYD DE2322, duckbill BSN TN01-11, and duckbill BSN TN01-12) | 6287 HCWs  (2089 males, 4198 females) | Australia | OSHA 29 CFR 1910.134  QNFT protocol, TSI PortaCount Pro+ model 8038 | The effects of respirator model, age, and gender of the HCWs on the results of fit testing were examined. | 93.3% passed the fit test. 57% passed the first FFR, 21% and 14%, and 9% of the participants passed the 2 and 3, 4 or more models of FFRs, respectively. Among all, the cup-shaped 3M 1860S respirator had the highest OR for passing the fit test (2.22, 95% CI, 1.94-2.54). The passing rates for the participants in the age group 18-29 were significantly higher than those with ages ranging from 30 to 59 (58.9% vs. 53.56%). The OR for passing rate for males was lower than for females (48.1% vs. 59.9%, OR: 0.85 vs. 1, p <0.001). |
| Ng et al., 2022 [39] | Four N95 respirators, including: semi-rigid cup 3M 1860 or 1860S, flat-fold cup BYD Care DE2322, duckbill BSN Medical ProShield or Fluidshield surgical masks Halyard, and 3M Aura 9320A+ three-panel flat-fold types | 2161 HCWs, including medical practitioner, nursing, allied health medical imaging, other health care worker, non-clinical employee, pharmacist, dental professional  (532 males, 1586 females) | Australia | OSHA ‎‎29CFR ‎‎1910.134 QNFT protocol  TSI PortaCount Pro+ 8048 fit tester | The subjects underwent at least three of four styles of N95 respirators. | The passing rates for the semirigid cup respirators (65.0%), flat-fold respirators (32.4%), for the duckbill respirators (59.2%), and three-panel flat-fold respirators (96.4%) were obtained. The three-panel flat-fold respirators had the highest comfort and usability values and the semi-rigid cup respirators had the lowest comfort and usability values. |
| **Study** | **Respirator Features**  **(Brand, model, size, style)** | **Subject Characteristics** | **Country** | **Standard Fit Testing Procedure** | **Study Procedure** | **Findings** |
| O’Kelly et al., 2021 [40] | N95 respirator,  surgical and two fabric face masks | One participant | USA | OSHA 29 CFR 1910.134  QNFT protocol, TSI PortaCount Pro+ model 8038 | Twenty-five tests were carried out on a participant using various masks and respirators. | The filtration efficiency of the N95 respirator, surgical mask, and two fabric face masks against the fine particles was 99.6%, 78.2%, and 62.6%-87.1%, respectively. Their FFs were 250, 4.59, and 2.68-7.75, respectively. |
| O’Kelly et al. 2021 [41] | Five N95 respirators, including 3M N95 8511, 3M 8200, Aero Pro AP0028, Makrite 9500, Xiantao Zong ZYB-11, one Zhong Jian Le KN95 respirator, one surgical mask, and five fabric masks, including a cotton bandana, a double-layered surgical-style pleated mask, a mask made of stretch material, and two masks designed to contour to the face | Seven participants | USA | OSHA 29CFR 1910.134  QNFT protocol,  PortaCount Pro+ model 8038 | The fit checks and QNFT procedures were conducted on the participants using the seven studied respirators and masks. | The N95 respirators provided more protection than the others. Three out of seven subjects passed the fit testing of the 3M model 8511 N95, and two subjects passed the 3M 8200 N95 respirator (mean FF: 72.3). All subjects failed the Xiantao Zong respirator and Aero Pro respirator (mean FF: 13.2 and 35.5, respectively). One passed the Makrite respirator (FF: 37.7). The KN95 respirator, fabric masks, and surgical mask had a mean FF of 2.2, 2.2, and 3.2, respectively. There was a poor correlation between the fit checks and QNFF values.  The mean fit of respirators which were considered to fit with a low degree of confidence was 138.5, with a medium degree of confidence was 74.1, with a high confidence was 75.5. |

| **Study** | **Respirator Features**  **(Brand, model, size, style)** | **Subject Characteristics** | **Country** | **Standard Fit Testing Procedure** | **Study Procedure** | **Findings** |
| --- | --- | --- | --- | --- | --- | --- |
| O’Kelly et al., 2022 [42] | 3M 8511 and 3M 8200 N95 respirators | Two participants (One male, one female) | USA | OSHA 29 CFR 1910.134  QNFT protocol, TSI PortaCount Pro model 8038 | The size of the required to compromise the fit of an N95 respirator was measured by two methods, including resin spacers and steel tubes. | The experiments indicated that as the FF increased, the gap size decreased. The minimum gap size to compromise N95 performance was about 1.5-3 mm^2^. The gap sizes of 0.4 mm and 0.8 mm had no impact on FF, while a gap size of 1.4 mm led to decrease in FF by a factor of 2.0, and a 2.9-mm gap decreased FF by approximately a factor of 4.0. A gap size of 1.4 mm or higher resulted in an FF of 23.9. |
| Park et al., 2021 [43] | Five 3M N95 respirators (Ever Green C250, DOBU LIFE TECH 201, DOBU LIFE TECH 500, 3M1860, and 3M 9210+) and six KF94 medical masks (3 horizontal and 3 vertical folding types; two large and one medium-sizes) | 30 HCWs, including nurses and doctors  (14 males, 16 females) | Korea | Modified  OSHA ‎‎29CFR ‎‎1910.134 QNFT protocol,  TSI PortaCount model 8048 Pro^+^ & Sibata fit tester model MT-03 | The passing rates of the N95 respirators against the KF masks were assessed using the QNFT procedure. The adequate protection rates were obtained before and after ear strap fixation. | The N95 respirators had a higher overall adequate protection rate (pass rate) by FFs than the KF masks (48.7% vs. 1.1%, 94.0 vs. 4.0, OR: 84.4, p<0.001, respectively) and by leakage rate (42.0% vs. 2.8%). Also, the passing rates and FFs for the 3M N95 respirators were higher than for the Korean (domestic) masks (25.6% vs. 83.3%, 38.5 vs. 200, OR: 25.3, p<0.001, respectively). Face length and age were significantly associated with adequate protection. |

| **Study** | **Respirator Features**  **(Brand, model, size, style)** | **Subject Characteristics** | **Country** | **Standard Fit Testing Procedure** | **Study Procedure** | **Findings** |
| --- | --- | --- | --- | --- | --- | --- |
| Popov et al., 2022 [44] | Five type respirators, including 3M EHR 7502, 3M particulate respirator 8511 N95, Particulate respirator KN95, Surgical mask, and Cotton mask | Three Caucasian male volunteers | USA | Modified OSHA ‎‎29CFR ‎‎1910.134 QNFT protocol, TSI PortaCount  Pro+ model 8020 | The studied respirators were quantitatively tested over time. | The N95 respirator passed the fit test (FF>100). But the KN95 had a low FF> 4 or 7 after multiple tests. The FFs were for the 3M EHR 7502: 460–660, 3M 8511 N95: 113–211, KN95 respirator: 24–57, surgical mask: 2–4, and cotton mask: 2–7. The extensive head and body movements could affect the respirator adjustment. |
| Regli et al., 2022 [45] | BSN Medical Proshield N95 respirator model TN01 (TN01-11 medium or TN01-12 small) | Forty-four  HCWs | Australia | OSHA ‎‎29CFR ‎‎1910.134 QNFT protocol, TSI PortaCount pro model TSI 8038 & modified QNFT protocol, TSI PortaCount Pro model 8048 | The modified fast QNFT was compared to the standard QNFT procedure and assessed. | The fit test rates for modified and standard QNFT procedures were 74% and 42%, respectively. The modified fast QNFT had a low sensitivity of 50%, a TP of 26%, a TN of 47%, and a FN of 26% compared to the standard QNFT procedure. |
| Regli et al., 2021 [46] | ProshieldVR N95 respirators (small TN01-12 and medium TN01-11) & 3M 9322A^+^ P2 N95 respirator | 84 staff from the Department of Anaesthesia and Pain Medicine including 53 predominantly  anaesthetists and 31 predominantly  anaesthetic technicians (40 males, 44 females) | Australia | OSHA ‎‎29CFR ‎‎1910.134 QNFT protocol, TSI PortaCount  Pro+ model 8038 | The participants underwent fit testing procedures using the N95/FFP2. | The first passing rate was 47% (34 out of 72), and the overall fit pass rate was approximately 79% (63 out of 80). Different mask types and sizes resulted in higher fit test pass rates. The QNFT had a higher pass rate than the QLFT per N95 respirator not used (74 vs. 59, p<0.006). The QNFT and QLFT had a low significant agreement (k=0.32). |
| **Study** | **Respirator Features**  **(Brand, model, size, style)** | **Subject Characteristics** | **Country** | **Standard Fit Testing Procedure** | **Study Procedure** | **Findings** |
| Prince et al., 2021 [47] | Five commonly protective face masks including 3M N95 respirator model 8210, Dr Puri KF94 supplied with ear loops and clip, Lei Shi De KN95, Medline Industries ear loop procedure masks, Hanesbrands reusable 3-ply 100% cotton fabric masks | Ten adult male staff members (Five with full facial hair, and five with no facial hair) | USA | OSHA ‎‎29CFR ‎‎1910.134 QNFT protocol, modified ambient aerosol CNC, condensation  particle counters (CPCs) TSI model 3775 | The FFE for the five commonly used face masks was assessed among bearded volunteers (beard length: 0-10 mm) using the TSI CPC model 3775. | The N95 respirator had the highest FFE compared to all the studied masks (85.3%, FF: 6.8). The FFEs for the KF94 and KN95 decreased to 61.9% and 54.9%, with FFs of 2.62 and 2.22, respectively. The FFEs for the procedure and cloth masks ranged from 30.6% to 39.4% (FF: 1.44 and 1.65). Also, the exercise band resulted in improving the FFE (96.1%, FF:25.64 for the N95 and 80.2%, FF: 5.05 for the KF94, 65.7%, FF: 2.91 for the KN95, 36.6%, FF: 1.58 for the Procedure mask, and 40.6%, FF: 1.68 for the Cloth/cotton mask, respectively). |
| Sandaradura et al., 2020 [48] | 3M Flat fold 1870 P2/N95 respirator | 105 male hospital employees (38 clean-shaven, 67 unclean- shaven) | Australia | OSHA ‎‎29CFR ‎‎1910.134 QNFT protocol, TSI PortaCount  Pro+ model 8038 | The effects of facial hair on respirator fit testing were assessed. | Approximately 32% passed the fit test, of which 47% were clean-shaven. Facial hair growth resulted in FF reduction. The OR for respirator fit was 0.74 (95% CI 0.21-2.52, P=0.08) for light stubble, 0.45 (95% CI 0.12-1.57, P=0.26) for moderate to heavy stubble, 0.04 (95% CI 0-0.28, p<0.001) for full beard, and 0.56 [95% CI 0.05-4.48, P=0.85] for other types of facial hair, compared to no facial hair. |

| **Study** | **Respirator Features**  **(Brand, model, size, style)** | **Subject Characteristics** | **Country** | **Standard Fit Testing Procedure** | **Study Procedure** | **Findings** |
| --- | --- | --- | --- | --- | --- | --- |
| De-Yñigo-Mojado et al., 2021 [49] | Surgical masks and FFP3 respirators (Moldex-2505, 3M Aura-9332+, and 3M K-113, Surgical masks (Shell type)) | 63 male HCWs (32 with facial hair, 31 without facial hair) | Spain | OSHA ‎‎29CFR ‎‎1910.134 QNFT protocol, TSI PortaCount model 8038 Pro+ model 8038 | The studied masks and respirators were examined using the QNFT procedure. | No significant difference was found between the bearded and non-bearded HCWs by the FFs of the studied surgical masks (2.37 ± 0.73 vs. 4.68 ± 7.52 p= 0.788). However, significant differences were found between the HCWs with and without facial hair by the FFs of FFP3 (30.59 ± 29.98 vs. 65.75 ± 37.58 p<0.01). |
| Sasko et al., 2023 [50] | N95, P2, and reusable respirators | HCWs | Australia | OSHA 29 CFR 1910.134  QNFT protocol | The fitting of the usual and additional respirators supplied was evaluated. | One reusable respirator failed the fit test. Of the 686 N95 and P2 respirators tested, 377 (55%) passed the fit test. But 22.3% failed at least one or more fit test exercises. 109 out of the 294 usual respirators supplied before the COVID-19 pandemic passed the fit test. 268 out of the 392 additional respirators supplied passed the fit test. |

| **Study** | **Respirator Features**  **(Brand, model, size, style)** | **Subject Characteristics** | **Country** | **Standard Fit Testing Procedure** | **Study Procedure** | **Findings** |
| --- | --- | --- | --- | --- | --- | --- |
| Seo et al., 2021 [51] | Two types of N95 filtering face-piece respirators (DOBU MASK 201 N95, Clean Top N95 C250) | 56 HCWs, including doctor, nurse paramedic, other medical technologists  (14 males, 42 females) | South Korea | OSHA 29 CFR 1910.134  QNFT protocol, TSI PortaCount Pro+ model 8038 | The effects of face sizes on the quantitative fit test were evaluated based on the NIOSH-bivariate panel. | The overall fit test pass rate was approximately 98.2%. The medium face size (51.8%), small face size (35.7%), and outlier group (10.7%) had the highest passing rate, respectively. No significant difference was found between the participants’ face sizes, whether they passed or failed the fit test (p<0.767). The GM±GSD FFs for the medium, small, and outlier categories were 25.72±2.41, 25.51±4.58, and 22.97±8.12, respectively. Females had significantly higher passing rates than males (41.1% vs. 10.7%; p=0.028). A significant difference was observed between the males’ and females’ face length (114.8 vs. 108.5 mm, p<0.05) and face width (142.7 vs. 134.1 mm, p<0.05). The face size distribution was significantly different between the NIOSH bivariate panel subjects and Korean HCWs (p=0.009). 10.7% of the subjects were outliers who did not place within the panel’s cells. |

| **Study** | **Respirator Features**  **(Brand, model, size, style)** | **Subject Characteristics** | **Country** | **Standard Fit Testing Procedure** | **Study Procedure** | **Findings** |
| --- | --- | --- | --- | --- | --- | --- |
| Seo et al., 2020 [52] | Four types of N95 masks | Thirty-five HCWs (14 males, 21 females) | Korea | Modified OSHA ‎‎29CFR ‎‎1910.134  QNFT protocol,  TSI PortaCount Plus model 8048 | The fitting four studied respirators (large size with/without a nose pad, medium size with/without nose pad) were compared. | The overall passing rate was 21%. There was no significant difference between the N95 respirators with/without a nose pad (25.6±23.1 vs. 29.1±47.6, p=0.1551. Also, no significant difference was found among the four types of respirators (p=0.4863). No significant difference was found between the males and females (29.2±38.6 vs. 26.1±36.8, p=0.9961). |
| Sheikh et al., 2022 [53] | 3M N95 1870+ respirator, Honeywell DC 365, 3M  1860, 3M 1860s, 3M 1804s, a 3M, 6000 reusable elastomeric half-facepiece respirator | 36 HCWs, including physician, nurse, respiratory therapist (Six males, 30 females) | USA | OSHA ‎‎29CFR ‎‎1910.134 QNFT protocol, TSI PortaCount (N95-Companion) | The fitting of the studied respirators was conducted. | 36 out of 41 (97.3%) HCWs passed the fit test. 23 of the 36 (63.9%) passed the first try, while the remaining 13 required more than one fit test. 27 (75%) HCWs were fitted to the 3M 1870+, 4 (11.2%) were fitted to the Honeywell DC 365, 3 (8.3%) were fitted to the 3M 1804s, and one subject (2.8%) was fitted to the 3M 1860s. The overall FF for non-White 175 (32) and White males; 200 (0), for non-White 165 (30), and for white 175 (31) females were obtained. The FFs for the males were higher than for the females (majority of the participants). 27 of the 36 (75%) HCWs were out of panel. |

| **Study** | **Respirator Features**  **(Brand, model, size, style)** | **Subject Characteristics** | **Country** | **Standard Fit Testing Procedure** | **Study Procedure** | **Findings** |
| --- | --- | --- | --- | --- | --- | --- |
| Sickbert Bennett et al., 2020 [54] | 3M 1860 N95 respirator, surgical mask with ties, procedure mask with ear loops | Two participants (one male, one female) | USA | OSHA 29 CFR 1910.134  QNFT protocol, TSI PortaCount Pro^+^ model 8038 | The fitted filtration efficiencies (FFE) of the studied masks were assessed during the QNFT procedure. | The FFE for the N95 respirator in the wrong size was not significantly decreased (90-95%, FF: 10-20). The FFEs for all non-approved respirators (n=6) were lower than 95% (FF: 20). The mean FFE of surgical masks with ties (71.5% (5.5%), FF: 3.45 (1.06)) and procedure masks with ear loops (38.1% (11.4%), FF: 1.62 (1.13)) was lower than that of the 3M 1860 N95 respirator (98.5% (0.4%), FF: 66.67 (1)). |
| Suen et al., 2022 [55] | Four N95 FFRs, including three traditional 3M FFR models 1860, 1860S and 1870+ and a nanofibre N95 FFR | 104 nursing students (21 males, 83 females) | Hong Kong | OSHA ‎‎29CFR ‎‎1910.134  QNFT protocol,  TSI PortaCount Plus | The FFs of 3M FFRs compared to the nanofibre N95 FFR before and after nursing procedures | 69 (66.3%) subjects passed the best-fitting 3M FFR, and 82 (78.8%) passed the nanofiber FFR. The best-fitting 3M FFR had a higher failure rate than the nanofibre FFR (33.7% vs. 21.2%, p= 0.417) after the procedures. The average FFs of both traditional and nanofibre FFRs decreased after performing nursing procedures (3M FFR: 185.08 vs. 135.52; nanofibre FFR: 188.44 vs. 149.13, p>0.05). The nanofibre FFR had significantly higher usability than the 3M FFRs (i.e., facial heat, breathability, facial pressure, speech intelligibility, itchiness, difficulty of maintaining the mask in place, comfort on the ear lobe, and overall comfort level), p<0.001. |
| **Study** | **Respirator Features**  **(Brand, model, size, style)** | **Subject Characteristics** | **Country** | **Standard Fit Testing Procedure** | **Study Procedure** | **Findings** |
| Goh et al., 2022 [56] | Two N95 respirators  with micro fan (MF) and AIR+ Smart Mask | 106 children (59 boys, 47 girls) | Singapore | OSHA ‎‎29CFR ‎‎1910.134 QNFT protocol,  TSI PortaCount Pro model 8038 | The FF of the studied masks was assessed while subjects ran on treadmills. | All subjects passed the fit tests. The respirators with or without MF were safe for children. The novel respirator could enhance the comfort and experience of wearing the mask. |
| Salter et al., 2021 [57] | Cloth masks (17 cotton batting masks), Moldex N95 respirator model 2212 (reference) | Laboratory set up | Canada | CSA Z94.4-18  QNFT protocol, Accufit9000 & PortaCount 8020 fit testers | The cotton bathing cloth masks were fit tested by three independent investigators. | There were not significant differences between FFs and filtration efficiencies for the cloth masks with and without the gaskets in the first set (90.4% vs. 90.0%, FF: 10.0 vs. 10.3).  In the second set, the average filtering effectiveness for the masks with gaskets was 77.3% (FF: 4.4). The average filtering effectiveness for the mask with and without a nylon layer over the mask included 76.5% vs. 83.7%, FF: 4.25 vs. 6.13, per third set. |
| [Vahabzadeh‐Hagh](https://scholar.google.com/citations?user=JOaLyxoAAAAJ&hl=en&oi=sra) et al., 2022 [58] | 3M 1860 N95  respirator | One patient | USA | OSHA ‎‎29CFR ‎‎1910.134 QNFT protocol, TSI PortaCount model 8020 | A novel mask was designed from polypropylene  filter material equipped with a nose-cone, cap, and silicone valve in order to perform endoscopy and protect against aerosol transmission. | The FE for the Polypropylene  sterilization wrap (97.31 ± 0.32, FF: 37.17) was similar to the 3M N95 1860 respirator (96.52%, FF: 28.73). |

| **Study** | **Respirator Features**  **(Brand, model, size, style)** | **Subject Characteristics** | **Country** | **Standard Fit Testing Procedure** | **Study Procedure** | **Findings** |
| --- | --- | --- | --- | --- | --- | --- |
| Vo et al., 2020 [59] | North N95 FFR model 7130N95 | Eight subjects (four males, four  females) | USA | OSHA ‎‎29CFR ‎‎1910.134 QNFT protocol,  TSI PortaCount Pro model 8038 | The subjects underwent fit testing. Then, SWPFs were measured using the two CPC, and two portable aerosol mobility spectrometers (PAMSs), compared to a set of two reference scanning mobility particle sizers (SMPSs). | All subjects passed the fit test. The SWPFs obtained from CPCs had a good agreement with SMPSs. The CPCs, PAMSs, and reference SMPSs had GM SWPF trends under similar simulated workplace activities. GM SWPF decreased with increasing simulated activities.  There were no significant differences between the GM overall SWPF of SMPS and CPC (at low concentration: 28.56 ± 1.07 vs. 23.16 ± 1.16, p=0.17, and at medium concentration: 36.93 ± 1.35 vs. 29.48 ± 1.41, p=0.23). |

| **Study** | **Respirator Features**  **(Brand, model, size, style)** | **Subject Characteristics** | **Country** | **Standard Fit Testing Procedure** | **Study Procedure** | **Findings** |
| --- | --- | --- | --- | --- | --- | --- |
| Vuma et al., 2021 [60] | N95 FFR (3M 1860 FFR) | 25 employees of the National Institute for Occupational Health (NIOH) (Nine males, 16 females) | South Africa | OSHA ‎‎29CFR ‎‎1910.134  QNFT protocol, TSI PortaCount Plus 8038 | Subjects donned the N95 FFRs six times. The FFs from the six times were compared with each other. | The median FFs were 195 (139–200) for the fit test 1, 161 (110–200) for the fit test 2, 167 (132–200) for the fit test 3, 124 (79–198) for the fit test 4, 168 (75–200) for the fit test 5, and 150 (72–192) for the fit test 6.  Two subjects (8%) had FF<100 fit test 2, 6 (24%) at fit test 3, 8 (32%) at tests 4, 5, and 6. Thirteen subjects (52%) had FF>100. There was a significant difference between the FFs of the first and sixth tests (195 vs. 150; p= 0.0271) but not between the second and sixth FFs (161 vs. 150; p= 0.3584). The FFs for the males and females were similar, except for the third and sixth donnings (p=0.041 and p=0.0015, respectively). Also, the overall FFs for infrequent users were higher than for frequent users. |

| **Study** | **Respirator Features**  **(Brand, model, size, style)** | **Subject Characteristics** | **Country** | **Standard Fit Testing Procedure** | **Study**  **Procedure** | **Findings** |
| --- | --- | --- | --- | --- | --- | --- |
| Williams et al., 2021 [61] | Two duckbill models of N95 FFRs, including Halyard FluidshieldVR N95 and the BSN Medical ProShieldVR N95 respirators | 96 anaesthetic staff, including Anaesthetic consultants and trainees (55 males and 41 females) | Australia | OSHA ‎‎29CFR ‎‎1910.134 QNFT protocol, TSI PortaCount  Pro+ model 8038 | The results of QNFT procedure and USCs of studied respirators were compared. | The passing rates for the Halyard Fluidshield (77%) and ProShieldVR (65%) were not statistically significant (p=0.916). The median IQR for the Halyard Fluidshield was 144 (102–196) and the ProShieldVR was 119 (29–200), p=0.09. Also, there were low agreements between the USCs and fit tests (0.16 for the Halyard Fluidshield, 0.08 for the ProShieldVR). The diagnostic tests showed PPV: 79.8%, NPV: 41.7% Sensitivity: 90.5% Specificity: 22.7% Overall accuracy: 75%, for the Halyard Fluidshield respirator and PPV: 66.7%, and NPV: 46.4% Sensitivity: 80.6% Specificity: 26.5% Overall accuracy: 61.5%, for the ProShield respirator. |
| Williams et al., 2021 [62] | Two types of three-panel flat-fold respirators including Trident^TM^ P2 FFR and 3M 9320A+ Aura three-panel flat-fold N95 FFR | 500 HCWs, including nursing, medical practitioner aged care/disability worker, allied health, medical imaging, other healthcare worker pharmacist, non-clinical role (122 males, 378 females) | Australia | AS/NZS 1715 &  Modified OSHA ‎‎29CFR ‎‎1910.134 QNFT protocol, TSI PortaCount Pro+ model 8048 | The comparison of the FFs of two studied FFR were made using the quantitative fit tester | The Trident^TM^ respirator had a significantly higher overall fit test passing rate (99.2% vs. 92.6%, p<0.001) and first-attempt passing rate (76.4% vs. 92.6%, p<0.001) than that of the 3M^TM^ Aura respirator. Also, the median (IQR) FFs for the Trident^TM^ were significantly higher than for the 3M^TM^ Aura (201 (201-201) vs. 201 (166-201), p<0.001). |
| **Study** | **Respirator Features**  **(Brand, model, size, style)** | **Subject Characteristics** | **Country** | **Standard Fit Testing Procedure** | **Study Procedure** | **Findings** |
| Williams et al., 2022 [63] | Two brands of 3M Aura 3M 9320A+ FFP2 and 3M 1870+ N95 surgical masks | 1000 participants from Royal Melbourne Hospital (332 males, 668 females) | Australia | Modified OSHA ‎‎29CFR ‎‎1910.134 QNFT protocol  TSI PortaCount Pro+ 8048 fit tester | The FFs of the studied respirators were compared. | The 3M 9320A+ had a significantly higher passing rate (94.6% vs. 91.7%, p<0.001) and FF (183±37.9 vs. 175.0±45.4, p<0.001) than the 3M 1870+ FFR. The overall passing rate was 89.2%. A fair agreement was observed between the passing rates of two FFRs (k=0.38).  Males had higher passing rates and FFs than females: 96.7% vs. 93.6%, p=0.04; 187.2±32.2 vs. 181.0±40.3, p=0.006, per 3M Aura 9320A+; 97.6% vs. 88.8%, p<0.001, 185.2±31.5; 170.0 vs. 50.2, per 3M Aura 1870+, p<0.001. |
| Williams et al., 2022 [64] | Halyard N95 FFR flat-fold duckbill respirator | 350 HCWs (81 males, 230 females) | Australia | Modified OSHA ‎‎29CFR ‎‎1910.134 QNFT protocol, TSI PortaCount  Pro 8048 | The performance of the commercial hand-hold technique compared with the manufacturer-recommended lanyard technique in stabilizing the sampling tube during the fit testing. | 72.2% of participants passed the fit testing using the handhold, and 52.0% passed using the lanyard technique (p<0.001). The overall FF for the Hand-hold technique, 167 (89-201) was higher than the Lanyard technique, 112 (52-196), p<0.001. A fair agreement was observed between the two techniques (k=0.39). The method of sampling tube stabilization during QNFT could lead to false negative fit testing results due to inadequate tube stabilization. |

| **Study** | **Respirator Features**  **(Brand, model, size, style)** | **Subject Characteristics** | **Country** | **Standard Fit Testing Procedure** | **Study Procedure** | **Findings** |
| --- | --- | --- | --- | --- | --- | --- |
| Lim et al., 2020 [65] | PNTD KF80 disposable particulate respirator | 20 older women participants | Korea | OSHA ‎‎29CFR ‎‎1910.134  QNFT protocol, Sibata quantitative fit tester model MT-03 | The leakage rates of the studied respirator were assessed at the 1st, 2nd, and 3rd tests. | The mean leakage rates in the first, second, and third tests were 73.6%, 71.5%, and 72.8%, respectively. The overall passing rate was 14.3%. Only 3 (14.3%), 4 (19%), and 6 (29%) of the participants passed the fit test (leakage test), respectively. The effects of particulate respirator use should also be considered via physiological stress marker elevation. |
| Mottay et al., 2020 [66]  ‎ | ‎Twelve KN95 ‎respirator brands ‎‎(A total of 36 ‎masks)‎ | Seven HCWs and laboratory workers  (One male, six females) | South Africa | OSHA ‎‎29CFR ‎‎1910.134 QNFT protocol, TSI PortaCount Pro+‎ model 8038 ‎ | All participants underwent USCs, QLFT, and then QNFT procedures, if passed the QLFT. The Halyard FLUID SHIELD 3 N95 and 3M 1860 N95 respirators, sterile surgical masks were used as controls. | 35 out of 36 masks failed the USCs. The KN95 respirators had lower passing proportions of the USC than that of the N95 respirators (1/36 (3%) vs. 12/12 (100%); p<0.0001). 15 out of 36 (42%) and 12 out of 12 (100%) passed the USCs of the KN95 respirators and N95 respirators, respectively using modification of ear-loop tension using head straps or staples or the face seal improvement using Micropore 3M tape. None of the respirators passed the QLFT, and then they did not proceed to the QNFT. |

| **Study** | **Respirator Features**  **(Brand, model, size, style)** | **Subject Characteristics** | **Country** | **Standard Fit Testing Procedure** | **Study Procedure** | **Findings** |
| --- | --- | --- | --- | --- | --- | --- |
| Zhang et al., 2020 [67] | Four models of N95 FFRs (two cup-shaped, two flat-fold styles) | 85 volunteers  (31 males, 54 females) | China | OSHA 29 CFR 1910.134  QNFT protocol, TSI PortaCount Pro+ model 8038 | The fitting of three NIOSH-approved N95 respirators and one EN-approved respirator was assessed. | The passing rates and GSD FF for four models were 52.9% (112.5±58.4), 61.2% (121.5±57.0), 40.0% (92.2±62.6), and 63.5% (121.0±58.7), respectively. A significant difference in passing rates among four models was found (p<0.05). Only 17 (20%) subjects passed the fit test of four models. There was a significant difference in passing rates for model 3 between males (54.8%) and females (31.5%). The passing rates and GM FFs for the flat-fold respirators (51.8%, 92.2; model 3 and 121; model 4) were lower than those for cup-shaped ones (57.1%, 121.5; model 1 and 121.5; model 2). There were significant differences between passed and failed subjects in face length, and nose height, nose length (p<0.05). |
| Boogaard et al., 2020 [68] | Three different types of locally-produced facemasks, including Reinier 0.1, DSM 1.0, and Reinier 1.0 | Three subjects | Netherlands | OSHA ‎‎29CFR ‎‎1910.134 & EN149 standard, QNFT protocol, TSI PortaCount  model 8030 | The IL of the studied face masks was assessed and compared to the 3M Aura 1862+ FFP2. | The min and max IL were obtained for the Reinier 0.1: 4.2, 4.8; for the DSM 1.0: 6.7%, 14.6%; and for the Reinier 1.0: 0.5%, 0.8%. The Reinier-0.1 and -1.0 models had acceptable Max IL <8%. Whereas, the DSM 1.0 did not meet the value (14.6%). |

| **Study** | **Respirator Features**  **(Brand, model, size, style)** | **Subject Characteristics** | **Country** | **Standard Fit Testing Procedure** | **Study Procedure** | **Findings** |
| --- | --- | --- | --- | --- | --- | --- |
| Carvalho et al., 2021 [69] | EN149:2001  approved-N99 and FFP3 respirators | 1443 HCWs  (included): 1182 HCWs (365 males, 817 females) | UK | HSE 282/28, QNFT protocol, TSI PortaCount model 8030 | The participants underwent the QNFT procedure. | Males were better fitted to the respirators than females (mean first-attempt passing rate: 51.46% vs. 42.66%, adjusted OR: 2.07, 95%CI (1.66-2.60) p<0.001). Among the various ethnic groups, White staff were better fitted than other participants (p<0.001). The Whites had a significantly higher pass rate than non-Whites (48.7%). |
| Caggiari et al., 2023 [70] | FFP3 respirator | 9592 HCWs (2009 males, 7583 females) | UK | OSHA ‎‎29CFR ‎‎1910.134 QNFT protocol | The influence of age, gender, ethnicity, and facial dimensions on fitting were assessed. | 17% of the subjects failed all attempts. 60% passed one attempt, and <15% passed between the 2 and 5 attempts. The males had higher passing rates than the females (OR: 1.51: 95%CI 1.27-1.81). The HCWs with a low BMI <18.5 kg/m^2^ had significantly lower odds of fit testing passing compared with other groups (OR: 0.516, 95%CI: 0.362-0.735, p<0.0001). There was a little discrimination between fit testing results and facial measurements. |

| **Study** | **Respirator Features**  **(Brand, model, size, style)** | **Subject Characteristics** | **Country** | **Standard Fit Testing Procedure** | **Study Procedure** | **Findings** |
| --- | --- | --- | --- | --- | --- | --- |
| De‐Yñigo‐Mojado et al., 2021 [71] | FFP3 and surgical  masks | 74 nurses  (37 males, 37 females) | Spain | ANSI/AIHA Z88.10 & OSHA 29CFR ‎‎1910.134  QNFT protocol, TSI PortaCount Pro+ model 8038 | The comparison of FFs for the studied respirators and masks was made between males and females. | There were no significant differences among males (2.86±2.73) and females (3.55±6.34) by mean FFs for the surgical masks (p= 0.18). There were significant differences among males (30.82±28.42) and females (49.65±43.04) by mean FFs for the FFP3 (p= 0.037). According to the OSHA criteria, only 2.70% and 13.51% of male and female nurses passed (p=0.199). Whereas, 21.62% and 48.64% of male and female nurses passed using the FFP3 respirator, according to the AIHA criteria (p= 0.027). |
| De-Yñigo-Mojado et al., 2020 [72] | FFP3 respirators, surgical masks, and other types of masks | 78 physicians  (37 males, 41 females) | Spain | ANSI/AIHA Z88.10-2010, OSHA ‎‎29CFR ‎‎1910.134 QNFT protocol, TSI PortaCount Pro+ model 8038 | The studied masks and respirators were assessed using the QNFT procedure. | The FFs for the FFP3 respirator were higher than for the surgical mask and other types of masks (40.7±37.8, 95% CI (32.3-49.1) vs. 3.2±5.0, 95% CI (2.1-4.3), p<0.001). |

| **Study** | **Respirator Features**  **(Brand, model, size, style)** | **Subject Characteristics** | **Country** | **Standard Fit Testing Procedure** | **Study Procedure** | **Findings** |
| --- | --- | --- | --- | --- | --- | --- |
| Green et al., 2021 [73] | 86 FFP3 respirator types (3M, RFP3FV, Easimask FSM, and Alpha Solway) | 17920 males, 4863 females) per seven hospitals  (21.90% males, 76.49% females) | UK | OSHA ‎‎29CFR ‎‎1910.134  QNFT  protocol | QNFT procedures were assessed by 41/58 (70.7%), QLFT procedures were used by 55/58 (94.8%), and 37/58 (63.8%) of the hospitals were employed both QLFT and QNFT procedures. | Approximately 20% of the HCWs failed the fit test during the COVID-19 pandemic. The mean passing rate was 80.74%. The males had higher failure rates for all respirators than the females (20.1% vs. 19.9%)  Failure rates of the HCWs from BAME backgrounds were high (25.69%). Across all seven hospitals, 18.98% of males tested failed the fit-test for all masks tested; 19.89% of females tested failed the fit-test for all masks used (X^2^ = 0.079, p= 0.398). |
| Sun et al., 2020 [74] | Two brands of FFRs, including FFP3 brand A and FFP1 brand B | Eight test subjects | Germany | OSHA 29 CFR 1910.134  QNFT protocol, TSI PortaCount model 8020 | The WPF obtained from PortaCount (CPC) with and without the N95-Companion was compared to the Moores flame photometer model 1100. The SWPF measured with the CPC was compared to the TSI SMPS. | A linear relationship was determined between the PortaCount (without N95-Companion) and flame photometer under all conditions (R^2^ = 0.9704). The distribution of particle size was similar in almost all cases. The SWPFs from CPC were correlated with SMPS (R^2^ = 0.70). |
| Vanhooydonck et al., 2021 [75] | Novel FFP3 FFR | ND | Belgium | OSHA ‎‎29CFR ‎‎1910.134  QNFT protocol, TSI PortaCount  Pro+ model 8038 | The fitting of various materials was evaluated in order to develop the novel FFR. | The fit testing of VMX Silicon 10A (1.5 mm) and Rolyan Polycushion (3.2 mm) in three replications showed that both obtained FF ranges of 210-550 and 320-420, respectively. |

| **Study** | **Respirator Features**  **(Brand, model, size, style)** | **Subject Characteristics** | **Country** | **Standard Fit Testing Procedure** | **Study Procedure** | **Findings** |
| --- | --- | --- | --- | --- | --- | --- |
| Winski et al., 2019 [76] | 3M 8835 + FFP3 respirator | 262 employees (237 males, 25 females) | UK | HSE OC282/28  QNFT protocol, TSI PortaCount Pro+ model 8038 | The effects of facial dimension on respirator fitting were investigated. | Fourteen (5.3%) subjects had FF < 100. The median FF was 416 (IQR: 294-604). No correlation was found between FF and face length (r= −0.08; p=0.214) and a negative correlation was observed between FF and face width (r= −0.17; p=0.006) and jaw width (r= −0.28; p<0.001). No differences were determined between the NIOSH panel face sizes (including small: 17, medium: 145, and large: 97) and FF (p= 0.194). All small-face subjects passed the fit test. |
| Chapman et al., 2022 [77] | A locally manufactured N95 respirator | 33 HCWs | Australia | OSHA ‎‎29CFR ‎‎1910.134 QNFT protocol, TSI PortaCount Pro+ | The QNFT procedure was performed and repeated five times to achieve an FF≥100. | The fit test passing rate was 63.6% (21 out of 33). Also, 19 participants passed the regular size and two passed the small size. No participants passed the X-large size. 84.8% of the participants failed at least one of the fit tests before passing. The fit coaching for the failure groups was provided by the manufacturer’s instructions to assure the users’ well-fitting respirators.  The mean FF was 162.4 ± 31.8 for the pass groups and 65.4 ± 60.8 for the failure groups. |

| **Study** | **Respirator Features**  **(Brand, model, size, style)** | **Subject Characteristics** | **Country** | **Standard Fit Testing Procedure** | **Study Procedure** | **Findings** |
| --- | --- | --- | --- | --- | --- | --- |
| Chen et al., 2022 [78] | 3M N95 respirator model 8210 | 21 healthy participants (Seven males, 14 females) | USA | Modified OSHA ‎‎29CFR ‎‎1910.134 QNFT protocol, CPC TSI model 3775 | The FFEs of the N95 respirator were compared among four scenarios, including uninstructed (reference), written/pictorial manufacturer's instructions, step-by-step video demonstration, and staff instruction (visual inspection of respirator fit and verbal suggestions to adjust). | The progressive trend in the FFEs from reference (86.1%, FF: 7.19) to manufacturer paper (93.3%, FF: 14.92), video (97.5%, FF: 40), and post-staff intervention (98.3%, FF: 58.82) was observed. The video instruction (p<0.037) and staff intervention (p<0.033) sessions significantly improved the FFEs for the baseline. |
| Clark et al., 2021 [79] | 3M 8210 N95 & 3M 1860S N95 surgical masks | Sixty-five dental and dental hygiene students  (45 males, 20 females) | USA | OSHA ‎‎29CFR ‎‎1910.134 QNFT protocol, Accufit 9000 fit tester | The fit testing procedure was performed on the participants. Next, the “safety perceptions” questionnaire was completed pre- and post-fit testing. | All participants knew how to wear the N95 respirator. 41 (63%) participants noted that their safety perceptions altered after fit testing. |

| **Study** | **Respirator Features**  **(Brand, model, size, style)** | **Subject Characteristics** | **Country** | **Standard Fit Testing Procedure** | **Study Procedure** | **Findings** |
| --- | --- | --- | --- | --- | --- | --- |
| Inolopú et al., 2023 [80] | 12 models FFRs, including 3M N95 1860, Xiantao Zhong N95 Yi, ZYB-11, Makrite N95 9500, 3M N95 9010, 3M PFF 9920H, Grande FFP2CDN3S-P2, KN95, PGT Care FFP2 PGT-0095, GIKO N95, 1200H, Benehal N95 MS6115L, Y&Z N95 Safety, Work F720, and Lucca Light KN95 / FFP2 Lucca Care | 263 HCWs | Perú | OSHA ‎‎29CFR ‎‎1910.134 QNFT protocol, TSI PortaCount Pro+ model 8048 | The FF of all studied respirators was assessed, and the effect of training on FF was determined for 184 HCWs. | Among all, 87 (33.1%) HCWs had FF>100, 27 (10.3%) ranged 50-99, and 149 (56.7%) had FF<50. Of 87 HCWs with optimal fitting, 83.9% were 3M 1860 FFR (mean FF: 126.0, 95% CI (109.4-146.6)). Of 27 HCWs with 50<FF<99, 25.9% were Makrite 9500. Of the 149 HCWs with FF<50, 38.9% and 31.5% were Xiantao Zhong Yi ZYB-11 and Makrite 9500, respectively. Also, the 3M respirator models increased the FF after post-instructional FF (p≤0.01). However, the Xiantao Zhong Yi ZYB-11 and Makrite 9500, KN95, Y&Z, Lucca Light, and Giko FFRs could not improve the post-instructional FF. |
| Low et al., 2021 [81] | BSN Medical ProShieldVR N95 respirator model TN01 (TN01-11 medium or TN01-12 small) | 65 participants including the Anaesthetists, anaesthesia registrars,  and nurses (33 males, 32 females) | Australia | OSHA ‎‎29CFR ‎‎1910.134 QNFT protocol, TSI PortaCount pro model 8048 | The quantitative respirator fit testing was assessed among the participants. | The fit test passing rate was lower than the USCs passing rate (22 (34%) vs. 65 (100%), p<0.0001). The overall passing rate following the education of the remaining 16 participants was 38 (58%). |

| **Study** | **Respirator Features**  **(Brand, model, size, style)** | **Subject Characteristics** | **Country** | **Standard Fit Testing Procedure** | **Study Procedure** | **Findings** |
| --- | --- | --- | --- | --- | --- | --- |
| Ngobeni et al., 2020 [82] | HALYARD Health N95-FFRs (46827, small size and 46727, regular size) | 37 HCWs (Two males, 35 females) | South Africa | OSHA ‎‎29CFR ‎‎1910.134 QNFT protocol, TSI PortaCount  Pro+ model 8038 | The fit test results obtained from the QNFT and QLFT and effects of models on respirator fit were evaluated. | About 37 out of 99 (37.4%) HCWs underwent QNFT and QLFT. Overall, 17 (45.9%) passed the QNFT procedures (S_e_= 0.45, Sp= 0.50). About eight out of 37 (34.8%) passed the N95-FFR model 46727 and three (60%) passed the N95-FFR model 46827. 46% of the HCWs (11/24) who had worn a respirator before and 47% of the HCWs (9/19) who had received prior training passed the fit test. |
| Robertsen et al., 2020 [83] | ND | 240 participants (146  males, 18 females, others not determined) | USA | HSE QNFT protocol,  TSI PortaCount Plus model 8038 | The respondents were classified into intervention group 1 (fit testing) and group 2 (fit testing with a lecture on exposure. The control group received no training. The respondents completed subjective knowledge, attitudes and behavior questionnaire on the RPE use. | An improvement in knowledge of Group 1 (5.0 vs. 6.0) and Group 2 (5.50 vs. 6.25), attitudes (4.29 vs. 4.43), and organizational support of Group 1 (5.50 vs. 5.67) occurred, while an improvement in subjective norms related to RPE use occurred in intervention Group 2 (3.5 vs. 4.33). No significant difference was observed in intention to use or rate of respirator use. Participation in both groups could improve the intention to use respirators. |
| Seo et al., 2021 [84] | Two types of domestic N95 masks (folder and cup styles) | 59 HCWs (16 males, 43 females) | Korea | OSHA ‎‎29CFR ‎‎1910.134  QNFT protocol,  TSI PortaCount Plus model 8038 | The FFs of folder types were compared to cup-types before and after training. | The GM±GSD FF value for the cup-style was significantly higher than the folder type (62.18±3.22 vs. 22.65±4.18, p=0.001). There was a significant difference between the FFs before and after training, p= 0.0015). |
| **Study** | **Respirator Features**  **(Brand, model, size, style)** | **Subject Characteristics** | **Country** | **Standard Fit Testing Procedure** | **Study Procedure** | **Findings** |
| Williams et al., 2021 [85] | Four N95 respirators, including Semi-rigid cup 3M-1860 or 1860S, Flat-fold BYD Care, Duckbill BSN medical  ProShield, and Halyard Fluidshield | 125 HCWs  (29 males, 94 females, 2 other) | Australia | OSHA ‎‎29CFR ‎‎1910.134  QNFT protocol,  TSI PortaCount Plus model 8038 | Subjects underwent online training, and the QNFT procedure. After that, subjects’ skills in respirator donning and doffing were checked. The knowledge, attitudes, and skills toward RPE use were assessed. | The knowledge, donning and doffing skills, and USCs were significantly improved (p< 0.01). |
| Yeon et al., 2020 [86] | TB N95 mask | 56 HCWs, including, nurses (one male, 55 females) | Korea | OSHA ‎‎29CFR ‎‎1910.134  QNFT protocol, SIBATA MT-03 | The subjects were assigned into experimental (n=28) and control groups (n=28). The knowledge and attitude toward PPE use were evaluated.  Then, the fit test was conducted between the experimental and control groups. | There were no significant differences between knowledge and attitude toward PPE use. 19 (68%) of the experimental group and 14 (50%) of the control group passed the fit test (p= 0.354). |
| Xiao et al., 2023 [87] | N95 mask | 442 Hospital staff, Property logistics staff (272 males, 170 females) | China | OSHA ‎‎29CFR ‎‎1910.134 QNFT protocol, TSI PortaCount Pro+ model 8048 | The fitting of the N95 mask was assessed after various training sessions. | Significant differences were found between various training programs and the passing fit test rate (p<0.05). Passing rates increased after three tests, as follows: 239 (54.07%), 355 (80.32%) and 405 (91.63%), respectively. |

**Note:**

FFR: filtering facepiece respirator

QNFT: Quantitative Fit Test

QLFT: Qualitative Fit Test

CNC: Condensation Nuclei Counter

CPC: Condensation Particle Counter

FF: Fit Factor

TILPF: Total Inward Leakage Protection Performance

aFF: adopted fit factor

HCWs: Healthcare workers

Se: Sensitivity

Sp: Specificity

PPV: Positive predictive value

NPV: Negative predictive value

FFE: fitted filtration efficiency

GRPF: General respirator protection factor

PPG: partially passed group

APG: group that passed all exercises

ET: Endotracheal tube intubation

VL: video laryngoscopy

DL: direct laryngoscopy

OR: odd ratio

AUC: area under the curve

ROC: receiver operating characteristic curve

QNFF values: quantitative fit factor

TP: true positive

TN: true negative

FN: false negative

FE: filtration efficiency

SWPF: simulated workplace protection factor

IL: inward leakage

CI: Confidence Interval

BAME: Black, Asian, and Minority Ethnic

NIOSH: The National Institute for Occupational Safety and Health

NIOH: National Institute for Occupational Health

EHRs: Elastomeric half-facepiece respirators /reusable facepiece respirators

PF: protection factor

SSM: silicone-molded face mask

GM: geometric mean

GSD: geometric standard deviation

RFC: respirator fit capability

PPR: panel passing rate

PAPR: powered air purifying respirator

TIL: Total Inward Leakage

MAVerIC: Modified Airway from VEntilatoR Circuit

LPFs: laboratory protection factors

OV cartridges: organic vapor cartridges

AFM: Anaesthesia Face Mask

MSM: Modified Snorkeling Mask

IQR: Interquartile Range

APR: air-purifying respirator

SCBA: self-contained breathing apparatus

ND: Not determined

**Table 2.** **Quantitative fit testing of reusable respirators and affective factors**

| **Study** | **Respirator Features**  **(Brand, model, size, style)** | **Subject Characteristics** | **Country** | **Standard Fit Testing Procedure** | **Study Procedure** | **Findings** |
| --- | --- | --- | --- | --- | --- | --- |
| Anwari et al., 2021 [88] | A novel reusable half-face  respirator | Eight different volunteers, including members of the design and testing team (six males, two females) | Canada | CSA Z94.4–18 QNFT protocol | The fitting of the developed reusable half-face respirator was assessed over nine prototype iterations. | Seven out of eight (87.5%) tests passed. Although the Manitoba SSR mask with Intersurgical Hydro-Mini filter obtained the FF of the 108, failed the fit test exercises, including turning side-to-side; 93, talking; 83, and bending; 92 <100. |
| Chichester et al., 2020 [89] | Additively manufactured respirators | ND | USA | OSHA ‎‎29CFR ‎‎1910.134‎  QNFT protocol, ‎TSI PortaCount  ‎Pro+ model 8038 | Several AMR were modified and equipped with filter media, and their fitting was compared to the N95 mask. | Nine separate fit test evaluations were conducted. The AMR equipped with large foam and N95 and P100 filter could provide satisfactory protection (FF≥200) compared to the N95 mask (FF: 189). |
| Fadairo et al., 2020 [90] | Eight brands of half-facepiece and full-facepiece respirators (3M, MSA, North and Moldex) equipped with 3M, North, MSA, Moldex P-100 filters | Mannequin and eight subjects (Six African American males, one African American female, and one Asian male) | USA | OSHA 29 CFR 1910.134 & ANSI Z88.10-2001, QNFT protocols, CNC TSI PortaCount Pro^+^ model 8038 & CNP QHD fit testers | The fitting of CNC and CPC protocols was assessed using both humans fit factor (HFF) and mannequin fit factor (SMFF). | There was a significant difference in the results of CNC using the mannequin under ambient and controlled environmental conditions (26319.1 vs. 18382.6, p=0.0005) in contrast to the CNP results (1679.50 vs. 1879.75, p= 0.7247). While no significant difference was observed in the CNP or CNC for the subjects (p> 0.05). Also, significant differences were observed in ambient and environmental conditions using the mannequin and subjects. |
| **Study** | **Respirator Features**  **(Brand, model, size, style)** | **Subject Characteristics** | **Country** | **Standard Fit Testing Procedure** | **Study Procedure** | **Findings** |
| Hondjeu et al., 2021 [91] | Duo silicone respirator  and 3M N95 respirators (1870+, 1860, 1860S  8210, 14.6%, and 9105S) | 41 HCWs | Canada | CSA Z94.4-18  QNFT protocol, Levitt-Safety AccuFIT 9000™ fit tester | The fit testing of 3M N95 respirators was compared to the Duo respirator. | The passing rates for the 3M N95 disposable and Duo reusable respirators were 58.5% and 100%, respectively. The 3M 1870+ and 8210 respirators had the highest pass rates (78% and 83%, respectively). The harmonic means of the FFs for the Duo respirator was higher than for the N95 respirators (2959 vs. 77.4, p<0.0001). The N95 respirators had a lower passing rate during dynamic maneuvers than stationary maneuvers (61% vs. 73%, p< 0.0001). Also, seven subjects (17.1%) were outside of the NIOSH panel. |
| Ballard et al., 2021 [92] | 3D-printed prototypes from the rigid (n=5 designs) and flexible polymers (n=5 designs**),** and disposable N95 respirator | Four HCWs | USA | OSHA 29CFR 1910.134  QNFT protocol, TSI PortaCount Pro model 8048 | The 3D-printed prototypes underwent QNFT procedures on subjects who previously passed the fit testing of the commercial N95 respirators. | The 3D-printed prototypes with rigid materials did not pass the QNFT procedure. Also, three out of the five prototypes with flexible materials failed the fit test.  Only two final 3D-printed prototypes with flexible materials had an overall mean FF of 138 (108-168) compared to the control N95 respirator (FF> 200, p<0.001). |
| **Study** | **Respirator Features**  **(Brand, model, size, style)** | **Subject Characteristics** | **Country** | **Standard Fit Testing Procedure** | **Study Procedure** | **Findings** |
| Ballard et al., 2021 [93] | N95 respirators (a cloth-based respirator (Sewn Sterilization Wrap), three 3D-printed respirators (P100 Adaptor, Self-Moldable 3D Printed and Multi-Part 3D Printed) and one repurposed from medical supplies (Elastomeric), and 3M 1860 N95 FFR | Seven adult volunteers, including, intended users (HCWs) | USA | OSHA ‎‎29CFR ‎‎1910.134 QNFT protocol, TSI PortaCount Pro+ model 8048 | All six designs were assessed during the QNFT. | Only the EHR equipped with a HEPA filter passed the fit test on both small and large face- standardized users (FF: 110 and 108, respectively). |
| Duda et al., 2020 [94] | Six 3D-printed  face mask designs | Four participants | Germany | Modified OSHA ‎‎29CFR ‎‎1910.134‎ EN149 standard  QNFT ‎ protocol, TSI PortaCount^R^ model 8020M ‎Pro | The PF and TIL values of six different face mask designs, including HSU*/*BwKWST FM V3, HSU*/*BwKWST FM V4, Montana mask, Maker mask respirator, PLA COVID-19 mask, TPU COVID-19 mask, and fabric mask, were assessed using the PortaCount. | The PF and TIL values were measured: HSU FM V3: 2.19, 45.69%; HSU FM V4: 2.43, 41.24; Montana mask: 1.72, 58.25%; Maker mask: 1.88, 53.35%; PLA COVID-19 mask: 2.81, 35.71%; TPU COVID-19 mask: 2.33, 43.01%; and Fabric mask: 2.23, 44.78%. |
| **Study** | **Respirator Features**  **(Brand, model, size, style)** | **Subject Characteristics** | **Country** | **Standard Fit Testing Procedure** | **Study Procedure** | **Findings** |
| Imbrie-Moore et al., 2020 [95] | 3D-printed mask adaptor | Six subjects | USA | Modified  OSHA ‎‎29CFR ‎‎1910.134‎  QNFT protocol, ‎TSI PortaCount  ‎Pro^+^ | The 3D-printed mask adaptor with the 3M 1860 N95 respirator was developed. The quantitative fit test was conducted using the PortaCount respirator fit tester. | All subjects passed the fit testing of the proposed mask. The overall FF was 148>100. |
| Levine et al., 2022 [96] | 3D-Printed Masks (Covid Mask Respirator, Low Poly, and Covid-19 Respirator), N95 and a KN95 respirators | Five volunteers (Three males, two females) | USA | Modified OSHA ‎‎29CFR ‎‎1910.134 QNFT protocol, TSI PortaCount  Pro+ | The FFs of a novel mask were compared to the N95 and KN95 respirators. | The Mask 1, Mask 3, and KN95 respirators had an FF of 52.2, 1.8, and 5.4, respectively. The Mask 2 (Low Poly Low Poly Covid-19 Face Mask Respirator) had a higher FF≥100. All subjects passed the quantitative fit testing of Mask 2 and the N95 respirator.  There was no significant difference between the mean FFs for the Mask 2 and the N95 respirators (141.25 vs. 175.60, p< 0.226). |
| Liu et al., 2020 [97] | 3M^TM^ re-usable elastomeric respirators equipped with  a 3D-printed adaptor | Eight volunteers  (Five males, three females) | China | OSHA 29CFR 1910.134  QNFT protocol, TSI PortaCount Pro model 8038 | A 3D-printed adaptor was designed for the 3M 7501 (small) and 3M 6200 (medium) elastomeric respirators. All volunteers underwent USCs (NPCs and PPCs) and quantitative fit testing procedures using the modified respirator. | All volunteers passed the USCs. Four out of eight volunteers passed the 3M 7501 (small) respirator. Also, all females were fitted with the 3M 7501 (small) respirator. |
| **Study** | **Respirator Features**  **(Brand, model, size, style)** | **Subject Characteristics** | **Country** | **Standard Fit Testing Procedure** | **Study Procedure** | **Findings** |
| Manomaipiboon et al., 2020 [98] | Silicone VJR-NMU N99 half-piece respirator | Forty-one  HCWs (21 males, 20 females) | Thailand | OSHA 29 CFR 1910.134, QNFT protocol,  Sibata MT-05U | The fitting of a novel respirator was assessed. | 32 (78%) subjects passed the first fit test. After tightening the O-ring trap, seven subjects passed the fit test (77.8%). Five subjects passed the third fit test (80%). The overall fit test passing rate was 40/41 (97.6%). One subject failed, even after adjusting the strap for the third time. |
| Martelly et al., 2021 [99] | A Reusable, Hot Water Moldable, Additively Manufactured Mask | 13 subjects  (Six males, seven females) | USA | OSHA ‎‎29CFR ‎‎1910.134 QNFT protocol, TSI PortaCount | A 3D-printed moldable mask with multiple sizes, including small, medium, large, medium-narrow, medium-wide, and medium-flat, was developed as a substitution for the N95 respirator and was assessed during the QNFT procedures. | There was an improvement in fit between the unmolded and molded masks (7 ± 17 vs. 143 ± 62). The molded mask had a passing rate of 77% (10 out of 13). |
| Meadwell et al., 2019 [100] | Nine designs of elastomer | One human subject | UK | OSHA ‎‎29CFR ‎‎1910.134 QNFT protocol, TSI PortaCount | The pressure set up, including inhaling and exhaling against the elastomers containing short and long beard, were developed and compared to the formal fit testing. Any air leakage resulted in low pressure. | The pressure testing performed well; however, it could not be substituted by robust fit testing. The highest FF obtained by continuous ribs-soft elastomer (18.51) with regards to the long beard. |

| **Study** | **Respirator Features**  **(Brand, model, size, style)** | **Subject Characteristics** | **Country** | **Standard Fit Testing Procedure** | **Study Procedure** | **Findings** |
| --- | --- | --- | --- | --- | --- | --- |
| McLeod et al., 2021 [101] | 3M EHR model 6000 | Mannequin | Canada | OSHA ‎‎29CFR ‎‎1910.134 QNFT protocol, TSI PortaCount  Pro+8038 | The FFs for the EHRs equipped with the studied filters were compared to the controls (P100 and N95 masks). | The FFs were highest for the EHRs with two layers of 7093 3M NIOSH P100 Particulate Filter was 2281, and two layers of P100 3M 2097 NIOSH were 1678. The FF for the combinations of Super-calendered Final Product (1 ply) -Side overhang and P100 3M 2097 NIOSH was 341. The FF for the combinations of uncalendered Final Product (2 ply) -Side overhang and P100 3M 2097 NIOSH was 215. |
| Ng et al., 2020 [102] | The reusable silicone-molded face mask (SSM) and N95 respirator | 40 HCWs  (20 males, 20 females) | Canada | CSA Z94.4–18 &  OSHA ‎‎29CFR ‎‎1910.134‎ CNC QNFT  ‎ protocol,  AccuFIT 9000™ fit tester | The subjects were quantitatively fitted using the SSM and also, the N95 respirator, which previously passed the qualitative fit testing procedure. | The mean harmonic FFs for the N95 respirator and SSM were 137.9 and 6316.7, respectively. The overall passing rates for the mentioned masks were 65% and 100%, respectively. |

| **Study** | **Respirator Features**  **(Brand, model, size, style)** | **Subject Characteristics** | **Country** | **Standard Fit Testing Procedure** | **Study Procedure** | **Findings** |
| --- | --- | --- | --- | --- | --- | --- |
| Roche et al., 2022 [103] | Personalized 3D-printed respirator | 50 HCWs (21 males, 29 females) | UK | OSHA ‎‎29CFR ‎‎1910.134 QNFT protocol, TSI PortaCount Pro+ model 8038 | The fitting of the 3D-printer respirator was compared to the control Alpha Solway 3030V FFP3 respirator. | In the control group, 38 subjects passed and 12 failed the FFP3. In the test group, 44 passed and six failed the 3D-printed respirator. 11 subjects who failed the FFP3 passed the 3D-printed respirator. Conversely, five who passed the FFP3 failed the 3D-printed respirator. No significant difference was found in the fitting rate of both respirators (170 vs. 180, p= 0.21). |
| Chughtai et al., 2020 [104] | CleanSpace™ lightweight tight-fitting half-facepiece PAPR | 20 HCWs including nursing and medical staff (13 males, seven females) | Australia | OSHA ‎‎29CFR ‎‎1910.134 QNFT protocol, TSI PortaCount  Pro+8038 | The quantitative fitting of novel respirator was examined. | All participants passed the fit test with a GM FF (GSD) of 6768 (3755). |
| Germonpre et al., 2020 [105] | Snorkel Masks | Staff of Belgium  Hospitals (HCWs) | Belgium | Modified OSHA ‎‎29CFR ‎‎1910.134‎  QNFT protocol, TSI PortaCount ‎  Pro+ 8038 | The fit testing procedure was conducted by the TSI PortaCount Pro+ 8038 on the thirteen modified snorkel masks. The FFs of the studied masks were compared to the 3M Aura 9322+ FFP2 NR). | The modified snorkel mask had high FFs. Subea A: 58, Subea B, C: 200+, Subea D: 200++, Subea E: 52, Seac: 200+, Aqualung: 117, Cressi:157, Ocean Reef A:57, Ocean Reef B, C, D, and E:200+, and 3M Aura 9322+ FFP2: 62. |

| **Study** | **Respirator Features**  **(Brand, model, size, style)** | **Subject Characteristics** | **Country** | **Standard Fit Testing Procedure** | **Study Procedure** | **Findings** |
| --- | --- | --- | --- | --- | --- | --- |
| Greig et al., 2020 [106] | Modified full-face snorkel mask | One male user | UK | OSHA ‎‎29CFR ‎‎1910.134‎  QNFT ‎ protocol, TSI PortaCount ‎Pro model 8038 | A developed mask made from Omew full-face snorkel mask, 3D printed adaptor, and Intersurgical Cleartherm-3 HME filter. The fitting of novel snorkel mask was evaluated. | The novel mask failed the fit test despite passing the USCs. Then, it was considered that the QNFT procedure was required for the full-face mask. |
| Greig et al., 2022 [107] | Full-face snorkel mask | 16 clinical staff (Seven males, nine females) | UK | BS ISO 16975-3:2017, QNFT ‎ protocol, TSI PortaCount ‎Pro model 8030 | A novel full-face snorkel mask was made from an  anaesthetic breathing circuit heat and moisture exchange filter and/or a CE-marked P3- grade filter. The P3 filters were mounted in two styles of both epoxy-coated and uncoated adaptors. Then, the developed mask was fit tested. | One fit test considered a pass when a P3 was mounted with an uncoated adaptor to a snorkel mask (FF: 564). No subjects passed using the coated adaptor. All subjects who used the HME filter failed the fit test (median (IQR) FF: 8 (3–23)). The coated P3 adaptors had a higher median (IQR) FF than the uncoated P3 ones (899 (350–1396) vs. 349 (169–462)). |
| Grinshpun et al., 2020 [108] | Three makes and models of respirators, N95  FFR, P100 FFR, and half-mask elastomeric facepiece (11 respirators) | 25 adult subjects (9 males, 16 females) | USA | ANSI standard (Z88.10-2010)  QNFT protocol, TSI PortaCount model 8038 | The performance of the novel AccuFIT 9000 apparatus was extensively compared against the PortaCount^R^ (reference instrument) during the QNFT procedure. | The AccuFIT 9000 could identify poor-fitting respirators with a sensitivity of 0.95, a specificity of 0.97, and a Kappa of 0.92. |

| **Study** | **Respirator Features**  **(Brand, model, size, style)** | **Subject Characteristics** | **Country** | **Standard Fit Testing Procedure** | **Study Procedure** | **Findings** |
| --- | --- | --- | --- | --- | --- | --- |
| Harmata et al., 2022 [109] | Three Full-face piece gas respirators, including MP-5, MP-6, and Promask | Ten participants | Poland | OSHA 29 CFR 1910.134  QNFT protocol, TSI PortaCount  Pro+ | The FFs of respirators were evaluated after seven days. | The FFs for the MP-6 mask, 1460, for the MP-5 mask, 950, and for the Promask mask, 850, were obtained. The FFs for the MP-6 masks after three days, MP-5 mask, and Promask after two days reached <10000. |
| Kechli et al., 2020 [110] | Full-face snorkel mask | - | USA | OSHA ‎‎29CFR ‎‎1910.134‎  QNFT protocol, TSI PortaCount ‎  Pro+ model 8020 | The QNFT procedure was done on the full-face snorkel mask using the PortaCount Plus Model 8020. | The modified full-face snorkel mask had an overall FF of 142. The only talking exercise had an FF of 94< 100. |
| Kroo et al., 2021 [111] | Modified Full-Face Snorkel Masks (Pneumask) | Three volunteers | USA | OSHA ‎‎29CFR ‎‎1910.134‎  QNFT ‎ protocol, TSI PortaCount ‎Pro ‎ | We created a 3D printed adapter and a full-face snorkel mask modified with HEPA anesthesia circuit filter and a HME anesthesia circuit filter on a Dolfino Frontier mask. The QLFT and QNFT procedures were carried out. | All three subjects passed the QNFT procedure. |

| **Study** | **Respirator Features**  **(Brand, model, size, style)** | **Subject Characteristics** | **Country** | **Standard Fit Testing Procedure** | **Study Procedure** | **Findings** |
| --- | --- | --- | --- | --- | --- | --- |
| Nicholson et al., 2021 [112] | Ocean Reef Aria full face snorkel masks (medium/large, small/medium, large/extra large), and S/M full-faced snorkel masks | One user | USA | OSHA 29 CFR 1910.134  QNFT protocol, TSI PortaCount  model 8030 | The fitting characteristics of the snorkel masks were examined during the fit test. | The FFs of the 3M 6800 full face respirator, Snorkel mask with a duct tape, Snorkel mask with no modifications, and snorkel mask with a mouth cover remove (333867, 32281, 15448, and 1105) were obtained. |
| Persing et al., 2021 [113] | 3M EHR with P100 organic vapor (OV) cartridges model 65021HA1 | A single member of the  research team | USA | Modified OSHA 29 CFR 1910.134  QNFT protocol, TSI PortaCount Pro^+^  model 8038 | The laboratory protection factors (LPFs) of a dual channel optical particle counter (DC OPC) and a dual-channel condensation particle counter (DC CPC) were compared to the TSI PortaCount Pro^+^. | The LPFs for the DC CPC and PortaCount were similar, while the DC OPC was different from PortaCount. The LPF of the PortaCount was 89, DC CPC was 77, and DC OPC was 156, per the target LPF of 100 against the Sodium chloride aerosol, and 370, 330, and 961, respectively, per the target LPF of 300 against the Sodium chloride aerosol. |
| Pettinger et al., 2021 [114] | Three respirators, including  FFP2 respirator, Anaesthesia Face Mask (AFM), and full-face Modified Snorkeling Mask (MSM) | Ten HCWs, including anaesthesiology  residents (Five males, five females) | Belgium | OSHA 29 CFR 1910.134  QNFT protocol, TSI PortaCount  model 8038 | The three different respirators were evaluated by the QNFT procedure. | The seal check failure rates were 37 (41%) for the FFP2 (control), 10 (11%) for the AFM, and 6 (7%) for the MSM. There was no significant difference among the FFs of the studied respirators. The fit test passed rates for the FFP2 (control) were 5 (50%), 8 (80%) for the AFM, and 7 (70%) for the MSM, p= 0.69. |
| **Study** | **Respirator Features**  **(Brand, model, size, style)** | **Subject Characteristics** | **Country** | **Standard Fit Testing Procedure** | **Study Procedure** | **Findings** |
| Bergman et al., 2019 [115] | Six respirators, including three families of full-facepiece respirators, including a one-size-only family, a  two-size family, and a three-size family equipped with P-100 filters | 25 subjects | USA | OSHA ‎‎29CFR ‎‎1910.134  QNFT protocol, TSI PortaCount model 8038 | The feasibility assessment of half-facepiece respirator fit capability (RFC) criteria according to ASTM F23.65 was conducted for full-facepiece respirators. Subjects performed two donning. The panel passing rate (PPR) determines the number or percentage of subjects in the panel achieving acceptable fit on at least one of two donning. | The PPR was more than 75%. One of two donnings achieved the FF of 500. The PPRs for the three-size, two-size, and one-size families were 100%, 79%, and 88%, respectively. The PPR decreased with increasing FFs of 500, 1000, and 2000. |
| Chehade et al., 2021 [116] | Two masks, including assembled mask Hans Rudolf full-face mask & Respironics Performax full-face mask | Twenty volunteers from Oklahoma City Veteran Affairs Health Care System  (Ten males, ten females) | USA | OSHA ‎‎29CFR ‎‎1910.134 QNFT protocol, AccuFit 9000 fit testing system | The novel mask underwent quantitative fit testing. | All participants passed the test with a GM±GSD of 2317±3.8. |

| **Study** | **Respirator Features**  **(Brand, model, size, style)** | **Subject Characteristics** | **Country** | **Standard Fit Testing Procedure** | **Study Procedure** | **Findings** |
| --- | --- | --- | --- | --- | --- | --- |
| Han et al., 2022 [117] | Three types of respirators, including N95, half mask, and full facepiece | 50 volunteer college students (25 males, 25 females) | Korea | OSHA ‎‎29CFR ‎‎1910.134 QNFT protocol, TSI PortaCount  Pro model 8038 | The SIBATA MT 05U fit tester was compared to the PortaCount Pro+ 8038 (as a reference) for three respirators. | There was a high correlation between two fit testers (p< 0.00001). The FF of 100 per N95 respirator determined by PortaCount equalized to the FF of 75 by SIBATA MT. There was very high consistency between two devices for half- and full-facepiece respirators, which both satisfied the values specified by the ANSI standard. But the N95 respirator did not meet the ANSI requirement. |

| **Study** | **Respirator Features**  **(Brand, model, size, style)** | **Subject Characteristics** | **Country** | **Standard Fit Testing Procedure** | **Study Procedure** | **Findings** |
| --- | --- | --- | --- | --- | --- | --- |
| Rengasamy et al., 2021 [118] | NIOSH-approved elastomeric  half-facepiece, full-facepiece, and PAPRs with respirators tight-  fitting and loose-fitting facepiece | 16 subjects | USA | OSHA 29CFR 1910.134  QNFT protocol, TSI PortaCount Pro+ model 8038 | The FFs and TILs for the four respirators were assessed using the NaCl and corn oil aerosol. | The FFs were obtained for the MSA EHR: 1507, North EHR: 1667, MSA Full-facepiece: 4670, North Full-facepiece: 7753, PAPR-tight fitting; MSA: 7731, Bullard: 3799. Also, the TILs for the MSA EHR for corn oil aerosol were significantly larger than for NaCl aerosol (0.197 vs. 0.056) and for the North EHR (0.086 vs. 0.038). However, the TILs for the NaCl aerosol were significantly larger than for corn oil aerosol per the PAPRs but not per the full-facepiece respirators, including the MSA PAPR-tight fitting (0.010 vs. 0.003), Bullard PAPR-tight fitting (0.011 vs. 0.002), 3M PAPR-loose-fitting (0.013 vs. 0.003), Bullard PAPR-fitting (0.015 vs. 0.002), MSA Full-facepiece (0.046 vs. 0.049), and 3M Full-facepiece (0.015 vs. 0.016). |

| **Study** | **Respirator Features**  **(Brand, model, size, style)** | **Subject Characteristics** | **Country** | **Standard Fit Testing Procedure** | **Study Procedure** | **Findings** |
| --- | --- | --- | --- | --- | --- | --- |
| Sietsema et al., 2022 [119] | NIOSH-approved Envo quarter-facepiece  elastomeric respirator | 25 HCWs of Rush University hospital (14 males, 11 females) | USA | OSHA 29CFR 1910.134  QNFT protocol, TSI PortaCount Pro model 8038 | The simulated workplace protection factor of the studied respirator was assessed while subjects doing the simulated healthcare activities for 30 min. | The median (5th and 95th percentile) FF was 188 (48, 201), SWPF-truncated SWPF was 181 (94, 199), and non-truncated SWPF was 570 (153, 1508). |
| Weng et al., 2022 [120] | Novel full-face mask | 18 participants, (Eight males, 10 females) | Taiwan | OSHA ‎‎29CFR ‎‎1910.134 QNFT protocol, PortaCount® fit tester model 8038 | The fitting of a novel full-face nebulizer mask equipped with a HEPA filter was assessed. | The mask could provide acceptable protection. |

| **Study** | **Respirator Features**  **(Brand, model, size, style)** | **Subject Characteristics** | **Country** | **Standard Fit Testing Procedure** | **Study Procedure** | **Findings** |
| --- | --- | --- | --- | --- | --- | --- |
| Clinkard et al., 2021 [121] | N95, snorkel masks with high-efficiency filters and snorkel masks with powered-air purifying  respirators | 51 HCWs, including physicians and nurses (24 males, 27 females) | Canada | CAN/CSA-Z94.4-18  CNC QNFT protocol,  AccuFIT 9000 respirator fit tester | Three studied respirators were tested using the aerosol condensation nuclei counter quantitative fit testing method, AccuFIT 9000.  A high-efficiency filter and PAPR were attached to the snorkel mask. Then, 51 HCWs underwent the QNFT procedure. | 59% and 20% of participants failed at one or more fit test exercises using the N95s and snorkel masks with high-efficiency filters, respectively. 24% and 12% of the subjects failed the overall FFs of N95 and snorkel masks with high-efficiency filters. The mean FF for snorkel masks with a PAPR (12177) and snorkel masks with a high-efficiency filter (2939) was significantly higher than that of the N95 mask (144), p<0.05. The passing proportions of the N95 respirator (65%) and snorkel mask with a high-efficiency filter (92%) were lower than those of the snorkel mask with PAPR (100%, p<0.01). |
| Convissar et al., 2020 [122] | Modified Airway from VEntilatoR Circuit (MAVerIC) | One anesthesia provider | USA | ND | A makeshift filter mask called “MAVerIC” which made from the standard adult anesthesia breathing circuit with HEPA filters, a facemask, and an operating room head strap. | The cost-benefit quantitative fit testing procedure consisted of Bag valve mask (an Ambu ‎bag) with a pressure manometer was carried out using the MAVerIC. |

| **Study** | **Respirator Features**  **(Brand, model, size, style)** | **Subject Characteristics** | **Country** | **Standard Fit Testing Procedure** | **Study Procedure** | **Findings** |
| --- | --- | --- | --- | --- | --- | --- |
| Toigo et al., 2021 [123] | Aria Ocean Reef® full-face snorkeling mask | 71 HCWs,  including nurses, respiratory therapists, physicians, residents, patient attendants, technicians, and care advisors | Canada | OSHA ‎‎29CFR ‎‎1910.134 QNFT protocol, TSI PortaCount Pro^+^ model 8038 | The QNFT procedure using the novel mask were assessed. | Four out of 71 subjects underwent the QNFT, and all of them passed.  55 out of 67 conducted fit tests and passed the QLFT. 83.1% of the subjects who could not pass the fit testing of medical respirators passed the fit testing of the snorkel mask. |
| Cass et al., 2022 [124] | Two N95 respirator brands and CleanSpace  HALO® powered air-purifying respirator | 189 ICU staff members, including doctors, nurses, allied health professionals, and support staff member  (61 males, 128 females) | Australia | OSHA ‎‎29CFR ‎‎1910.134 QNFT protocol, TSI PortaCount 8040 fit tester | Passing rates of the studied masks were compared between the provision of assisted and unassisted fitting. | Fit testing failure rates were 18/60 (30.0%) for the 3M and 33/107 (30.8%) for the Halyard. The passing fit test rate increased from 88/189 (46.6%, 95% CI, 39.3-53.9%) on unassisted fitting to 105/189 (55.6%, 95% CI 48.2-62.8%) after the provision of assistance on the first respirator type worn and 131/189 (69.3%, 95% CI ¼ 62.2e75.8%) per the second respirator type. Fifty-eight of 189 (30.7%, 95% CI, 24.2-37.8%) failed on both N95 respirator types, and 47 (100%) subjects proceeded to and passed the fit testing on CleanSpace HALO^®^ PAPR. |

| **Study** | **Respirator Features**  **(Brand, model, size, style)** | **Subject Characteristics** | **Country** | **Standard Fit Testing Procedure** | **Study Procedure** | **Findings** |
| --- | --- | --- | --- | --- | --- | --- |
| Baba et al., 2022 [125] | Replaceable particulate  respirators (RPRs) Chiyoda model 1180–05 and PAPR Chiyoda model BL–321S. | Ten participants from University of Occupational and Environmental Health (Eight males, two females) | Japan | Japan Industrial Standard (JIS) T8150 fit test  MT–03 device Sibata fit tester | The FFs for the studied respirators were assessed during the exercising tasks and resting state. | The passing rate and mean FFs of both RPR (i.e., RPR-H: at resting state 3 and at exercise state: 2 out of 10 subjects, 68.2 vs. 118.7) and PAPR (i.e., PAPR-R: at resting state 10 and at exercise state: 9, 786.5 vs. 444.5) obtained from the exercising tasks were higher than the resting state (p<0.001). But the PAPR provided satisfactory protection (FF> 100). |
| Grinshpun et al., 2020 [126] | 3M Versaflow, TR-300+ PAPR | Ten human subjects and one manikin | USA | OSHA 29 CFR 1910.134  CPC/CNC QNFT protocol, TSI PortaCount | The efficacy of real-time performance monitor (RePM) for PAPR was assessed compared to the CPC method by mPF (manikin) and SWPF (human subject). | The MPF was measured ranged from 5000-10000. The SWPF ranged from 3000-10000. A near-perfect correlation was observed between the two methods (0.997). There was a high correlation between RePM and CPC in measuring different particle size ranges. High sensitivity (96.3%) and specificity (100%) achieved on human subjects at a response time of 60 sec. |
| Kessel et al., 2022 [127] | PAPR | One HCW  (A rural healthcare provider) | USA | OSHA ‎‎29CFR ‎‎1910.134 QNFT protocol, TSI PortaCount  model 8030 | The Stryker Flyte surgical helmets were converted into emergency PAPRs and their FFs were assessed. | The helmet equipped with two layers of H600 filter media had the highest FF of 2229 against NaCl and 28942 against SiO2. |
| **Study** | **Respirator Features**  **(Brand, model, size, style)** | **Subject Characteristics** | **Country** | **Standard Fit Testing Procedure** | **Study Procedure** | **Findings** |
| McGrath et al., 2022 [128] | Bubble-PAPR | 15 clinical and non-clinical staff, including  nurses, doctors, physiotherapists, advanced practitioners, speech and language therapists, representing emergency medicine, critical care, orthopaedics and obstetric specialties  (Five males, 10 females) | UK | HSE282  QNFT protocol, TSI PortaCount Pro^+^ model 8040 | Ten subjects underwent fit testing of a novel PAPR. | Ten subjects passed the fit test. The mean FF was 16931> 500. |
| Nagel et al., 2021 [129] | 3D-printable  PAPR | Two subjects | USA | OSHA 29 CFR 1910.134  QNFT protocol, TSI PortaCount Pro^+^ model 8038 | The modified PAPR was assessed using the QNFT procedure. | The novel PAPR obtained the FF of 1362≥500 using the PortaCount. |

| **Study** | **Respirator Features**  **(Brand, model, size, style)** | **Subject Characteristics** | **Country** | **Standard Fit Testing Procedure** | **Study Procedure** | **Findings** |
| --- | --- | --- | --- | --- | --- | --- |
| Goto et al., 2021 [130] | Tight-fitting PAPR (BL-321H half-mask respirator  and a BLA-62; KOKEN LTD filter) | Fifty-four HCWs, including doctor, nurse, and other HCWs (33 males, 21 females) | Japan | OSHA ‎‎29CFR ‎‎1910.134 QNFT protocol, TSI PortaCount | The SWPF of the studied PAPRs was assessed. | 42 (78%) of the subjects failed at least one of the three sessions of chest compression (SWPF <500). 39 (72%), 30 (56%), and 25 (46%) failed in the first, second, and third sessions, respectively. The median (IQR) for overall SWPF was 4304 (685–16191). Therefore, tight-fitting PAPR could not provide adequate protection. |
| Ng et al., 2023 [131] | HALO PAPR | Eight HCWs (Four males, four females) | Australia | OSHA ‎‎29CFR ‎‎1910.134 QNFT protocol, TSI PortaCount model 8048 | The SPF was assessed at rest, during, and immediately after subjects performed the chest compression. | The mean FF was higher than 1000. There were no significant differences before, during, or after the chest compression. The FFs were when power off: 1869 (617–4333), 1748 (378–6881), and 1243 (669–3881), respectively, and when power on: 3576 (2128–6,109), 4290 (2048–4931), and 4135 (2913–6890), respectively. |
| Rees et al., 2021 [132] | PAPR | Five subjects (Three males, two females) | UK | OSHA ‎‎29CFR ‎‎1910.134,  QNFT protocol, PortaCount 8040 fit tester | A novel ESAB PAPR was adapted with a custom  cowl placed over the air intake fans to prevent from occlusion. Also, two different single-use hoods were made from TyvekVR. Then, subjects underwent fit testing while using the PAPR. | The mean FF for the PAPR was 1851 (277). The FF was not reduced during the speech, and there were exaggerated maneuvers. It is required that PAPR be equipped with a powered pack to ensure protection for the users. |
| **Study** | **Respirator Features**  **(Brand, model, size, style)** | **Subject Characteristics** | **Country** | **Standard Fit Testing Procedure** | **Study Procedure** | **Findings** |
| Sekoguchi et al., 2020 [133] | BL-321S Koken Ltd. PAPR with tight-fitting and half-facepiece respirator | Ten subjects of University of Occupational and Environmental Health (eight males, two females) | Japan | JIS T8150, QNFT protocol,  Sibata MT-03 fit tester | The leakage rate of the PAPR and RPR was assessed based on the recommended method and the non-recommended method (knit cover and towel placed over between the facepiece and face, or headband on a helmet). | The leakage rate for the RPR was 1.82-10.92% (FF: 9.16-54.94) and 0.18-0.42% (FF: 238.10-555.55) for the PAPR. The performance of the RPR decreased, while the performance of the PAPR was not significantly different. |
| Sekoguchi et al., 2022 [134] | Two respirators, including SHIGEMATSU WORKS DR77SR2  And SHIGEMATSU WORKS Sy11G2 PAPR | Eight men workplaces participants | Japan | JIS T8150 QNFT protocol, MT-05U fit tester | The WPFs of C-RPR (correct donning), U-RPR (incorrect donning), and PAPR were assessed. | The GM±SD WFPs for the C-RPR, U-RPR, and PAPR were 17.7±2.59, 27.0±3.86, and 117.3±5.25. |
| Temmesfeld et al., 2022 [135] | Novel PAPR | Six subjects (One male, five females) and one mannequin | Norway | OSHA ‎‎29CFR ‎‎1910.134‎  QNFT ‎ protocol, TSI PortaCount ‎Pro model 8038 | A filter adaptor was developed to convert the surgical helmet into a PAPR, and the TIL of the developed device was investigated. | The TIL for the surgical helmet with a PAPR filter adaptor using a mannequin did not exceed 0.07% (FF: 1428.57) for any particle size at any time of the 23-minute-lasting loading cycle. Also, the mean and maximum TIL obtained from testing on subjects were 0.00465% (FF: 21505.38) and 0.00759% (FF: 13175.23), respectively. |
| **Study** | **Respirator Features**  **(Brand, model, size, style)** | **Subject Characteristics** | **Country** | **Standard Fit Testing Procedure** | **Study Procedure** | **Findings** |
| Xu et al., 2023 [136] | Four MSA Safety respirators, including two half masks (410 and 420 APRs) and two full masks (3S APR and Ultra Elite SCBA) | 225 chemical plant operators and maintenance and laboratory personnel | China | OSHA ‎‎29CFR ‎‎1910.134 QNFT protocol, CNC TSI PortaCount model 8038 Pro+ & CNP OHD Quantifit fit testers | The studied subjects underwent CNC and CNP fit testing protocols, and then influencing factors, including gender, age, educational background, and training were examined. | The passing rates were 88.1% for males and 75.6% for females. Most females donned small-size respirators due to their thin faces and sharp chins. Gender had a significant effect on fitting (X^2^= 5.186, p=0.023). Other factors had no significant influence on respirator fitting. The half-masks had a lower passing rate than the full-masks (84.7% vs. 91.6%, p<0.05). The 410 and 420 models of APRs (81.6% vs. 86.5%, respectively). The passing rate for 3S APR was 90.0% and for Ultra Elite SCBA was 95.2%. Also, about 35.5% of the fit test exercises’ failures contributed to the talking. There were high correlations between CNC and CNP data with regards to facing forward, bending over, shaking the head, wearing the mask again, and moving the head up and down. |
| Rowlett et al., 2021 [137] | Elastomeric half-mask respirators | 327 ASSPs | USA | OSHA ‎‎29CFR ‎‎1910.134 QNFT protocol | The perceptions of the participants regarding the QNFT protocol were examined. | 90% of the participants were familiar with the QNFT procedures. Only a significant difference was found in the perceived accuracy of the QNFT by level of experience (p= 0.006). |

**Note:**

FFR: filtering facepiece respirator

QNFT: Quantitative Fit Test

CNC: Condensation Nuclei Counter

CPC: Condensation Particle Counter

FF: Fit Factor

TILPF: Total Inward Leakage Protection Performance

aFF: adopted fit factor

HCWs: Healthcare workers

Se: Sensitivity

Sp: Specificity

PPV: Positive predictive value

NPV: Negative predictive value

FFE: fitted filtration efficiency

GRPF: General respirator protection factor

PPG: partially passed group

APG: group that passed all exercises

ET: Endotracheal tube intubation

VL: video laryngoscopy

DL: direct laryngoscopy

OR: odd ratio

AUC: area under the curve

ROC: receiver operating characteristic curve

QNFF values: quantitative fit factor

TP: true positive

TN: true negative

FN: false negative

FE: filtration efficiency

SWPF: simulated workplace protection factor

IL: inward leakage

CI: Confidence Interval

BAME: Black, Asian, and Minority Ethnic

NIOSH: The National Institute for Occupational Safety and Health

NIOH: National Institute for Occupational Health

EHRs: Elastomeric half-facepiece respirator / reusable facepiece respirators

PF: protection factor

SSM: silicone-molded face mask

GM, GSD: geometric mean, geometric standard deviation

RFC: respirator fit capability

PPR: panel passing rate

PAPR: powered air purifying respirator

TIL: Total Inward Leakage

MAVerIC: Modified Airway from VEntilatoR Circuit

LPFs: laboratory protection factors

OV cartridges: organic vapor cartridges

S/M: small/medium

AFM: Anaesthesia Face Mask

MSM: Modified Snorkeling Mask

IQR: Interquartile Range

APR: air-purifying respirator

SCBA: self-contained breathing apparatus

ND: Not determined

**References**

1. Brandel A, Tanner J, Gao J, Kelly N, Snyder A. Testing and Developing DIY Masks. University of Michigan. 2020. Available from: <https://deepblue.lib.umich.edu/handle/2027.42/164436?show=full>.

2. Buckley J, Gladle M, Murray K, Sample W. SUBJECT: Quantitative Respirator Fit Testing of HensMask. University of Delaware. 2020. Available from: <https://me.udel.edu/wp-content/uploads/2020/04/HensNest-FitTest.pdf>.

3. Coyle JP, Derk RC, Lindsley WG, Boots T, Blachere FM, Reynolds JS, et al. Reduction of exposure to simulated respiratory aerosols using ventilation, physical distancing, and universal masking. Indoor Air. 2022;32(2):e12987. doi: 10.1111/ina.12987. PMID: 35225389

4. Dang AJ, Kumfer BM, Bertroche JT, Glidden JO, Oxford CR, Jammalamadaka U, et al. Challenges in predicting the filtration performance of a novel sewn mask: Scale-up from filter holder to mannequin measurements. Aerosol Air Qual Res. 2021;21(6). doi: 10.4209/aaqr.200629.

5. Drouillard KG, Tomkins A, Lackie S, Laengert S, Baker A, Clase CM, et al. Fitted filtration efficiency and breathability of 2-ply cotton masks: Identification of cotton consumer categories acceptable for home-made cloth mask construction. PLoS One. 2022;17(3):e0264090. doi: 10.1371/journal.pone.0264090. PMID: 35316263

6. Duncan S, Bodurtha P, Naqvi S. The protective performance of reusable cloth face masks, disposable procedure masks, KN95 masks and N95 respirators: Filtration and total inward leakage. PLoS One. 2021;16(10):e0258191. doi: 10.1371/journal.pone.0258191. PMID: 34614026

7. Mueller AV, Eden MJ, Oakes JM, Bellini C, Fernandez LA. Quantitative Method for Comparative Assessment of Particle Removal Efficiency of Fabric Masks as Alternatives to Standard Surgical Masks for PPE. Matter. 2020;3(3):950-62. doi: 10.1016/j.matt.2020.07.006. PMID: 32838296

8. Reutman SR, Reponen T, Yermakov M, A. Grinshpun S. Homemade facemasks: particle filtration, breathability, fit, and other performance characteristics. J Occup Environ Hyg. 2021;18(7):334-44. doi: 10.1080/15459624.2021.1925124. PMID: 34080950

9. Teesing GR, van Straten B, de Man P, Horeman-Franse T. Is there an adequate alternative to commercially manufactured face masks? A comparison of various materials and forms. J Hosp Infect. 2020;106(2):246-53. doi: 10.1016/j.jhin.2020.07.024. PMID: 32763333

10. Wentworth F. Advanced Manufacturing Center_Comparative Protective Mask Material Testing. The University of Maine. 2020. Available from: <https://digitalcommons.library.umaine.edu/c19_amc/19/>.

11. Lindsley WG, Blachere FM, Law BF, Beezhold DH, Noti JD. Efficacy of face masks, neck gaiters and face shields for reducing the expulsion of simulated cough-generated aerosols. Aerosol Sci Technol. 2021;55(4):449-57. doi: 10.1080/02786826.2020.1862409. PMID: 35924077

12. Sato J, Yamawaki Y, Ito M, Endo M, Tanaka R, Shino M. Measurement of the leak rate of masks used for anticancer drug handling using a mask fitting tester. J Oncol Pharm Pract. 2020;26(6):1318-23. doi: 10.1177/1078155219891210. PMID: 31822202

13. Ardon-Dryer K, Warzywoda J, Tekin R, Biros J, Almodovar S, Weeks BL, et al. Mask Material Filtration Efficiency and Mask Fitting at the Crossroads: Implications during Pandemic Times. Aerosol Air Qual Res. 2021;21(7). doi: 10.4209/aaqr.200571.

14. Bodas CR, Ng I, Kave B, Begg F, Williams DL. A randomised crossover trial of two flat-fold cup respirators: BYD DE2322 N95 versus Care Essentials MSK-002 P2. Infect Dis Health. 2022. doi: 10.1016/j.idh.2022.08.002. PMID: 36207250

15. Cameron S, Cheung W, Cronin N, Griffiths K, Hunt R, Innes L, et al. Quantitative fit testing with limited supplies of respirator masks in hospital personnel during the COVID-19 pandemic. Aust Health Rev. 2020;44(4):542-3. doi: 10.1071/ah20154. PMID: 32718421

16. Chan JK, Yep KH, Magarey S, Keon-Cohen Z, Acheson M. Fit Testing Disposable P2/N95 Respirators during COVID-19 in Victoria, Australia: Fit Check Evaluation, Failure Rates, and a Survey of Healthcare Workers. COVID. 2021;1(1):83-96. doi: 10.3390/covid1010007.

17. Christopher L, Rohr-Kirchgraber T, Mark S. The PPE Pandemic: Sex-Related Discrepancies of N95 Mask Fit. EMJ Microbiol Infect Dis.2(1):57-63. doi: 10.33590/emjmicrobiolinfectdis/20-00215.

18. Cloet A, Yu M, Arnold J, Griffin L. Activity and Usability Evaluation of Alternative Protective Face Mask Designs. Proc Hum Factors Ergon Soc Annu Meet. 2022;66(1):1682-6. doi: 10.1177/1071181322661162.

19. Cloet A, Griffin L, Yu M, Durfee W. Design considerations for protective mask development: A remote mask usability evaluation. Appl Ergon. 2022;102:103751. doi: 10.1016/j.apergo.2022.103751. PMID: 35339761

20. Griffin L, Yu MJ, Cloet A, Arnold S, Carlson N, Hillmyer M, et al. Protective Masks Utilizing Nonendangered Components. J Med Device. 2022;16(1). doi: 10.1115/1.4053720. PMID: 35280214

21. Duncan S, Bodurtha P, Bourgeois C, Dickson E, Jensen C, Naqvi S. The impact of extreme reuse and extended wear conditions on protection provided by a surgical-style N95 filtering facepiece respirator. J Occup Environ Hyg. 2020;17(11-12):546-59. doi: 10.1080/15459624.2020.1829633. PMID: 33166226

22. Fabre V, Cosgrove SE, Hsu YJ, Jones GF, Helsel T, Bukowski J, et al. N95 filtering face piece respirators remain effective after extensive reuse during the coronavirus disease 2019 (COVID-19) pandemic. Infect Control Hosp Epidemiol. 2021;42(7):896-9. doi: 10.1017/ice.2021.76. PMID: 33602376

23. Nakamoto K, SARAYA Sr T, Kurai D, Fukukawa N, Taneoka T, Shimasaki T, et al. Reusing N95 Respirators at Weekly Intervals During the COVID-19 Pandemic. Cureus. 2021;13(2).

24. Greenawald LA, Moore SM, Yorio PL. PPE CASE: Evaluation of Fit and Strap Extension Performance of Stockpiled Filtering Facepiece Respirators from One U.S. Facility. By Greenawald LA, Moore SM, and Yorio PL. Pittsburgh, PA: U.S. Department of Health and Human Services, Centers for Disease Control and Prevention, National Institute for Occupational Safety and Health, NPPTL Report Number P2021-0102. 2021.

25. Hai CH, Hua KK, Fu GQ, Singh K, Wah YC. Comparing Quality of Fitting of Different Filtering Face Pieces in a Healthcare Worker: A preliminary observational study. J Posit School Psychol. 2022;6(3):4012–8.

26. Han S-H, Cha K-S, Yoo S-Y, Han JO. Comparison of N95 Respiratory Mask Fit Testing. Korean J Healthc Assoc Infect Control 2021;26(2):108-14. doi: 10.14192/kjicp.2021.26.2.108.

27. Hwang SY, Yoon H, Yoon A, Kim T, Lee G, Jung KY, et al. N95 filtering facepiece respirators do not reliably afford respiratory protection during chest compression: A simulation study. Am J Emerg Med. 2020;38(1):12-7. doi: 10.1016/j.ajem.2019.03.041. PMID: 30955924

28. Fakherpour A, Jahangiri M, Seif M, Charkhand H, Abbaspour S, Floyd EL. Quantitative fit testing of filtering face-piece respirators during the COVID-19 pandemic reveals anthropometric deficits in most respirators available in Iran. J Environ Health Sci Eng. 2021;19(1):805-17. doi: 10.1007/s40201-021-00648-3. PMID: 33875931

29. Jankusol K, Chaiear N, Mitsungnern T. Different fit factors for the N95 respirator during endotracheal intubation: Comparing video laryngoscopy and direct laryngoscopy. Asia Pac J Sci Technol. 2023;28(2):APST-28-02-12. doi: 10.14456/apst.2023.28.

30. Joshi M, Khan A, Sapra BK. Quick laboratory methodology for determining the particle filtration efficiency of face masks/respirators in the wake of COVID-19 pandemic. J Ind Text.51(5S

):7622S-40S. doi: 10.1177/1528083720975084.

31. Jean-Romain D, David V, Guillaume S, de Damien C, Walter Z, Vincent P, et al. Distribution of low quality filtering facepiece respirators during the COVID-19 pandemic: An independent analysis of the situation in Switzerland. Swiss Med Wkly. 2021;151(3). doi: 10.4414/smw.2021.20459. PMID: 33516162

32. Jung J, Kim J, Yang H, Lim Y-J, Kwak S-H, Hong MJ, et al. Fit-failure rate associated with simulated reuse and extended use of N95 respirators assessed by a quantitative fit test. Infect Control Hosp Epidemiol. 2021;42(11):1313-7. doi: 10.1017/ice.2021.5. PMID: 33487185

33. Kamal M, Bhatti M, Stewart WC, Johns M, Collins D, Shehabi Y, et al. Safety Goggles with Elastic Headband to Improve N95 Fit Following Failed Quantitative Fit Test. Indian J Crit Care Med

2023;27(6):386. doi: 10.5005/jpjournals-10071-24473. PMID: 37378367

34. Kyaw S, Johns M, Lim R, Stewart WC, Rojas N, Thambiraj SR, et al. Prediction of N95 Respirator Fit from Fogging of Eyeglasses: A Pilot Study. Indian J Crit Care Med. 2021;25(9):976-80. doi: 10.5005/jp-journals-10071-23947. PMID: 34963713

35. Landry SA, Subedi D, Barr JJ, MacDonald MI, Dix S, Kutey DM, et al. Fit-tested N95 masks combined with portable HEPA filtration can protect against high aerosolized viral loads over prolonged periods at close range. J Infect Dis. 2022; 226(2):199-207. doi: 10.1093/infdis/jiac195. PMID: 35535021

36. Lindsley WG, Blachere FM, Beezhold DH, Law BF, Derk RC, Hettick JM, et al. A comparison of performance metrics for cloth masks as source control devices for simulated cough and exhalation aerosols. Aerosol Sci Technol. 2021;55(10):1125-42. doi: 10.1080/02786826.2021.1933377. PMID: 35923216

37. Long KD, Woodburn EV, Berg IC, Chen V, Scott WS. Measurement of filtration efficiencies of healthcare and consumer materials using modified respirator fit tester setup. PLoS One. 2020;15(10). doi: 10.1371/journal.pone.0240499. PMID: 33048980

38. Milosevic M, Biswas RK, Innes L, Ng M, Darendeliler AM, Wong A, et al. P2/N95 filtering facepiece respirators: Results of a large-scale quantitative mask fit testing program in Australian health care workers. Am J Infect Control. 2022;50(5). doi: 10.1016/j.ajic.2021.12.016. PMID: 34971710

39. Ng I, Kave B, Begg F, Bodas CR, Segal R, Williams D. N95 respirators: quantitative fit test pass rates and usability and comfort assessment by health care workers. Med J Aust. 2022;217(2):88-93. doi: 10.5694/mja2.51585. PMID: 35645035

40. O’Kelly E, Arora A, Ward J, Clarkson PJ. How well do face masks protect the wearer compared to public perceptions? medRxiv [Preprint] 2021 [Posted 2021 January 31]. [6 p.]. Available from: <https://www.medrxiv.org/content/10.1101/2021.01.27.21250645v1.full-text>. doi: 10.1101/2021.01.27.21250645.

41. O'Kelly E, Arora A, Pirog S, Ward J, Clarkson PJ. Comparing the fit of N95, KN95, surgical, and cloth face masks and assessing the accuracy of fit checking. PLoS One. 2021;16(1). doi: 10.1371/journal.pone.0245688. PMID: 33481870

42. O’Kelly E, Arora A, Pirog S, Ward J, Clarkson PJ. Experimental Measurement of the Size of Gaps Required to Compromise Fit of an N95 Respirator. Disaster Med Public Health Prep. 2022;17:1-13. doi: 10.1017/dmp.2022.23. PMID: 35057880

43. Park JJ, Seo YB, Lee J. Fit Test for N95 Filtering Facepiece Respirators and KF94 Masks for Healthcare Workers: a Prospective Single-center Simulation Study. J Korean Med Sci. 2021;36(21):e140. doi: 10.3346/jkms.2021.36.e140. PMID: 34060256

44. Popov T, Popov G, Basse A. Development and Application of a Modified Procedure for Quantitative Fit Testing of Disposable Masks and Respirators. J Occup Environ Hyg. 2022;19(5):266-70. doi: 10.1080/15459624.2022.2050741. PMID: 35259072

45. Regli A, Sommerfield A, Thalayasingam P, von Ungern-Sternberg BS. N95 Masks to Protect Health Care Workers: Is the New Fast Fit-Test Protocol Cutting Corners? Chest. 2022;161(6):1606-8. doi: 10.1016/j.chest.2022.01.048. PMID: 35131299

46. Regli A, Thalayasingam P, Bell E, Sommerfield A, von Ungern-Sternberg BS. More than half of front-line healthcare workers unknowingly used an N95/P2 mask without adequate airborne protection: An audit in a tertiary institution. Anaesth Intensive Care. 2021;49(5):404-11. doi: 10.1177/0310057X211007861. PMID: 34325537

47. Prince SE, Chen H, Tong H, Berntsen J, Masood S, Zeman KL, et al. Assessing the effect of beard hair lengths on face masks used as personal protective equipment during the COVID-19 pandemic. J Expo Sci Environ Epidemiol. 2021;31(6):953-60. doi: 10.1038/s41370-021-00337-1. PMID: 34006963

48. Sandaradura I, Goeman E, Pontivivo G, Fine E, Gray H, Kerr S, et al. A close shave? Performance of P2/N95 respirators in healthcare workers with facial hair: results of the BEARDS (BEnchmarking Adequate Respiratory DefenceS) study. J Hosp Infect. 2020;104(4):529-33. doi: 10.1016/j.jhin.2020.01.006. PMID: 31978416

49. De-Yñigo-Mojado B, Becerro-de-Bengoa-Vallejo R, Losa-Iglesias ME, Madera-García J, Rodríguez-Sanz D, Calvo-Lobo C, et al. Facial Hair Decreases Fit Factor of Masks and Respirators in Healthcare Providers. Biology (Basel). 2021;10(10). doi: 10.3390/biology10101031. PMID: 34681128

50. Sasko LM, Oliver B, Smith SM. Fit testing of masks worn by frontline healthcare workers. Infect Control Hosp Epidemiol. 2023:1-2. doi: 10.1017/ice.2022.268. PMID: 36594247

51. Seo H, Myong J-P, Kang B-k, Kwon Y-i. Necessity of the Fit Test Panel for Korean Respirator Users: Application to Korean Healthcare Workers. J Int Soc Respir Prot. 2021;38(2):1-11.

52. Seo H, Kang B-k, Kwon Y-i. Fit testing for domestic N95 medical masks. J Korean Soc Occup Environ Hyg. 2020;30(2):124-33. doi: 10.15269/JKSOEH.2020.30.2.124.

53. Sheikh F. N95 Respirators for a Diverse Population of Healthcare Workers: A Mixed-Methods, Pilot and Feasibility Study. M.Sc. Thesis, McMaster University. 2022. Available from: <https://macsphere.mcmaster.ca/handle/11375/28032>.

54. Sickbert-Bennett EE, Samet JM, Clapp PW, Chen H, Berntsen J, Zeman KL, et al. Filtration Efficiency of Hospital Face Mask Alternatives Available for Use During the COVID-19 Pandemic. JAMA Intern Med. 2020;180(12):1607-12. doi: 10.1001/jamainternmed.2020.4221. PMID: 32780113

55. Suen LKP, Guo YP, Ho SSK, Au-Yeung CH, Lam SC. Comparing mask fit and usability of traditional and nanofibre N95 filtering facepiece respirators before and after nursing procedures. J Hosp Infect. 2020;104(3):336-43. doi: 10.1016/j.jhin.2019.09.014. PMID: 31545991

56. Goh DYT, Mun MW, Lee WLJ, Teoh OH, Rajgor DD. A randomised clinical trial to evaluate the safety, fit, comfort of a novel N95 mask in children. Sci Rep. 2019;9(1):18952. doi: 10.1038/s41598-019-55451-w. PMID: 31831801

57. Salter SJRA. Reinventing cloth masks in the face of pandemics. 2021;41(5):731-44.

58. Vahabzadeh‐Hagh AM, Patel SH, Stramiello JA, Weissbrod PA. Patient‐worn endoscopy mask to protect against viral transmission. Laryngoscope Investig Otolaryngol. 2022;7(1):190-6. doi: 10.1002%2Flio2.708. PMID: 35155797

59. Vo E, Horvatin M, Bergman M, Wu B, Zhuang Z. A technique to measure respirator protection factors against aerosol particles in simulated workplace settings using portable instruments. J Occup Environ Hyg. 2020;17(5):231-42. doi: 10.1080/15459624.2020.1735640. PMID: 32243774

60. Vuma CD, Manganyi J, Wilson K, Rees D. The effect on fit of multiple consecutive donning and doffing of N95 filtering facepiece respirators. Ann Work Expo Health. 2019;63(8):930-6. doi: 10.1093/annweh/wxz060. PMID: 31504129

61. Williams DL, Kave B, Lee K, Segal R, Krieser RB, Mezzavia PM, et al. A randomised crossover study to compare the user seal check and quantitative fit test between two types of duckbill N95 particulate respirator masks: The Halyard Fluidshield (R) N95 and the BSN Medical ProShield (R) N-95 particulate respirator masks. Anaesth Intensive Care. 2021;49(2):112-8. doi: 10.1177/0310057X20974022. PMID: 33818131

62. Williams DL, Kave B, Begg F, Bodas C, Ng I. Randomized crossover study comparing quantitative fit tests between Trident (TM) and 3M (TM) Aura (TM) N95/P2 respirators. Infect Dis Health. 2022;27(2):61-5. doi: 10.1016/j.idh.2021.10.002. PMID: 34799300

63. Williams DL, Kave B, Begg F, Bodas C, Ng I. Quantitative fit-test concordance of a pair of similar-fit 3M Aura respirator models, 3M 9320A+ and 3M 1870+: A randomized crossover study. Infect Control Hosp Epidemiol. 2022;44(2):1-4. doi: 10.1017/ice.2022.67. PMID: 35387701

64. Williams DL, Kave B, Bodas C, Begg F, Roberts M, Ng I. Optimizing twin sampling tube stabilization improves quantitative fit test results for flat-fold duckbill filtering facepiece respirators. Am J Infect Control. 2022;51(6):694-8. doi: 10.1016/j.ajic.2022.09.026. PMID: 36216035

65. Lim Y-H, Kim W, Choi Y, Kim H-C, Na G, Kim H-R, et al. Effects of Particulate Respirator Use on Cardiopulmonary Function in Elderly Women: a Quasi-Experimental Study. J Korean Med Sci. 2020;35(10). doi: 10.3346/jkms.2020.35.e64. PMID: 32174063

66. Mottay L, Le Roux J, Perumal R, Esmail A, Timm L, Sivarasu S, et al. KN95 filtering facepiece respirators distributed in South Africa fail safety testing protocols. S Afr Med J. 2020;111(3):13162. doi: 10.7196/samj.2021.v111i3.15381. PMID: 33334390

67. Zhang X, Jia N, Wang Z. The relationship between the filtering facepiece respirator fit and the facial anthropometric dimensions among Chinese people. Ind Health. 2020;58(4):318-24. doi: 10.2486/indhealth.2019-0158. PMID: 31787708

68. Boogaard B, Tas A, Nijssen J, Broeren F, van den Dobbelsteen J, Verhoeven V, et al. Efficacy Assessment of Newly-designed Filtering Facemasks during the SARS-CoV-2 Pandemic. Aerosol Air Qual Res. 2021;21(3):200424. doi: 10.4209/aaqr.2020.07.0424.

69. Carvalho CYM, Schumacher J, Greig PR, Wong DJN, El-Boghdadly K. Prospective observational study of gender and ethnicity biases in respiratory protective equipment for healthcare workers in the COVID-19 pandemic. BMJ Open. 2021;11(5):e047716. doi: 10.1136/bmjopen-2020-047716. PMID: 34016664

70. Caggiari S, Bader D, Packman Z, Robinson J, Tranka S, Böhning D, et al. Retrospective evaluation of factors affecting successful fit testing of respiratory protective equipment during the early phase of COVID-19. BMJ Open. 2023;13(5):e065068. doi: 10.1136/bmjopen-2022-065068. PMID: 37230519

71. De‐Yñigo‐Mojado B, Madera‐García J, Becerro‐De‐Bengoa‐Vallejo R, Losa‐Iglesias ME, Rodríguez‐Sanz D, Calvo‐Lobo C, et al. Fit factor compliance of masks and FFP3 respirators in nurses: A case–control gender study. J Adv Nurs. 2021. doi: 10.1111/jan.14823. PMID: 33733471

72. De-Yñigo-Mojado B, Madera-García J, Becerro-de-Bengoa-Vallejo R, Losa-Iglesias ME, Rodríguez-Sanz D, San-Antolín M, et al. Fit factor of masks used by Physicians in Clinical Settings. Int J Med Sci. 2020;17(17):2696-702. doi: 10.7150/ijms.50657. PMID: 33162797

73. Green S, Gani A, Bailey M, Brown O, Hing CB. Fit-testing of respiratory protective equipment in the UK during the initial response to the COVID-19 pandemic. J Hosp Infect. 2021;113:180-6. doi: 10.1016/j.jhin.2021.04.024. PMID: 33940089

74. Sun C, Thelen C, Sanz IS, Wittmann A. Evaluation of a new workplace protection factor–measuring method for filtering facepiece respirator. Saf Health Work. 2020;11(1):61-70. doi: 10.1016/j.shaw.2019.11.001. PMID: 32206375

75. Vanhooydonck A, Van Goethem S, Van Loon J, Vandormael R, Vleugels J, Peeters T, et al. Case study into the successful emergency production and certification of a filtering facepiece respirator for Belgian hospitals during the COVID-19 pandemic. J Manuf Syst. 2021;60:876-92. doi: 10.1016/j.jmsy.2021.03.016. PMID: 33814674

76. Winski TA, Mueller WA, Graveling RA. If the mask fits: facial dimensions and mask performance. Int J Ind Ergon. 2019;72:308-10. doi: 10.1016/j.ergon.2019.05.011.

77. Chapman D, Chapman L, Ganesan A. Quantitative respirator fit tests for P2/N95 in Australian general practice. Aust J Gen Pract. 2022;51. doi: 10.31128/ajgp-covid-51-1. PMID: 35172324

78. Chen H, Pennington ER, Case MW, Tong H, Rappold AG, Samet JM, et al. Improvement in Fitted Filtration Efficiency of N95 Respirators With Escalating Instruction of the Wearer. AJPM Focus. 2022;1(1):100014. doi: 10.1016/j.focus.2022.100014. PMID: 36338466

79. Clark TH. Alteration of Perceptions of Safety Before and After Fit Testing among College of Dentistry Students. MSc. degree, University of Nebraska Medical Center. 2021. Available from: <https://digitalcommons.unmc.edu/cgi/viewcontent.cgi?article=1136&context=coph_slce>.

80. Inolopú J, Mayma K, Curisinche-Rojas M, Aylas R, Flores JA, Rosales J. Quantitative Fit Testing on Filtering Facepiece Respirators in Use by Peruvian Healthcare Workers Caring for Tuberculosis Patients During the COVID-19 Pandemic: PROFIT Study 2020. 2023. doi: 10.3390/ijerph20166618. PMID: 37623201

81. Low CS, Weinberg L, Ellard LM, Hacking DF, Banyasz D. Pass rate of the BSN Medical ProShield® N95 filtering facepiece using quantitative fit testing in frontline anaesthetists and anaesthesia nurses working on a COVID-19 airway team. Anaesth Intensive Care. 2021;49(4):322-3. doi: 10.1177/0310057x21997150. PMID: 34039048

82. Ngobeni K. Qualitative Versus Quantitative Fit-Testing of Two Commonly Used Respirators in Resource-Limited Healthcare Facilities: University of Johannesburg (South Africa); 2020.

83. Robertsen Ø, Hegseth MN, Føreland S, Siebler F, Eisemann M, Vangberg HCB. The Effect of a Knowledge-Based Intervention on the Use of Respirators in the Norwegian Smelter Industry. Front Psychol. 2020;11:270. doi: 10.3389%2Ffpsyg.2020.00270. PMID: 32153476

84. Seo H, Kwon Y-i, Myong J-P, Kang B-k. Fit comparison of Domestic N95 Medical Masks in a Fit test. J Korean Soc Occup Environ Hyg. 2021;31(1):94-104. doi: 10.15269/JKSOEH.2021.31.1.94.

85. Williams D, Kave B, Begg F, Marshall C, Segal R, Ng I. Impacts on staff after implementation of a respiratory protection program in a Victorian public hospital. Infect Dis Health. 2021;26(4):265-72. doi: 10.1016/j.idh.2021.06.001. PMID: 34176771

86. Yeon JH, Shin YS. Effects of education on the use of personal protective equipment for reduction of contamination: a randomized trial. SAGE Open Nurs. 2020;6:2377960820940621. doi: 10.1177/2377960820940621. PMID: 33415295

87. Xiao B, Sun L-L, Yuan J, Xiao W-L, Liu Y, Cai M-Y, et al. Investigation of a Mask Fitness Test Based on Self-Efficacy and Diversified Training in the Assessment System for Nosocomial Infection Training. Infect Drug Resist. 2023;16:313-22. doi: 10.2147/idr.s388784. PMID: 36691491

88. Anwari V, Ng WCK, Hondjeu ARM, Xiao ZX, Afenu E, Trac J, et al. Development, manufacturing, and preliminary validation of a reusable half-face respirator during the COVID-19 pandemic. PLoS One. 2021;16(3). doi: 10.1371/journal.pone.0247575. PMID: 33730106

89. Chichester DL, Hix JD, Johnson JT, Ocampo Giraldo LA, Watson SM, Mortensen BT, et al. Evaluation of an Additively Manufactured Respirator for Personnel Protection from Particulates. Idaho National Lab.(INL), Idaho Falls, ID (United States); 2020.

90. Fadairo OJ. Comparison of Condensation Nuclei Counter and Controlled Negative Pressure Methods under Different Environmental Conditions Tested with a Mannequin and Human Subjects. Doctoral dissertation, West Virginia University. 2020. Available from: <https://researchrepository.wvu.edu/etd/7986/>. doi: 10.33915/etd.7986.

91. Hondjeu ARM, Ng WC, Anwari V, Xiao MZ, Rozenberg D, Kazlovich K, et al. A reusable, locally manufactured, half-face respirator provides better protection than fitted disposable N95 masks: development and quantitative fit-testing comparison. Research Square [Preprint] 2021 [cited 2023 July 9] Available from: <https://wwwresearchgatenet/publication/351605094_A_reusable_locally_manufactured_half-_face_respirator_provides_better_protection_than_fitted_disposable_N95_masks_development_and_quantitative_fit-testing_comparison>. doi: 10.21203/rs.3.rs-456096/v1.

92. Ballard DH, Jammalamadaka U, Meacham KW, Hoegger MJ, Burke BA, Morris JA, et al. Quantitative Fit Tested N95 Respirator-Alternatives Generated With CT Imaging and 3D Printing: A Response to Potential Shortages During the COVID-19 Pandemic. Acad Radiol. 2021;28(2):158-65. doi: 10.1016%2Fj.acra.2020.11.005. PMID: 33257256

93. Ballard DH, Dang AJ, Kumfer BM, Weisensee PB, Meacham JM, Scott AR, et al. Protection levels of N95-level respirator substitutes proposed during the COVID-19 pandemic: safety concerns and quantitative evaluation procedures. BMJ Open. 2021;11(9):e045557. doi: 10.1136/bmjopen-2020-045557. PMID: 34475144

94. Duda S, Hartig S, Hagner K, Meyer L, Intriago PW, Meyer T, et al. Potential risks of a widespread use of 3D printing for the manufacturing of face masks during the severe acute respiratory syndrome coronavirus 2 pandemic. J 3D Print Med. 2020;4(3):135-47. doi: 10.2217%2F3dp-2020-0014.

95. Imbrie-Moore AM, Park MH, Zhu Y, Paulsen MJ, Wang H, Woo YJ. Quadrupling the N95 Supply during the COVID-19 Crisis with an Innovative 3D-Printed Mask Adaptor. Healthcare (Basel). 2020;8(3). doi: 10.3390/healthcare8030225. PMID: 32717841

96. Levine M, Levine L, Xun H, Mathew PJ, Singh D, Gerber A, et al. Face Off: 3D Printed Masks as a Cost-Effective and Reusable Alternative to N95 Respirators: A Feasibility Study. Am J Med. 2022;135(9):1109-15. doi: 10.1016/j.amjmed.2022.04.026. PMID: 35580720

97. Liu DCY, Koo TH, Wong JKK, Wong YH, Fung KSC, Chan Y, et al. Adapting re-usable elastomeric respirators to utilise anaesthesia circuit filters using a 3D-printed adaptor - a potential alternative to address N95 shortages during the COVID-19 pandemic. Anesthesia. 2020;75(8):1022-7. doi: 10.1111/anae.15108. PMID: 32348561

98. Manomaipiboon A, Pupipatpab S, Chomdee P, Boonyapatkul P, Trakarnvanich T. The new silicone N99 half-piece respirator, VJR-NMU N99: A novel and effective tool to prevent COVID-19. PLoS One. 2020;15(12). doi: 10.1371/journal.pone.0237206. PMID: 33382705

99. Martelly E, Li C, Shimada K. Moldable Mask: A Reusable, Hot Water Moldable, Additively Manufactured Mask to Be Used as an N95 Alternative. Materials (Basel). 2021;14(22). doi: 10.3390/ma14227082. PMID: 34832483

100. Meadwell J, Paxman-Clarke L, Terris D, Ford P. In search of a performing seal: Rethinking the design of tight-fitting respiratory protective equipment facepieces for users with facial hair. Saf Health Work. 2019;10(3):275-304. doi: 10.1016/j.shaw.2019.05.001. PMID: 31497325

101. McLeod KER. An exploration of thermomechanical softwood pulp for N95 respiratory mask production. BSc. Thesis, Saint Mary’s University, Halifax, Nova Scotia. 2021. Available from: <https://library2.smu.ca/handle/01/29526?show=full>.

102. Ng WCK, Mbadjeu Hondjeu AR, Syrett A, Caragata R, Rozenberg D, Xiao Z, et al. Subject validation of reusable N95 stop-gap filtering facepiece respirators in COVID-19 pandemic. PLoS One. 2020;15(11):e0242304. doi: 10.1371/journal.pone.0242304. PMID: 33186406

103. Roche AD, McConnell AC, Donaldson K, Lawson A, Tan S, Toft K, et al. Personalised 3D printed respirators for healthcare workers during the COVID-19 pandemic. Front Med Technol. 2022;4:45. doi: 10.3389/fmedt.2022.963541. PMID: 35982716

104. Chughtai AA, Seale H, Rawlinson WD, Kunasekaran M, Macintyre CR. Selection and use of respiratory protection by healthcare workers to protect from infectious diseases in hospital settings. Ann Work Expo Health. 2020;64(4):368-77. doi: 10.1093/annweh/wxaa020c. PMID: 32144412

105. Germonpre P, Van Rompaey D, Balestra C. Evaluation of Protection Level, Respiratory Safety, and Practical Aspects of Commercially Available Snorkel Masks as Personal Protection Devices Against Aerosolized Contaminants and SARS-CoV2. Int J Environ Res Public Health. 2020;17(12). doi: 10.3390%2Fijerph17124347. PMID: 32575366

106. Greig P, Carvalho C, El‐Boghdadly K, Ramessur S. Safety testing improvised COVID‐19 personal protective equipment based on a modified full‐face snorkel mask. Anaesthesia. 2020;75(7):970-1. doi: 10.1111/anae.15085. PMID: 32275770

107. Greig PR, Bradshaw J, Carvalho C, Iwaszko L, Ramessur S, Schumacher J, et al. A crossover study assessing the protective efficacy of improvised snorkel-based improvised respirators. J Intensive Care Soc. 2022;23(3):359-61. doi: 10.1177/1751143721991056. PMID: 36033251

108. Grinshpun SA, Yermakov M, Kano M. Evaluation of AccuFIT 9000: A Novel Apparatus for Quantitative Fit Testing of Particulate Respirators. Ann Work Expo Health. 2020;65(4):458-62. doi: 10.1093/annweh/wxaa116. PMID: 33345279

109. Harmata W, Kamionek D. Rules for fitting filtering gas masks. Sci J Mil Univ Land Forces. 2022;54(2 ):179-95. doi: 10.5604/01.3001.0015.8971.

110. Kechli MK, Lerman J, Ross MM. Modifying a Full-Face Snorkel Mask to Meet N95 Respirator Standards for Use With Coronavirus Disease 2019 Patients. AA Pract. 2020;14(7):e01237. doi: 10.12132FXAA.0000000000001237. PMID: 32539273

111. Kroo L, Kothari A, Hannebelle M, Herring G, Pollina T, Chang R, et al. Modified full-face snorkel masks as reusable personal protective equipment for hospital personnel. PLoS One. 2021;16(1). doi: 10.1371/journal.pone.0244422. PMID: 33439902

112. Nicholson K, Henke-Adams A, Henke DM, Kravitz AV, Gay HA. Modified full-face snorkel mask as COVID-19 personal protective equipment: Quantitative results. HardwareX. 2021;9:e00185. doi: 10.1016/j.ohx.2021.e00185. PMID: 33655089

113. Persing AJ, Sietsema M, Farmer K, Peters TM. Comparing respirator laboratory protection factors measured with novel personal instruments to those from the PortaCount. J Occup Environ Hyg. 2021;18(2):65-71. doi: 10.1080/15459624.2020.1864152. PMID: 33406010

114. Pettinger M, Momeni M, Michaud C, Van Dyck M, Kahn D, Lemaire G. Verification of two Alternative Do-it-yourself Equipment Respirators Seal as COVID-19 Protection (VADERS-CoV): a quality assessment pilot study. Acta Anaesth Belg. 2020;72(2):101-7. doi: 10.56126/72.2.7.

115. Bergman MS, Zhuang Z, Xu SS, Rengasamy S, Lawrence RB, Boutin B, et al. Assessment of respirator fit capability test criteria for full-facepiece air-purifying respirators. J Occup Environ Hyg. 2019;16(7):489-97. doi: 10.1080/15459624.2019.1609006. PMID: 31107187

116. Chehade AEH, Stephenson J, Floyd E, Keddissi J, Abdo T, Thind S, et al. Feasibility study: Proposed alternative to N95 respirator during the personal protective equipment shortage from COVID-19 pandemic. J Emerg Manag. 2021;19(7):193-202. doi: 10.5055/jem.0611. PMID: 34723379

117. Han D-H, Seo H, Kang B-k, Jang H, Kim H, Shim S. Comparisons of Fit Factors Between Two Quantitative Fit Testers (PortaCount vs. MT). Saf Health Work. 2022;13(4):500-6. doi: 10.1016/j.shaw.2022.10.001. PMID: 36579005

118. Rengasamy S, Zhuang Z, Lawrence RB, Boutin B, Yorio P, Horvatin M, et al. Evaluation of total inward leakage for NIOSH-approved elastomeric half-facepiece, full-facepiece, and powered air-purifying respirators using sodium chloride and corn oil aerosols. J Occup Environ Hyg. 2021;18(7):305-13. doi: 10.1080/15459624.2021.1919685. PMID: 34038318

119. Sietsema M, Hamza H, Brosseau LM. Simulated workplace protection factor study of a quarter facepiece elastomeric respirator. J Occup Environ Hyg. 2022;20(1):33-9. doi: 10.1080/15459624.2022.2145014. PMID: 36416662

120. Weng C-H, Kao C-L, Chiu P-W, Huang S-P, Kuo Y-S, Lin Y-Y, et al. A full-face mask for protection against respiratory infections. Biomed Eng Online. 2022;21(1):62. doi: 10.1186/s12938-022-01027-1. PMID: 36064546

121. Clinkard D, Mashari A, Karkouti K, Fedorko L. Evaluation of N95 respirators, modified snorkel masks and low-cost powered air-purifying respirators: a prospective observational cohort study in healthcare workers. Anaesthesia. 2021. doi: 10.1111/anae.15392. PMID: 33470422

122. Convissar D, Berra L, Chang MG, Bittner EA. Personal Protective Equipment N95 Facemask Shortage Quick Fix: The Modified Airway From VEntilatoR Circuit (MAVerIC). Cureus. 2020;12(5). doi: 10.7759/cureus.7914. PMID: 32440384

123. Toigo S, Jacques M, Razek T, Rajda E, Omelon S, Dankoff F, et al. Fit Testing Retrofitted Full-Face Snorkel Masks as a Form of Novel Personal Protective Equipment During the COVID-19 Pandemic. Disaster Med Public Health Prep. 2021:1-16. doi: 10.1017/dmp.2021.133. PMID: 33926606

124. Cass HG, Hanlon GC, McKenzie DP, Harley NS, Kelly DN, Barrett JA. The adequacy of user seal checking for N95 respirators compared to formal fit testing: A multicentred observational study. Aust Crit Care. 2022. doi: 10.1016/j.aucc.2022.08.012. PMID: 36244917

125. Baba H, Ando H, Ikegami K, Sekoguchi S, Shirasaka T, Ogami A. Comparison of respiratory protection during exercise tasks between different methods of wearing replaceable particulate respirators and powered air-purifying respirators. Ind Health. 2022:2021-0268. doi: 10.2486/indhealth.2021-0268. PMID: 35569997

126. Grinshpun SA, Corey J, Yermakov M, Wu B, Strickland KT, Bergman M, et al. New respirator performance monitor (RePM) for powered air-purifying respirators. J Occup Environ Hyg. 2020;17(11-12):538-45. doi: 10.1080/15459624.2020.1814491. PMID: 32941118

127. Kessel J, Saevig CS, Hill WC, Kessel B, Hull MS. An Emergency Powered Air-Purifying Respirator From Local Materials and its Efficacy Against Aerosolized Nanoparticles. Inquiry. 2022;59:469580221087837. doi: 10.1177/00469580221087837. PMID: 35341353

128. McGrath BA, Shelton CL, Gardner A, Coleman R, Lynch J, Alexander PG, et al. Bubble-PAPR: a phase 1 clinical evaluation of the comfort and perception of a prototype powered air-purifying respirator for use by healthcare workers in an acute hospital setting. BMJ Open. 2023;13(5):e066524. doi: 10.1136/bmjopen-2022-066524. PMID: 37156585

129. Nagel J, Gilbert C, Duchesne J. Novel 3D printable powered air purifying respirator for emergency use during PPE shortage of the COVID-19 pandemic: a study protocol and device safety analysis. BMJ Open. 2021;11(8):e049605. doi: 10.1136/bmjopen-2021-049605. PMID: 34446492

130. Goto Y, Jingushi N, Hiraiwa H, Ogawa H, Sakai Y, Kasugai D, et al. The protective effect of tight-fitting powered air-purifying respirators during chest compressions. Am J Emerg Med. 2021;49:172-7. doi: 10.1016/j.ajem.2021.06.012. PMID: 34118785

131. Ng I, Lee K, Kave B, Kluger M, Paynter C, Segal R, et al. HALO CleanSpace PAPR evaluation: Communication, respiratory protection, and usability. Infect Control Hosp Epidemiol. 2023;44(2):295-301. doi: 10.1017/ice.2022.71. PMID: 35361300

132. Rees P, Watson S, Corcoran J, Slade D, Pathmanaban O, Bibi A, et al. Powered air-purifying respirators: a solution to shortage of FFP3 filtering facepiece respirators in the operating theatre. Br J Surg. 2021;108(4):e160-e1. doi: 10.1093/bjs/znab008. PMID: 33778849

133. Sekoguchi S, Shirasaka T, Ando H, Ikegami K, Ogami A. Evaluation of the performance of replaceable particulate and powered air-purifying respirators considering non-recommended wearing methods. Ind Health. 2020;58(6). doi: 10.2486%2Findhealth.2020-0056. PMID: 32863380

134. Sekoguchi S, Ando H, Ikegami K, Yoshitake H, Baba H, Ogami A. Measurement of the workplace protection factor of replaceable particulate and powered air-purifying respirators in Japanese dust-generating occupations. J UOEH. 2022;44(1):15-24. doi: 10.7888/juoeh.44.15. PMID: 35249937

135. Temmesfeld MJ, Gorzkowska-Sobas AA, Hedlund K, Øyen MØ, Kanten L, Grant P, et al. Surgical helmets can be converted into efficient disinfectable powered air-purifying respirators. Am J Infect Control. 2022;50(6):624-30. doi: 10.1016/j.ajic.2021.12.002. PMID: 34958857

136. Xu X, Zhao L, Zhu Y, Du B, Zhu B, Zhang H, et al. Conducting quantitative mask fit tests: application details and affecting factors. Front Public Health. 2023;11. doi: 10.3389/fpubh.2023.1218191.

137. Rowlett JM. The Perceptions of the Quantitative and Qualitative Fit Testing Protocols and Relevance of the Clean-Shaven Guidance for the Current Field of Reusable Elastomeric Half-Mask Respirators. Doctoral dissertation. Indiana University of Pennsylvania. 2021. Available from: <https://www.proquest.com/openview/81c0bc1ae4ea1bc16767325e4240ec44/1?pq-origsite=gscholar&cbl=18750&diss=y>. .
